# Genome-Wide Association Study Identifies Novel Loci Associated with Circulating Phospho- and Sphingolipid Concentrations

**DOI:** 10.1371/journal.pgen.1002490

**Published:** 2012-02-16

**Authors:** Ayşe Demirkan, Cornelia M. van Duijn, Peter Ugocsai, Aaron Isaacs, Peter P. Pramstaller, Gerhard Liebisch, James F. Wilson, Åsa Johansson, Igor Rudan, Yurii S. Aulchenko, Anatoly V. Kirichenko, A. Cecile J. W. Janssens, Ritsert C. Jansen, Carsten Gnewuch, Francisco S. Domingues, Cristian Pattaro, Sarah H. Wild, Inger Jonasson, Ozren Polasek, Irina V. Zorkoltseva, Albert Hofman, Lennart C. Karssen, Maksim Struchalin, James Floyd, Wilmar Igl, Zrinka Biloglav, Linda Broer, Arne Pfeufer, Irene Pichler, Susan Campbell, Ghazal Zaboli, Ivana Kolcic, Fernando Rivadeneira, Jennifer Huffman, Nicholas D. Hastie, Andre Uitterlinden, Lude Franke, Christopher S. Franklin, Veronique Vitart, DIAGRAM Consortium, Christopher P. Nelson, Michael Preuss, CARDIoGRAM Consortium, Joshua C. Bis, Christopher J. O'Donnell, Nora Franceschini, CHARGE Consortium, Jacqueline C. M. Witteman, Tatiana Axenovich, Ben A. Oostra, Thomas Meitinger, Andrew A. Hicks, Caroline Hayward, Alan F. Wright, Ulf Gyllensten, Harry Campbell, Gerd Schmitz, on behalf of the EUROSPAN consortium

**Affiliations:** 1 Genetic Epidemiology Unit, Departments of Epidemiology and Clinical Genetics, Erasmus University Medical Center, Rotterdam, The Netherlands; 2 Centre for Medical Sytems Biology, Leiden, The Netherlands; 3 Netherlands Consortium for Healthy Aging, Netherlands Genomics Initiative, Leiden, The Netherlands; 4 Institute for Clinical Chemistry and Laboratory Medicine, University Hospital Regensburg, Regensburg, Germany; 5 Center for Biomedicine, European Academy Bozen/Bolzano (EURAC), Bolzano, Italy; 6 Department of Neurology, General Central Hospital, Bolzano, Italy; 7 Department of Neurology, University of Lubeck, Lubeck, Germany; 8 Centre for Population Health Sciences, The University of Edinburgh Medical School, Edinburgh, United Kingdom; 9 Department of Genetics and Pathology, Rudbeck Laboratory, Uppsala University, Uppsala, Sweden; 10 Institute for Clinical Medical Research, University Hospital “Sestre Milosrdnice”, Zagreb, Croatia; 11 Croatian Centre for Global Health, Faculty of Medicine, University of Split, Split, Croatia; 12 Institute of Cytology and Genetics, Siberian Division of the Russian Academy of Sciences, Novosibirsk, Russia; 13 Department of Epidemiology, Erasmus University Medical Center, Rotterdam, The Netherlands; 14 Groningen Bioinformatics Centre, Groningen Biomolecular Sciences and Biotechnology Institute, University of Groningen, Groningen, The Netherlands; 15 Wellcome Trust Sanger Institute, Wellcome Trust Genome Campus, Hinxton, United Kingdom; 16 Andrija Stampar School of Public Health, Faculty of Medicine, University of Zagreb, Zagreb, Croatia; 17 Department of Internal Medicine, Erasmus University Medical Center, Rotterdam, The Netherlands; 18 Medical Research Council Human Genetics Unit, Institute of Genetics and Molecular Medicine, Edinburgh, United Kingdom; 19 Genetics Department, University Medical Centre Groningen and University of Groningen, Groningen, The Netherlands; 20 Department of Cardiovascular Science, University of Leicester, Leicester, United Kingdom; 21 Institut fur Medizinische Biometrie und Statistik and Medizinische Klinik II, Universitat zu Lubeck, Lubeck, Germany; 22 Cardiovascular Health Research Unit and Department of Medicine, University of Washington, Seattle, Washington, United States of America; 23 National Heart, Lung, and Blood Institute's Framingham Heart Study, Framingham, Massachusetts, United States of America; 24 Cardiology Division, Department of Medicine, Massachusetts General Hospital, Harvard Medical School, Boston, Massachusetts, United States of America; 25 Department of Epidemiology, University of North Carolina at Chapel Hill, Chapel Hill, North Carolina, United States of America; 26 Department of Clinical Genetics, Erasmus University Medical Center, Rotterdam, The Netherlands; 27 Institut for Human Genetics, Helmholtz-Zentrum München, Neuherberg, Germany; 28 Institute of Human Genetics, Technische Universität München, München, Germany; 29 Munich Heart Alliance, Munich, Germany; Georgia Institute of Technology, United States of America

## Abstract

Phospho- and sphingolipids are crucial cellular and intracellular compounds. These lipids are required for active transport, a number of enzymatic processes, membrane formation, and cell signalling. Disruption of their metabolism leads to several diseases, with diverse neurological, psychiatric, and metabolic consequences. A large number of phospholipid and sphingolipid species can be detected and measured in human plasma. We conducted a meta-analysis of five European family-based genome-wide association studies (N = 4034) on plasma levels of 24 sphingomyelins (SPM), 9 ceramides (CER), 57 phosphatidylcholines (PC), 20 lysophosphatidylcholines (LPC), 27 phosphatidylethanolamines (PE), and 16 PE-based plasmalogens (PLPE), as well as their proportions in each major class. This effort yielded 25 genome-wide significant loci for phospholipids (smallest *P*-value = 9.88×10^−204^) and 10 loci for sphingolipids (smallest *P*-value = 3.10×10^−57^). After a correction for multiple comparisons (*P*-value<2.2×10^−9^), we observed four novel loci significantly associated with phospholipids (*PAQR9*, *AGPAT1*, *PKD2L1*, *PDXDC1*) and two with sphingolipids (*PLD2* and *APOE*) explaining up to 3.1% of the variance. Further analysis of the top findings with respect to within class molar proportions uncovered three additional loci for phospholipids (*PNLIPRP2*, *PCDH20*, and *ABDH3*) suggesting their involvement in either fatty acid elongation/saturation processes or fatty acid specific turnover mechanisms. Among those, 14 loci (*KCNH7*, *AGPAT1*, *PNLIPRP2*, *SYT9*, *FADS1-2-3*, *DLG2*, *APOA1*, *ELOVL2*, *CDK17*, *LIPC*, *PDXDC1*, *PLD2*, *LASS4*, and *APOE*) mapped into the glycerophospholipid and 12 loci (*ILKAP*, *ITGA9*, *AGPAT1*, *FADS1-2-3*, *APOA1*, *PCDH20*, *LIPC*, *PDXDC1*, *SGPP1*, *APOE*, *LASS4*, and *PLD2*) to the sphingolipid pathways. In large meta-analyses, associations between *FADS1-2-3* and carotid intima media thickness, *AGPAT1* and type 2 diabetes, and *APOA1* and coronary artery disease were observed. In conclusion, our study identified nine novel phospho- and sphingolipid loci, substantially increasing our knowledge of the genetic basis for these traits.

## Author Summary

Phospho- and sphingolipids are integral to membrane formation and are involved in crucial cellular functions such as signalling, membrane fluidity, membrane protein trafficking, neurotransmission, and receptor trafficking. In addition to severe monogenic diseases resulting from defective phospho- and sphingolipid function and metabolism, the evidence suggests that variations in these lipid levels at the population level are involved in the determination of cardiovascular and neurologic traits and subsequent disease. We took advantage of modern laboratory methods, including microarray-based genotyping and electrospray ionization tandem mass spectrometry, to hunt for genetic variation influencing the levels of more than 350 phospho- and sphingolipid phenotypes. We identified nine novel loci, in addition to confirming a number of previously described loci. Several other genetic regions provided substantial evidence of their involvement in these traits. All of these loci are strong candidates for further research in the field of lipid biology and are likely to yield considerable insights into the complex metabolic pathways underlying circulating phospho- and sphingolipid levels. Understanding these mechanisms might help to illuminate factors leading to the development of common cardiovascular and neurological diseases and might provide molecular targets for the development of new therapies.

## Introduction

Phospho- and sphingolipids are present in all eukaryotic cell membranes and contribute to organelle structure and signalling events that influence cell behaviour and function [1]–[3]. Phosphatidylcholines (PC), phosphatidylethanolamines (PE), lysophosphatidylcholines (LPC) and PE-based plasmalogens (PLPE) are major classes of phospholipids that play an important role in several key processes such as cell survival and inflammation [4]–[6]. Sphingolipids are also essential components of plasma membranes and endosomes and are believed to play critical roles in cell surface protection, protein and lipid transport and sorting, and cellular signalling cascades [7]. In plasma, PC, PE and sphingomyelin (SPM) are included in the structure of lipoproteins; they constitute more than two-thirds of the total phospholipid content in HDL-C and LDL-C, as well as in platelets [8], [9]. Remarkable differences in plasma lipoprotein acceptor affinities for the phospholipids exist (LDL-C is the major acceptor for SPM, whereas HDL-C is the predominant acceptor for PC) [9]. Altered concentrations of circulating phospholipids have been implicated in the pathology of type 2 diabetes, dyslipidemia and cardiovascular disease [10]–[15], as well as a wide range of other common diseases including dementia and depression [16].

Identifying genetic variants that influence phospho- and sphingolipid concentrations will be an important step towards understanding pathways contributing to common human disease. Earlier studies of these metabolites identified a number of genetic loci associated with their levels in blood [17]–[19]. We conducted a meta-analysis of genome-wide association studies (GWAS) on plasma levels of 24 SPMs, 9 ceramides (CER), 57 PCs, 20 LPCs, 27 PEs and 16 PLPEs in five European populations: (1) the Erasmus Rucphen Family (ERF) study, conducted in the Netherlands, (2) the MICROS study from the Tyrol region in Italy, (3) the Northern Swedish Population Health Survey (NSPHS) in Norrbotten, Sweden, (4) the Orkney Complex Disease Study (ORCADES) in Scotland, and (5) the CROAS (CROATIA_Vis) study conducted on Vis Island, Croatia.

The top findings were further analysed by adjusting for plasma HDL-C, LDL-C, TG and TC levels. The influences of these top hits on within class lipid ratios were also assessed, to help elucidate potential mechanisms. Finally, the variants that were associated with plasma phospho- and sphingolipid levels were tested for association with carotid intima media thickness (IMT), type 2 diabetes (T2DM), and coronary-artery disease (CAD) using large consortia meta-analysis results.

## Results

**Figure 1:**
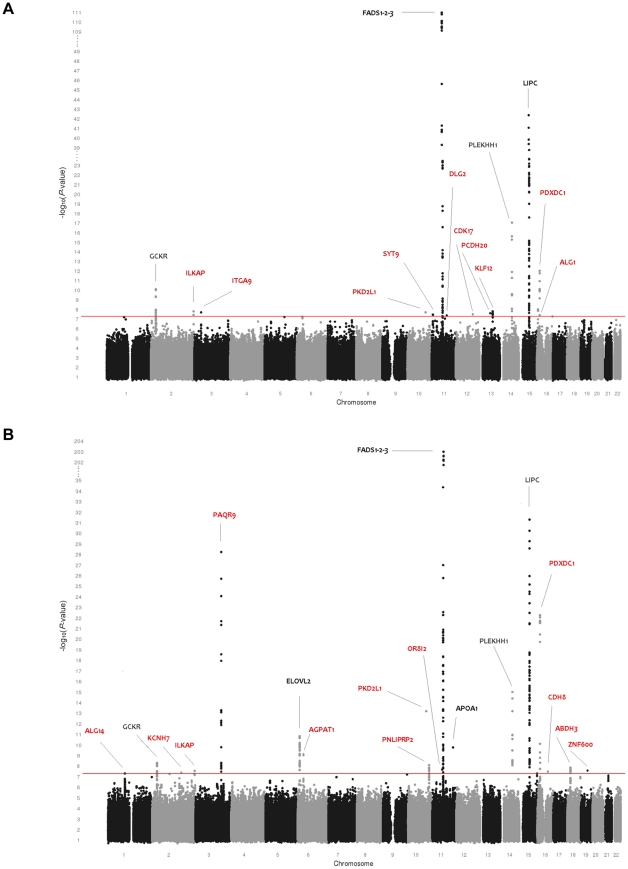
Genome-wide association results for 115 phospholipid species. (A) Genome-wide association results for plasma levels of 115 phospholipid species. (B) Genome-wide association results for within-class proportions of 115 plasma phospholipid species. Manhattan plots show the combined association signals (−log_10_(*P*-value)) on the y-axis versus SNPs according to their position in the genome on the x-axis (NCBI build 36). Novel genes are represented in red, while previously known loci are represented in black.

**Figure 2:**
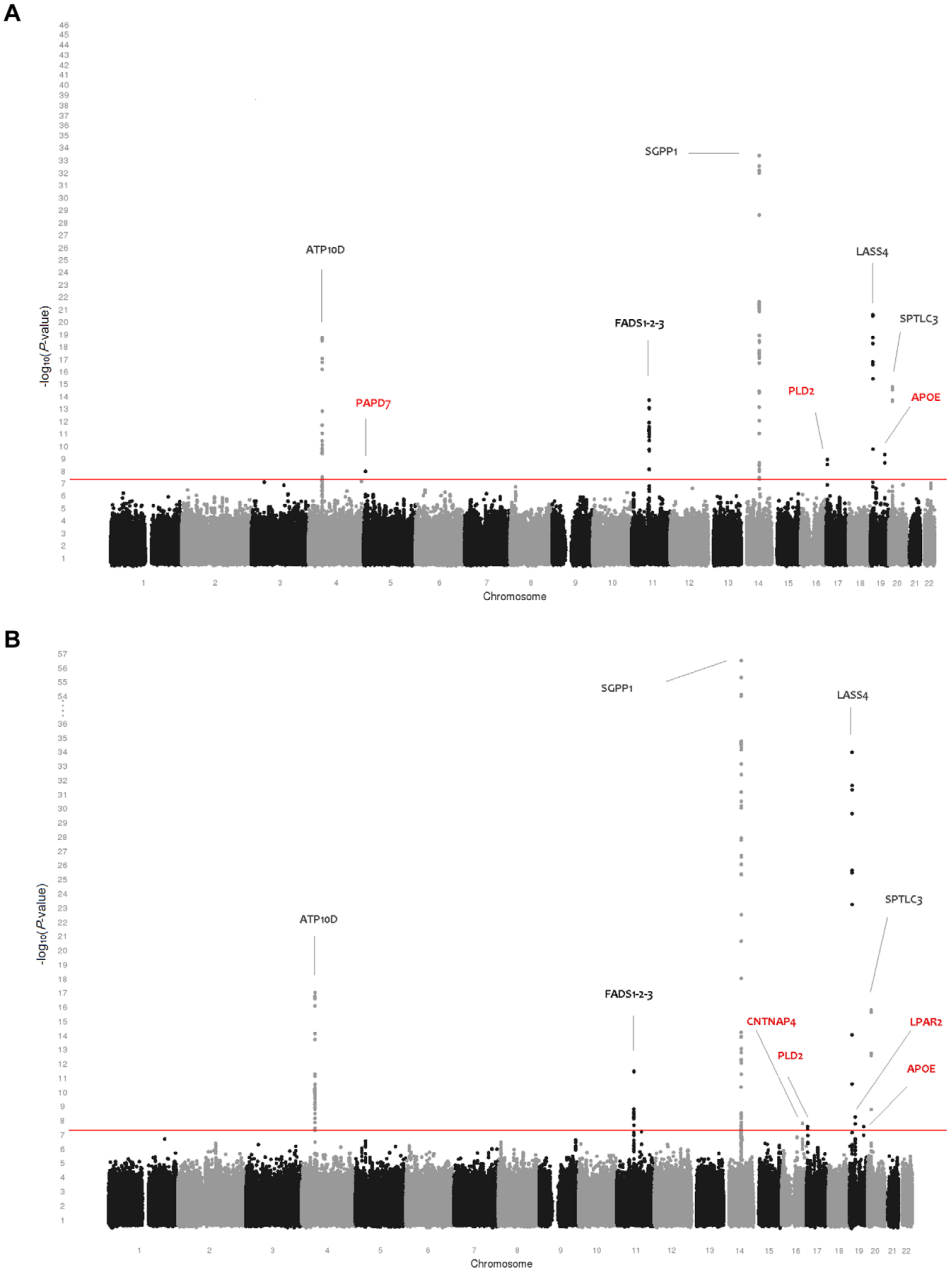
Genome-wide association results for 33 sphingolipid species. (A) Genome-wide association results for plasma levels of 33 sphingolipid species. (B) Genome-wide association results for within-class proportions of 33 sphingolipid species. Manhattan plots show the combined association signals (−log_10_(*P*-value)) on the y-axis versus SNPs according to their position in the genome on the x-axis (NCBI build 36). Novel genes are represented in red, while previously known loci are represented in black.

Table 1 provides an overview of the study populations. The mean age, gender ratio and mean values of major classes of phospho- and sphingolipids were comparable among the 5 populations. Means for the individual species are presented in Table S1. Figure 1A and 1B shows the combined Manhattan plot for the meta-analyses of the absolute values and proportions of all phospholipid traits, respectively; Figure 2A and 2B provides the same for the sphingolipids. Out of 357 meta-analyses performed, 202 outcomes yielded genome-wide significant findings, most of which were located around two genes, *FADS* and *LIPC*, which were identified previously [17], [19] as key lipid regulators and are associated with a large number of species (Table 2 and Table 3). Q-Q plots for the lipid GWAS that yielded significant associations are provided in Figure S1.

**Table 1:** Study populations.

	ERF	MICROS	NSPHS	ORCADES	VIS
**N (4034)**	800	1086	654	714	780
**% Female (56.4)**	60.13	56.63	52.75	53.36	57.94
**Age**	49.65 (15.20)	45.26 (16.08)	46.98 (20.70)	53.59 (15.71)	56.55 (15.36)
**Total SPM**	532.21 (109.39)	587.03 (114.19)	516.98 (121.98)	468.25 (96.4)	499.01 (105.26)
**Total PC**	2198.55 (444.36)	2527.88 (457.25)	2249.18 (493.67)	1941.73 (372.96)	2066.56 (427.91)
**Total PLPE**	52.68 (14.91)	58.21 (17.05)	62.28 (24.28)	42.15 (13.16)	55.60 (15.39)
**Total PE**	37.22 (16.69)	37.15 (18.12)	20.58 (7.25)	25.02 (11.19)	34.34 (14.55)
**Total CER**	8.45 (1.95)	9.26 (2.12)	9.30 (2.52)	7.12 (1.83)	9.05 (2.23)

Values for age and lipid concentrations are presented as mean (standard deviation).

**Table 2:** Variants significantly associated with circulating phospholipid levels and proportions.

Variants significantly associated with circulating phospholipid levels.									
SNP	Chromosome	Position	Gene	Distance (kb)	*P*-value_Nominal_	*P*-value_Corrected_	% Explained variance	MAF	Species associated
rs4666002 *	2p23.3	27840640	GCKR	97	7.48×10^−11^	1.72×10^−09^	1.1–0.7	0.21	PC 34∶4
rs12472274⋈	2q37.3	239095422	ILKAP	intronic	1.52×10^−08^	3.49×10^−07^	0.8	0.18	PC 40∶3
rs197770⋈	3p22.2	37515827	ITGA9	intronic	1.95×10^−08^	4.49×10^−07^	0.8	0.14	PLPE 18∶0/22∶6
rs603424* ⋈ <$>\vskip -3\raster="rg1"<$>	10q24.31	102075479	PKD2L1	intronic	6.41×10^−14^	1.47×10^−12^	1.4	0.17	LPC 16∶1
rs10769780⋈	11p15.4	7367095	SYT9	intronic	3.31×10^−08^	7.61×10^−07^	0.8	0.38	PC O 42∶6
rs102275*	11q12.2	61557803	FADS1-2-3	21	9.88×10^−204^	2.27×10^−202^	0.8–23.0	0.30	PE 32∶1, PE 34∶1, PE 34∶2, PE 34∶3. See Text S2 for the full list.
rs17148090⋈	11q14.1	85017476	DLG2	intronic	4.14×10^−08^	9.52×10^−07^	0.7	0.05	PC O 42∶5
rs12423247⋈	12q23.1	96858362	CDK17	22	3.09×10^−08^	7.11×10^−07^	0.8	0.04	PC O 42∶6
rs7337573⋈ <$>\vskip -3\raster="rg1"<$>	13q21.2	61513669	PCDH20	470	2.39×10^−08^	5.49×10^−07^	0.8	0.00	PC 32∶1
rs17718828⋈	13q22.1	75128114	KLF12	422	1.49×10^−08^	3.43×10^−07^	0.8	0.05	PC O 42∶5
rs1077989*	14q24.1	67975822	PLEKHH1	23	9.29×10^−18^	2.14×10^−16^	0.8–1.8	0.47	PC O 36∶5, PC O 32∶1
rs10468017*	15q21.3	58678512	LIPC	20	6.59×10^−43^	1.52×10^−41^	0.8–4.7	0.30	PE 36∶4, PE 34∶2, PE 38∶5. See Text S2 for the full list.
rs870288⋈	16p13.3	5585852	ALG1	410	9.57×10^−09^	2.20×10^−07^	0.8	0.24	PC 30∶1
rs4500751* ⋈	16p13.11	15140211	PDXDC1	intronic	5.59×10^−23^	1.29×10^−21^	0.8–2.4	0.30	LPC 20∶3, PC 38∶3

Variants significantly associated with circulating phospholipid proportions.									
SNP	Chromosome	Position	Gene	Distance (kb)	*P*-value_Nominal_	*P*-value_Corrected_	% Explained variance	MAF	Species associated
rs9437689⋈	1p21.3	95549536	ALG14	11	4.92×10^−08^	1.13×10^−06^	0.7	0.46	%LPC 18∶0
rs4666002 *	2p23.3	27840640	GCKR	97	7.48×10^−11^	1.72×10^−09^	1.1–0.7	0.21	%PC 34∶4
rs1424760⋈	2q24.3	163781787	KCNH7	85	4.11×10^−08^	9.46×10^−07^	0.7	0.29	%PC O 36∶5
rs12472274⋈	2q37.3	239095422	ILKAP	intronic	1.52×10^−08^	3.49×10^−07^	0.8	0.18	%PC 40∶3
rs9832727* ⋈	3q23	142649110	PAQR9	18	5.88×10^−29^	1.35×10^−27^	0.8–3.1	0.31	%PE 34∶1, %PE 36∶1, % mono-unsaturated PEs
rs17606561*	6p24.2	10982359	ELOVL2	3′UTR	1.49×10^−11^	3.44×10^−10^	0.8–1.1	0.21	%PC 38∶5, %PE 38∶5, %LPC 22∶5
rs1061808* ⋈	6p21.32	32136547	AGPAT1	3′UTR	7.78×10^−10^	1.79×10^−08^	0.9	0.45	%PC 32∶0
rs603424* ⋈ <$>\vskip -3\raster="rg1"<$>	10q24.31	102075479	PKD2L1	intronic	6.41×10^−14^	1.47×10^−12^	1.4	0.17	%LPC 16∶1
rs10885997⋈ <$>\vskip -3\raster="rg1"<$>	10q25.3	118397971	PNLIPRP2	synonymous	7.88×10^−09^	1.81×10^−07^	0.8	0.38	%PC 36∶1
rs2945816⋈	11q12.1	55862091	OR8I2	0.3	2.15×10−^08^	4.93×10^−07^	0.8	0.01	%PC 26∶0
rs102275*	11q12.2	61557803	FADS1-2-3	21	9.88×10^−204^	2.27×10^−202^	0.8–23.0	0.30	%PC 36∶4, %PC 38∶4, %PE 38∶4. See Text S2 for the full list.
rs964184*	11q23.3	116648917	APOA5	11	1.71×10^−10^	3.93×10^−09^	1.0	0.13	PC/SPM, %PC O 34∶1, %PC O 32∶0, % poly-unsaturated PCs
rs1077989*	14q24.1	67975822	PLEKHH1	23	9.29×10^−18^	2.14×10^−16^	0.8–1.8	0.47	%PC O 36∶5
rs10468017*	15q21.3	58678512	LIPC	20	6.59×10^−43^	1.52×10^−41^	0.8–4.7	0.30	%PE 32∶0, %PE 32∶2, %PE 34∶0. See Text S2 for the full list.
rs4500751* ⋈	16p13.11	15140211	PDXDC1	intronic	5.59×10^−23^	1.29×10^−21^	0.8–2.4	0.30	%LPC 20∶3, %PC 34∶2, %PC 36∶3, %PC 38∶3, %PE 38∶3,
rs9932186⋈	16q21	60314656	CDH8	1300	3.33×10^−08^	7.67×10^−07^	0.8	0.24	%saturated LPCs
rs11662721⋈ <$>\vskip -3\raster="rg1"<$>	18q11.2	19261413	ABHD3	intronic	1.36×10^−08^	3.13×10^−07^	0.7–0.8	0.26	%PC 32∶2
rs10404486⋈	19q13.41	53284135	ZNF600	intronic	8.53×10^−09^	1.96×10^−07^	0.8	0.16	PC/LPC

*P*-value_Corrected_: Genome-wide association p-value after adjustment for number of independent vectors; MAF: Minor Allele Frequency.

*Loci significantly associated with lipid levels.

**⋈:** Loci associated with phospholipids for the first time.

<$>\vskip -3\raster="rg1"<$>Loci significantly (*P*-value<2.2×10^−9^) associated with within-class phospholipid ratios.

**Table 3:** Variants significantly associated with circulating sphingolipid levels and proportions.

Variants significantly associated with circulating sphingolipid levels.									
SNP	Chromosome	Position	Gene	Distance (kb)	*P*-value_Nominal_	*P*-value_Corrected_	% Explained variance	MAF	Species associated
rs13106975*	4p12	47551863	ATP10D	intronic	1.93×10^−19^	4.45×10^−18^	0.7–2.0	0.20	Glu-CER 16∶0, Glu-CER 24∶1, Glu-CER
rs1566039⋈	5p15.31	6821914	PAPD7	62	1.09×10^−08^	2.51×10^−07^	0.8	0.40	SPM 17∶0
rs174479*	11q12.2	61678754	FADS-1-2-3	17	1.99×10^−14^	4.58×10^−13^	0.8–1.5	0.49	SPM 16∶1, SPM 18∶1, SPM 20∶1, SPM 22∶1
rs17101394*	14q23.2	64232386	SGPP1	37	3.10×10^−57^	7.13×10^−56^	0.7–6.3	0.15	SPM 14∶0, SPM 15∶0, SPM dih16∶0
rs12051548* ⋈ <$>\vskip -3\raster="rg1"<$>	17p13.2	4683035	PLD2	24	1.21×10^−09^	2.78×10^−08^	0.7–0.9	0.03	SPM 23∶0
rs7258249*	19p13.2	8271721	LASS4	2	1.09×10^−34^	2.52×10^−33^	0.8–3.7	0.48	CER 20∶0, SPM 20∶1, SPM 20∶0, SPM 18∶1, SPM 18∶0
rs7259004* ⋈	19q13.32	45432557	APOE-C1-C2-C4	9	4.89×10^−10^	1.12×10^−08^	0.8–1.0	0.11	SPM 24∶0, SPM 22∶0
rs680379*	20p12.1	12969400	SPTLC3	20	1.61×10^−16^	3.70×10^−15^	0.8–1.7	0.29	CER 22∶0, CER 23∶0, CER 24∶1, CER 24∶0, saturated-CER, total CER, unsaturated CER, Glu-CER, SPM 17∶0

Variants significantly associated with circulating sphingolipid proportions.									
SNP	Chromosome	Position	Gene	Distance (kb)	*P*-value_Nominal_	*P*-value_Corrected_	% Explained variance	MAF	Species associated
rs13106975*	4p12	47551863	ATP10D	intronic	1.93×10^−19^	4.45×10^−18^	0.7–2.0	0.20	%Glu-CER 16∶0, %Glu-CER
rs174479*	11q12.2	61678754	FADS-1-2-3	17	1.99×10^−14^	4.58×10^−13^	0.8–1.5	0.49	% SPM 16∶1, SPM/CER
rs17101394*	14q23.2	64232386	SGPP1	37	3.10×10^−57^	7.13×10^−56^	0.7–6.3	0.15	% SPM 14∶0, %SPM 22∶1, %SPM 23∶0,
rs4485401⋈	16q23.1	76566157	CNTNAP4	intronic	1.60×10^−08^	3.68×10^−07^	0.8	0.45	% Glu-CER 24∶1, %Glu-CER
rs12051548* ⋈ <$>\vskip -3\raster="rg1"<$>	17p13.2	4683035	PLD2	24	1.21×10^−09^	2.78×10^−08^	0.7–0.9	0.03	% SPM 23∶0
rs2304130⋈	19p13.11	19789528	LPAR2	49	5.85×10^−09^	1.34×10^−07^	0.8	0.06	% CER 18∶0
rs7258249*	19p13.2	8271721	LASS4	2	1.09×10^−34^	2.52×10^−33^	0.8–3.7	0.48	%CER 20∶0,%SPM 20∶1, %SPM 20∶0, %SPM 24∶1, %SPM 18∶1, %SPM 18∶0
rs7259004* ⋈	19q13.32	45432557	APOE-C1-C2-C4	9	4.89×10^−10^	1.12×10^−08^	0.8–1.0	0.11	%SPM 22∶0
rs680379*	20p12.1	12969400	SPTLC3	20	1.61×10^−16^	3.70×10^−15^	0.8–1.7	0.29	%CER 16∶0, %Glu-CER 16∶0, SPM/CER, % SPM 20∶0, %SPM 16∶1, %SPM 17∶0

*P*-value_Corrected_: Genome-wide association p-value after adjustment for number of independent vectors; MAF: Minor Allele Frequency.

*Loci significantly associated to lipid levels after Bonferroni correction.

**⋈:** Loci associated to sphingolipids for the first time.

<$>\vskip -3\raster="rg1"<$>Loci significantly (*P*-value<2.2×10^−9^) associated to within class sphingolipid ratios.

### Phospholipids

As shown in Table 2, 25 loci were nominally associated (*P*-value<5×10^−8^) with absolute plasma levels and/or proportions of the phospholipid species. Among those loci, previously reported relationships between the *FADS1*, *LIPC*, *PLEKHH1*, *GCKR*, *APOA1-5*, and *ELOVL2* loci and phospholipids were successfully replicated [17], [19]. Four novel genome-wide significant loci were also detected after a multiple testing correction to adjust for the approximate number of independent genotypes and phenotypes (n = 23) studied (*P*-value<2.2×10^−9^). These included *PAQR9* on 3q23 (associated with %PE 34∶1 and %PE 36∶1), *AGPAT1* on 6p21.32 (associated with PC 32∶0), *PKD2L1* on 10q24.31 (LPC 16∶1), and *PDXDC1* on 16p13.11 (LPC 20∶3, PC 34∶2, PC 36∶3 and PC 38∶3). Fifteen additional regions provided suggestive evidence of association (2.2×10^−9^<*P*-value<5×10^−8^) with phospholipids including the *PNLIPRP2* locus, associated with %PC 36∶1; *ZNF600* with PC/LPC ratio; *ALG1* with PC 30∶1; *ABHD3* with %PC 32∶2; *KLF12* and *DLG2*, both associated with PC O 42∶5; *ILKAP* with PC 40∶3 and %PC 40∶3; *ITGA9* with PLPE 18∶0/22∶6; *OR8I2* with %PC 26∶0; *PCDH20* with PC 32∶1; *CDK17* and *SYT9*, both associated with PC O 42∶6; *CDH8* with the proportion of saturated LPC; *KCNH7* with %PC O 36∶5; and *ALG14* with %LPC 18∶0. Regional association plots for all phospholipid loci are presented in Figure S2.

Many of the genome-wide significant and suggestive loci in Table 2 were associated with the percentage of each lipid molecule within its own class (mol%) rather than to absolute values. Single SNP analysis of ratios showed that rs4500751 *(PDXDC1)* was strongly associated with PC 36∶3/PC 34∶2 (*P*-value = 4.37×10^−25^) and LPC 20∶3/LPC 16∶1 (*P*-value = 6.84×10^−23^) (Table S2). Further, rs11662721 *(ABHD3)* was associated with the ratio of PC 32∶2 to PC 36∶2 (*P*-value = 9.35×10^−10^), but also to PC 36∶3 (*P*-value = 1.80×10^−9^) and PC 38∶3 (*P*-value = 6.71×10^−9^). rs9437689 *(ALG14)* and rs603424 *(PKD2L1)* were associated with the ratios of LPC 16∶0 to LPC 18∶0 (*P*-value = 2.70×10^−8^) and LPC 16∶1 (*P*-value = 2.25×10^−15^), respectively. SNP rs10885997 *(PNLIPRP2)* was associated with PC 36∶1/PC 34∶1 (*P*-value = 3.28×10^−10^) and PC 36∶1/PC 34∶3 (*P*-value = 1.15×10^−9^). SNP rs7337585 *(PCDH20)* was associated with the ratio of PC 32∶1 to several ether-bound PC species (the strongest association was with PC 32∶1/PC O 32∶0; *P*-value = 1.82×10^−18^) and, finally, rs2945816 *(OR8I2)* was associated with the ratio of PC 26∶0 to several long chain PCs (the strongest association was with PC 26∶0/PC 36∶1; *P*-value = 2.93×10^−9^).

### Sphingolipids

Table 3 shows the 10 loci that were associated with either absolute plasma levels (panel A) or percentages (panel B) of sphingomyelin species or ceramides. Among those loci, 5 *(ATP10D*, *FADS1-3*, *SGPP1*, *SPTLC3*, *LASS4)* were previously described in genome-wide analyses [18], [19]. These loci retained significance after adjustment for the number of genotypes and phenotypes tested. In addition, five novel loci were identified at a nominal *P*-value of 5×10^−8^ (*PAPD7*, *CNTNAP4*, *PLD2*, *LPAR2*, and *APOE*). Two of these, *APOE* on 19q13.32 (associated with SPM 24∶0 and SPM 22∶0) and *PLD2* on 17p13.2 (associated with SPM 23∶0), remained significant after correction for the number of phenotypes tested. The other three showed suggestive evidence of association (2.2×10^−9^<*P*-value<5×10^−8^) to either sphingomyelins or ceramides: *PAPD7* on 5p15.31 (SPM 17∶0), the *CNTNAP4* region on 16q23.1 (% Glu-CER 24∶1, %Glu-CER) and *LPAR2* on 19p13.11 (%C 18∶0). Regional association plots for the sphingolipid loci are presented in Figure S3.

When studying the ratios of the index lipid to the other lipids within the same class, the strongest association for rs12051548 *(PLD2)* was found with the SPM 23∶0/SPM 16∶1 ratio (*P*-value = 2.43×10^−10^). SNP rs7259004 in the *APOE* locus was strongly associated with the ratio of SPM 24∶0 to SPM 24∶2 (*P*-value = 5.11×10^−9^) and SPM 16∶1 (*P*-value = 4.79×10^−8^) but also with the ratio of SPM 22∶0 to the same lipids (SPM 24∶2: *P*-value = 2.91×10^−8^ and SPM 16∶1: *P*-value = 1.98×10^−8^).

### HDL-C, LDL-C, TG, and TC

As a point of reference, the genome-wide significant findings (*P*-value<5×10^−8^) from the GWAS of TC, LDL-C, HDL-C, and TG in these samples are provided in Table S3. *CETP* was associated with HDL-C levels (*P*-value = 8.5×10^−20^), *APOE* was associated with LDL-C (*P*-value = 9.2×10^−26^) and TC levels (*P*-value = 4.6×10^−11^). *APOA1-5* (*P*-value = 1.6×10^−8^) and *PDCD11* (*P*-value = 2.7×10^−10^) were associated with TG levels. Except for the *PDCD11* locus, these associations have all been previously reported [20].

To determine if the associations of the phospho- and sphingolipid loci were mediated by these major classes of plasma lipoproteins, conditional analyses were performed. Table S4 shows the effect size, standard error, and *P*-values for the genome-wide significant loci when adjusted for HDL-C, LDL-C, TG and TC. Only the association of the *APOE* locus (rs7259004) with SPMs was greatly affected by the incorporation of LDL-C and TC. No other major differences were observed in effect size or *P*-value.

### Pathway analyses

Additionally, we investigated whether the genes from the GWAS fit into previously known sphingolipid and glycerophospholipid pathways, which are available among the canonical pathways from various data bases provided by ConsensusPathDB [21]. By testing for enrichment of known pathways, glycerolipid metabolism (*P*-value = 0.002; KEGG), chylomicron-mediated lipid transport (*P*-value = 0.003; Reactome), triglyceride biosynthesis (*P*-value = 0.006; Reactome), metabolism of lipids and lipoproteins (*P*-value = 0.002; Reactome) and biosynthesis of the N-glycan precursor (*P*-value = 0.005; Reactome) were found to be significantly enriched among the phospholipid related loci. Considering the sphingolipid associated loci, the same analysis implicated the sphingolipid metabolism (*P*-value = 1.0×10^−5^; Reactome), metabolism of lipids and lipoproteins (*P*-value = 1.0×10^−5^; Reactome), and LPA receptor mediated events (*P*-value = 0.002; PID) pathways. These analyses suggested that, among genes from the same locus, *SRD5A1* is a more likely candidate than *PAPD7* and *LPAR2* is a more likely candidate than neighbouring *ZNF101* and *ATP13A1* (Tables S5 and S6).

Figure S4 places all of the nearest, or most likely, genes from genome-wide significant and suggestive loci in the Ingenuity glycerophospholipid metabolism pathway [22]. Of the 25 loci associated with phospholipids at a nominal *P*-value<5×10^−8^, 13 genes *(KCNH7*, *AGPAT1*, *PNLIPRP2*, *SYT9*, *FADS2*, *DAGLA*, *DLG2*, *APOA1*, *APOC3*, *ELOVL2*, *CDK17*, *LIPC* and *PLA2G10)* from 11 loci can be mapped to the glycerophospholipid metabolism pathway; among the 10 loci associated with sphingomyelins or ceramides, 6 genes (*FADS2*, *DAGLA*, *PLD2*, *LASS4*, *APOE*, *APOC2*) from 4 loci can be mapped to the same pathway (Figure S4). Figure S5 maps the same genes onto the Ingenuity sphingolipid metabolism pathway. Of the 10 sphingomyelin or ceramide loci, 9 genes from 5 loci (*FADS1*, *FADS2*, *C11orf10*, *SGPP1*, *APOE*, *APOC1*, *APOC2*, ,*LASS4*, and *PLD2*) can be placed in this pathway, as was the case for 12 genes from 8 loci implicated in phospholipids (*ILKAP*, *ITGA9*, *AGPAT1*, *FADS1*, *FADS2*, *C11orf10*, *APOA1*, *APOA5*, *APOC3*, *PCDH20*, *LIPC*, and *PDXDC1*).

### Association with IMT, T2DM, and CAD risk

The top 35 SNPs were assessed for association with IMT, T2DM, and CAD using the GWAS results from the CHARGE [23], DIAGRAM [24] and CARDIoGRAM [25] consortia, respectively. For IMT, we observed a significant association (*P*-value = 7×10^−4^) with the *FADS1-2-3* locus SNP rs102275 (Table S7). rs1061808, located in the *HLA* region on chromosome 6, and two SNPs from the *FADS1-2-3* region (rs174479 and rs102275) were associated with T2DM risk (nominal *P*-value<0.05) (Table S8). rs964184 from the *APOA1-5* region was previously reported to be associated with CAD risk (*P*-value = 8.02×10^−10^) by the CARDIoGRAM meta-analysis study (Table S9). For all three outcomes, the observed *P*-value distribution differed significantly from that expected under the null hypothesis (Kolmogorov Smirnov *P*-value≤3.3×10^−16^; Figure S6).

## Discussion

This genome-wide association study of more than 350 phospho- and sphingolipid measurements in five European populations yielded 25 loci associated with phospholipids and 10 loci associated with sphingolipids using a nominal *P-*value of 5×10^−8^. After correction for the number of independent phenotypes, the novel genome-wide significant loci included: *PAQR9*, *AGPAT1*, *PKD2L1*, *PDXDC1*, *APOE* and *PLD2*. In addition, further analysis of suggestive SNPs with lipid ratios showed significant association for an additional 3 loci *(ABDH3*, *PNLIPRP2*, and *PCDH20)*.

The strongest association in the *PAQR9* locus was observed between rs9832727 and the proportion of mono-unsaturated PEs, especially with the ratios PE 34∶1/PE 34∶2 and PE 36∶1/PE 36∶2. The protein coded by*PAQR9* is an integral membrane receptor and functions as receptor for the hormone adiponectin, suggesting a molecular link with obesity and T2DM [26]. However, we did not observe an association between T2DM risk and this variant.

In the *AGPAT1* locus, rs1061808 was associated with the proportion of PC 32∶0, and, especially, with the ratio of PC 32∶0/PC 34∶1. *AGPAT1* is directly connected to phospholipid metabolism (Figures S4 and S5), as the product of this gene converts lysophosphatidic acid (LPA) into phosphatidic acid (PA) [27]. The locus lies 400 kb distant from the *HLA-DRB1* gene which was previously associated with insulin secretion [28]. A suggestive association between rs1061808 and increased T2DM risk was observed in the DIAGRAM consortium meta-analysis results.

We found two loci that strongly influence plasma LPC levels: *PKD2L1* and *PDXDC1*. An intronic variant, rs603424 in the *PKD2L1* gene, was strongly associated with LPC 16∶1. Pathway analyses suggest that another gene in the same region, *SCD* (*FADS-5*), 25 kb away, may be a better candidate since it encodes the stearoyl-CoA desaturase (delta-9-desaturase) enzyme which is involved in fatty acid desaturation. Other members of the *FADS* family are the strongest genetic regulators of phospholipid metabolism identified to date. In the *PDXDC1* locus, the strongest association was observed for intronic SNP rs4500751. This variant is 300 kb distant from *PLA2G10*, a gene that plays a major role in releasing arachidonic acid from cell membrane phospholipids [29] and the protein can be mapped to both the glycerophospholipid and the sphingolipid metabolism pathways by Ingenuity (Figures S4 and S5). In our study, the variant was strongly associated with the ratios of 20∶3 fatty acid carrying LPCs, as well as PEs, and PCs, but not with the others, suggesting a fatty-acid specific mechanism for this enzyme.

Another index SNP (rs7259004), associated with SPMs, maps to the well known *APOE* locus, which also includes three other lipid genes (*APOC1*, *APOC2* and *APOC4*). Results from the conditional analyses (Table S4) suggest that the effect of this variant on SPM 22∶0 levels is dependent on plasma LDL-C levels and that SPM 22∶0 and SPM 24∶0 are likely be abundant in LDL-C particles, which can also be inferred from their high phenotypic correlations with LDL-C (r = 0.6, *P*-value = 2.8×10^−68^ for SPM 22∶0 and r = 0.6, *P*-value = 2.8×10^−66^ for SPM 24∶0).

A second locus associated with the SPMs is *PLD2 (phospholipase D2)*. PLD2 catalyzes the hydrolysis of PC to produce phosphatidic acid and choline and the PLD2 signalling pathway is involved in the destabilization of ABCA1 and, therefore, plays a role in the generation of plasma HDL-C particles [30]. PLD2-related processes may be responsible, in part, for determining the SPM content of HDL-C. Unexpectedly, we did not observe an association between PC levels and the *PLD2* locus.

The analysis of the ratios of the phospholipids uncovered three additional associations significant at the adjusted genome-wide threshold (*P*-value<2.2×10^−9^): *ABDH3*, *PNLIPRP2* and *PCDH20*. The exact function of the ABDH3 and PCDH20 proteins, and how they relate to phospholipid metabolism, has not been determined. PNLIPRP2 (pancreatic lipase-related protein 2) fulfils a key function in dietary fat absorption by hydrolyzing triglycerides into diglycerides and, subsequently, into monoglycerides and free fatty acids (Figure S4) [31]. We found that a synonymous coding SNP (rs10885997) in *PNLIPRP2* was associated with the ratios PC 36∶1/PC 34∶1 and PC 36∶1/PC 34∶3, suggesting a fatty-acid specific turnover between these lipids.

A closer examination of the findings published by Illig et al., supports the association signals within 100 kb of loci *PDXDC1* (same SNP, *P*-value = 2.8×10^−7^), *AGPAT1* (*P*-value = 4.9×10^−7^), *PNLIPRP2* (*P*-value = 2.7×10^−7^), *KLF12* (*P*-value = 5.9×10^−7^), *ALG1* (*P*-value = 4.7×10^−3^), *CDH8* (*P*-value = 7.6×10^−7^), *PLD2* (*P*-value = 9.4×10^−4^) and *ZNF600* (*P*-value = 3.3×10^−7^) for various phospho- and sphingolipid outcomes. SNP rs603424 in *PKD2L1* was previously associated with acylcarnitine C 16∶1, although this result was not replicated [19].

The significant hits from the current study were further studied for potential associations with IMT, T2DM, and CAD. For all three outcomes, the *P*-value distributions differed significantly from the expected null distribution even after exclusion of nominally significant SNPs, suggesting that some of these variants contribute to these outcomes even when they do not achieve statistical significance.

Among our top hits, rs102275 from the *FADS* cluster was associated with IMT in the CHARGE meta-analysis results [23]. This finding demonstrates the involvement of the *FADS* locus in the development of atherosclerosis.

In addition, the top SNP from the *APOA1-5* locus was implicated in CAD risk in the CARDIoGRAM study [25]. This locus, previously associated with TG levels [20], influenced two ether-bound PCs and the PC/SPM ratio in our study. APOA1 and APOA2 are the predominant proteins in HDL-C particles, which also transport TG. The association between the phospholipids and rs964184 remained significant after adjustment for TG levels, suggesting that this signal is not due solely to TG mediated effects. APOA1 is also a cofactor for lecithin cholesterol acyltransferase (LCAT) which converts cholesterol and PC to cholesteryl esters and LPC on the surface of HDL-C [32] and it is possible that the association we observe here is due to LCAT mediated phospholipid cleavage.

Mapping the findings into the glycerophospholipid and sphingolipid metabolism pathways uncovered several enzymes, kinases, peptidases and G-protein coupled receptors that may also be relevant for phospho- and sphingolipid metabolism. Among those involved in sphingolipid metabolism (Figure S5), *HNF4A* (*hepatocyte nuclear factor-4*) appears to be a common interacting factor for several genes *(PCDH20*, *APOC1*, *AGPAT1*, *ITGA9*, *PLD2*, *C11ORF10*, *APOC2*, *GCKR*, *APOE*, *APOC3* and *LIPC)* from our GWAS. It is already known that the extinction of many hepatic functions and their expression are correlated with expression of *HNF4A* which is a candidate transcription factor for further research on lipidomics [33].

In conclusion, we identified 15 previously undescribed loci that were suggestively associated (2.2×10^−9^<*P*-value<5×10^−8^) with phospho- and sphingolipid levels. These included interesting candidate genes such as *LPAR2*. These loci will require follow-up to definitively establish their relationship with these phenotypes. We also identified nine novel loci below the corrected genome-wide significance threshold (*P-value*<2.2×10^−9^). These loci considerably expand our knowledge of genes/regions involved in the determination of phospho- and sphingolipid concentrations and provide interesting avenues for future research into this important topic.

## Materials and Methods

All studies were approved by the local ethical committees. Detailed descriptions of the study populations that contributed to the meta-analysis, as well as detailed information on ethical statements, genotyping, lipid measurements and pathway analysis, are presented in Text S1. Briefly, lipid species were quantified by electrospray ionization tandem mass spectrometry (ESIMS/MS) using methods validated and described previously [34], [35]. For each lipid molecule, we adopted the naming system where lipid side chain composition is abbreviated as C*x∶y*, where *x* denotes the number of carbons in the side chain and y the number of double bonds. For example, PC 34∶4 denotes an acyl-acyl phosphatidylcholine with 34 carbons in the two fatty acid side chains and 4 double bonds in one of them. Lipid traits were analysed individually as well as aggregated into groups of species with similar characteristics (e.g. unsaturated ceramides). These were then analyzed as both absolute concentrations (µM) and as molar percentages within lipid sub-classes (mol%) (calculated as the proportion of each lipid molecule among its own class (e.g. PC, PE, PLPE, LPC). The additive value of the analyses of molar proportions is that it may bring to light genes involved in the transition of one species to another, such as through fatty acid chain elongation or (de)saturation. We also performed single SNP association analyses for each novel locus and the ratio of the index lipid (for example, PC 34∶1) to the other lipids in the same class (in the example, PC 34∶1/PC 36∶1, PC 34∶1/PC 38∶1) so that we could determine whether the SNP might be involved in elongation or (de)saturation.

DNA samples were genotyped according to the manufacturer's instructions on Illumina Infinium HumanHap300v2, HumanHap300v1 or HumanCNV370v1 SNP bead microarrays. Genotype data for these five populations were imputed using MACH 1.0 (v1.0.16) [36], [37] using the HapMap CEU population (release 22, build 36).

As all of the studies included related individuals, testing for association between lipid and allele dosage were performed using a mixed model approach as implemented with the ‘mmscore’ option in the GenABEL software [38]. Results from the five populations were combined using inverse variance weighted fixed-effects model meta-analyses using the METAL software [39]. To correct for multiple testing, we adopted a Bonferroni correction for the number of phenotypes studied. Since most of the lipid values are correlated with each other, we used the number of principal components (n = 23) that accounted for 79% of the phenotypic variance for this correction and applied it to the classical genome-wide significance threshold (5×10^−8^).

## Supporting Information

Figure S1 Q-Q Plots from the GWAS of phospho- and sphingolipid traits with genome-wide significant findings. The x-axis shows the expected chi-square value, the y-axis shows the observed. Lambda (λ): Genomic control inflation factor.
(PDF)

Figure S2 Regional plots of phospholipid related loci. Regional association plots covering a 1 Mb window around the top SNPs were created using Locus Zoom (https://statgen.sph.umich.edu/locuszoom). Diamonds denote the index SNPs (with the smallest *P*-value). The color scale on the right refers to the linkage disequilibrium (R^2^) between each SNP and the index SNP. Blue peaks show recombination rates.
(PDF)

Figure S3 Regional plots of sphingolipid related loci. Regional association plots covering a 1 Mb window around the top SNPs were created using Locus Zoom (https://statgen.sph.umich.edu/locuszoom). Diamonds denote the index SNPs (with the smallest *P*-value). The color scale on the right refers to the linkage disequilibrium (R^2^) between each SNP and the index SNP. Blue peaks show recombination rates.
(PDF)

Figure S4 Lipid Pathways by Ingenuity (glycerophospholipid metabolism). Genes discovered in the GWAS are shown in orange. 1.Phosphatidylcholine-sterol O-acyltransferase has member mouse Lcat. 2. ApoA-I [APOA1] increases activation of LCAT. Binding of apoA-I [APOA1] and LCAT occurs. 3. Association of human APOA1 protein and human APOL1 protein occurs. 4. The affinity of binding of human APOL1 protein and cardiolipin in a system of purified components is greater than the affinity of binding of human APOL1 protein and phosphatidylinositol 3,5-bisphosphate in a system of purified components. 5. Binding of cardiolipin and human Matrilysin [MMP7] protein occurs in a cell-free system. 6. MMP7 protein increases C-terminal truncation cleavage of APOA1 protein. 7. In cell surface from RAW 264.7 cells, 8-bromo-cAMP increases binding of APOA1 protein and phosphatidylserine. 8. Phospholipase A1 catalyzes the following reaction: 1 phosphatidylserine+1 water−>1 2-Acyl-sn-glycero-3-phosphoserine+1 fatty acid. 9. Phosphatidylserine decarboxylase catalyzes the following reaction: 1 carbon dioxide+1 phosphatidylethanolamine−>1 phosphatidylserine. 10. Binding of phosphatidylserine and PKC ALPHA [PRKCA] protein occurs in a system of purified components. 11. Binding of human APOA1 protein and human GPI-PLD [GPLD1] protein occurs in human plasma. 12. Binding of apoA-I [APOA1] and phosphatidylcholine occurs. A molecular complex consisting of APOAI [APOA1] and of phosphatidylcholine increases secretion of phosphatidylcholine. 13. Lysophospholipase catalyzes the following reaction: 2 fatty acid+1 sn-glycero-3-phosphocholine−>1 phosphatidylcholine+2 water. 14.Human SPLA2 [PLA2G10] protein increases release of phosphatidylcholine to arachidonic acid. Mammalian group I sPLA2 [PLA2G10] increases hydrolysis of phosphatidylcholine. 15. In pulmonary surfactant, mouse sPLA2-X [Pla2g10] protein increases hydrolysis of phosphatidylglycerol. 16. Phospholipase A2 has member rat Lcat. 17. Rat Pla2 has member rat Pla2g10. 18. Human PLA2 has member human PLA2R1. 19. Phospholipase A2 has member human YWHAZ. 20. Binding of human PCTK2 [CDK17] protein and human YWHAZ protein occurs. 21. Phospholipase A2 catalyzes the following reaction: 1 1-acyl-sn-glycero-3-phosphocholine+1 water−>1 monocarboxylic acid+1 sn-glycero-3-phosphocholine. 22. Phospholipase A2 catalyzes the following reaction: 1 1-(1-alkenyl)-sn-glycero-3-phosphocholine−>1 choline plasmalogens. 23. Phospholipase A2 catalyzes the following reaction: 1 phosphatidylethanolamine+1 water−>1 1-acyl-sn-glycero-3-phosphoethanolamine+1 fatty acid. 24. Phospholipase A2 has member rat Lamb2. 25. Binding of human LAMB2 protein and human ZNF512B protein occurs. 26. Binding of human PLCG2 protein and human ZNF512B protein occurs. 27. Interaction of cytochrome b5 [CYB5] and phosphatidylcholine bilayers occurs. 28. Binding of rat Cytb5 [Cyb5a] protein and rat Delta 6 desaturase [Fads2] protein occurs in Cos-7 cells. 29. In CHO-ldlA7 cells, binding of a protein-protein complex consisting of mutant human APOA1 (R160V;H162A with its amphipathic helix 6 mutated) and of cholesterol and of phosphatidylcholine and mouse Sr-bi [Scarb1] protein is the same as binding of a protein-protein complex consisting of mutant human APOA1 (R160V;H162A with its amphipathic helix 6 mutated) and of cholesterol and of phosphatidylcholine and mutant mouse Sr-bi [Scarb1] protein (M158R). 30. Interaction of C-reactive [CRP] and artificial phosphatidylcholine bilayers occurs. 31. Binding of 10 kd mutant human APOE protein (N-terminal truncation 1–222 with its carboxy terminal domain retained) and phosphatidylcholine and triolein occurs in a system of purified components. 32. Binding of phosphatidylcholine and PRNP protein occurs in detergent-resistant membrane fraction from FRT cells. 33. Brefeldin A decreases association of phosphatidylcholine and a protein fragment containing a N-terminal domain from human Apob protein. 34. Binding of phosphatidylcholine and Phospholipase c [Plc] protein(s) occurs. Phospholipase C catalyzes the following reaction: 1 phosphatidylcholine+1 water−>1 1,2-diacylglycerol+1 phosphorylcholine. 35. In NCI-H295r cells, binding of human APOE3 protein and human SR-BI [SCARB1] protein increases uptake of cholesteryl ester. 36. Interaction of apolipoprotein E [APOE] and rat CRP occurs. 37. Association of apoE and phospholipid occurs. 38. Association of apoE and phospholipid occurs. 39. Binding of phospholipid and Phospholipase c [Plc] protein(s) occurs. 40. Human PLC has member human PLCG2. 41. Human EDG4 [LPAR2] is involved in activation of phospholipase C. 42. Plc has member PLCB1. 43. Binding of human APOB protein and human LIPC protein occurs. 44. Triacylglycerol lipase has member mouse Lipc. 45. Triacylglycerol lipase has member human DAGLA. 46. Triacylglycerol lipase has member rat Pnliprp2. 47. Triacylglycerol lipase catalyzes the following reaction: 1 fatty acid+1 sn-2-monoacylglycerol−>1 1,2-diacylglycerol+1 water. 48. Carnitine O-palmitoyltransferase catalyzes the following reaction: 3 coenzyme A+1 triglyacylglycerol−>3 acyl-coenzyme A+1 glycerol. 49. Phospholipid:diacylglycerol acyltransferase catalyzes the following reaction: 1 lysophospholipids+1 triglyacylglycerol−>1 1,2-diacylglycerol+1 phospholipid. 50. Sphingosine N-acyltransferase catalyzes the following reaction: 1 acyl-coenzyme A+1 sphingosylphosphocholine−>1 coenzyme A+1 sphingomyelin. 51. Sphingosine N-acyltransferase has member rat Lass4. 52. Non-amino-acyl group acyltransferase catalyzes the following reaction: 1 2-acyl-sn-glycerol 3-phosphate+1 coenzyme A−>1 acyl-coenzyme A+1 glycerophosphoric acid. 53. Non-amino-acyl group acyltransferase has member rat Elovl2. 54. Association of apoC-III [APOC3] and triglyacylglycerol occurs. 55. Lipoprotein lipase has member human APOC2. 56. 1-acylglycerol-3-phosphate O-acyltransferase has member rat Agpat1. 57. 1-acylglycerol-3-phosphate O-acyltransferase catalyzes the following reaction: 1 coenzyme A+1 phosphatidic acid−>1 1-Acyl-sn-glycerol 3-phosphate+1 2,3-dehydroacyl-coenzyme A. 58. Binding of purified rat Plcb1 protein and a protein fragment containing a phox homology domain from human PLD2 protein occurs in a cell free system. 59. EGF protein increases dissociation of human PLD2 protein and mouse Munc-18a [Stxbp1] gene. Binding of a protein fragment containing a PX domain from human PLD2 protein and mouse Munc-18a [Stxbp1] protein occurs in a system of purified components. 60. Binding of rat Pld protein(s) and rat Munc-18 [Stxbp1] protein occurs in rat brain. 61. GRB2 protein increases activation of rat Pld protein(s) that is increased by Pdgf complex(es). Association of growth factor receptor-bound protein 2 [GRB2] and PLD occurs. 62. Binding of human GRB2 protein and human KCNH7 protein occurs. 63. Mouse Pld2 protein increases activation of human Pld protein(s) in HPAEC cells that is increased by hyperoxia of HPAEC cells. Phospholipase D has member mouse Pld2. 64. In cytoplasm, PLD protein(s) catalyzes the following reaction: phosphatidylcholine−>phosphatidic acid. 65. Phospholipase D has member rat Gpld1. 66. Binding of transgenic human APOA-I [APOA1] protein and cholesteryl ester and mouse HDL in plasma from blood of mutant mouse with a homozygous knockout of mouse Apoa1 occurs. 67. Binding of ARF1 protein and phosphatidic acid occurs in a cell fraction from Cos cells. 68. Binding of ARF1 protein and a protein fragment containing a C2A domain from rat Syt9 protein occurs in lysate from RBL-2H3 cells. 69. Binding of phosphatidic acid and PKC ALPHA [PRKCA] protein occurs in a system of purified components. 70. In cytoplasm, DGK ζ [DGKZ] protein catalyzes the following reaction: DAG−>PA. 71. Association of rat Dgkz protein and rat Dlg1 protein and rat Dlg2 protein and rat Dlg3 protein and rat Dlg4 protein occurs. 72. Binding of human PLD2 protein and human PRKCA protein occurs. 73. Mouse Pla2g1br [Pla2r1] protein decreases binding of mouse Pla2g10 protein and PLA2R [PLA2R1] protein.
(PDF)

Figure S5 Lipid Pathways by Ingenuity (sphingolipid metabolism). Genes discovered in the GWAS are shown in orange. 1. In mouse liver, mouse PPAR-alpha [Ppara] protein is necessary for expression of mouse Delta 6 desaturase [Fads2] mRNA that is mediated by WY-14643. Binding of a DNA fragment (−385–373) containing a DR1 from human DELTA 6 DESATURASE [FADS2] gene and a protein-protein complex consisting of mouse PPAR-alpha [Ppara] and of rat Rxr alpha [Rxra] occurs in a cell free system. 2. In Hep3B cells expressing human APOAV [APOA5] protein, human Ppar alpha [PPARA] protein increases activation of promoter fragment (−617–18) from human APOAV [APOA5] gene. 3. In mouse liver, mouse PPAR-alpha [Ppara] protein is necessary for expression of mouse Fads1 mRNA that is mediated by WY-14643. 4. Binding of palmitoyl-CoA and mutant mouse Ppar alpha [Ppara] protein (N-terminal truncation 1–100 with its A/B domain deleted) occurs in a cell-free system. 5. PPARA protein decreases expression of APOC3 protein. Binding of promoter fragment (−96–61) from human APOC3 gene consisting of hormone response element and a protein-protein complex consisting of PPAR ALPHA [PPARA] and of RXR ALPHA [RXRA] occurs in a cell fraction from Cos-1 cells. PPAR ALPHA [PPARA] protein decreases transcription of APOC [APOC3] gene with a DNA endogenous promoter that has a PPAR response element. 6. PPAR ALPHA [PPARA] protein increases expression of human APOA1. 7. In HepG2 cells, mutant mouse HNF1-alpha [Hnf1a] protein (R131Q) causes little or no change in activation of APOC3 gene that is increased by human HNF4A2 protein. In HuH7 cells, rat Hnf4 alpha2 protein increases expression of human APOC3 mRNA. In a nuclear extract from Caco2 cells, the binding avidity of binding of promoter fragment from APOC3 gene consisting of HNF4 binding site and human HNF4A2 protein is greater than the binding avidity of binding of promoter fragment from FABP1 gene consisting of HNF4 binding site and human HNF4A2 protein. HNF4 protein increases trans-activation of DNA endogenous promoter from APOC [APOC3] gene that is decreased by C JUN [JUN] protein. Binding of rat Hnf4a protein and palmitoyl-CoA occurs in a cell-free system. 9. Binding of human HNF4A protein and human PCDH20 gene occurs. 10. In HuH7 cells, rat Hnf4 alpha2 protein increases expression of human APOC1 mRNA. 11. Binding of human AGPAT1 gene and human HNF4A protein occurs. 12. Binding of human HNF4A protein and human ITGA9 gene occurs. 13. Binding of human HNF4A protein and human PLD2 gene occurs. 14. In HuH7 cells, ARP-1 [NR2F2] protein decreases activation of promoter fragment (−851–29) containing a AP-1 site and a DR1 response element and a DR4 and a Hnf1 binding site and a USF binding site from human LIPC gene that is increased by HNF4A protein. In HuH7 cells, PGC1A [PPARGC1] protein increases trans-activation of promoter fragment (−851–29) containing a AP-1 site and a DR1 response element and a DR4 and a Hnf1 binding site and a USF binding site from human LIPC gene that is dependent on HNF4A protein. 15 Binding of human C11orf10 gene and human HNF4A protein occurs. 16. Binding of human GCKR gene and human HNF4A protein occurs. 17. In F9 cells, HNF4A protein and mouse Pgc-1alpha [Ppargc1a] protein increase expression of mouse Apoc2 mRNA. Binding of human APOC2 gene and human HNF4A protein occurs. 18. In HuH7 cells, rat Hnf4 alpha2 protein increases expression of human APOE mRNA, 19. In HuH7 cells, rat Hnf4 alpha2 protein increases expression of human APOA1 mRNA. Binding of a DNA fragment (−205–192) containing a ApoA1 site A from human Apoa1 gene and Hnf4 protein occurs in a nuclear extract from Cos cells. 20. CDPdiacylglycerol-serine O-phosphatidyltransferase catalyzes the following reaction: 1 cytidine 3′-phosphate+1 phosphatidylserine−>1 CDPdiacylglycerol+1 L-serine. 21. Substituted phosphate group transferase catalyzes the following reaction: 1 choline+1 phosphatidylserine−>1 L-serine+1 phosphatidylcholine. 22. Binding of CRP protein and PE occurs in a cell-free system. 23. Interaction of apolipoprotein E [APOE] and rat CRP occurs. 24. In cell surface from RAW 264.7 cells, 8-bromo-cAMP increases binding of APOA1 protein and phosphatidylserine. 25. Binding of fibrillar AMYLOID BETA protein(s) and human APOA1 protein occurs in human serum. 26. Binding of APOA1 protein and cholesterol occurs in extracellular space. 27. Association of human APOA1 protein and human APOL1 protein occurs. 28. Binding of V. cholerae Cholera toxin B [CtxB] protein and a molecular complex consisting of DMPC and of human APOA-I [APOA1] and of GM1 in a cell-free system occurs. 29. Beta-galactosidase catalyzes the following reaction: 1 galactose+1 ganglioside GM2−>1 ganglioside GM1+1 water. 30. Hexosyltransferase catalyzes the following reaction: 1 ganglioside GM1+1 GDP fucose−>1 fucosyl-GM1+1 GDP. 31. Exo-alpha-sialidase catalyzes the following reaction: 1 ganglioside GD1a+1 water−>1 ganglioside GM1+1 N-acetylneuraminic acid. 32. The affinity of binding of human APOL1 protein and 3-sulfogalactosylceramide in a system of purified components is greater than the affinity of binding of human APOL1 protein and phosphoinositol in a system of purified components. 33. Interaction of cholesterol and galactosylceramide occurs. 34. In hepatocytes from fasted female mouse, mutant mouse SR-BI [Scarb1] gene (homozygous knockout) and hypomorphic mouse SR-BI [Scarb1] gene increase accumulation of a molecular complex consisting of mouse Apoe and of free cholesterol in plasma from fasted female mouse that involves atherogenic diet. 35. Interaction of Cholesterol and sphingomyelin occurs. 36. Binding of human AMYLOID BETA 40 protein and galactosylceramide occurs in a cell-free system. 37. Binding of A BETA 42 [AMYLOID-BETA PEPTIDE 42] protein and sphingomyelin in phosphatidylcholine vesicles occurs. 38. Binding of mouse Apoe protein and mouse Prnp protein occurs. 39. Binding of PRNP protein and sphingomyelin occurs in detergent-resistant membrane fraction from FRT cells. 40. Sphingosine N-acyltransferase catalyzes the following reaction: 1 acyl-coenzyme A+1 sphingosylphosphocholine−>1 coenzyme A+1 sphingomyelin. 41. Sphingosine N-acyltransferase catalyzes the following reaction: 1 coenzyme A+1 phytoceramide−>1 hexacosanoyl-coenzyme A+1 phytosphingosine. 42. Sphingosine N-acyltransferase catalyzes the following reaction: 1 acyl-coenzyme A+1 D-erythro-dihydrosphingosine−>1 coenzyme A+1 dihydroceramide. 43. Sphingosine N-acyltransferase catalyzes the following reaction: 1 acyl-coenzyme A+1 D-sphingosine−>1 ceramide+1 coenzyme A. 44. Sphingosine N-acyltransferase has member rat Lass4. 45. Human Gpcr has member human LPAR2. 46.Binding of G protein coupled receptor [Gpcr] protein(s) and sphingosine-1-phosphate occurs. 47. Sphingosine-1-phosphate phosphatase protein(s) catalyzes the following reaction: 1 sphingosine-1-phosphate−>1 Sphingosine. 48. Sphingosine-1-phosphate phosphatase protein(s) catalyzes the following reaction: 1 ceramide-1-phosphate−>1 ceramide. 49. Binding of human S1PR5 protein and human SGPP1 protein occurs. 50. Dihydrosphingosine 1-phosphate increases activation of EDG8 protein. Binding of dihydrosphingosine 1-phosphate and EDG8 protein occurs. 51. S-1P in extracellular space increases activation of S1PR5 [EDG8] protein in plasma membrane. Binding of EDG8 protein and sphingosine-1-phosphate occurs in Cho cells. 52. Calcium and lipids are necessary for binding of ceramide-1-phosphate and a protein fragment containing a C2 domain from Cpla2 protein(s). 53.Human Cpla2 has member human PLA2G10. 54. Rat Pla2 has member rat Pla2g10. 55. Binding of ceramide and Phospholipase A2 [Pla2] protein(s) increases apoptosis of cells. 56. Ceramide increases activation of protein kinase C zeta [PRKCZ]. Binding of CDC42 protein in a cell-free system and ceramide in phosphatidylserine vesicles and PAR6 [PARD6A] protein in a cell-free system and human PKCZ [PRKCZ] protein in a cell-free system is greater than binding of CDC42 protein in a cell-free system and ceramide in phosphatidylcholine vesicles and PAR6 [PARD6A] protein in a cell-free system and human PKCZ [PRKCZ] protein in a cell-free system. 57. Ceramide is involved in activation of PKCA [PRKCA] protein in cells. Binding of ceramide and protein kinase C-alpha [PRKCA] occurs. 58. In a in vitro system, phox homology domain from human PLD2 protein increases activation of human PKC ZETA [PRKCZ] protein. Binding of PLD2 protein containing a CT motif and a CRIV and a CR1 domain and a CR2 domainand a CR3 domain and a phox homology domain and a pleckstrin homology (PH) domain and PKC ZETA [PRKCZ] protein containing a C1 domain and a PB1 domain and a serine/threonine kinase domain occurs in Cos-7 cells. 59. Binding of human PLD2 protein and human PRKCA protein occurs. 60. In Cos-7 cells, human PLD2 protein increases activation of c-src [SRC] protein. Human SRC protein increases phosphorylation of PLD2 protein in a cell-free system. 61. Binding of a protein fragment containing a pleckstrin homology (PH) domain from PLD2 protein and c-src [SRC] protein occurs in lysate from Cos-7 cells. In a cell-free system, SRC protein increases phosphorylation of phosphatase. 62. Pp60c-src increases phosphorylation of L-serine. 63. Human EGFR protein increases phosphorylation of PLD2 protein in a cell-free system. Binding of human EGFR protein and human PLD2 protein occurs. 64.Binding of extracellular domain from human EGFR protein and Ganglioside GM4 occurs in a system of purified components. 65. GSK3beta increases phosphorylation of serine [L-serine]. 66. In LNCaP cells, dominant negative mutant ILK protein (R211A) causes little or no change in phosphorylation of GSK-3beta [GSK3B] protein to phosphorylated (S9) GSK-3beta [GSK3B] protein that is decreased by ILKAP protein.
(PDF)

Figure S6 Q-Q plots for the association between the top loci and disease end points. *P*_KS_: *P*-value from a one sample Kolmogorov-Smirnov test comparing the observed *P*-value distribution to that expected under the null.
(PDF)

Table S1 Mean phospho- and sphingolipid concentrations of the study participants. SD: standard deviation; *P*-value: P-value for the test comparing means by gender.
(PDF)

Table S2 Association findings on selected lipid-lipid ratios. Effect: regression coefficient; seEffect: standard error of the regression coefficient.
(PDF)

Table S3 GWAS of standard plasma lipid measures in the EUROSPAN Consortium. Allele1: Effect allele; Effect: regression coefficient; StdErr: standard error of the regression coefficient.
(PDF)

Table S4 Conditional analysis of the genome-wide significant loci. Effect: regression coefficient; StdErr: standard error of the regression coefficient.
(PDF)

Table S5 ConsensusPathDB pathway enrichment for phospholipid related loci. Gene list: ALG14, GCKR, KCNH7, ILKAP, ITGA9, PAQR9, ELOVL2, AGPAT1, PKD2L1, PNLIPRP2, SYT9, OR8I2, FADS1, DLG2, APOA1, CDK17, PCDH20, KLF12, PLEKHH1, LIPC, ALG1, PDXDC1, CDH8, ABHD3, ZNF600. +: Pathways with *P*-value<0.01; *: *P*-value after correction for False Discovery Rate; KEGG: http://www.genome.jp/kegg/; Reactome: http://www.reactome.org; SMPDB: http://www.smpdb.ca/; HumanCyc: http://humancyc.org/; PharmGKB: http://www.pharmgkb.org/; Wikipathways: http://www.wikipathways.org; EHMN: http://www.ehmn.bioinformatics.ed.ac.uk.
(PDF)

Table S6 ConsensusPathDB pathway enrichment for sphingolipid related loci. Gene list: ATP10D, SRD5A1, FADS2, SGPP1, CNTNAP4, PLD2, LPAR2, LASS4, APOE, SPTLC3. +: Pathways with *P*-value<0.01; *: *P*-value after correction for False Discovery Rate; Reactome: http://www.reactome.org; Wikipathways: http://www.wikipathways.org; PID: http://pid.nci.nih.gov/.
(PDF)

Table S7 Genome wide significant SNPs and their associations with IMT (CHARGE Consortium, Bis et al, Nat Genet 43:940–947, 2011). Effect: regression coefficient; seEffect: standard error of the regression coefficient.
(PDF)

Table S8 Genome wide significant SNPs and their associations with type 2 diabetes risk (DIAGRAM Consortium, Voight et al, Nat Genet 42:579–589, 2010). 95% CI: 95% Confidence Interval.
(PDF)

Table S9 Genome wide significant SNPs and their associations with coronary artery disease risk (CARDIoGRAM Consortium, Schunkert et al, Nat Genet 43: 333–338, 2011). 95% CI: 95% Confidence Interval.
(PDF)

Text S1 Extensive materials and methods.
(DOC)

Text S2 Full list of phospholipids that are significantly associated to *FADS1-2-3* and *LIPC* region SNPs.
(DOC)

## References

[1] Holthuis JC, Pomorski T, Raggers RJ, Sprong H, Van Meer G (2001). The organizing potential of sphingolipids in intracellular membrane transport.. Physiol Rev.

[2] Lajoie P, Goetz JG, Dennis JW, Nabi IR (2009). Lattices, rafts, and scaffolds: domain regulation of receptor signaling at the plasma membrane.. J Cell Biol.

[3] Merrill AS, K, Vance DV, JE (2002). Sphingolipids: metabolism and cell signaling.. Biochemistry of Lipids, Lipoproteins and Membranes.

[4] Bakovic M, Fullerton MD, Michel V (2007). Metabolic and molecular aspects of ethanolamine phospholipid biosynthesis: the role of CTP:phosphoethanolamine cytidylyltransferase (Pcyt2).. Biochem Cell Biol.

[5] Poli G, Leonarduzzi G, Biasi F, Chiarpotto E (2004). Oxidative stress and cell signalling.. Curr Med Chem.

[6] van Meer G, Voelker DR, Feigenson GW (2008). Membrane lipids: where they are and how they behave.. Nat Rev Mol Cell Biol.

[7] Zheng W, Kollmeyer J, Symolon H, Momin A, Munter E (2006). Ceramides and other bioactive sphingolipid backbones in health and disease: lipidomic analysis, metabolism and roles in membrane structure, dynamics, signaling and autophagy.. Biochim Biophys Acta.

[8] Broekman MJ, Handin RI, Derksen A, Cohen P (1976). Distribution of phospholipids, fatty acids, and platelet factor 3 activity among subcellular fractions of human platelets.. Blood.

[9] Engelmann B, Kogl C, Kulschar R, Schaipp B (1996). Transfer of phosphatidylcholine, phosphatidylethanolamine and sphingomyelin from low- and high-density lipoprotein to human platelets.. Biochem J.

[10] Brugger B, Erben G, Sandhoff R, Wieland FT, Lehmann WD (1997). Quantitative analysis of biological membrane lipids at the low picomole level by nano-electrospray ionization tandem mass spectrometry.. Proc Natl Acad Sci U S A.

[11] Hodge AM, English DR, O'Dea K, Sinclair AJ, Makrides M (2007). Plasma phospholipid and dietary fatty acids as predictors of type 2 diabetes: interpreting the role of linoleic acid.. Am J Clin Nutr.

[12] Malerba G, Schaeffer L, Xumerle L, Klopp N, Trabetti E (2008). SNPs of the FADS gene cluster are associated with polyunsaturated fatty acids in a cohort of patients with cardiovascular disease.. Lipids.

[13] Mannheim D, Herrmann J, Versari D, Gossl M, Meyer FB (2008). Enhanced expression of Lp-PLA2 and lysophosphatidylcholine in symptomatic carotid atherosclerotic plaques.. Stroke.

[14] Matsumoto T, Kobayashi T, Kamata K (2007). Role of lysophosphatidylcholine (LPC) in atherosclerosis.. Curr Med Chem.

[15] Wang L, Folsom AR, Zheng ZJ, Pankow JS, Eckfeldt JH (2003). Plasma fatty acid composition and incidence of diabetes in middle-aged adults: the Atherosclerosis Risk in Communities (ARIC) Study.. Am J Clin Nutr.

[16] Farooqui AA, Horrocks LA, Farooqui T (2000). Glycerophospholipids in brain: their metabolism, incorporation into membranes, functions, and involvement in neurological disorders.. Chem Phys Lipids.

[17] Gieger C, Geistlinger L, Altmaier E, Hrabe de Angelis M, Kronenberg F (2008). Genetics meets metabolomics: a genome-wide association study of metabolite profiles in human serum.. PLoS Genet.

[18] Hicks AA, Pramstaller PP, Johansson A, Vitart V, Rudan I (2009). Genetic determinants of circulating sphingolipid concentrations in European populations.. PLoS Genet.

[19] Illig T, Gieger C, Zhai G, Romisch-Margl W, Wang-Sattler R (2009). A genome-wide perspective of genetic variation in human metabolism.. Nat Genet.

[20] Teslovich TM, Musunuru K, Smith AV, Edmondson AC, Stylianou IM (2010). Biological, clinical and population relevance of 95 loci for blood lipids.. Nature.

[21] Kamburov A, Wierling C, Lehrach H, Herwig R (2009). ConsensusPathDB–a database for integrating human functional interaction networks.. Nucleic Acids Res.

[22] Jimenez-Marin A, Collado-Romero M, Ramirez-Boo M, Arce C, Garrido JJ (2009). Biological pathway analysis by ArrayUnlock and Ingenuity Pathway Analysis.. BMC Proc.

[23] Bis JC, Kavousi M, Franceschini N, Isaacs A, Abecasis GR (2011). Meta-analysis of genome-wide association studies from the CHARGE consortium identifies common variants associated with carotid intima media thickness and plaque.. Nat Genet.

[24] Voight BF, Scott LJ, Steinthorsdottir V, Morris AP, Dina C (2010). Twelve type 2 diabetes susceptibility loci identified through large-scale association analysis.. Nat Genet.

[25] Schunkert H, Konig IR, Kathiresan S, Reilly MP, Assimes TL (2011). Large-scale association analysis identifies 13 new susceptibility loci for coronary artery disease.. Nat Genet.

[26] Tang YT, Hu T, Arterburn M, Boyle B, Bright JM (2005). PAQR proteins: a novel membrane receptor family defined by an ancient 7-transmembrane pass motif.. J Mol Evol.

[27] Aguado B, Campbell RD (1998). Characterization of a human lysophosphatidic acid acyltransferase that is encoded by a gene located in the class III region of the human major histocompatibility complex.. J Biol Chem.

[28] Howson JM, Rosinger S, Smyth DJ, Boehm BO, Todd JA (2011). Genetic Analysis of Adult-Onset Autoimmune Diabetes.. Diabetes.

[29] Singh DK, Subbaiah PV (2007). Modulation of the activity and arachidonic acid selectivity of group X secretory phospholipase A2 by sphingolipids.. J Lipid Res.

[30] Wang Y, Oram JF (2005). Unsaturated fatty acids phosphorylate and destabilize ABCA1 through a phospholipase D2 pathway.. J Biol Chem.

[31] Giller T, Buchwald P, Blum-Kaelin D, Hunziker W (1992). Two novel human pancreatic lipase related proteins, hPLRP1 and hPLRP2. Differences in colipase dependence and in lipase activity.. J Biol Chem.

[32] Breslow JL, Ross D, McPherson J, Williams H, Kurnit D (1982). Isolation and characterization of cDNA clones for human apolipoprotein A-I.. Proc Natl Acad Sci U S A.

[33] Chartier FL, Bossu JP, Laudet V, Fruchart JC, Laine B (1994). Cloning and sequencing of cDNAs encoding the human hepatocyte nuclear factor 4 indicate the presence of two isoforms in human liver.. Gene.

[34] Liebisch G, Drobnik W, Lieser B, Schmitz G (2002). High-throughput quantification of lysophosphatidylcholine by electrospray ionization tandem mass spectrometry.. Clin Chem.

[35] Liebisch G, Lieser B, Rathenberg J, Drobnik W, Schmitz G (2004). High-throughput quantification of phosphatidylcholine and sphingomyelin by electrospray ionization tandem mass spectrometry coupled with isotope correction algorithm.. Biochim Biophys Acta.

[36] Nothnagel M, Ellinghaus D, Schreiber S, Krawczak M, Franke A (2009). A comprehensive evaluation of SNP genotype imputation.. Hum Genet.

[37] Li Y, Willer CJ, Ding J, Scheet P, Abecasis GR (2010). MaCH: using sequence and genotype data to estimate haplotypes and unobserved genotypes.. Genet Epidemiol.

[38] Aulchenko YS, Ripke S, Isaacs A, van Duijn CM (2007). GenABEL: an R library for genome-wide association analysis.. Bioinformatics.

[39] Willer C, Li Y, Abecasis G (2010). METAL: fast and efficient meta-analysis of genomewide association scans.. Bioinformatics.

