# Supplementary material for: Genome-Wide Association Study Identifies Novel Loci Associated with Circulating Phospho- and Sphingolipid Concentrations
Source: PLoS Genet. 2012 Feb 16;8(2):e1002490. doi: 10.1371/journal.pgen.1002490 (PMC3280968; doi:10.1371/journal.pgen.1002490)

Figure S1

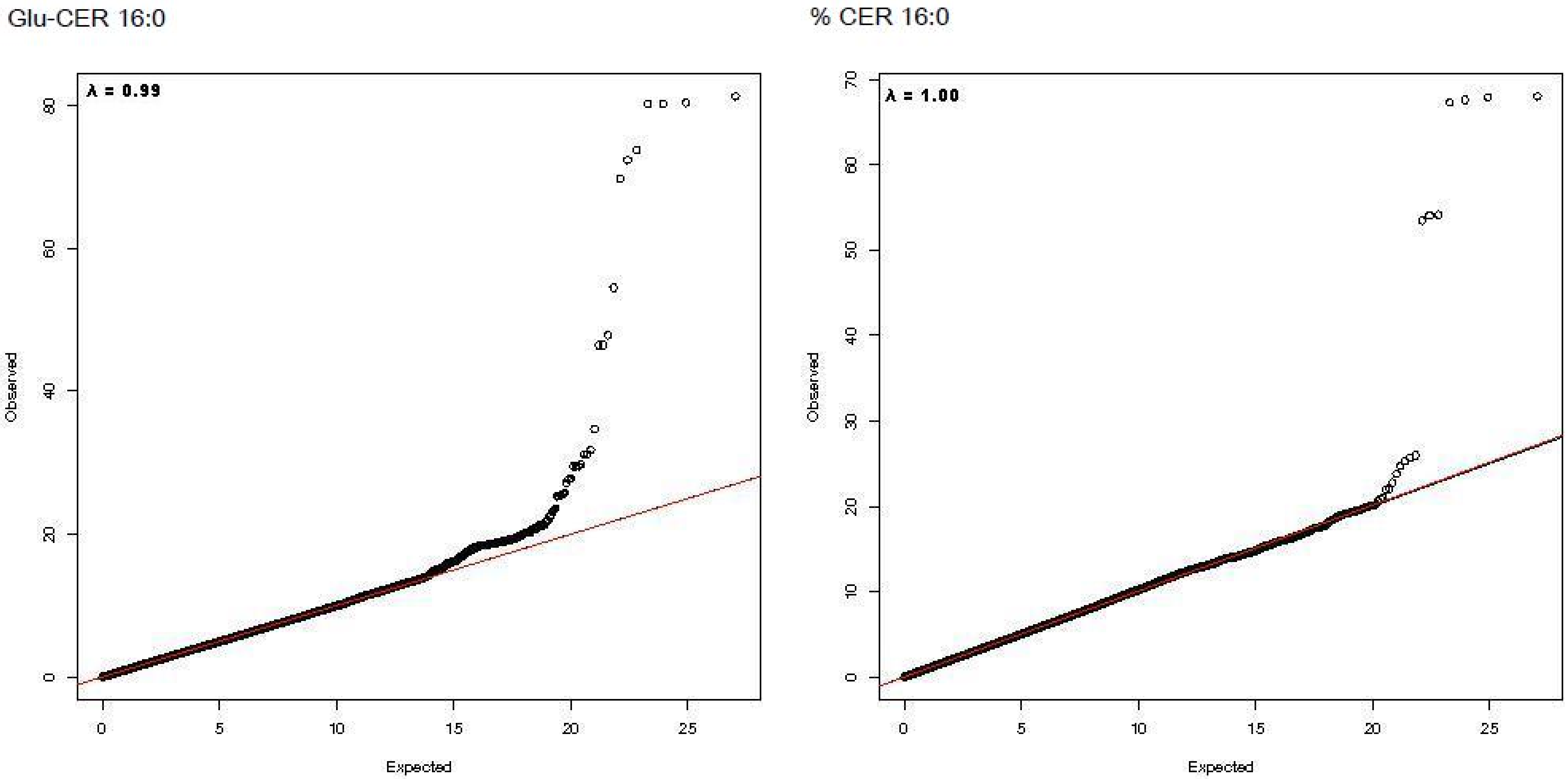
Glu-CER 16:0
λ = 0.99
Observed
Expected
0
20
40
60
80
5
10
15
25
% CER 16:0
λ = 1.00
30
50
70

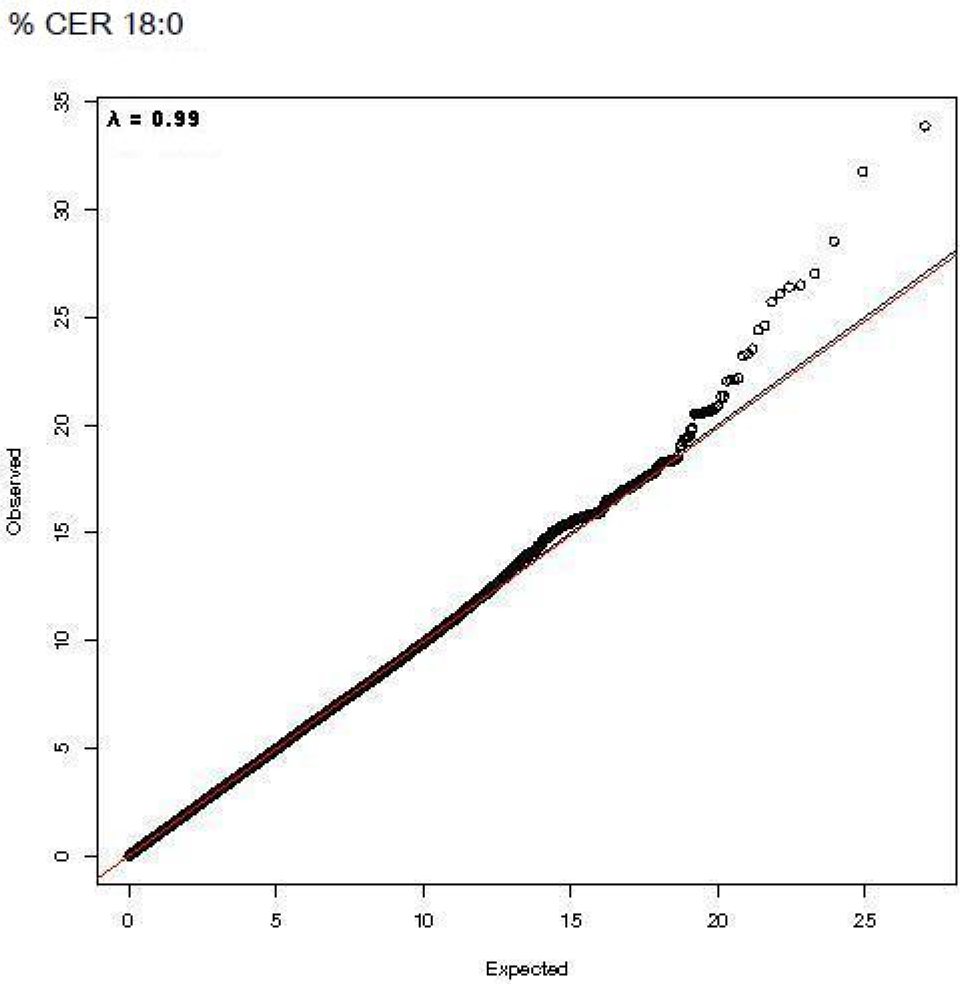
% CER 18:0
λ = 0.99
Observed
Expected
0
5
10
15
20
25
30
35

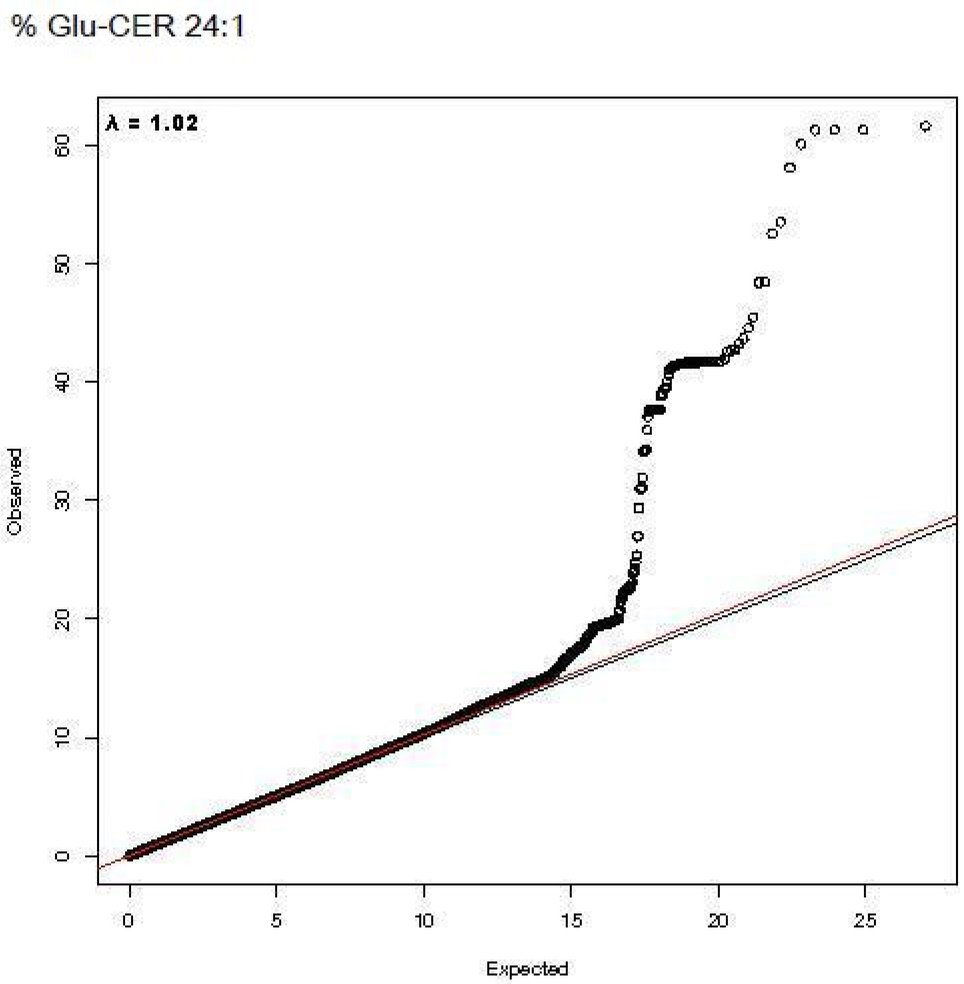
% Glu-CER 24:1
λ = 1.02
Observed
Expected
0
10
20
30
40
50
60
5
15
25

% LPC 16:1

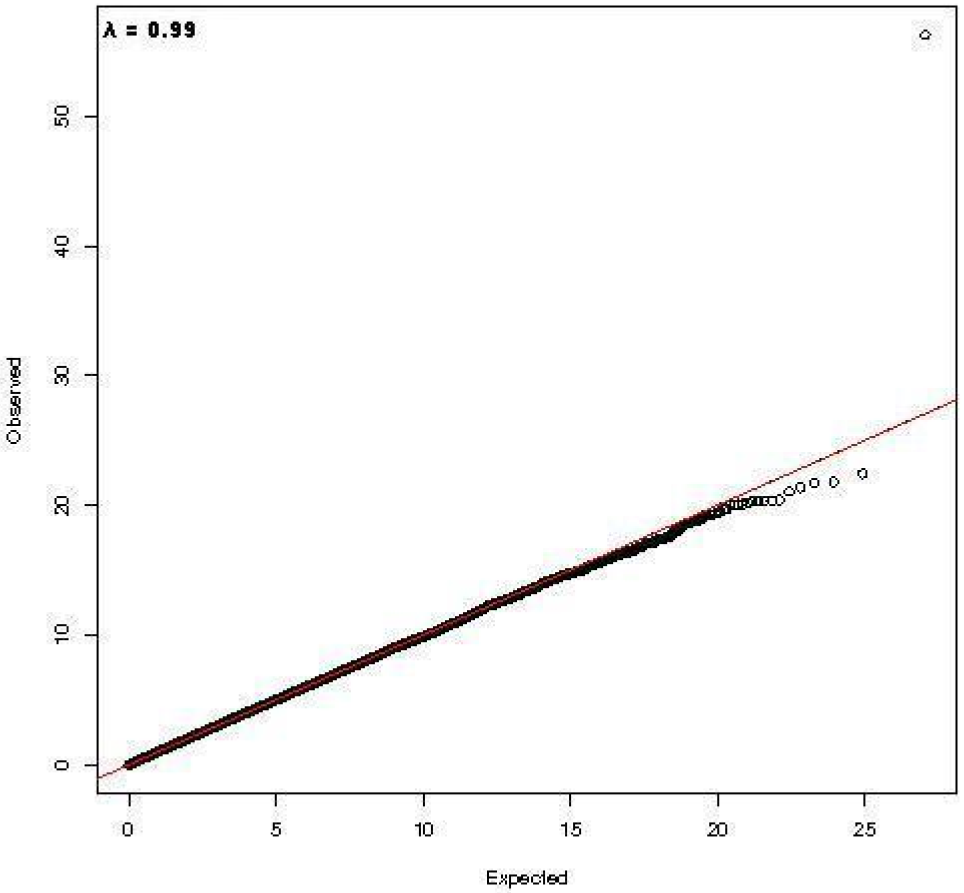

% LPC 18:0

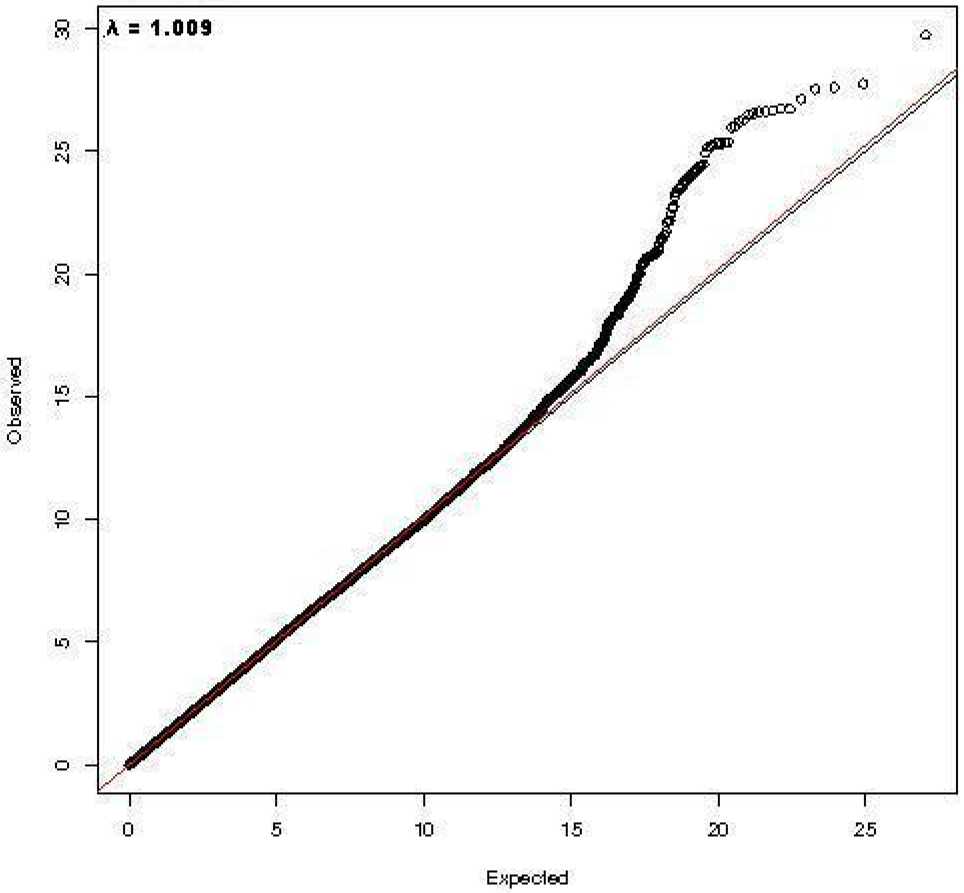

% LPC 20 :3

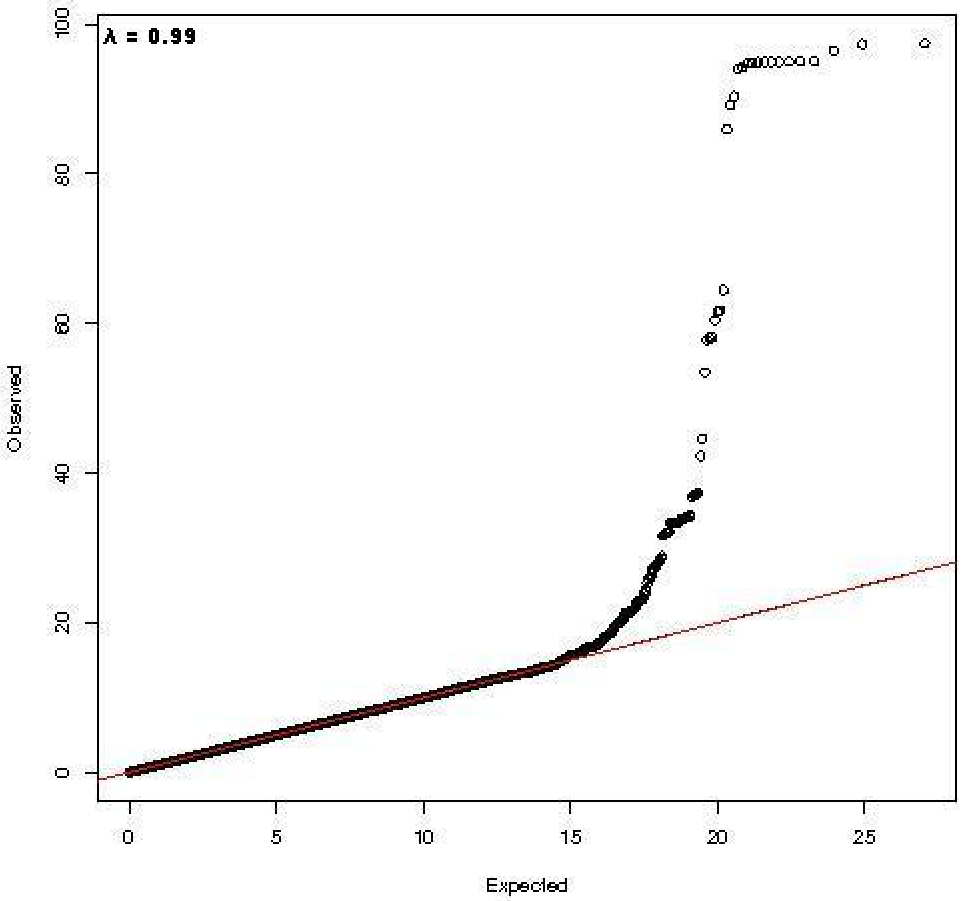

% Saturated LPC

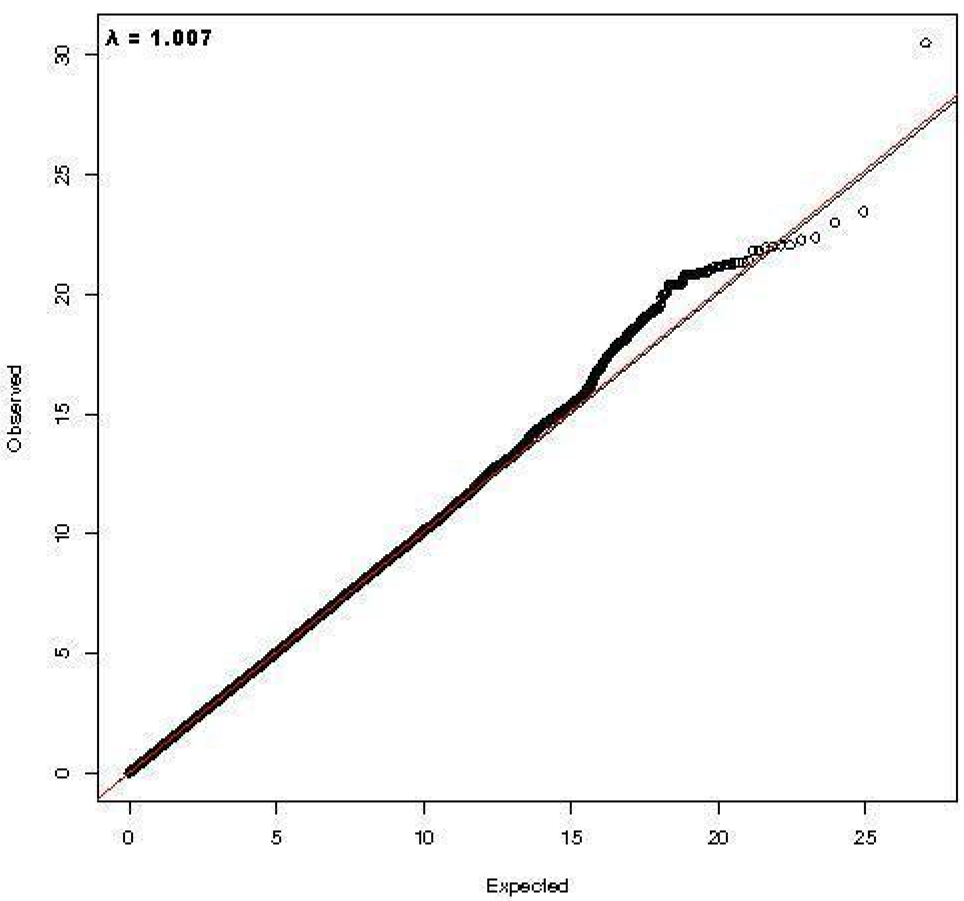

PC 30 :1

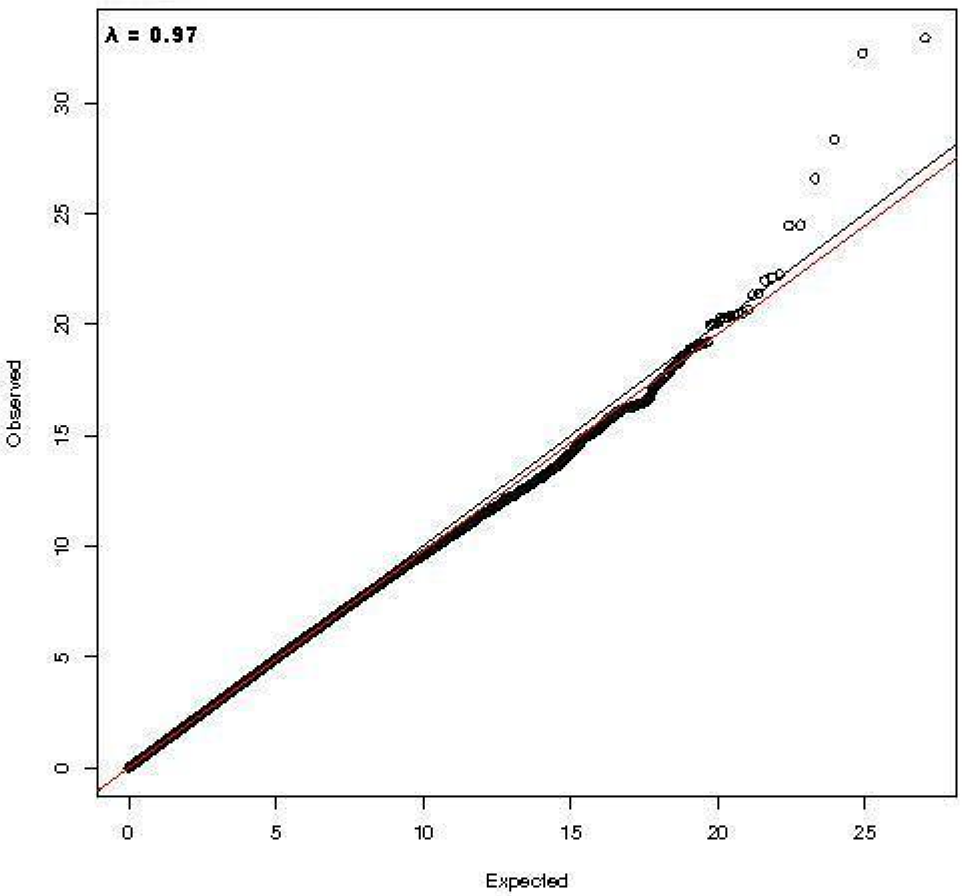

PC O 32 :1

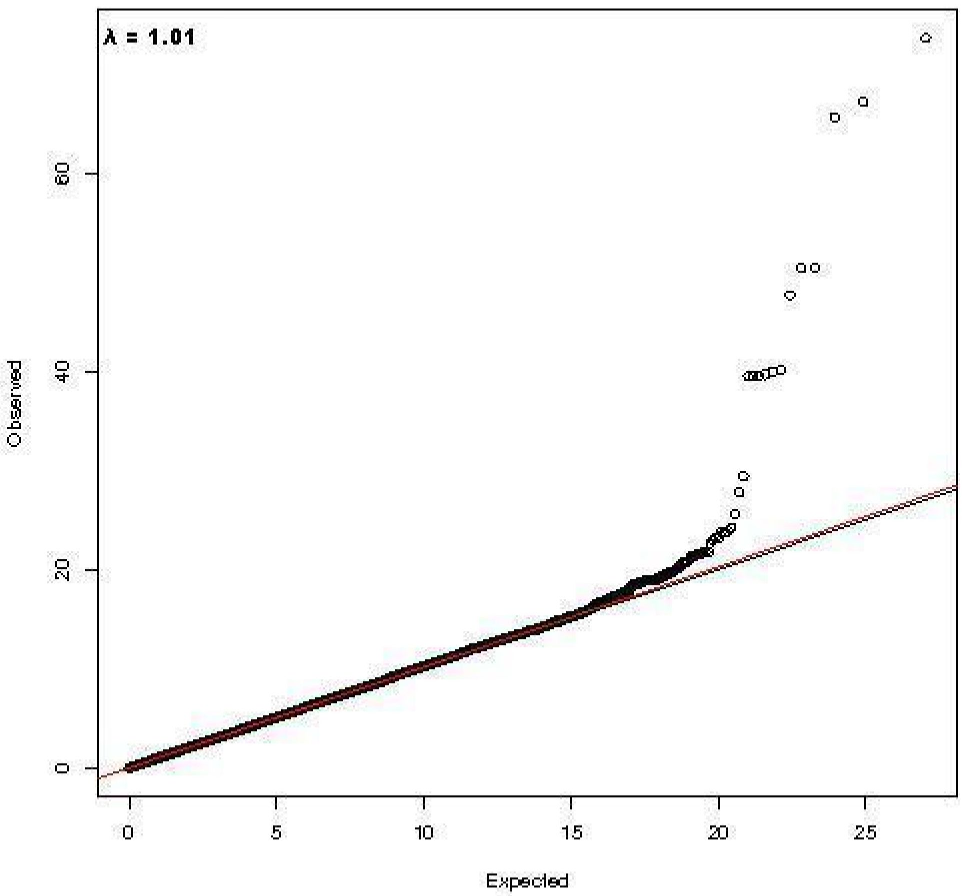

PC 32:1

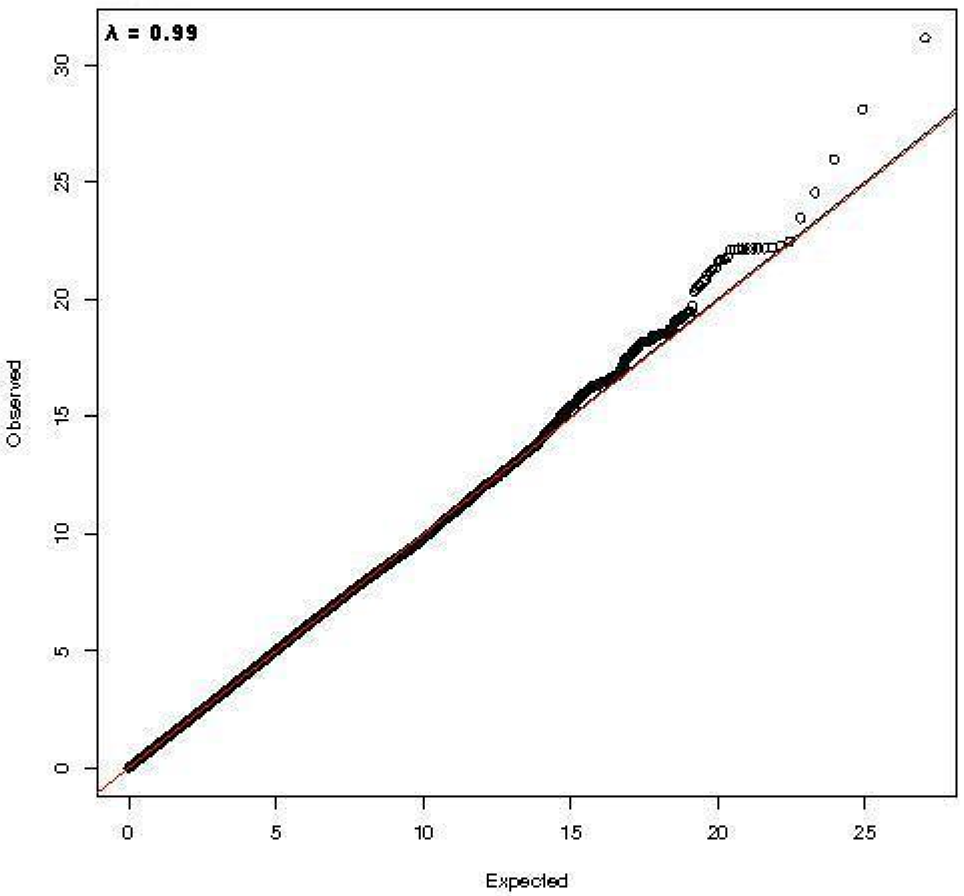
λ = 0.99
Observed
Expected
0
5
10
15
20
25
30

PC 34:4

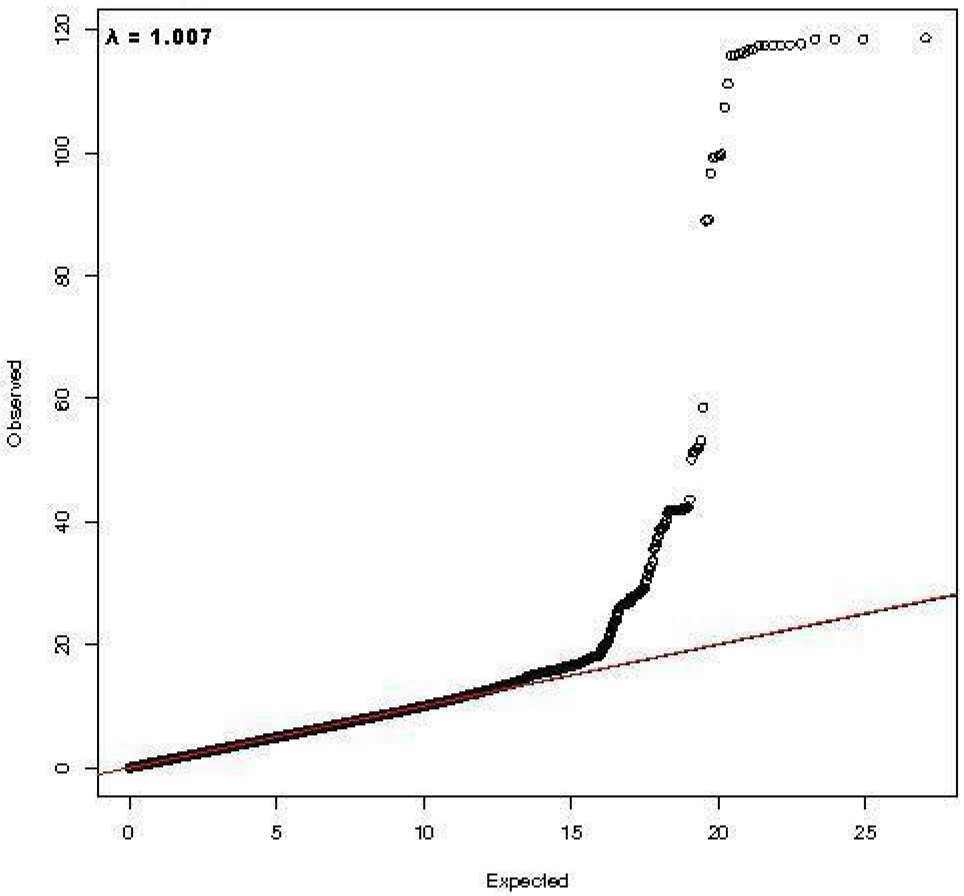
λ = 1.007
Observed
Expected
0
20
40
60
80
100
120
5
10
15
20
25

PC 40:3

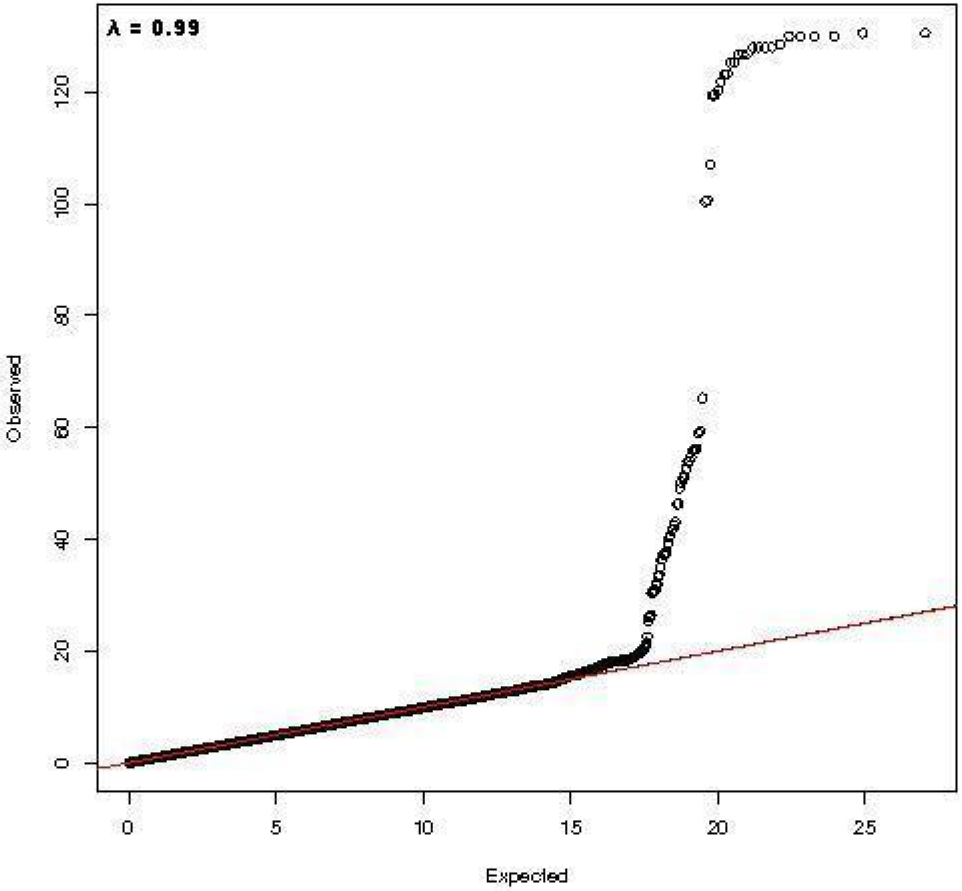

PC O 42:6

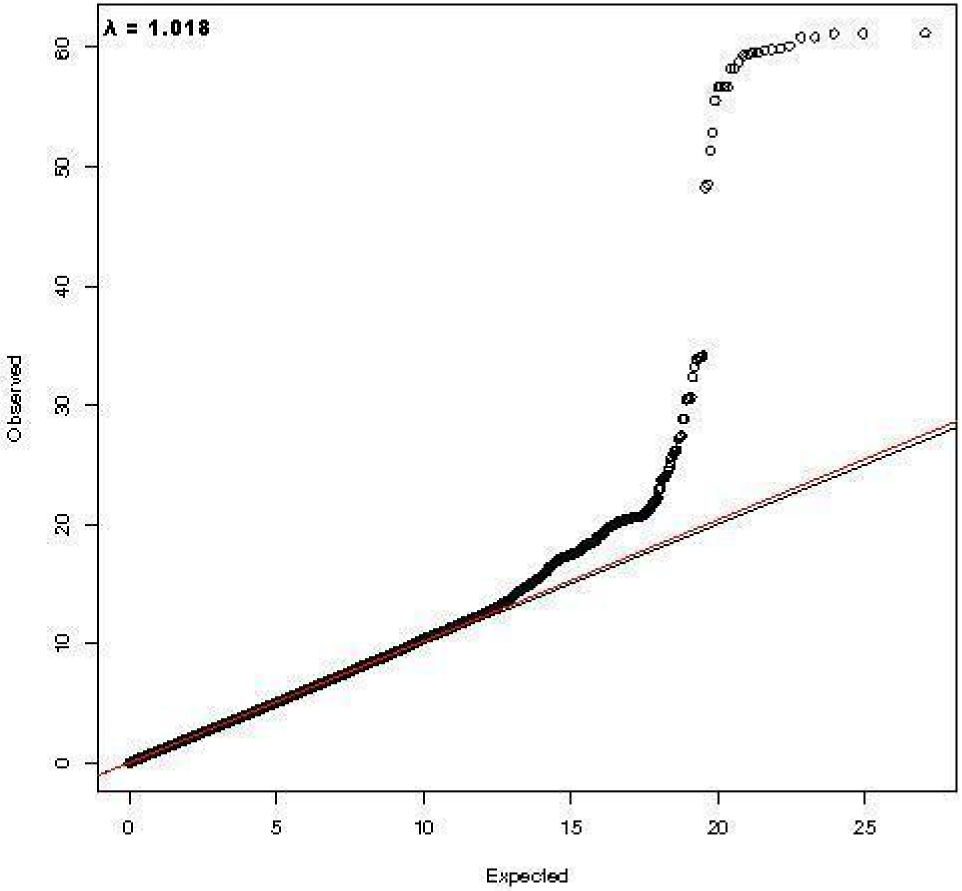

PC O 42:5

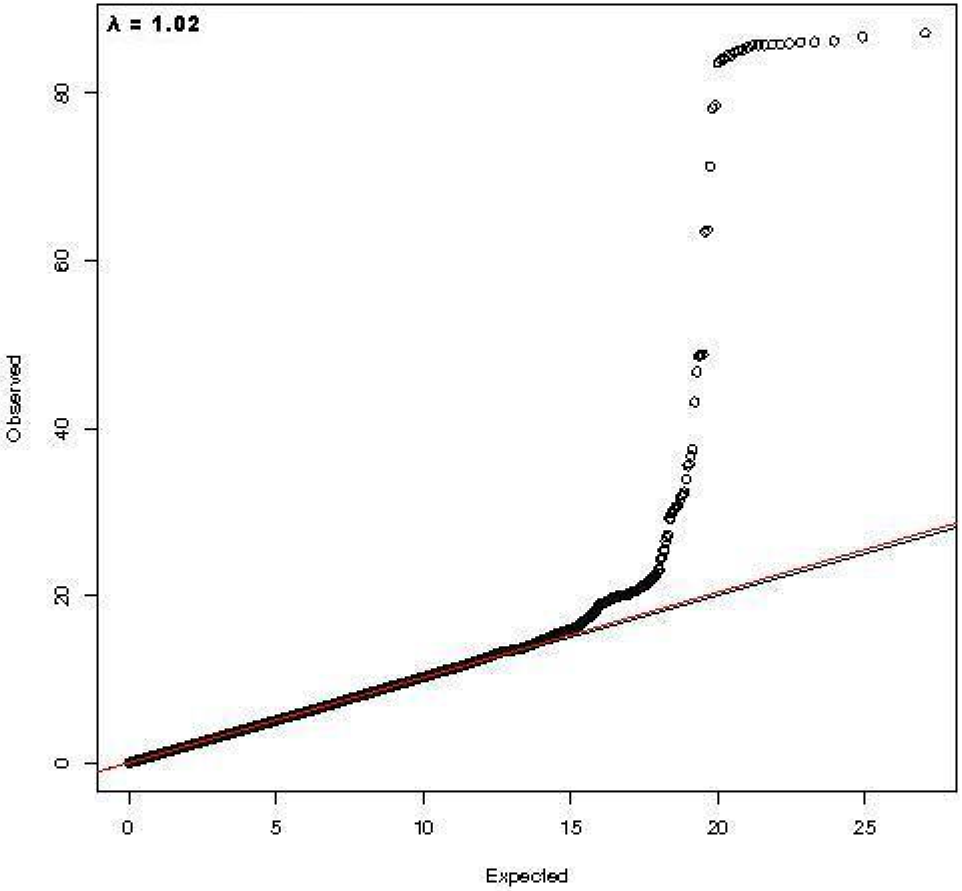

PC /LPC

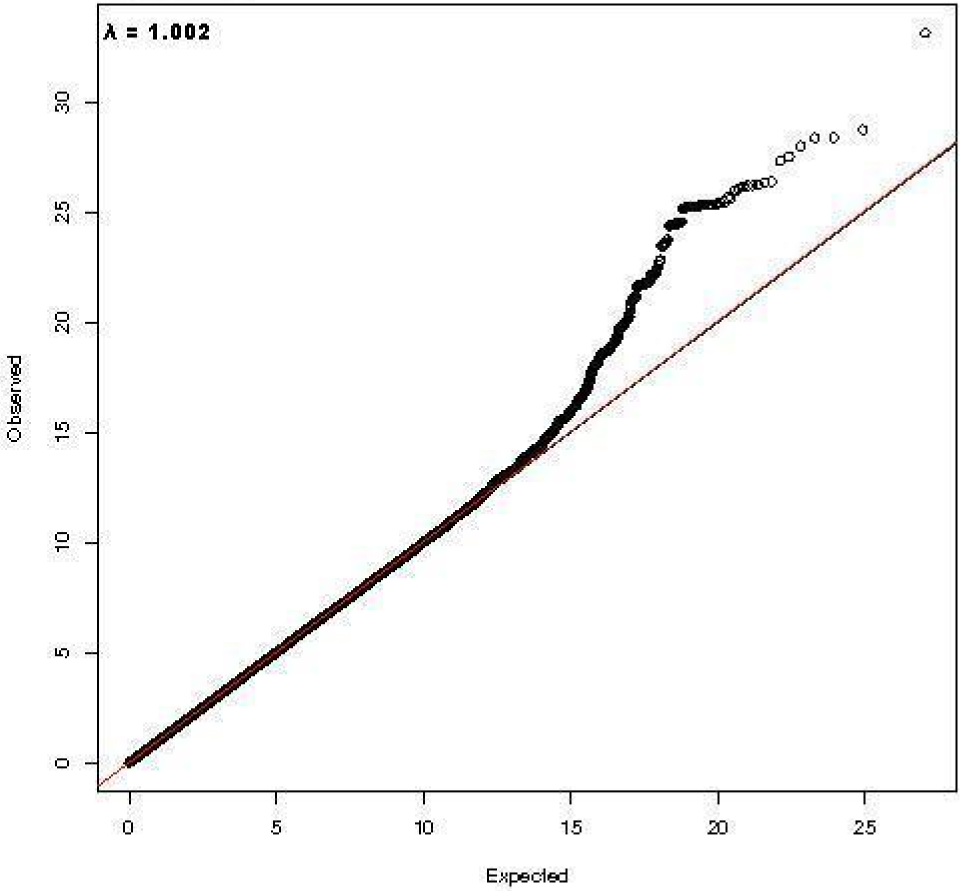

% PC O 32:0

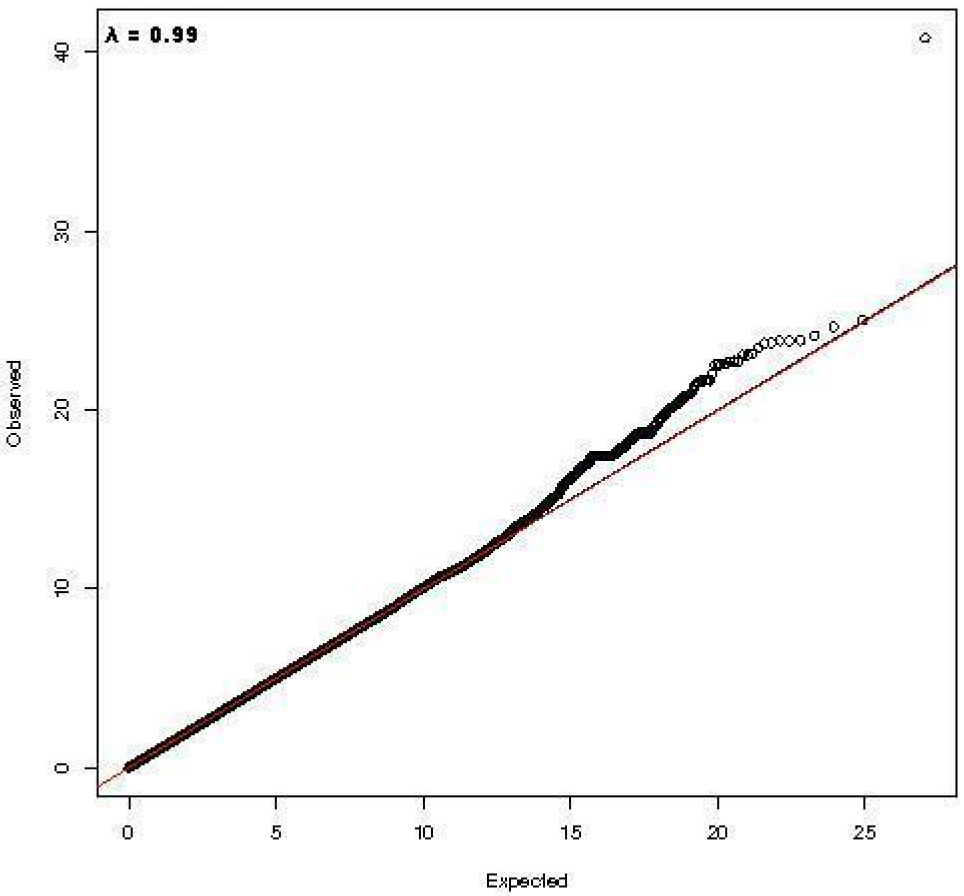

% PC 32:2

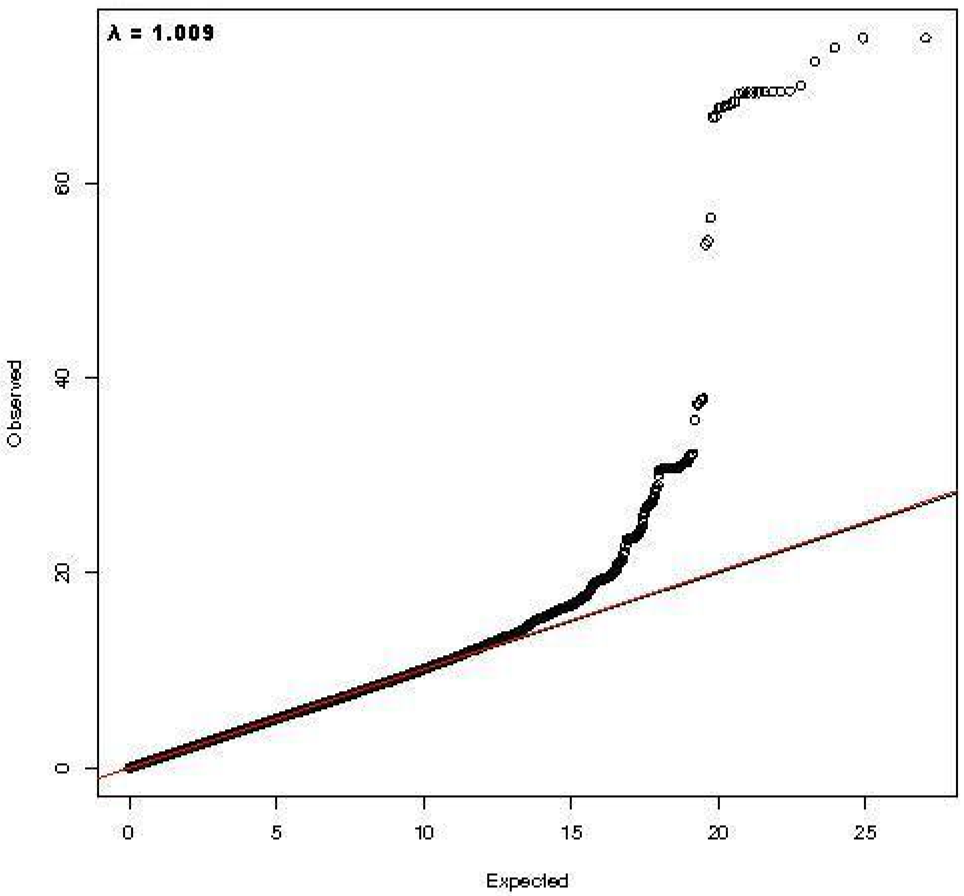

% PC 32:0

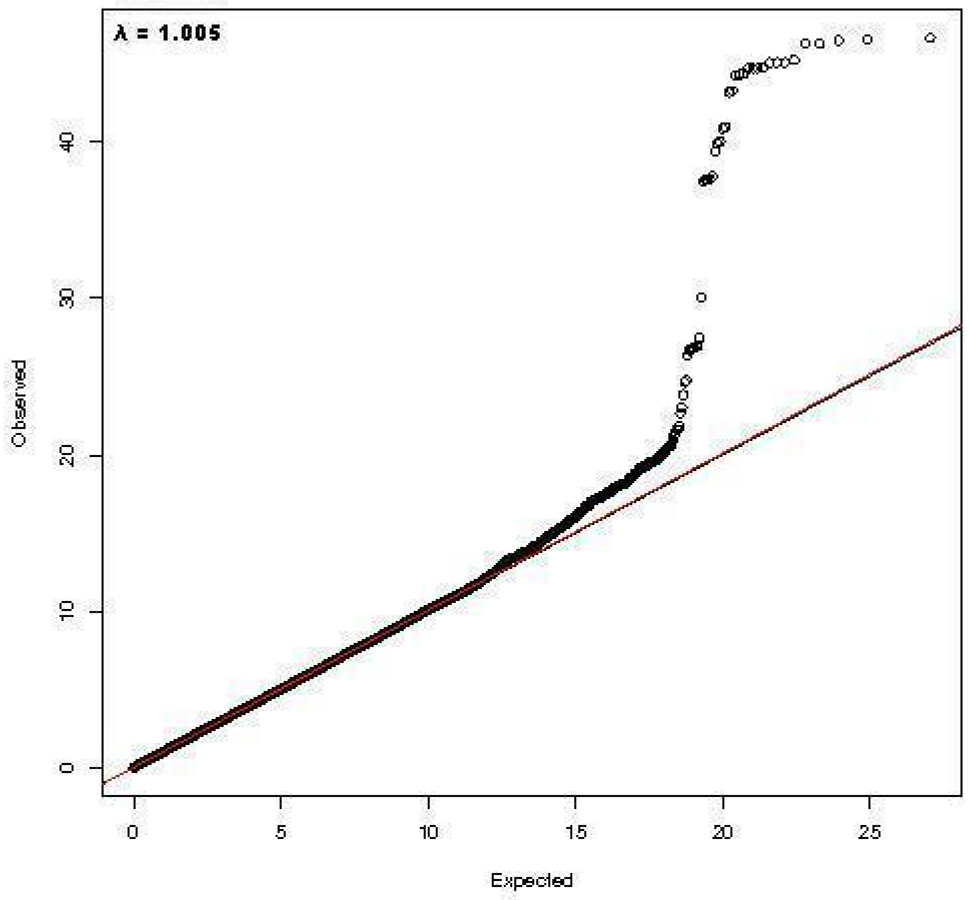

% PC O 36:5

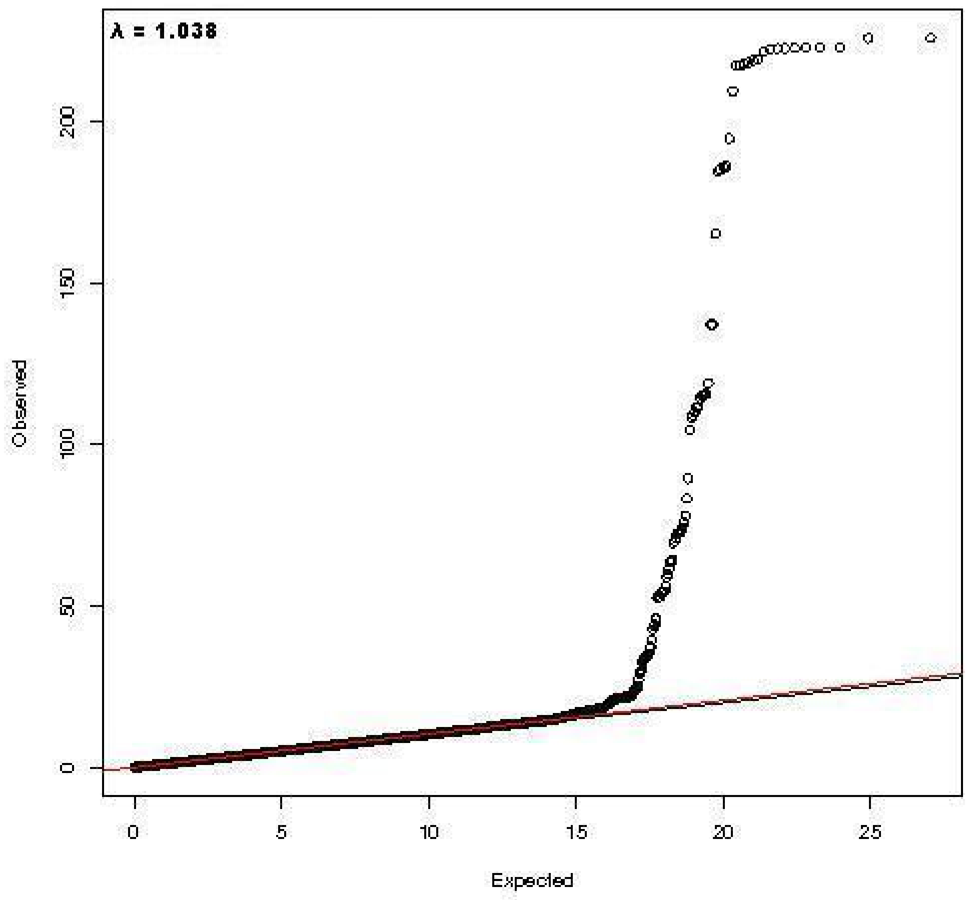

% PC 36:4

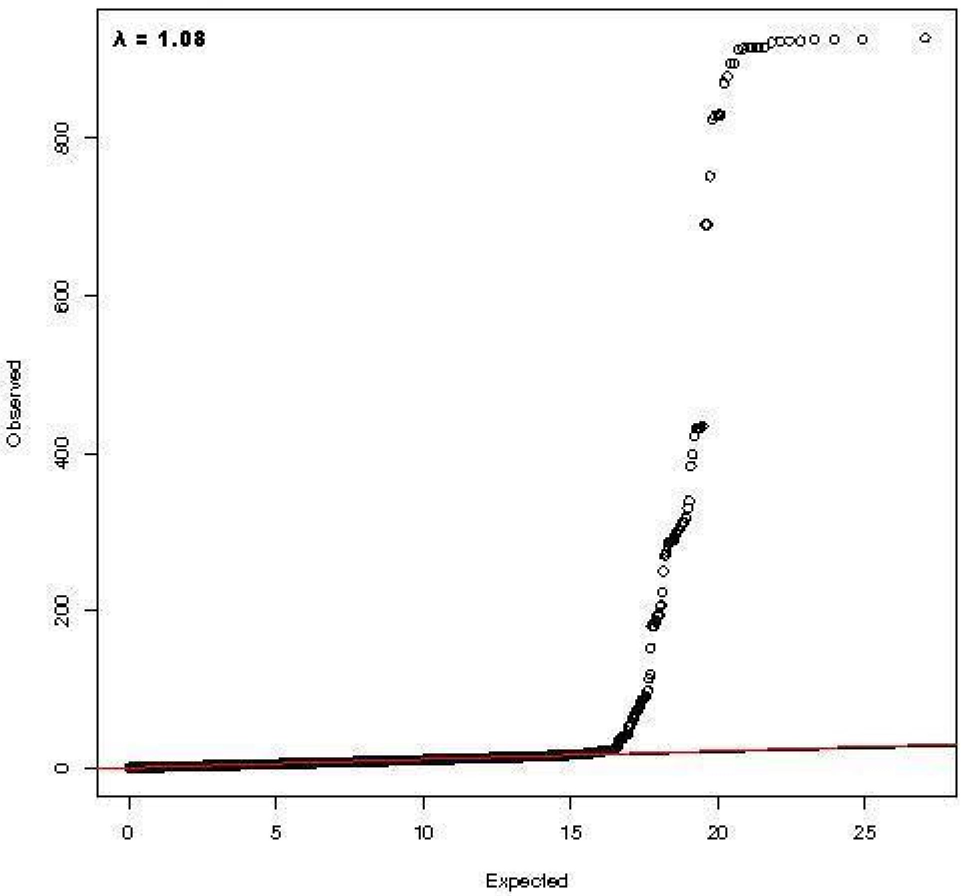
λ = 1.08
Observed
Expected
0
200
400
600
800
0
5
10
15
20
25

% PC 36:1

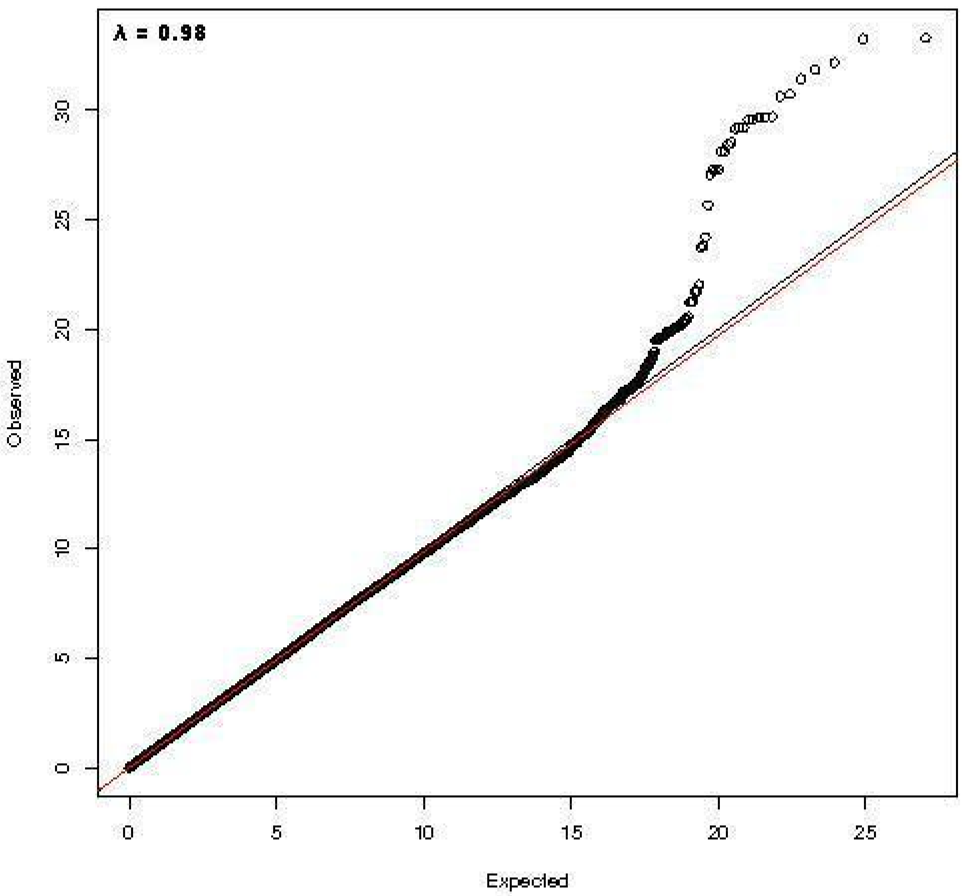
λ = 0.98
Observed
Expected
0
5
10
15
20
25
30
0
5
10
15
20
25

PC 38:5

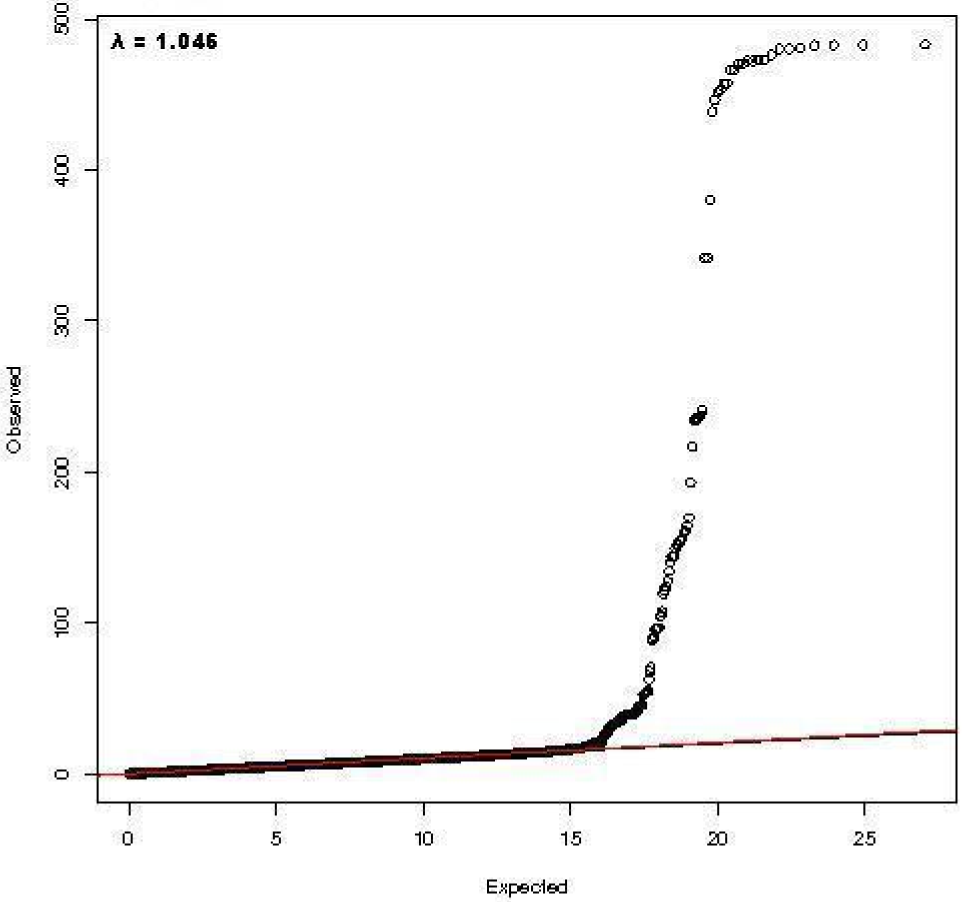
λ = 1.046
0
100
200
300
400
500
Observed
0
5
10
15
20
25
Expected

PE 36:4

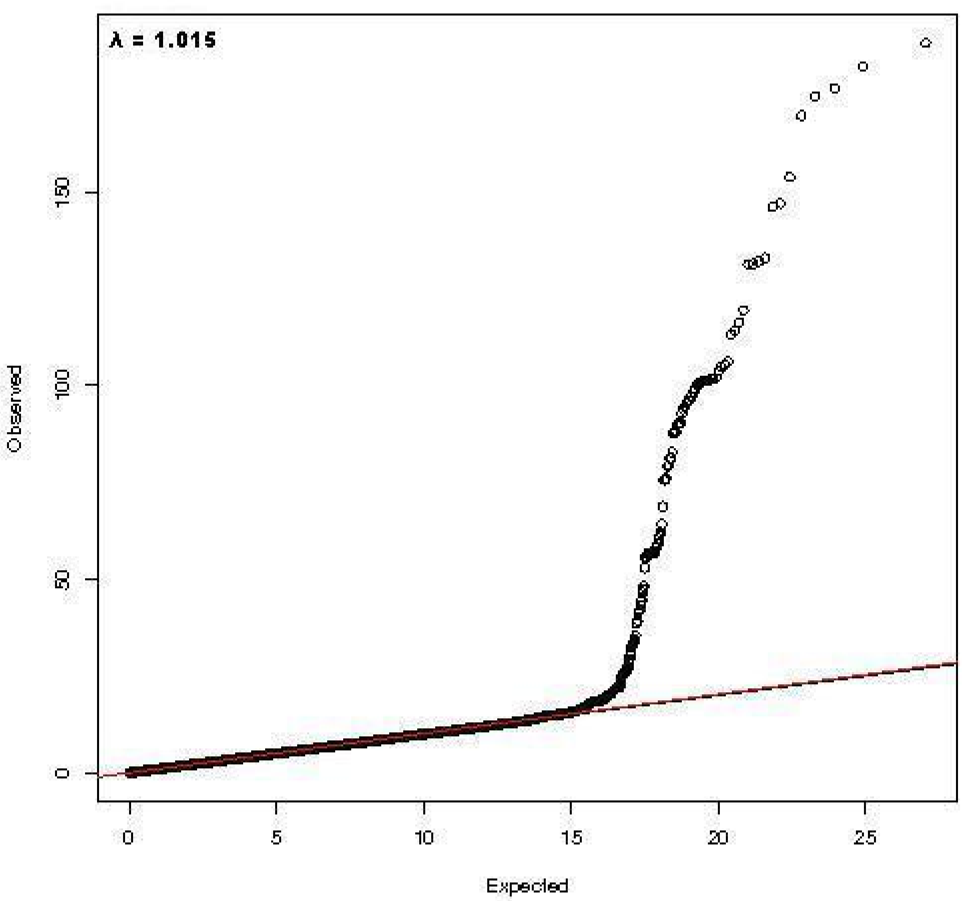
λ = 1.015
0
50
100
150
Observed
0
5
10
15
20
25
Expected

% Mono-unsaturated PE

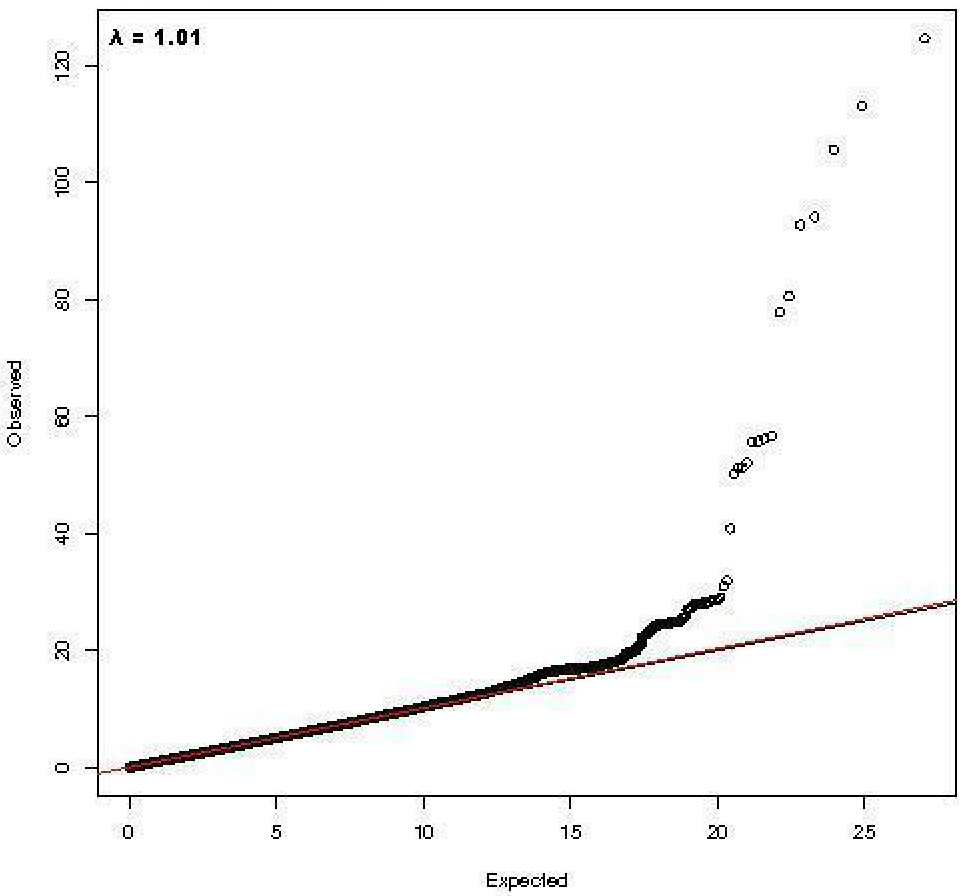

PLPE 18:0/22:6

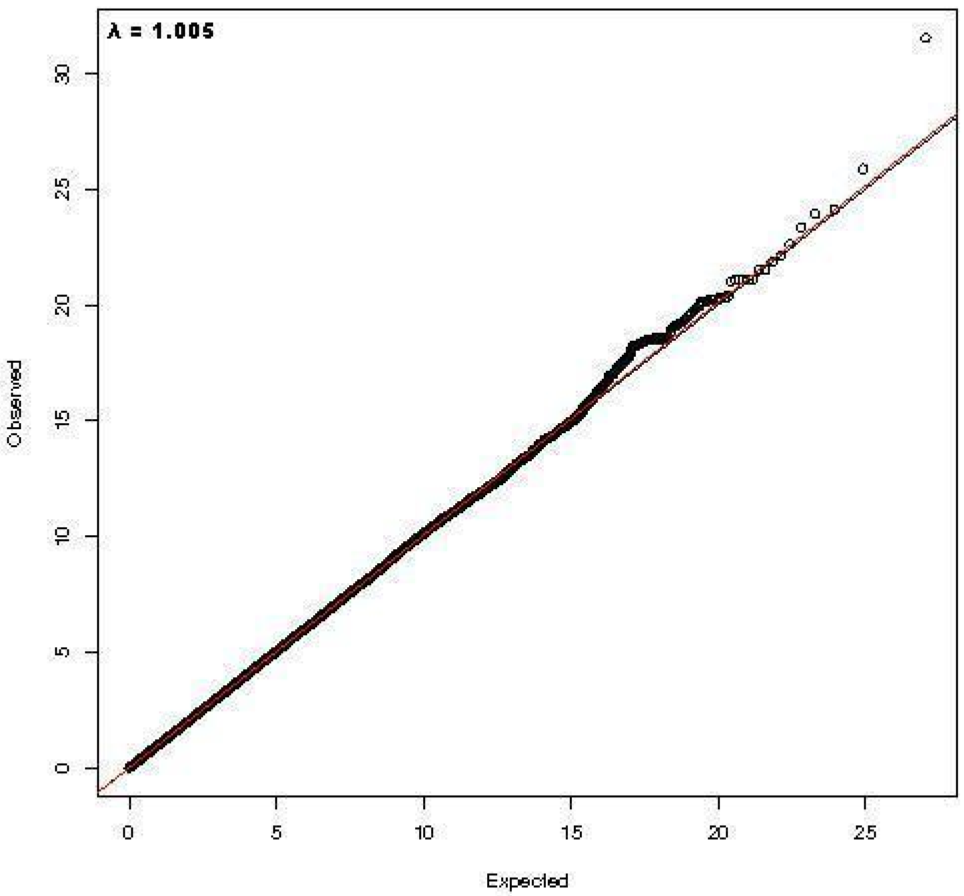

SPM 16:1

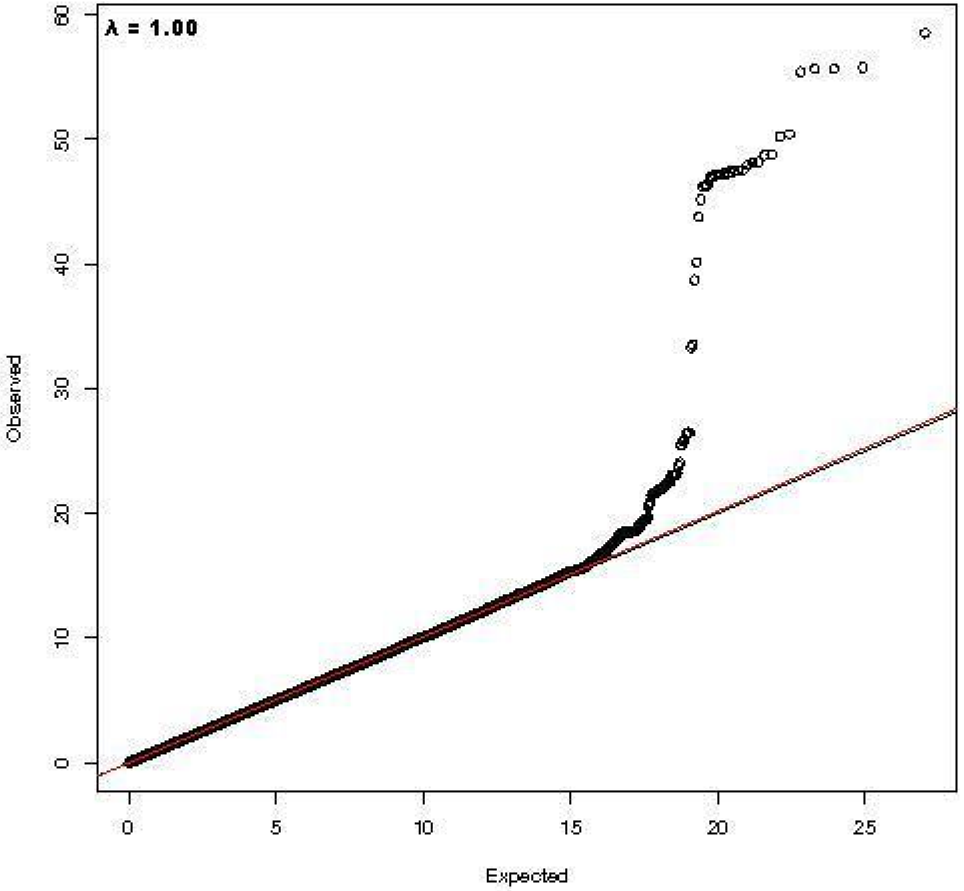

SPM 17:0

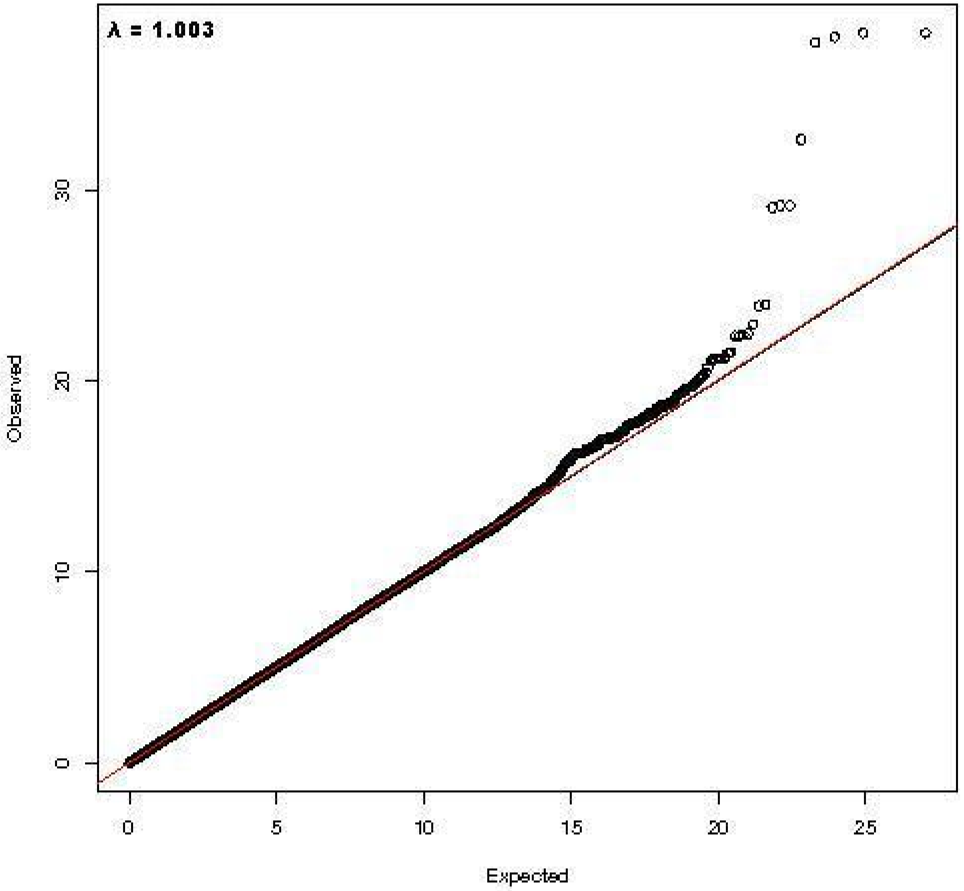

SPM 22:0

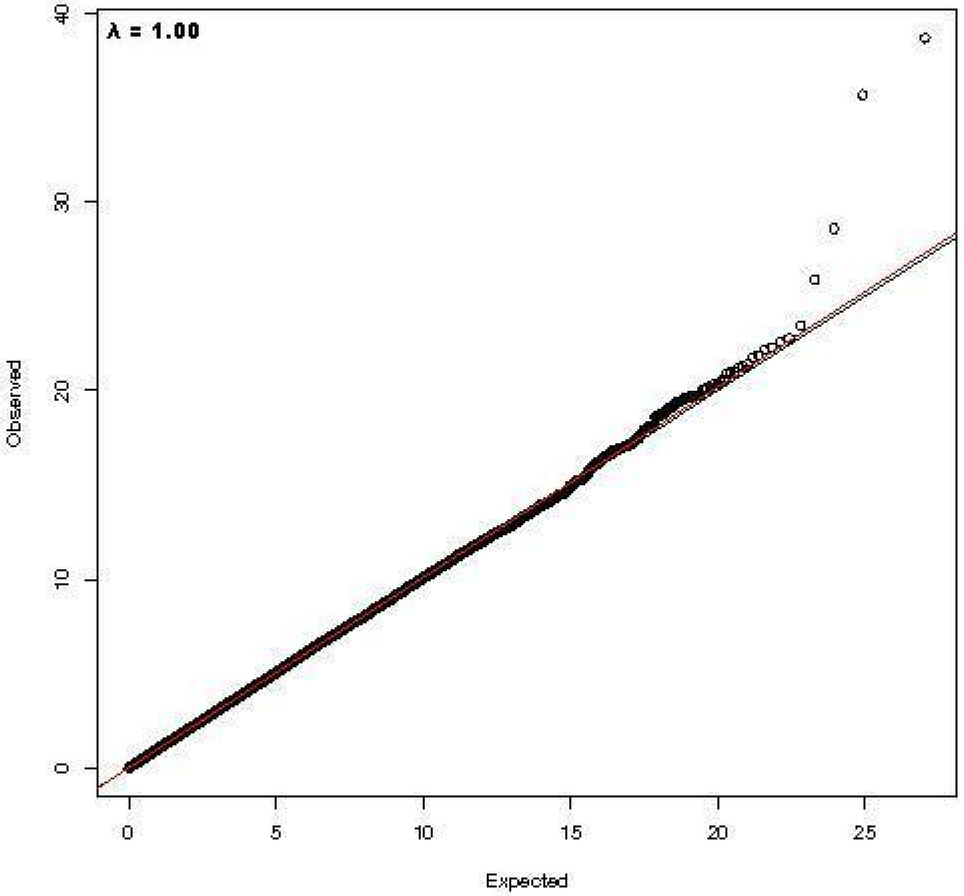

SPM 23:0

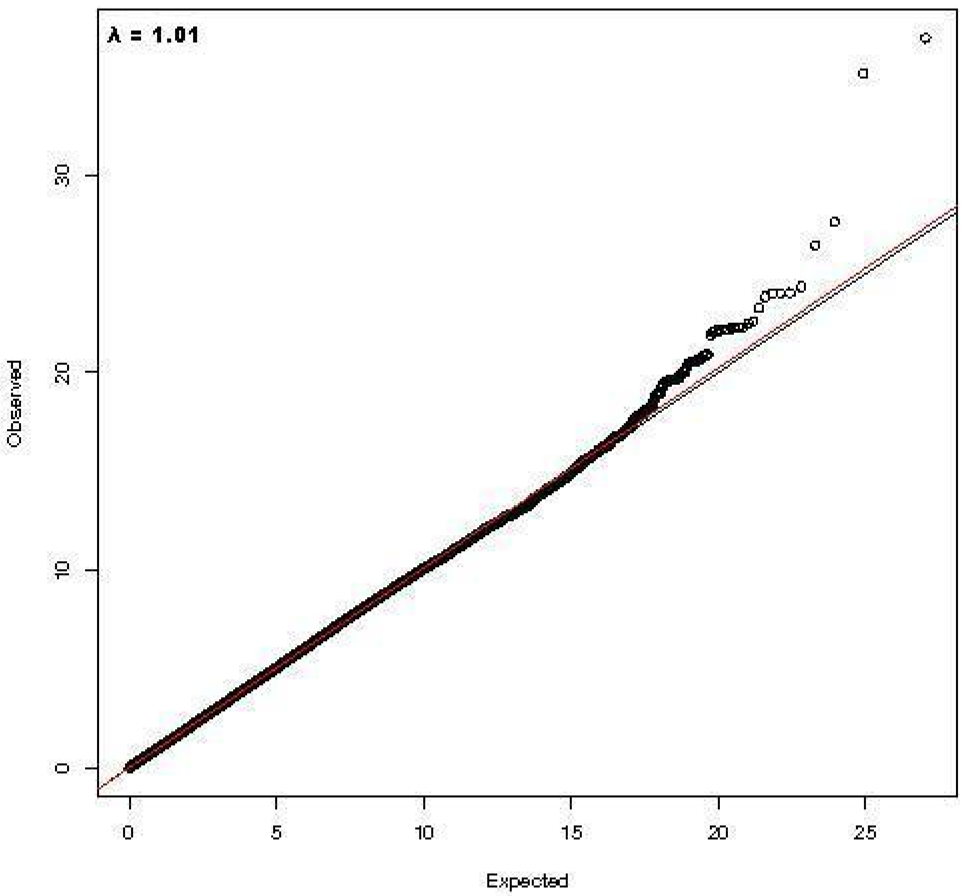

%SPM 14:0

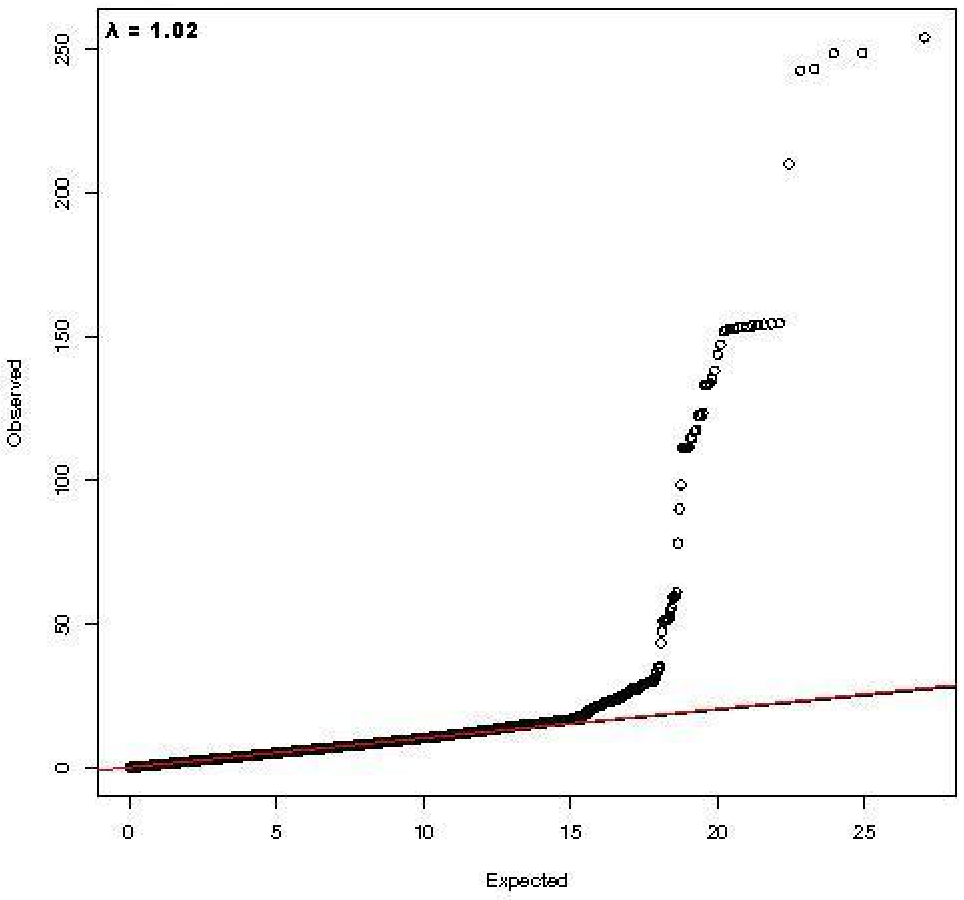

% SPM 20:1

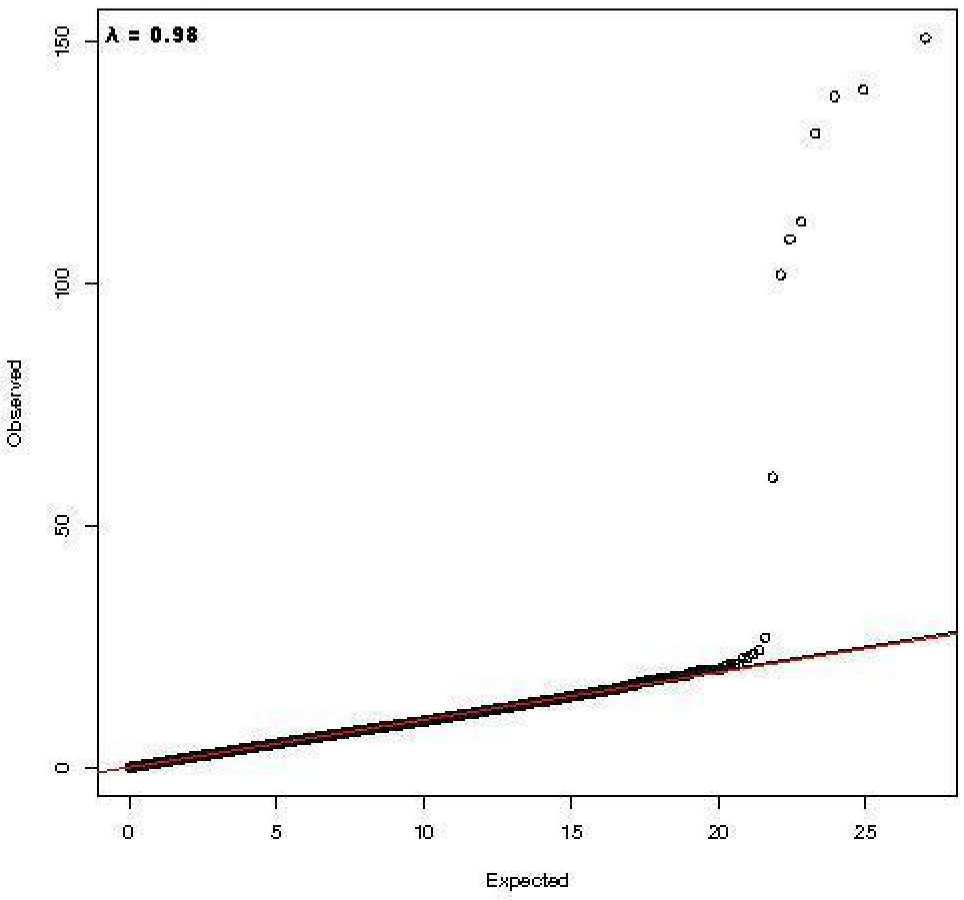

Supplement: Figure S1 — Q-Q Plots from the GWAS of phospho- and sphingolipid traits with genome-wide significant findings. The x-axis shows the expected chi-square value, the y-axis shows the observed. Lambda (λ): Genomic control inflation factor. (PDF) [file pgen.1002490.s001.pdf]
