# Supplementary figures and images for: Genome-Wide Association Study Identifies Novel Loci Associated with Circulating Phospho- and Sphingolipid Concentrations
Source: PLoS Genet. 2012 Feb 16;8(2):e1002490. doi: 10.1371/journal.pgen.1002490 (PMC3280968; doi:10.1371/journal.pgen.1002490)

Figure S2  
Regional association plots of phospholipid associated loci

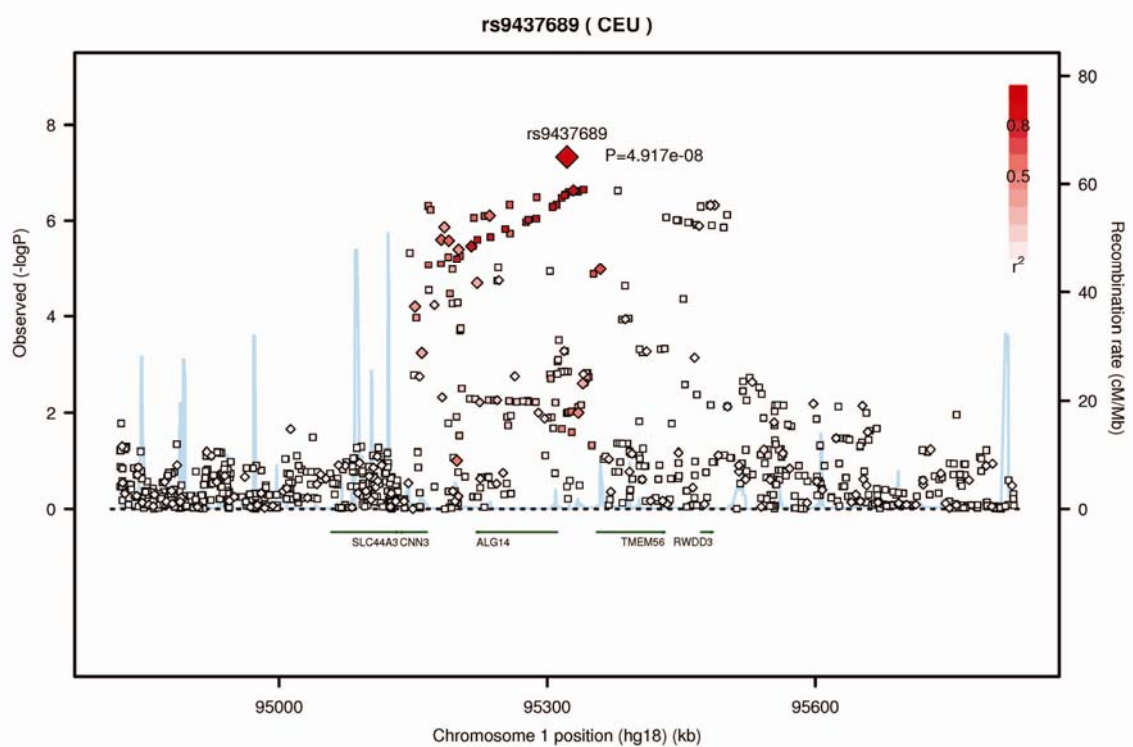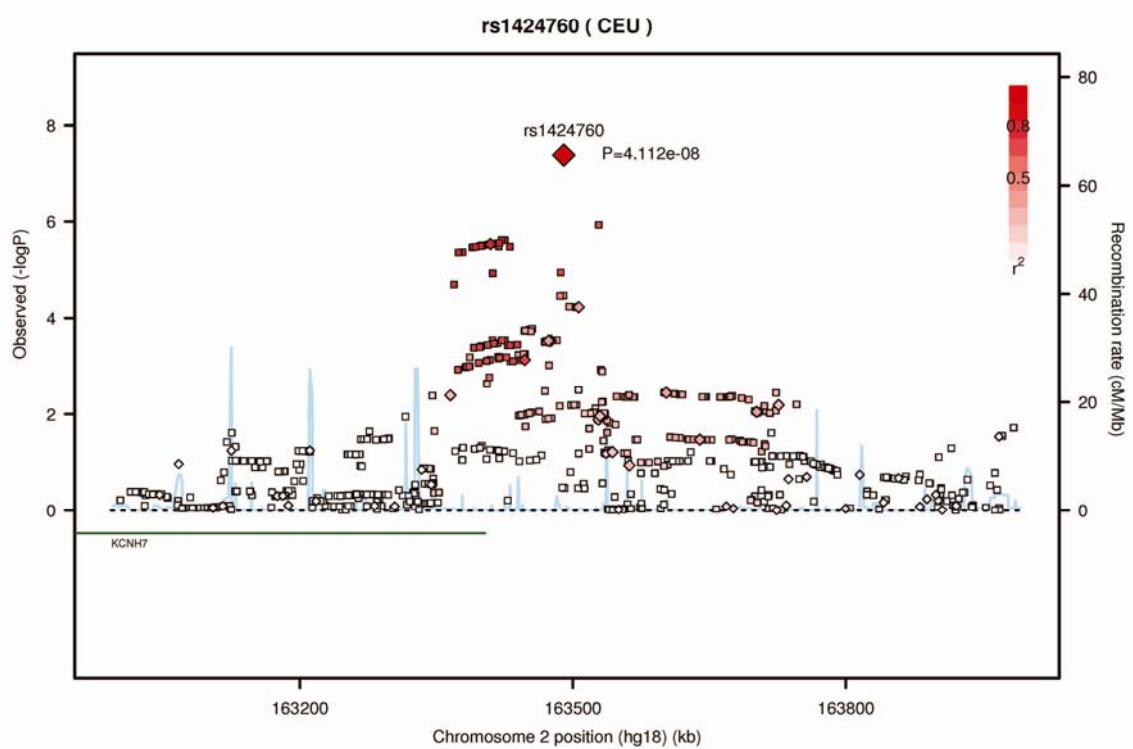

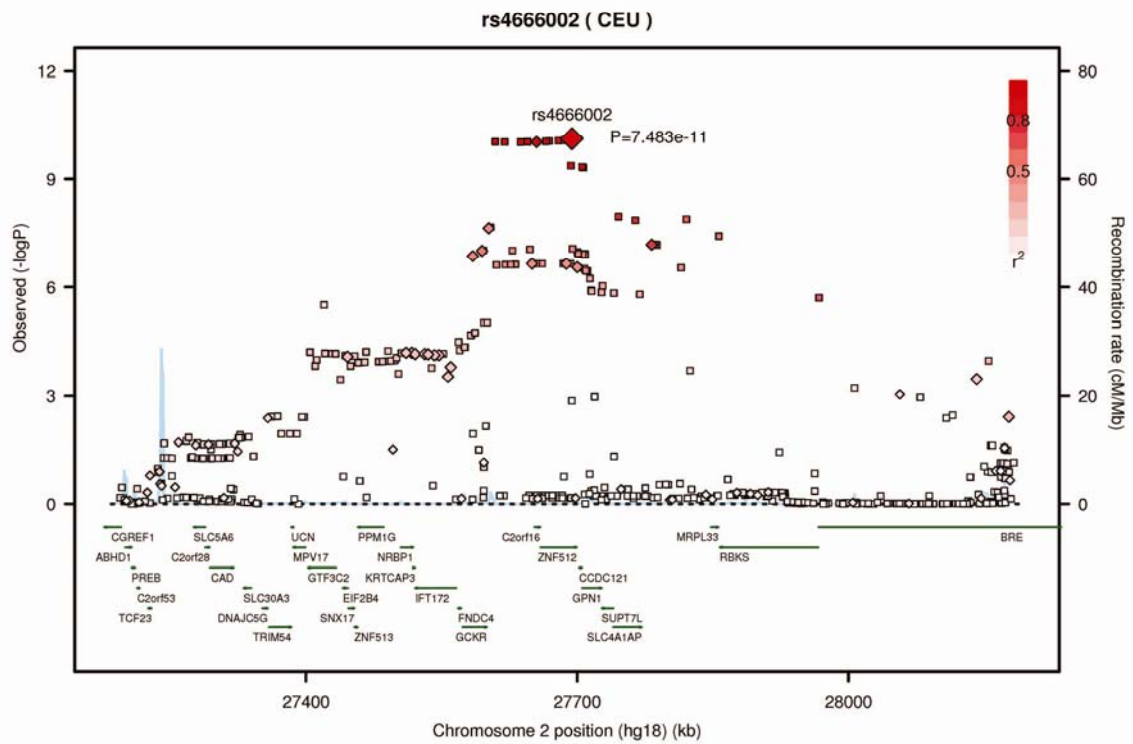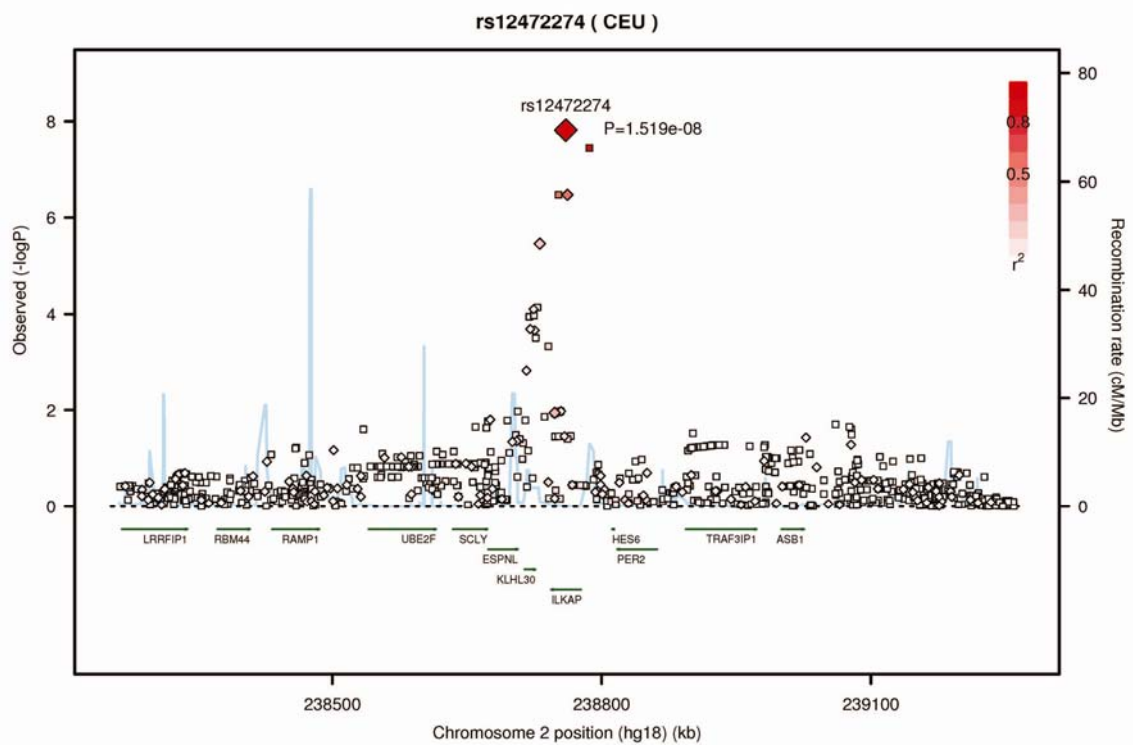

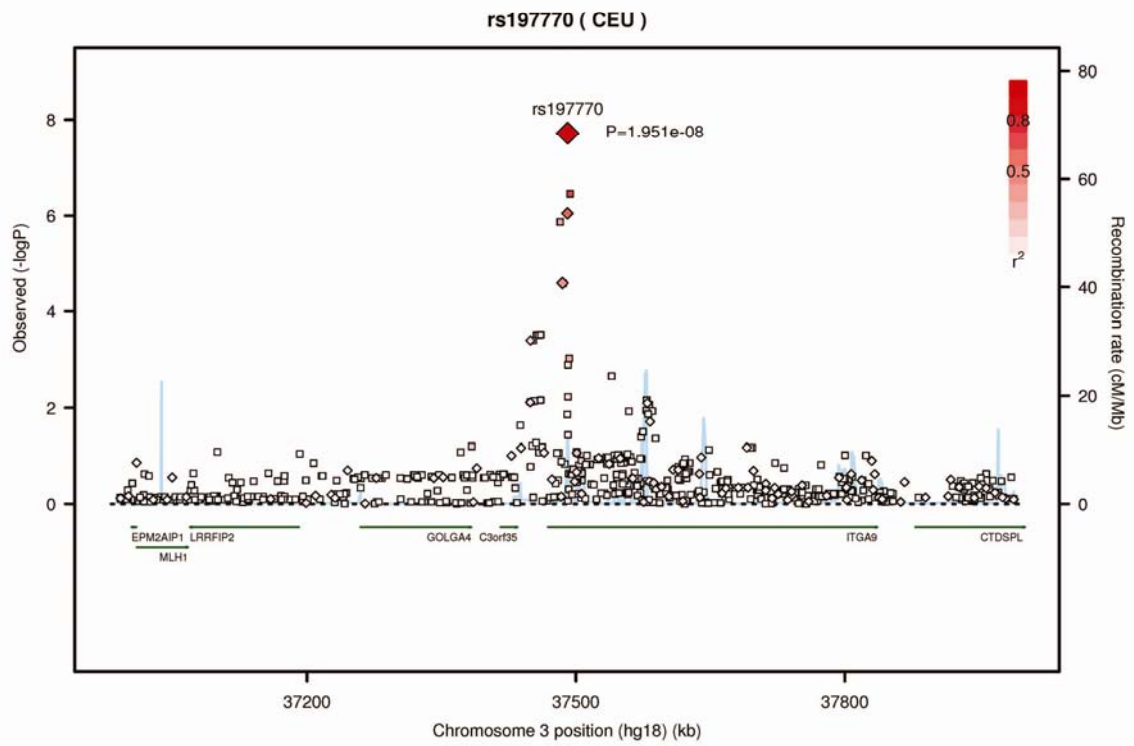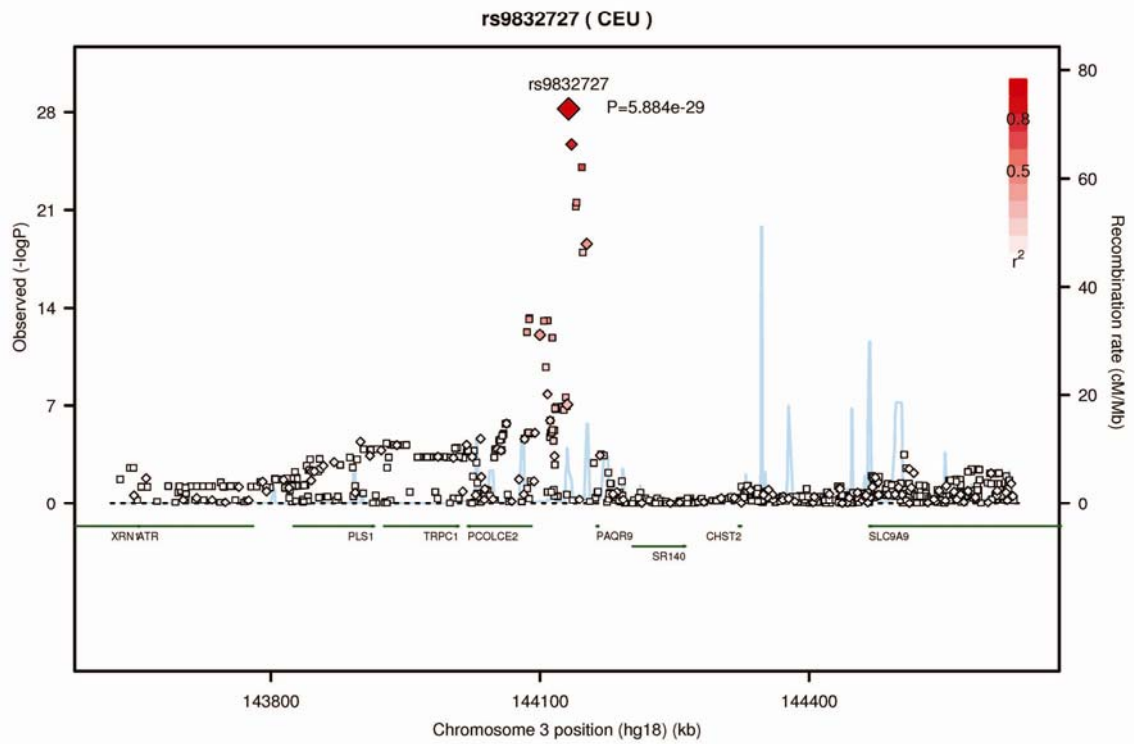

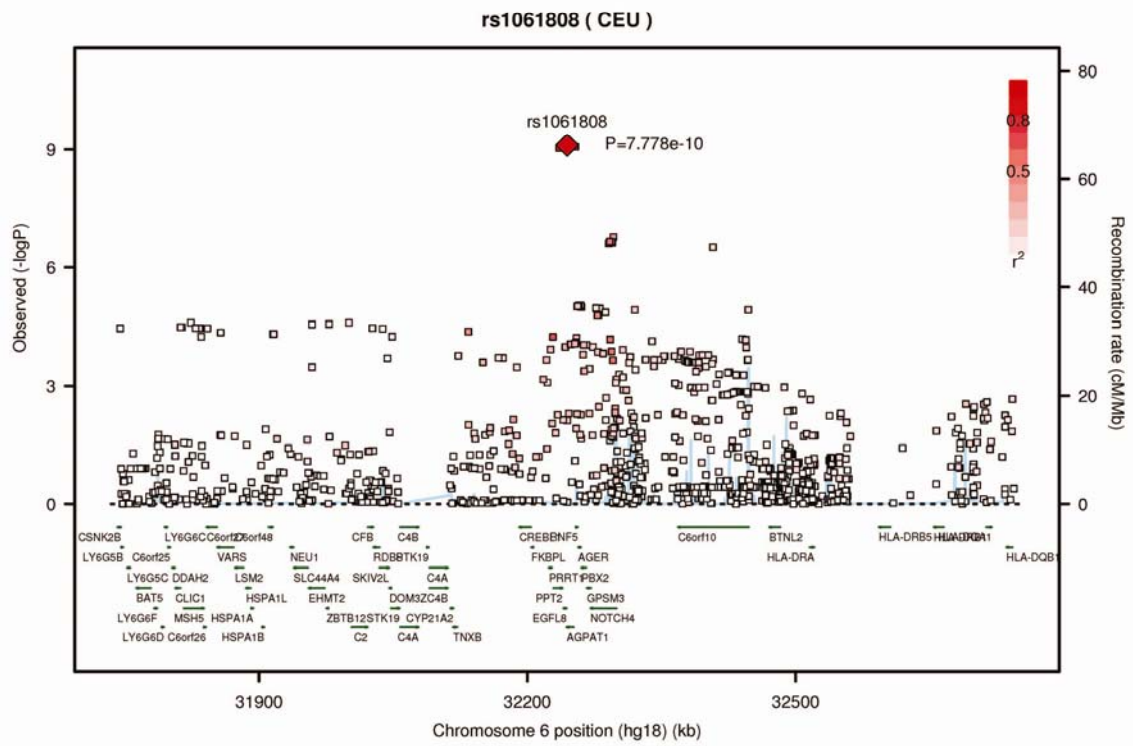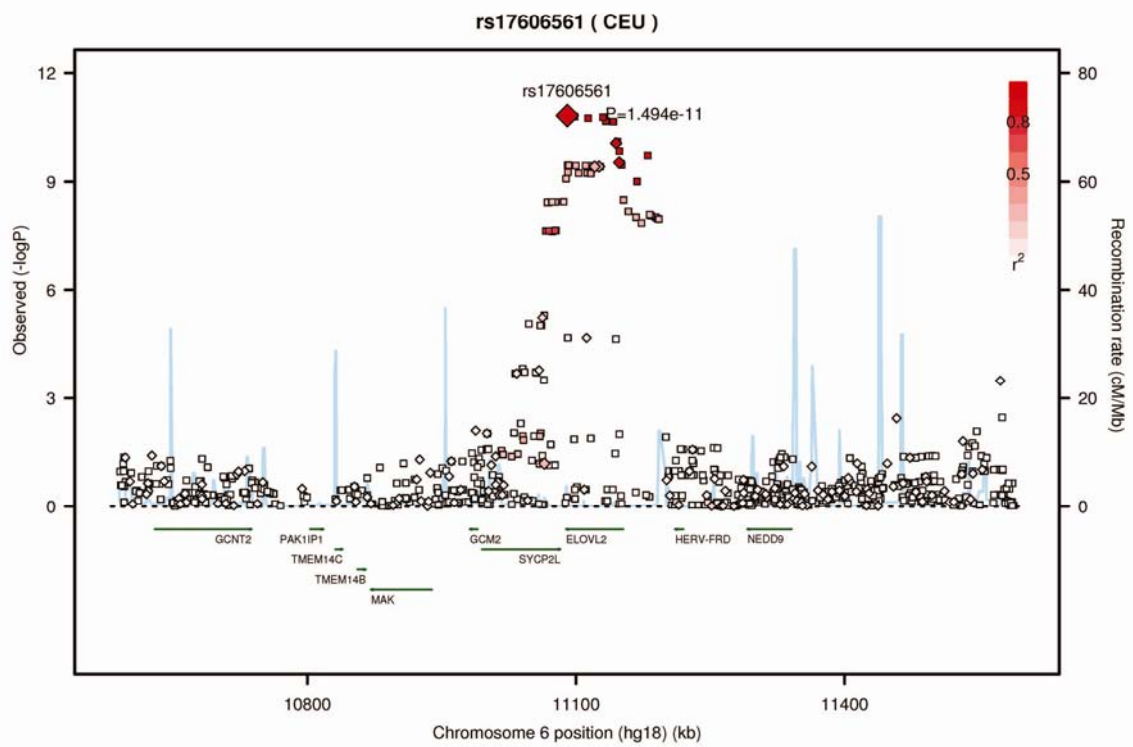

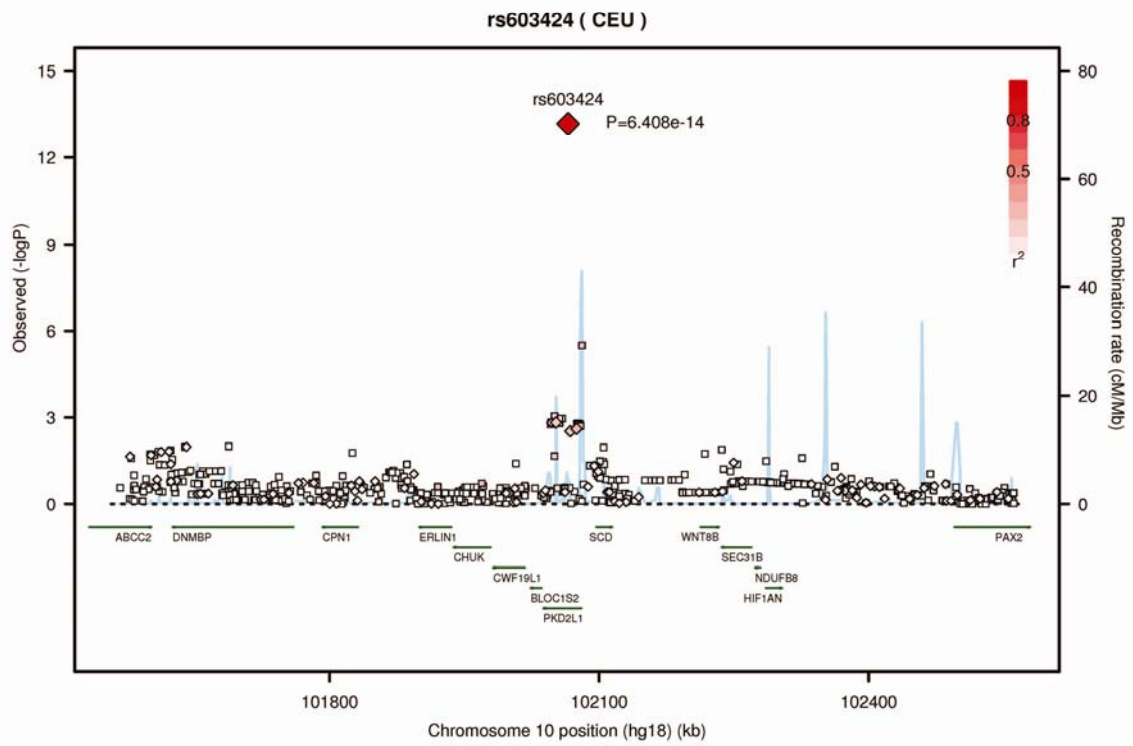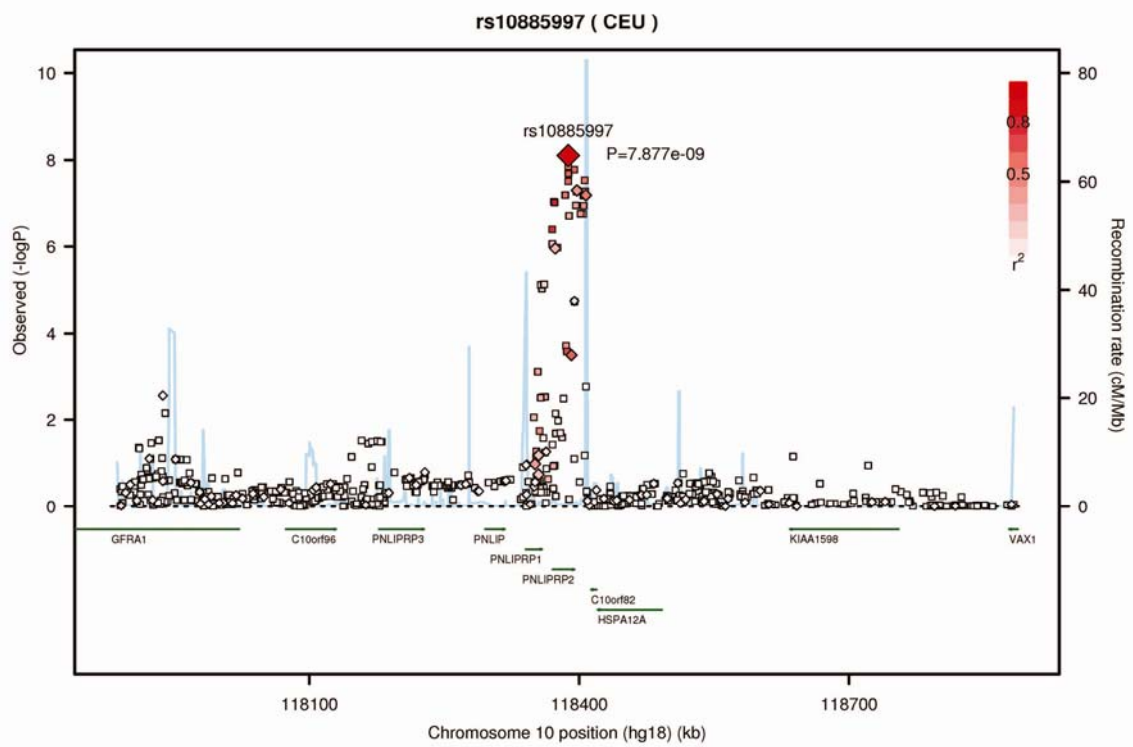

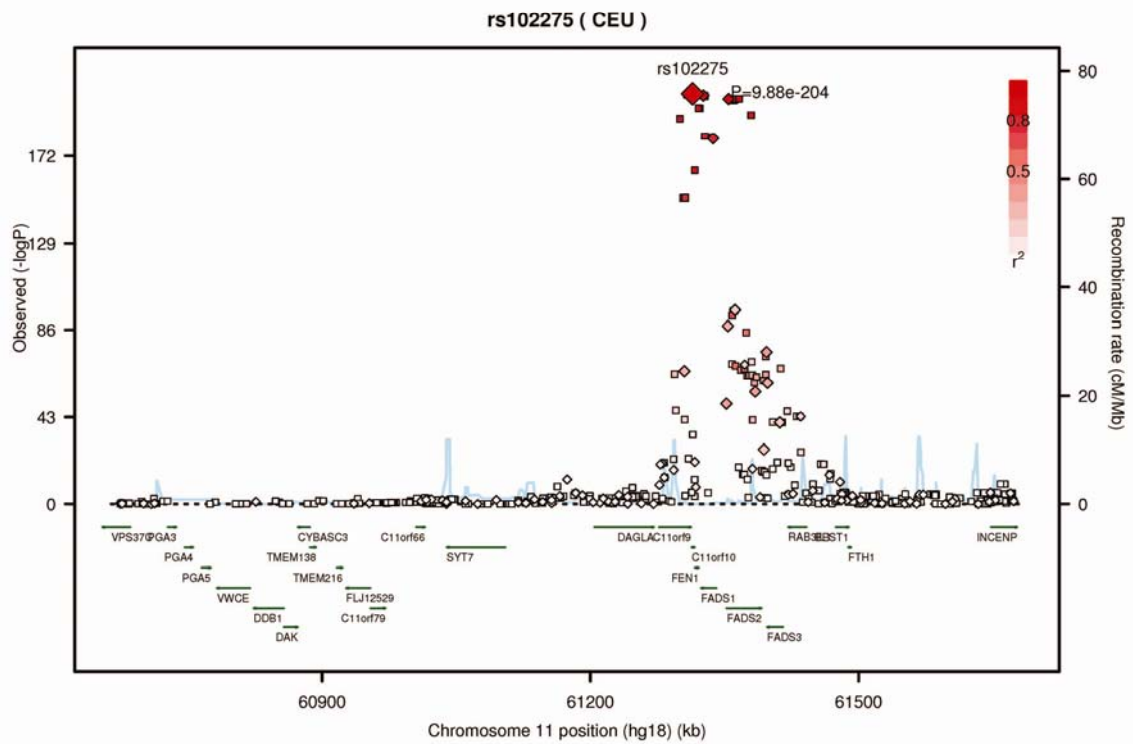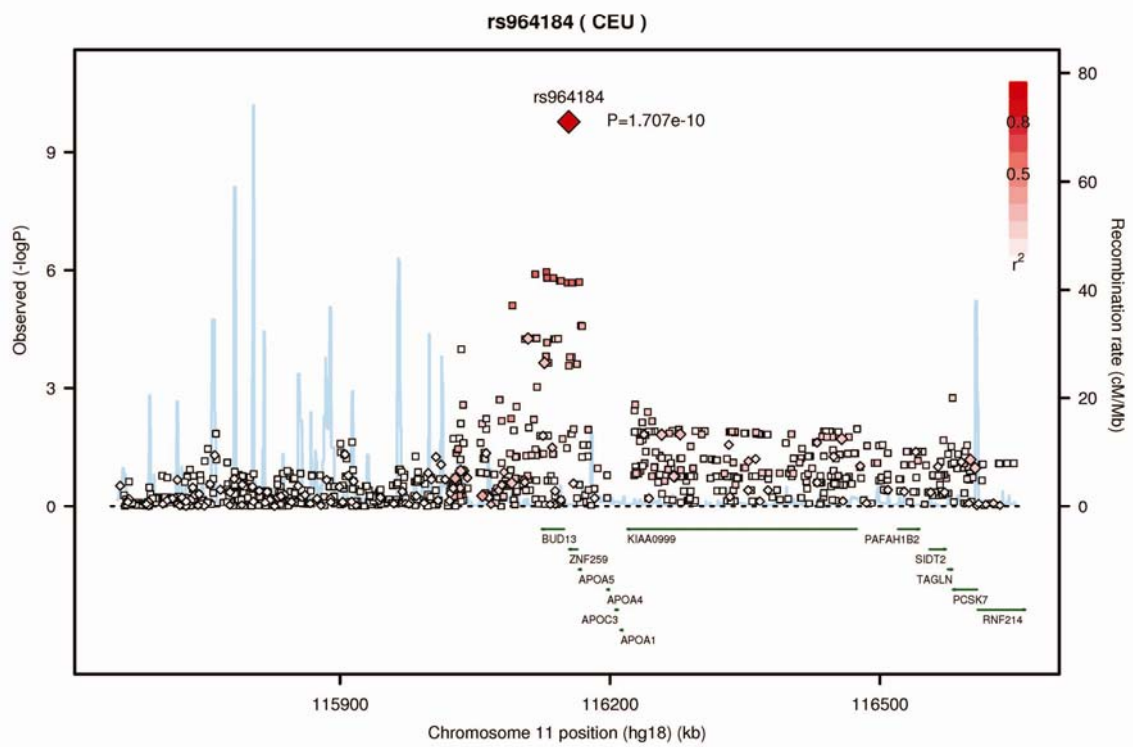

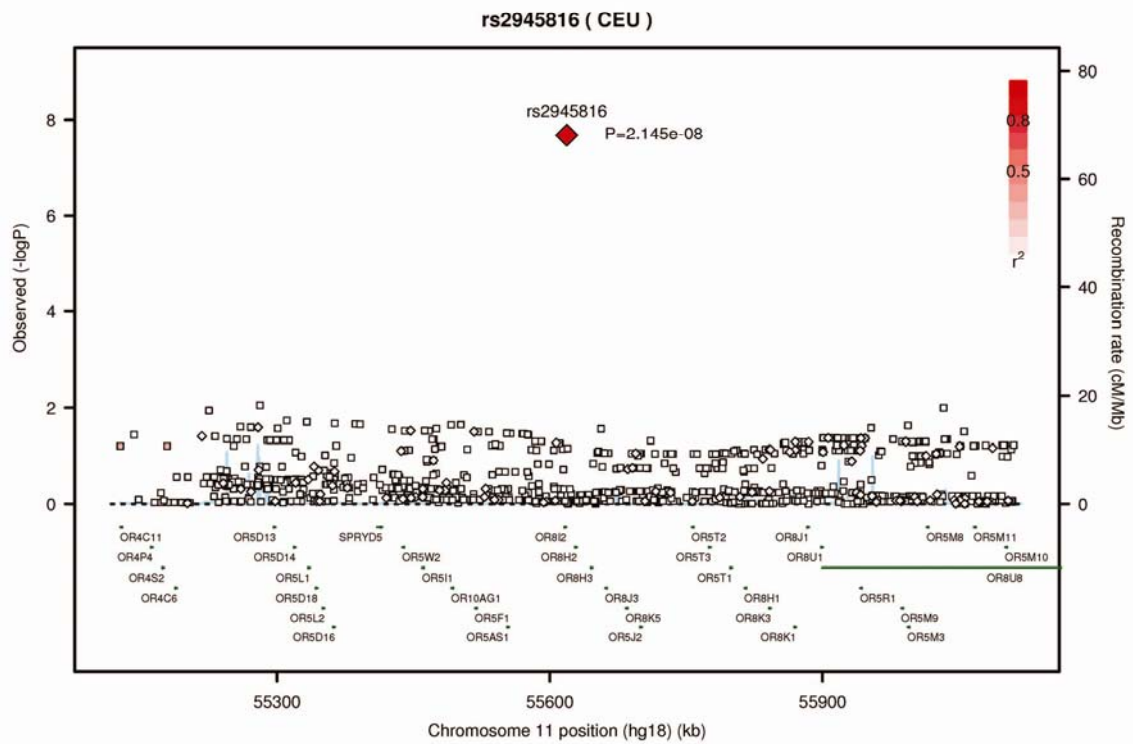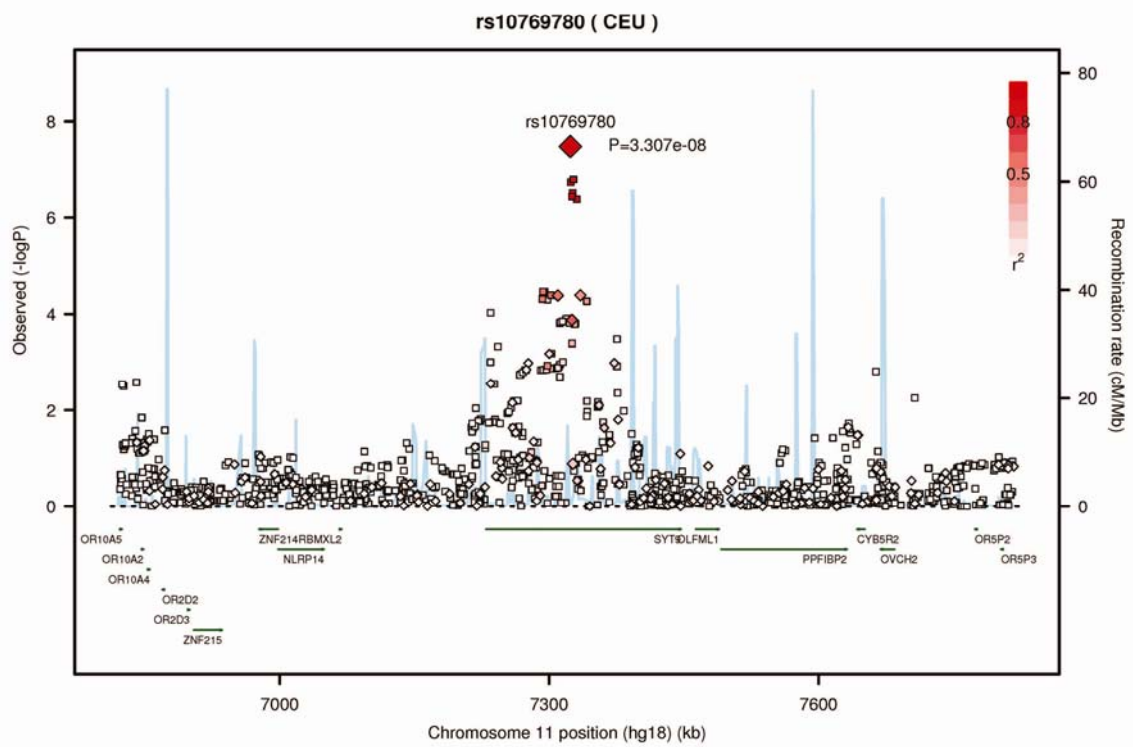

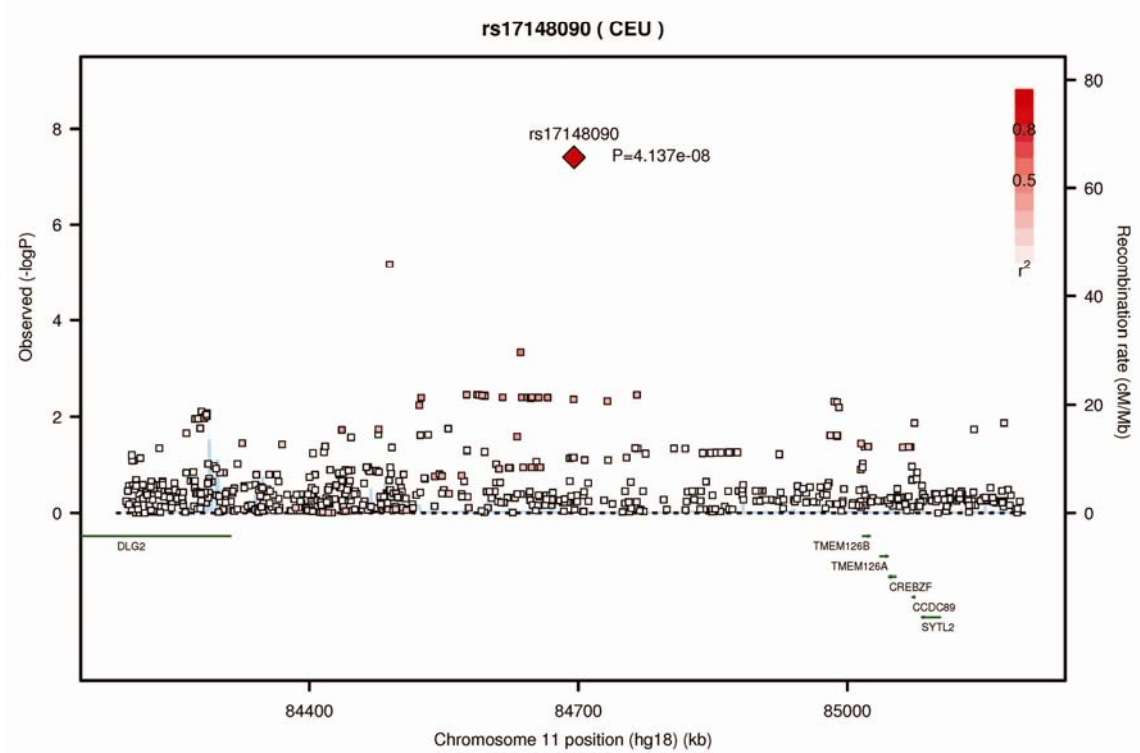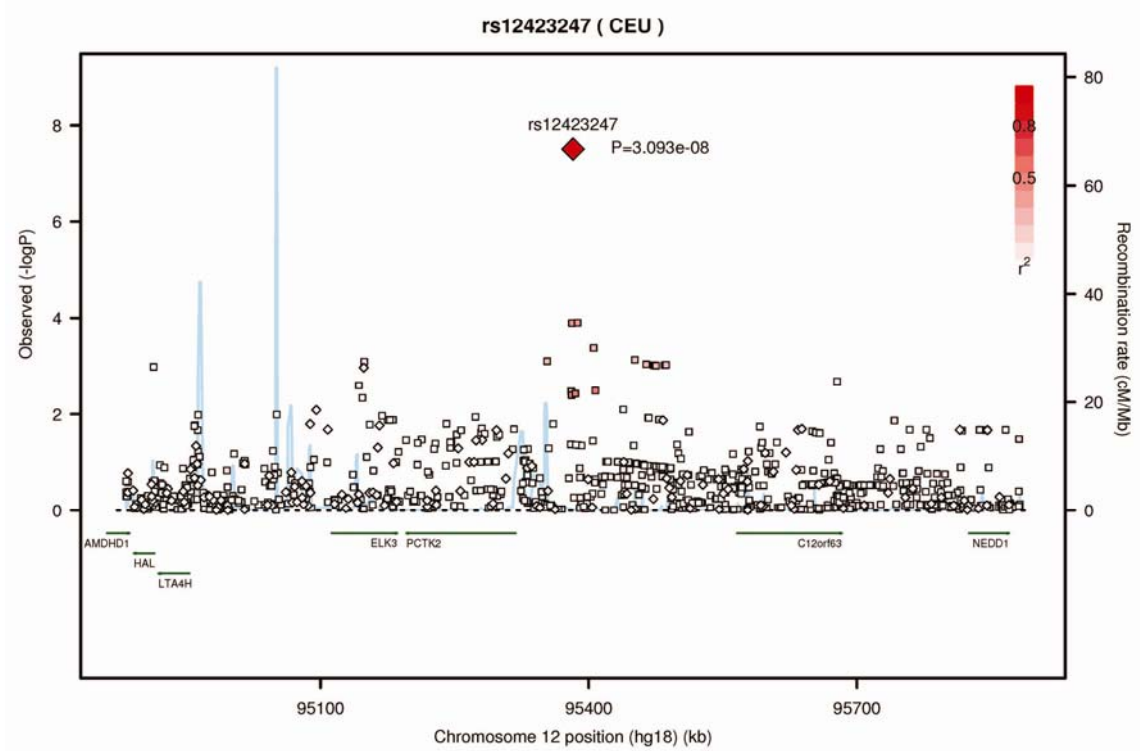

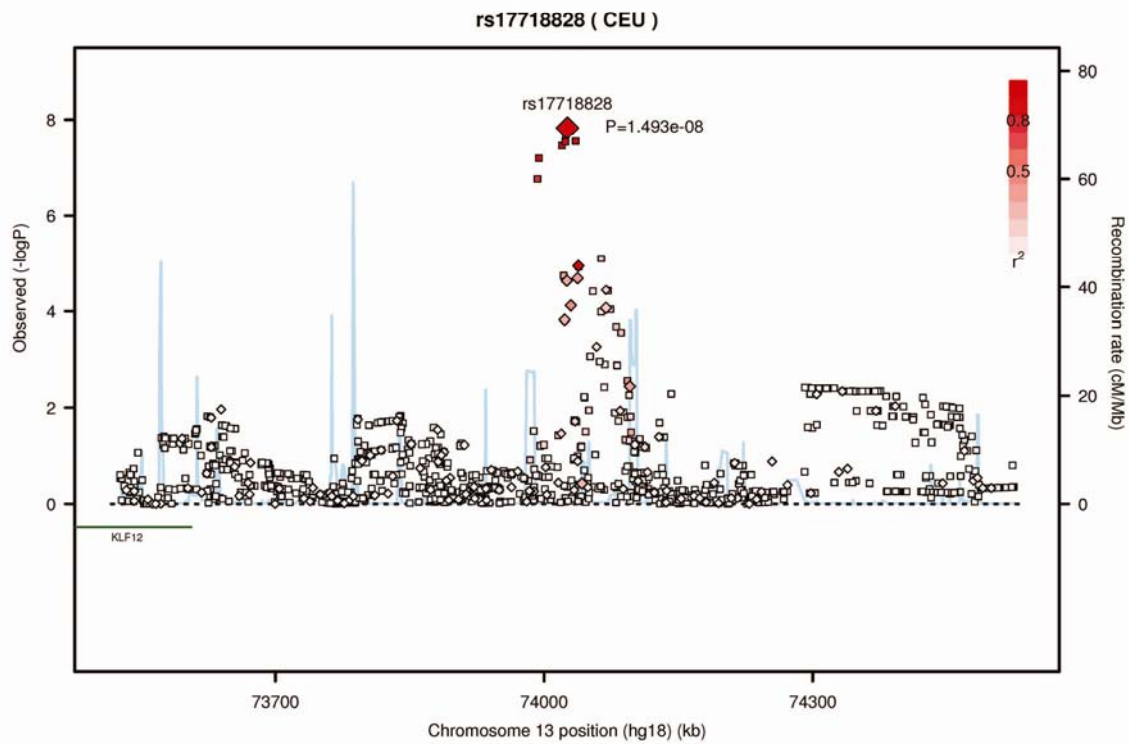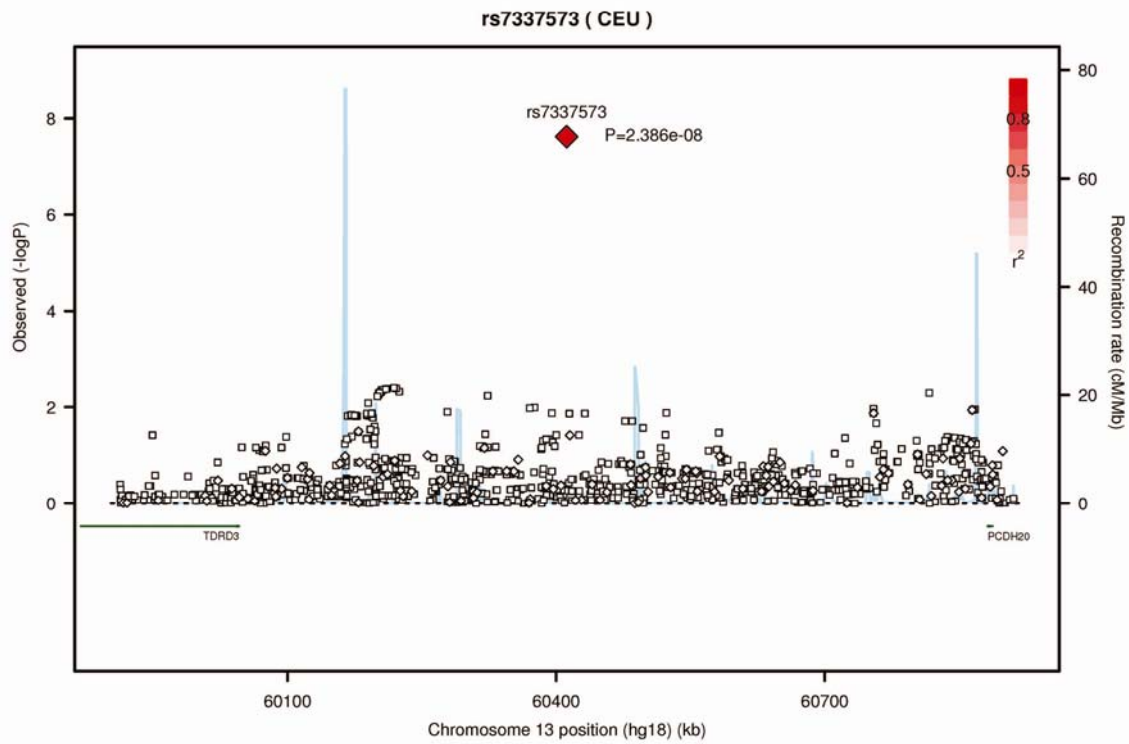

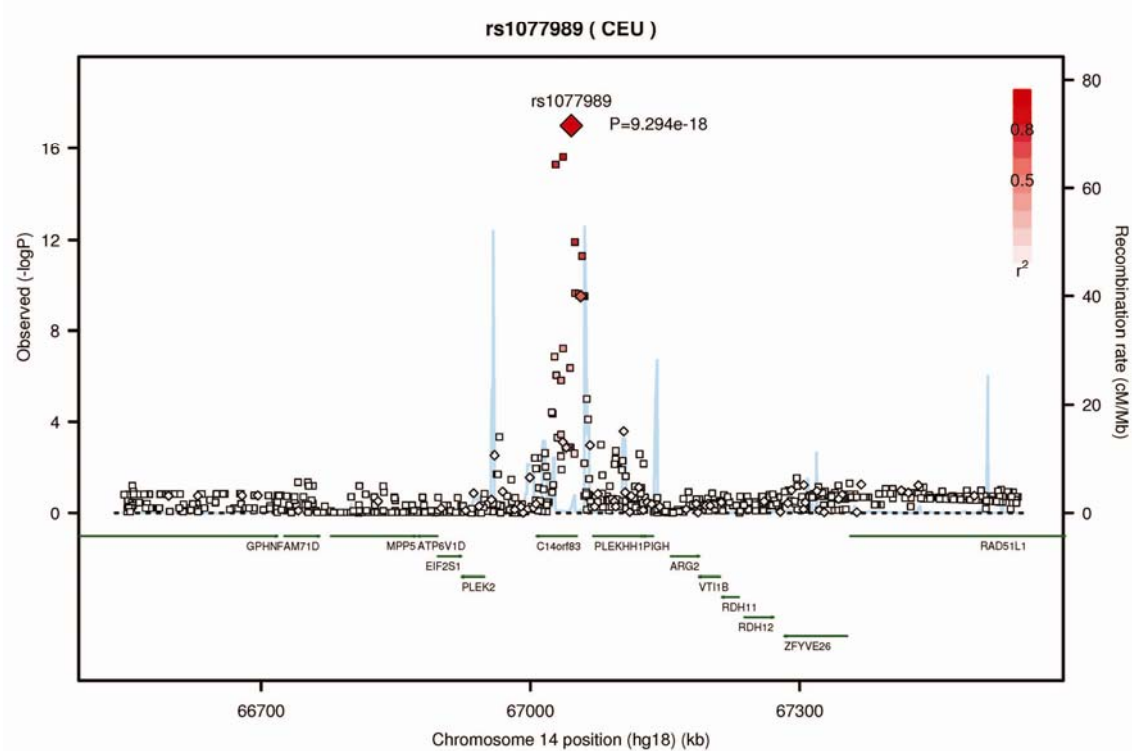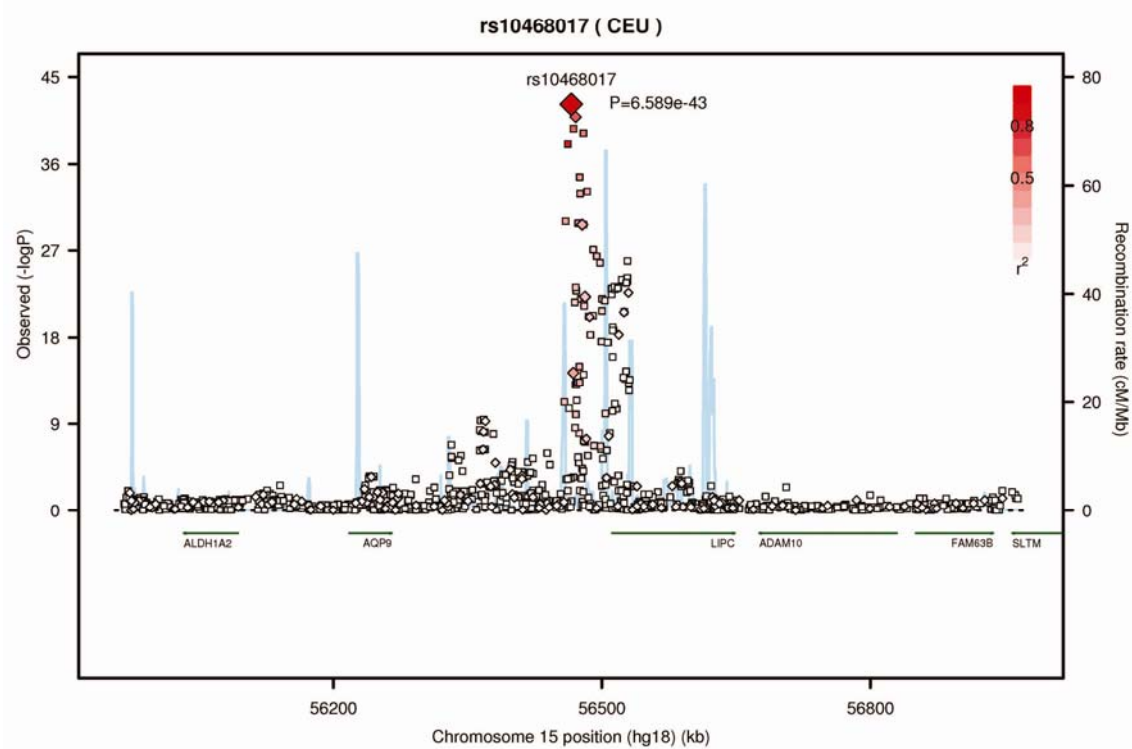

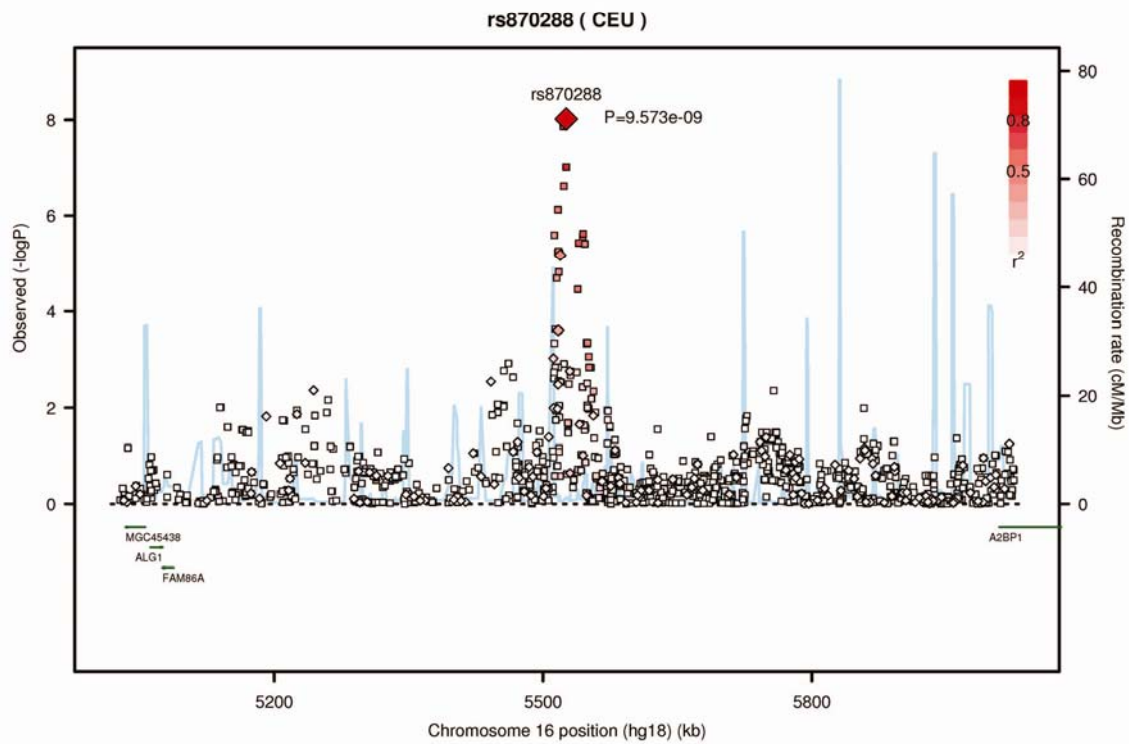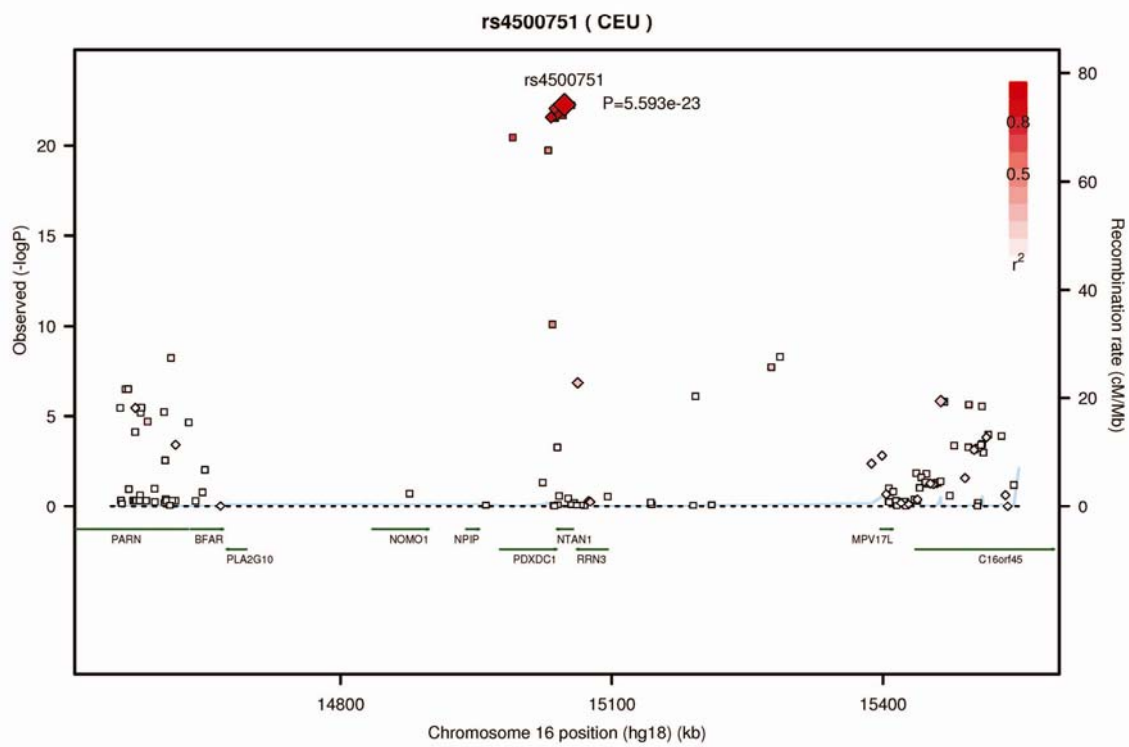

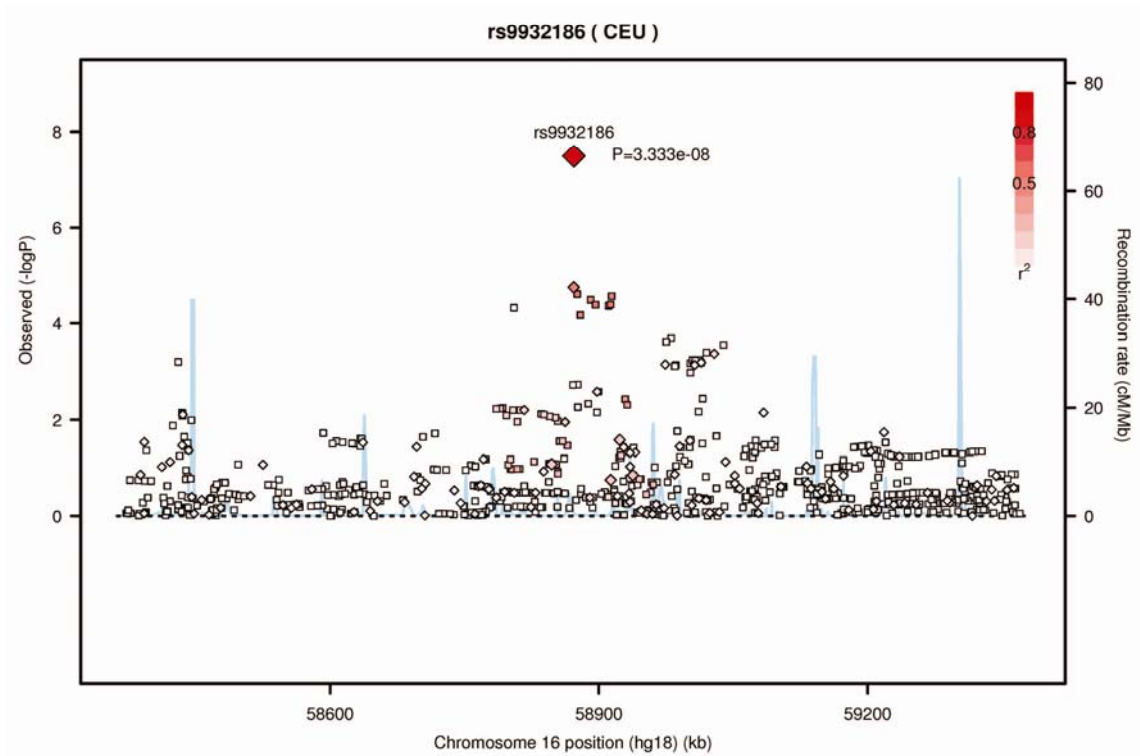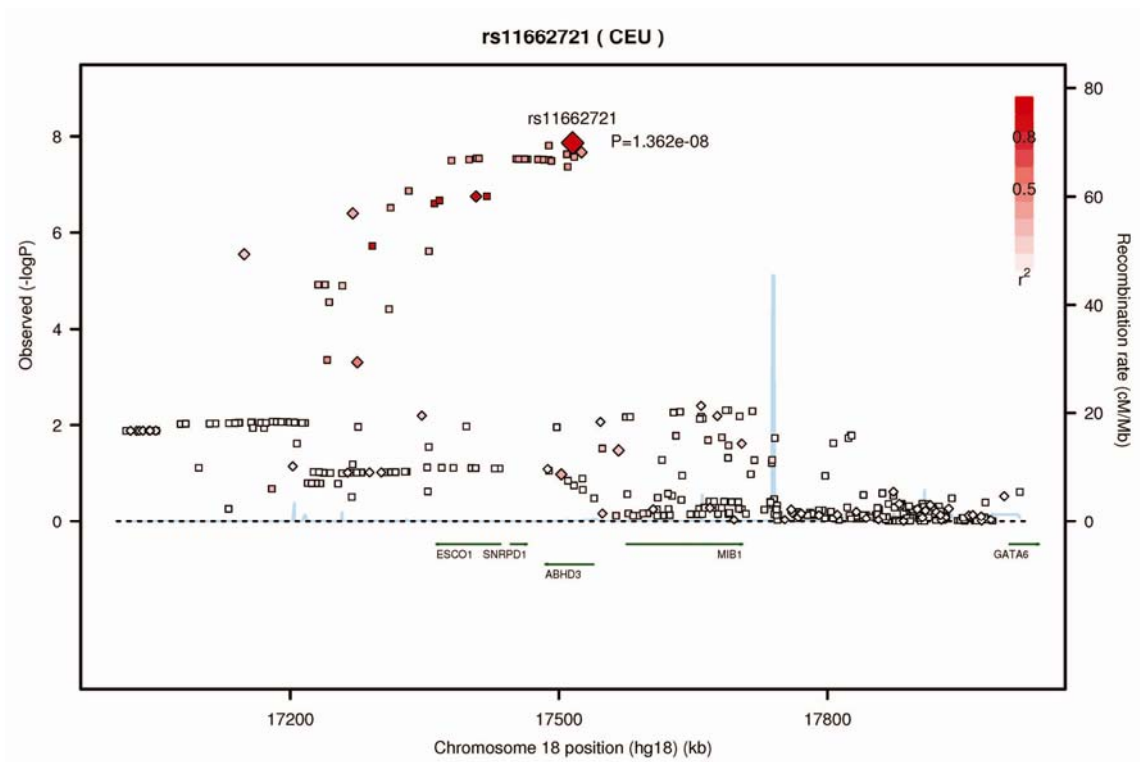

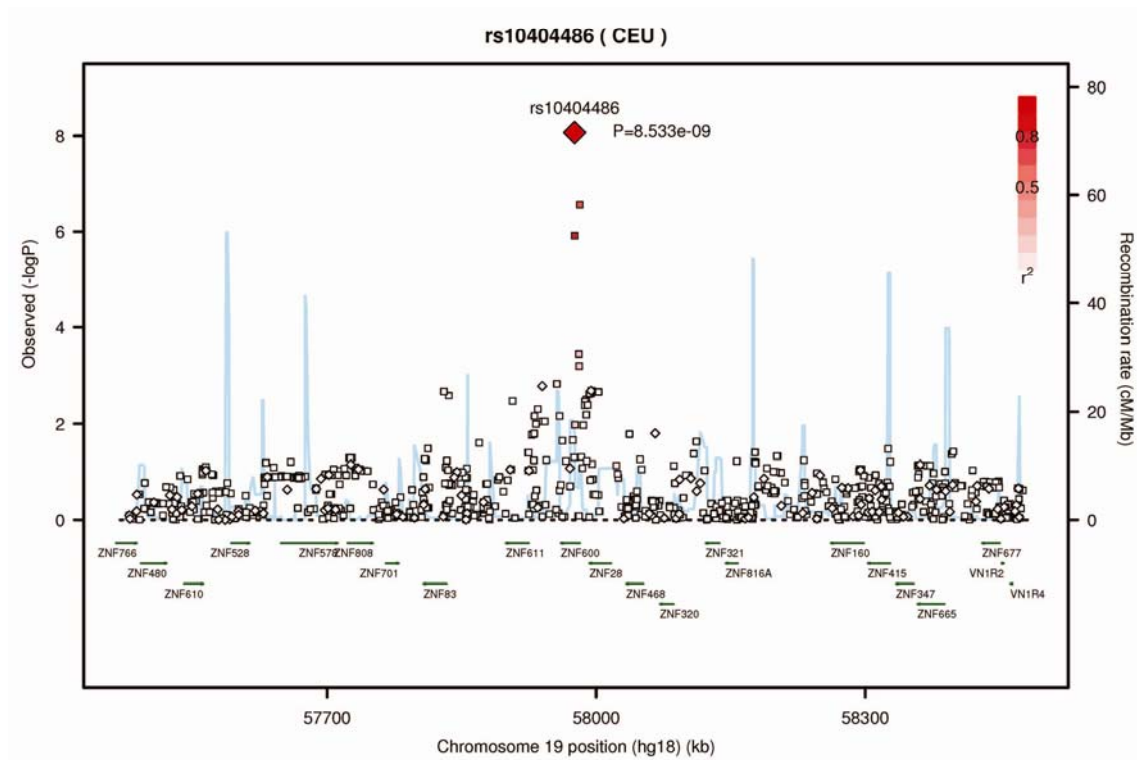

Supplement: Figure S2 — Regional plots of phospholipid related loci. Regional association plots covering a 1 Mb window around the top SNPs were created using Locus Zoom (https://statgen.sph.umich.edu/locuszoom). Diamonds denote the index SNPs (with the smallest P-value). The color scale on the right refers to the linkage disequilibrium (R2) between each SNP and the index SNP. Blue peaks show recombination rates. (PDF) [file pgen.1002490.s002.pdf]

Figure S3  
Regional association plots of sphingolipid associated loci

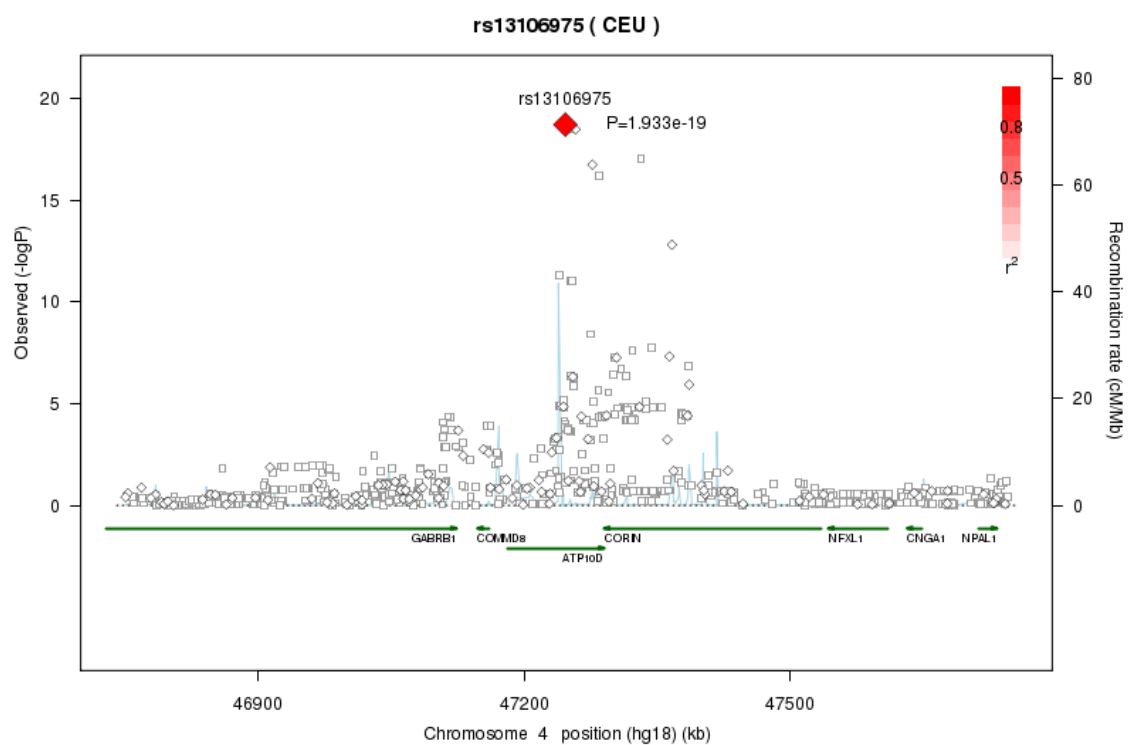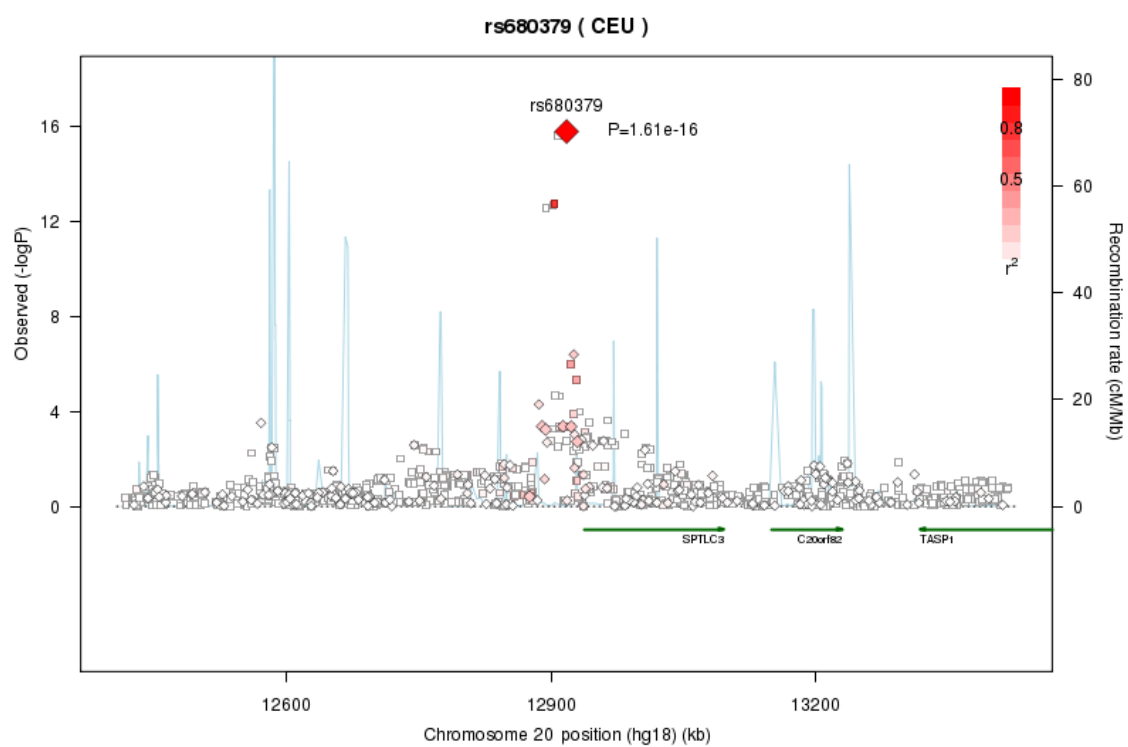

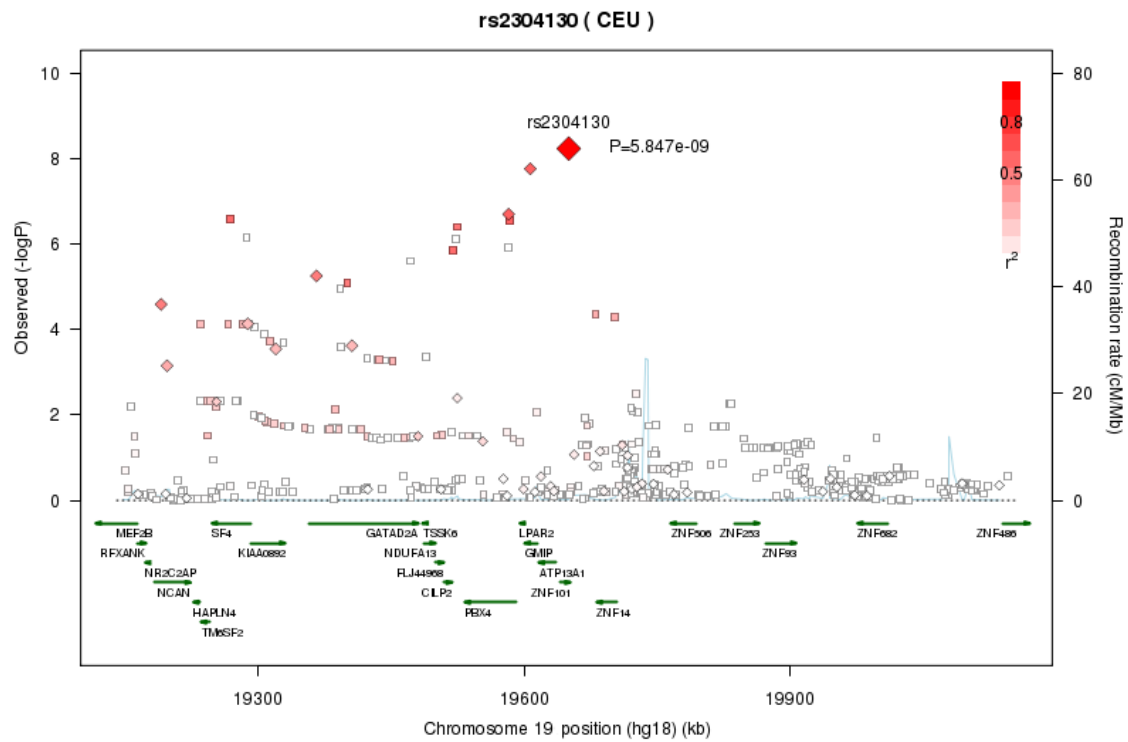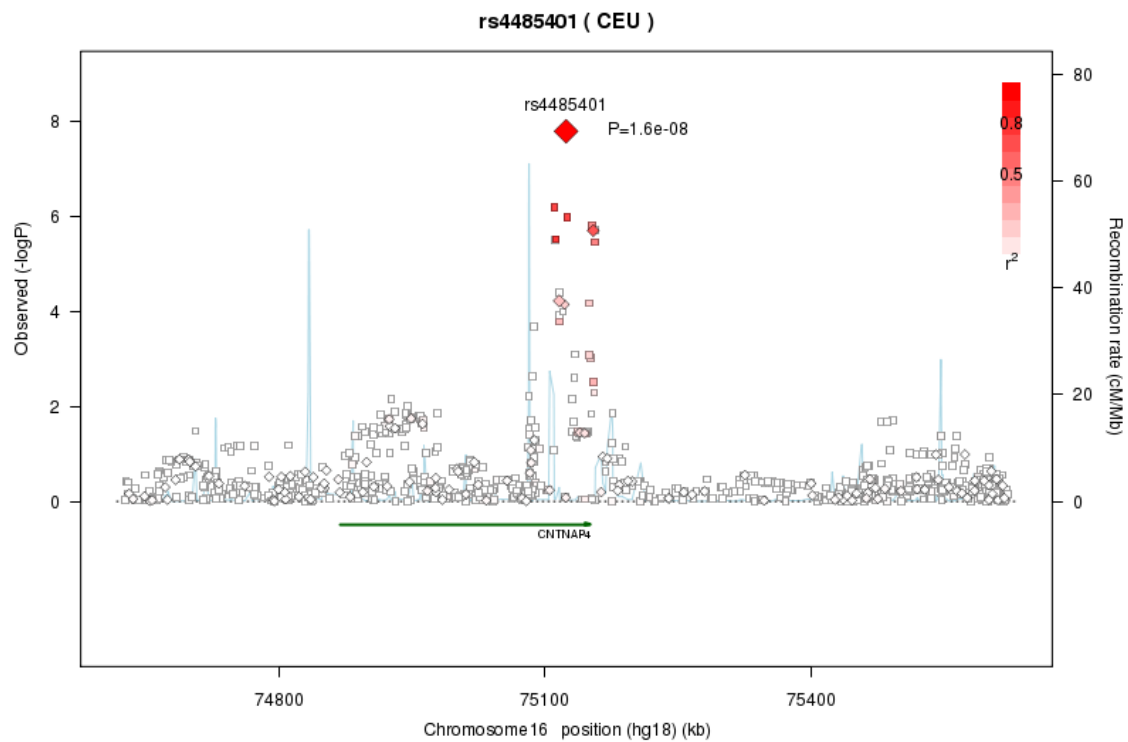

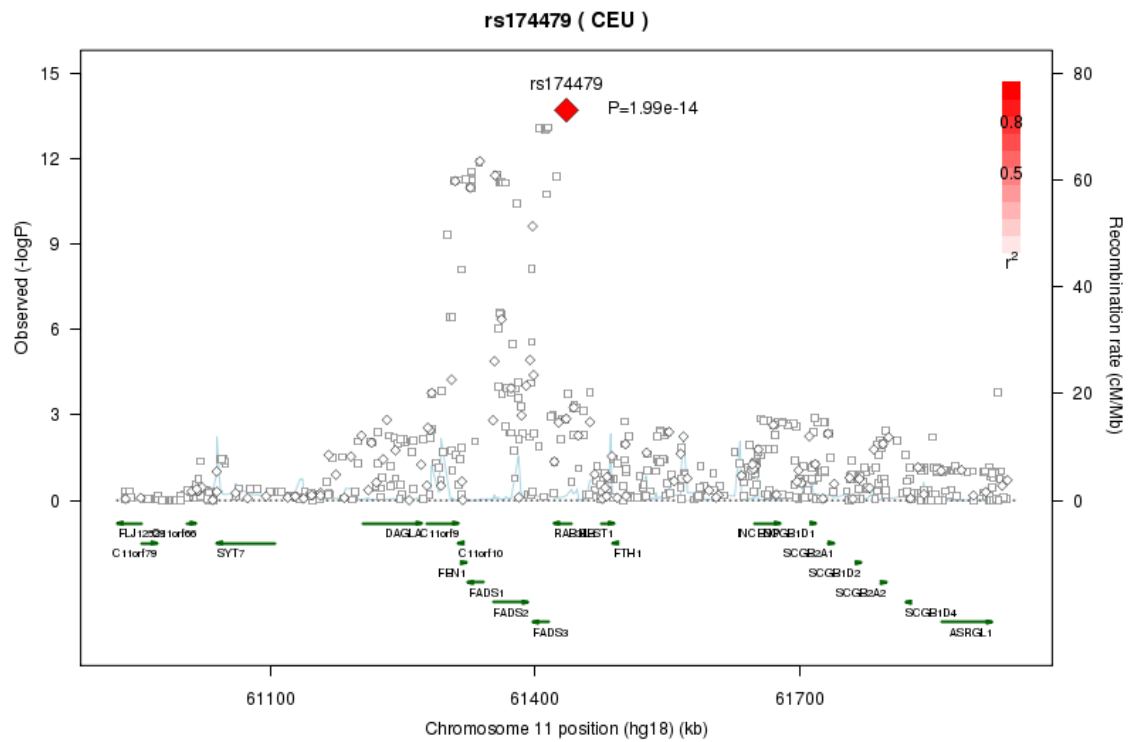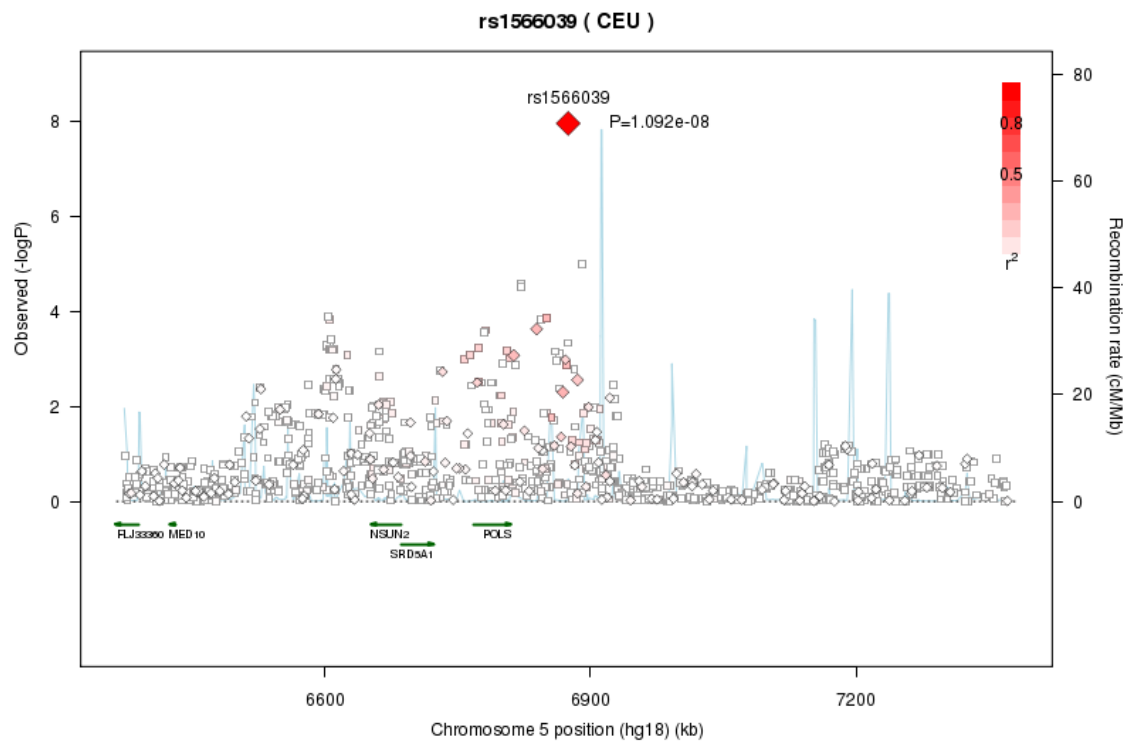

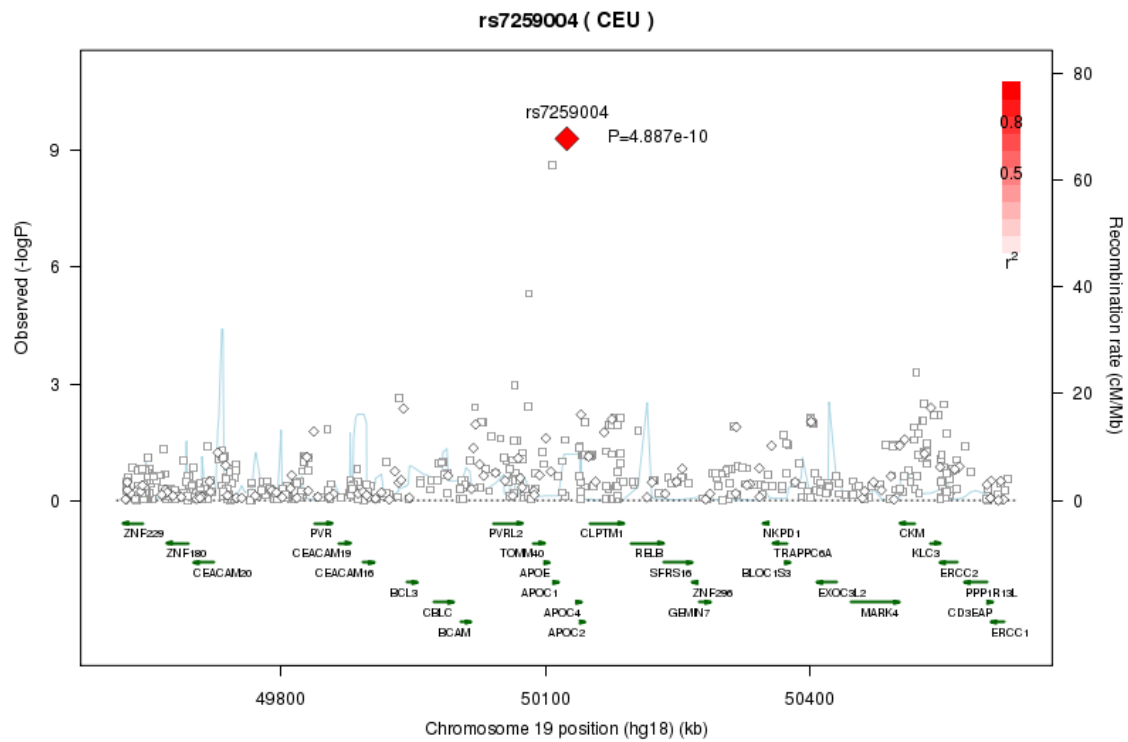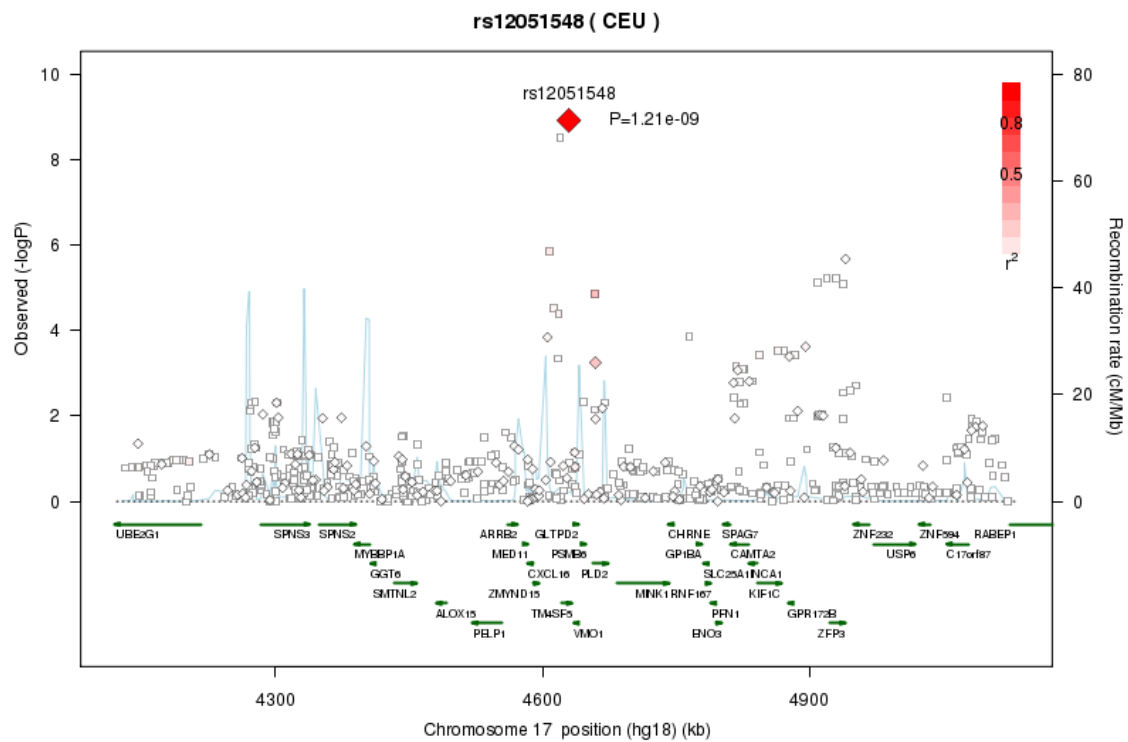

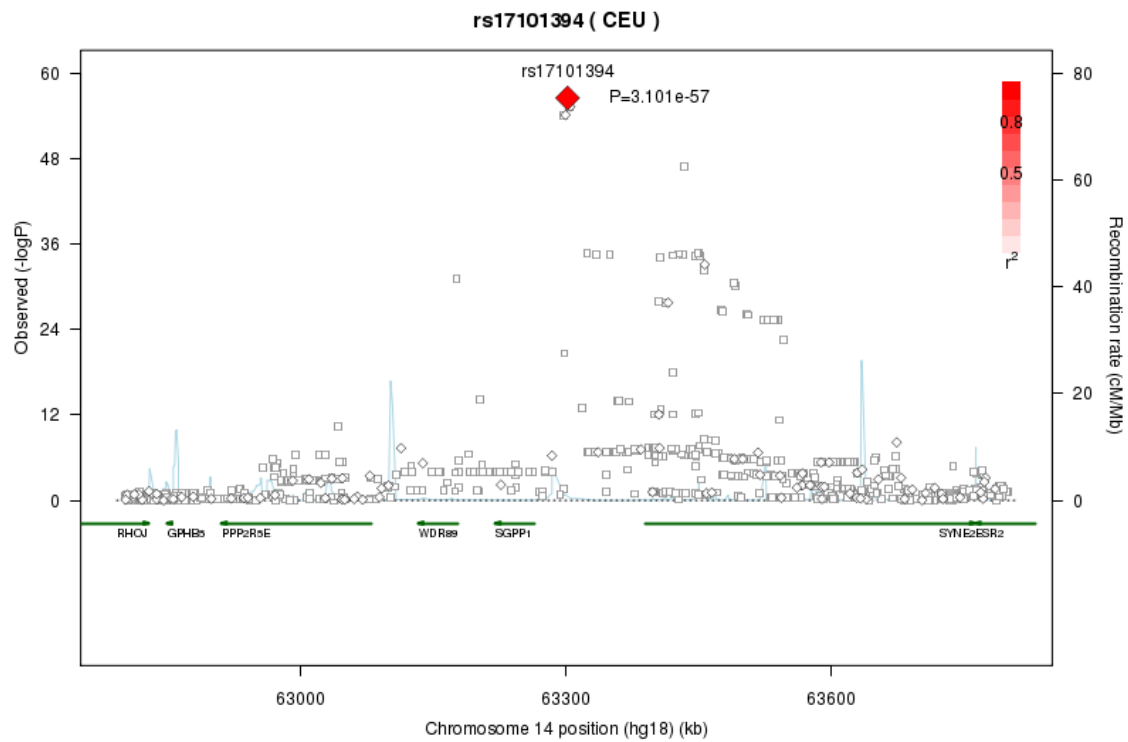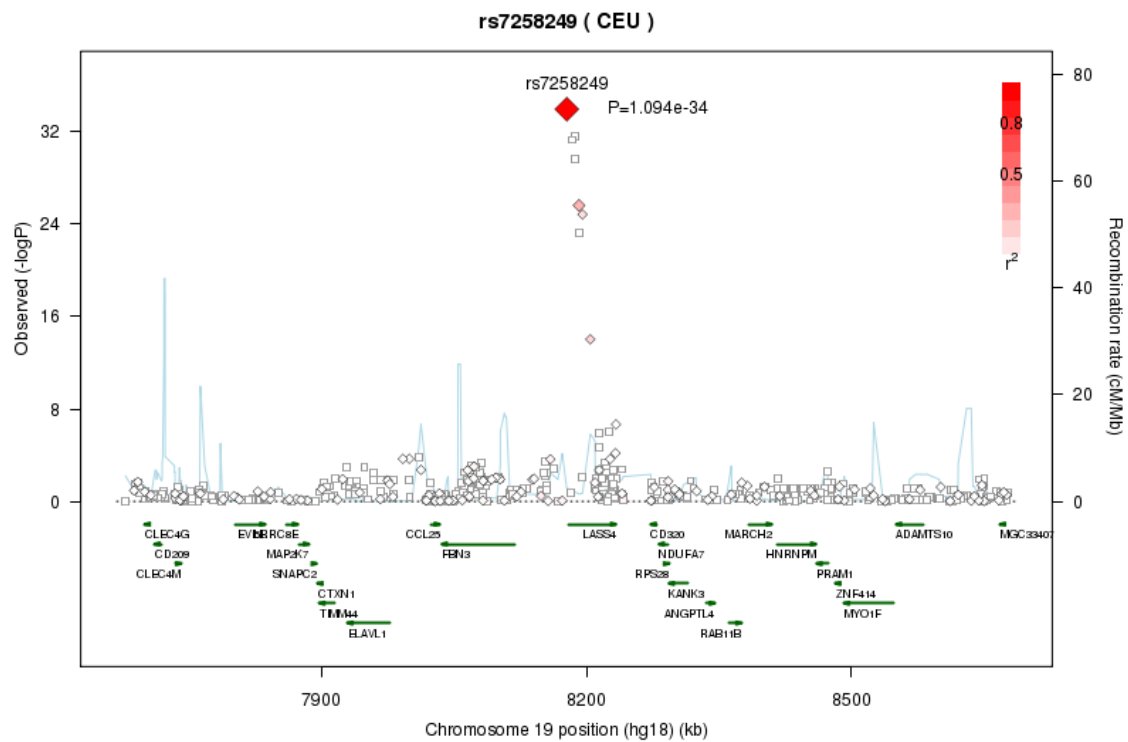

Supplement: Figure S3 — Regional plots of sphingolipid related loci. Regional association plots covering a 1 Mb window around the top SNPs were created using Locus Zoom (https://statgen.sph.umich.edu/locuszoom). Diamonds denote the index SNPs (with the smallest P-value). The color scale on the right refers to the linkage disequilibrium (R2) between each SNP and the index SNP. Blue peaks show recombination rates. (PDF) [file pgen.1002490.s003.pdf]

Lipid Pathways by Ingenuity, glycerophospholipid metabolism.

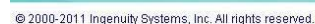

Supplement: Figure S4 — Lipid Pathways by Ingenuity (glycerophospholipid metabolism). Genes discovered in the GWAS are shown in orange. 1.Phosphatidylcholine-sterol O-acyltransferase has member mouse Lcat. 2. ApoA-I [APOA1] increases activation of LCAT. Binding of apoA-I [APOA1] and LCAT occurs. 3. Association of human APOA1 protein and human APOL1 protein occurs. 4. The affinity of binding of human APOL1 protein and cardiolipin in a system of purified components is greater than the affinity of binding of human APOL1 protein and phosphatidylinositol 3,5-bisphosphate in a system of purified components. 5. Binding of cardiolipin and human Matrilysin [MMP7] protein occurs in a cell-free system. 6. MMP7 protein increases C-terminal truncation cleavage of APOA1 protein. 7. In cell surface from RAW 264.7 cells, 8-bromo-cAMP increases binding of APOA1 protein and phosphatidylserine. 8. Phospholipase A1 catalyzes the following reaction: 1 phosphatidylserine+1 water−>1 2-Acyl-sn-glycero-3-phosphoserine+1 fatty acid. 9. Phosphatidylserine decarboxylase catalyzes the following reaction: 1 carbon dioxide+1 phosphatidylethanolamine−>1 phosphatidylserine. 10. Binding of phosphatidylserine and PKC ALPHA [PRKCA] protein occurs in a system of purified components. 11. Binding of human APOA1 protein and human GPI-PLD [GPLD1] protein occurs in human plasma. 12. Binding of apoA-I [APOA1] and phosphatidylcholine occurs. A molecular complex consisting of APOAI [APOA1] and of phosphatidylcholine increases secretion of phosphatidylcholine. 13. Lysophospholipase catalyzes the following reaction: 2 fatty acid+1 sn-glycero-3-phosphocholine−>1 phosphatidylcholine+2 water. 14.Human SPLA2 [PLA2G10] protein increases release of phosphatidylcholine to arachidonic acid. Mammalian group I sPLA2 [PLA2G10] increases hydrolysis of phosphatidylcholine. 15. In pulmonary surfactant, mouse sPLA2-X [Pla2g10] protein increases hydrolysis of phosphatidylglycerol. 16. Phospholipase A2 has member rat Lcat. 17. Rat Pla2 has member rat P [file pgen.1002490.s004.pdf]

Figure S5

Lipid Pathways by Ingenuity, sphingolipid metabolism.

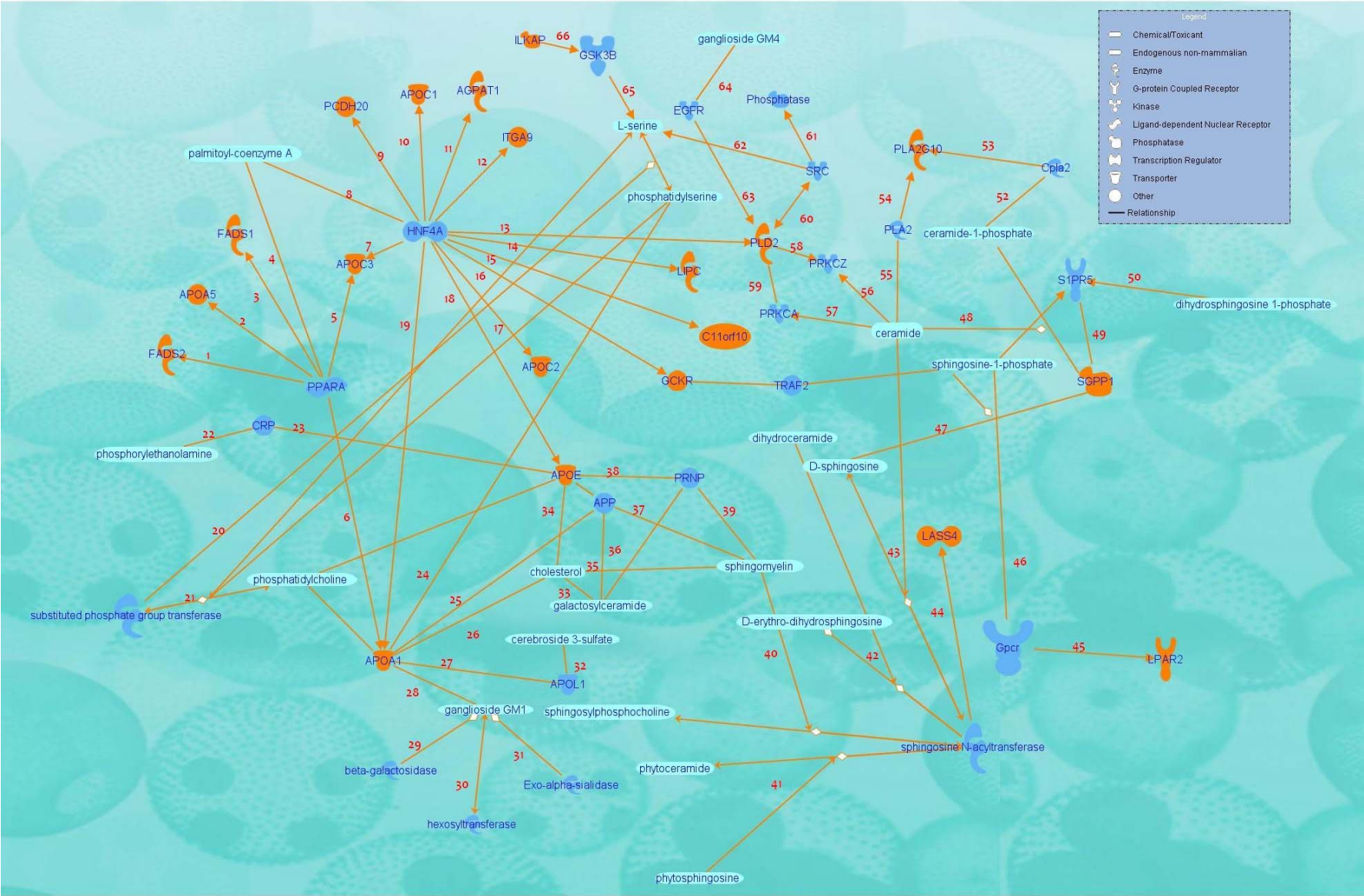

Supplement: Figure S5 — Lipid Pathways by Ingenuity (sphingolipid metabolism). Genes discovered in the GWAS are shown in orange. 1. In mouse liver, mouse PPAR-alpha [Ppara] protein is necessary for expression of mouse Delta 6 desaturase [Fads2] mRNA that is mediated by WY-14643. Binding of a DNA fragment (−385–373) containing a DR1 from human DELTA 6 DESATURASE [FADS2] gene and a protein-protein complex consisting of mouse PPAR-alpha [Ppara] and of rat Rxr alpha [Rxra] occurs in a cell free system. 2. In Hep3B cells expressing human APOAV [APOA5] protein, human Ppar alpha [PPARA] protein increases activation of promoter fragment (−617–18) from human APOAV [APOA5] gene. 3. In mouse liver, mouse PPAR-alpha [Ppara] protein is necessary for expression of mouse Fads1 mRNA that is mediated by WY-14643. 4. Binding of palmitoyl-CoA and mutant mouse Ppar alpha [Ppara] protein (N-terminal truncation 1–100 with its A/B domain deleted) occurs in a cell-free system. 5. PPARA protein decreases expression of APOC3 protein. Binding of promoter fragment (−96–61) from human APOC3 gene consisting of hormone response element and a protein-protein complex consisting of PPAR ALPHA [PPARA] and of RXR ALPHA [RXRA] occurs in a cell fraction from Cos-1 cells. PPAR ALPHA [PPARA] protein decreases transcription of APOC [APOC3] gene with a DNA endogenous promoter that has a PPAR response element. 6. PPAR ALPHA [PPARA] protein increases expression of human APOA1. 7. In HepG2 cells, mutant mouse HNF1-alpha [Hnf1a] protein (R131Q) causes little or no change in activation of APOC3 gene that is increased by human HNF4A2 protein. In HuH7 cells, rat Hnf4 alpha2 protein increases expression of human APOC3 mRNA. In a nuclear extract from Caco2 cells, the binding avidity of binding of promoter fragment from APOC3 gene consisting of HNF4 binding site and human HNF4A2 protein is greater than the binding avidity of binding of promoter fragment from FABP1 gene consisting of HNF4 binding site and human HNF4A2 protein. HNF4 protein i [file pgen.1002490.s005.pdf]
