# Supplementary material for: Genome-Wide Association Study Identifies Novel Loci Associated with Circulating Phospho- and Sphingolipid Concentrations
Source: PLoS Genet. 2012 Feb 16;8(2):e1002490. doi: 10.1371/journal.pgen.1002490 (PMC3280968; doi:10.1371/journal.pgen.1002490)

Figure S6

Q-Q plots of the association between the top loci and disease endpoints; IMT, CAD and type 2 diabetes.

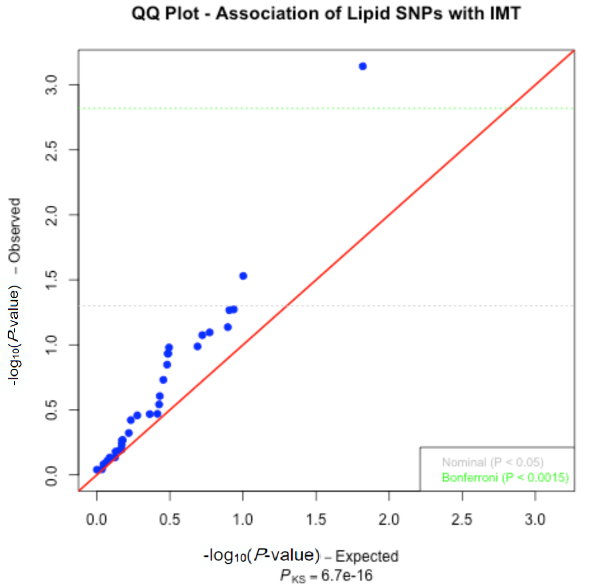

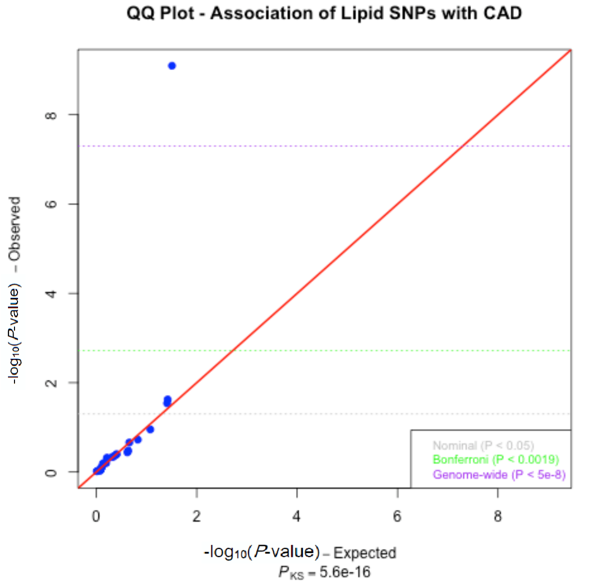

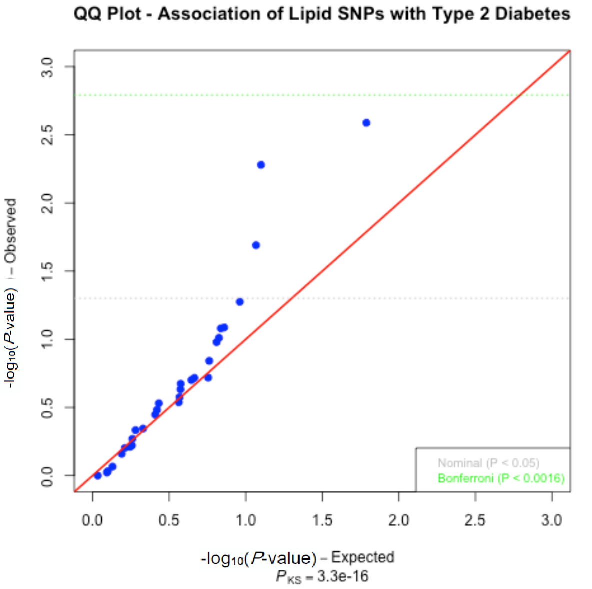

Supplement: Figure S6 — Q-Q plots for the association between the top loci and disease end points. P KS: P-value from a one sample Kolmogorov-Smirnov test comparing the observed P-value distribution to that expected under the null. (PDF) [file pgen.1002490.s006.pdf]
