# Supplementary material for: Genome-Wide Association Study Identifies Novel Loci Associated with Circulating Phospho- and Sphingolipid Concentrations
Source: PLoS Genet. 2012 Feb 16;8(2):e1002490. doi: 10.1371/journal.pgen.1002490 (PMC3280968; doi:10.1371/journal.pgen.1002490)
Supplement: Table S1 — Mean phospho- and sphingolipid concentrations of the study participants. SD: standard deviation; P-value: P-value for the test comparing means by gender. (PDF) [file pgen.1002490.s007.pdf]

Table S1

A. Mean levels and standard deviations (SD) of circulating phosphatidylcholines (PC) in men (1759) and women (2275)

	Absolute amounts of PC species [µM]					Proportions of PC species [% of total PC]				
	Men		Women			Men		Women		
PC- species	Mean	SD	Mean	SD	*P*-value	Mean	SD	Mean	SD	*P*-value
PC 26:0	0.420	0.429	0.416	0.434	7.72E-01	<0.001	<0.001	<0.001	<0.001	2.12E-01
PC 30:1	1.396	1.680	1.524	1.815	2.15E-02	0.001	0.001	0.001	0.001	3.33E-01
PC 30:0	4.838	2.516	5.280	2.782	1.82E-07	0.002	0.001	0.002	0.001	1.55E-02
PC O 32:1	2.855	0.907	3.083	0.988	5.93E-14	0.001	<0.001	0.001	<0.001	4.40E-02
PC O 32:0	3.654	1.160	4.066	1.274	8.21E-26	0.002	<0.001	0.002	<0.001	4.43E-08
PC 32:2	4.669	2.070	5.628	2.517	1.75E-38	0.002	0.001	0.002	0.001	3.00E-33
PC 32:1	19.572	12.510	20.559	10.926	9.59E-03	0.009	0.004	0.009	0.004	6.74E-01
PC 32:0	16.461	4.416	17.191	4.507	3.64E-07	0.008	0.001	0.008	0.001	2.12E-03
PC O 34:3	8.180	3.068	8.834	3.137	5.68E-11	0.004	0.001	0.004	0.001	2.99E-02
PC O 34:2	12.124	4.057	13.631	4.079	2.79E-30	0.006	0.001	0.006	0.001	3.36E-14
PC O 34:1	10.213	3.084	11.477	3.254	6.77E-35	0.005	0.001	0.005	0.001	7.42E-18
PC O 34:0	1.848	0.839	1.955	0.828	7.10E-05	0.001	<0.001	0.001	<0.001	9.94E-01
PC 34:4	1.865	0.909	2.067	0.957	1.71E-11	0.001	<0.001	0.001	<0.001	7.84E-07
PC 34:3	16.528	6.041	18.969	6.526	2.62E-33	0.008	0.002	0.008	0.002	2.55E-24
PC 34:2	522.212	130.552	566.750	139.528	1.45E-24	0.243	0.031	0.249	0.032	2.94E-09
PC 34:1	258.181	90.158	259.223	83.556	7.11E-01	0.118	0.024	0.113	0.022	1.23E-13
PC 34:0	4.678	3.317	4.566	3.163	2.86E-01	0.002	0.001	0.002	0.001	2.15E-04
PC O 36:5	12.930	4.863	11.964	3.917	2.14E-11	0.006	0.002	0.005	0.001	2.43E-41
PC O 36:4	18.213	6.300	17.519	4.956	1.76E-04	0.008	0.002	0.008	0.002	1.55E-26
PC O 36:3	7.938	2.452	8.723	2.394	1.56E-23	0.004	0.001	0.004	0.001	1.86E-08
PC O 36:2	14.843	4.857	16.954	5.026	1.35E-39	0.007	0.002	0.007	0.002	4.50E-23
PC O 36:1	9.613	3.312	10.368	3.224	8.62E-13	0.004	0.001	0.005	0.001	1.81E-02
PC O 36:0	1.060	0.522	1.102	0.518	1.26E-02	<0.001	<0.001	<0.001	<0.001	3.58E-01
PC 36:5	28.350	18.380	27.339	17.408	7.98E-02	0.013	0.008	0.012	0.007	2.91E-06
PC 36:4	209.772	67.612	215.995	65.029	3.61E-03	0.096	0.017	0.094	0.017	1.33E-04
PC 36:3	148.663	41.521	164.132	45.570	3.04E-28	0.069	0.009	0.072	0.009	2.78E-24
PC 36:2	279.314	73.646	297.449	73.464	2.16E-14	0.129	0.017	0.131	0.019	1.92E-02
PC 36:1	46.870	19.264	46.731	17.192	8.14E-01	0.021	0.006	0.020	0.005	4.60E-09
PC 36:0	3.533	1.989	3.545	2.000	8.45E-01	0.002	0.001	0.002	0.001	6.64E-03
PC O 38:5	17.301	4.681	17.183	4.199	4.11E-01	0.008	0.002	0.008	0.002	2.84E-17

PC O 38:4	12.554	3.855	13.134	3.645	1.68E-06	0.006	0.001	0.006	0.001	3.27E-01
PC O 38:3	4.532	1.441	5.161	1.609	2.37E-37	0.002	0.001	0.002	0.001	2.24E-16
PC O 38:2	2.288	1.022	2.524	1.131	8.31E-12	0.001	<0.001	0.001	<0.001	6.99E-04
PC O 38:1	1.142	0.822	1.233	0.894	1.02E-03	0.001	<0.001	0.001	<0.001	3.65E-01
PC 38:7	2.635	1.070	2.911	1.130	5.93E-15	0.001	<0.001	0.001	<0.001	1.19E-04
PC 38:6	97.510	35.674	103.511	35.544	1.67E-07	0.046	0.015	0.046	0.014	9.84E-01
PC 38:5	64.747	21.206	66.478	19.607	8.72E-03	0.030	0.006	0.029	0.006	5.98E-04
PC 38:4	126.366	43.447	130.748	40.326	1.22E-03	0.058	0.013	0.057	0.014	7.43E-02
PC 38:3	47.799	17.885	50.775	18.476	3.50E-07	0.022	0.005	0.022	0.006	4.34E-01
PC 38:2	6.493	3.276	7.071	3.384	6.64E-08	0.003	0.001	0.003	0.001	5.00E-03
PC 38:1	9.334	4.725	10.047	4.996	4.73E-06	0.004	0.002	0.004	0.002	1.65E-01
PC 38:0	7.225	2.944	7.446	2.870	1.80E-02	0.003	0.001	0.003	0.001	2.82E-02
PC O 40:6	4.482	1.400	4.863	1.510	3.55E-16	0.002	0.001	0.002	0.001	1.13E-02
PC O 40:5	3.359	0.984	3.528	1.000	1.05E-07	0.002	<0.001	0.002	<0.001	4.44E-01
PC O 40:4	2.271	0.719	2.448	0.756	8.27E-14	0.001	<0.001	0.001	<0.001	3.53E-02
PC 40:7	6.883	2.224	7.858	2.520	4.19E-37	0.003	0.001	0.003	0.001	1.50E-14
PC 40:6	32.534	13.532	33.844	13.473	2.55E-03	0.015	0.006	0.015	0.005	1.45E-01
PC 40:5	12.005	4.732	11.771	4.223	1.07E-01	0.005	0.001	0.005	0.001	3.34E-15
PC 40:4	4.493	1.939	4.412	1.663	1.68E-01	0.002	0.001	0.002	0.001	4.84E-12
PC 40:3	3.468	1.898	3.563	1.878	1.15E-01	0.002	0.001	0.002	0.001	1.22E-01
PC 40:2	2.535	1.306	2.810	1.454	4.82E-10	0.001	0.001	0.001	0.001	2.10E-03
PC 40:1	4.516	2.398	4.845	2.558	3.38E-05	0.002	0.001	0.002	0.001	2.50E-01
PC 40:0	1.597	0.750	1.662	0.754	7.30E-03	0.001	<0.001	0.001	0.001	8.83E-01
PC O 42:6	1.129	0.440	1.263	0.484	2.44E-19	0.001	<0.001	0.001	<0.001	6.96E-05
PC O 42:5	1.444	0.490	1.570	0.526	1.39E-14	0.001	<0.001	0.001	<0.001	4.98E-02
PC 42:5	1.580	0.810	1.678	0.863	2.36E-04	0.001	<0.001	0.001	0.001	5.13E-01
PC 42:4	0.945	0.587	0.962	0.588	3.66E-01	<0.001	<0.001	<0.001	<0.001	8.67E-02

B. Mean levels and standard deviations (SD) of circulating phosphatidylethanolamines (PE) in men (1759) and women (2275)

	Absolute amounts of PE species [µM]					Proportions of PE species [% of total PE]				
	Men		Women			Men		Women		
PE species	Mean	SD	Mean	SD	*P*-value	Mean	SD	Mean	SD	*P*-value
PE 32:2	0.109	0.051	0.122	0.056	1.49E-15	0.004	0.002	0.004	0.002	2.00E-01
PE 32:1	0.292	0.256	0.333	0.243	4.14E-07	0.009	0.003	0.009	0.003	5.93E-01
PE 32:0	0.128	0.067	0.130	0.066	4.47E-01	0.004	0.002	0.004	0.002	1.31E-10
PE 34:3	0.347	0.197	0.410	0.251	2.72E-18	0.011	0.003	0.011	0.003	2.73E-01
PE 34:2	4.911	2.879	5.914	3.761	4.39E-21	0.143	0.029	0.149	0.029	7.35E-09
PE 34:1	2.686	1.861	2.870	1.989	2.84E-03	0.077	0.017	0.072	0.018	5.11E-21
PE 34:0	0.141	0.095	0.134	0.085	8.20E-03	0.005	0.003	0.004	0.003	9.10E-13
PE 36:5	0.317	0.197	0.339	0.207	6.57E-04	0.010	0.004	0.009	0.003	3.97E-08
PE 36:4	2.494	1.305	2.915	1.633	3.26E-19	0.075	0.015	0.076	0.015	6.94E-02
PE 36:3	1.863	1.242	2.153	1.333	2.26E-12	0.054	0.015	0.055	0.014	2.05E-02
PE 36:2	6.484	4.161	6.950	3.968	3.90E-04	0.186	0.034	0.178	0.032	6.95E-13
PE 36:1	1.405	1.089	1.285	0.825	1.35E-04	0.040	0.013	0.033	0.010	5.30E-73
PE 38:6	2.613	1.856	3.492	2.481	2.50E-36	0.077	0.028	0.088	0.029	1.57E-33
PE 38:5	1.442	0.889	1.692	0.968	5.62E-17	0.042	0.010	0.044	0.009	1.02E-05
PE 38:4	4.433	2.486	4.975	2.510	1.68E-11	0.133	0.032	0.132	0.031	7.50E-01
PE 38:3	0.541	0.377	0.640	0.414	8.53E-15	0.016	0.005	0.016	0.004	7.00E-05
PE 38:2	0.160	0.094	0.173	0.098	4.41E-05	0.005	0.003	0.005	0.002	9.93E-04
PE 38:1	0.194	0.108	0.202	0.095	1.05E-02	0.007	0.003	0.006	0.003	2.09E-06
PE 40:6	1.783	1.204	2.182	1.411	2.03E-21	0.053	0.020	0.056	0.020	1.89E-07
PE 40:5	0.452	0.303	0.485	0.285	5.48E-04	0.013	0.004	0.013	0.003	5.42E-08
PE 40:4	0.163	0.105	0.167	0.094	1.61E-01	0.005	0.002	0.005	0.002	1.19E-15
PE 40:3	0.046	0.033	0.049	0.035	1.93E-03	0.002	0.001	0.001	0.001	3.98E-02
PE 42:7	0.048	0.023	0.054	0.028	7.90E-14	0.002	0.001	0.002	0.001	5.19E-02
PE 42:6	0.032	0.016	0.035	0.019	4.00E-11	0.001	0.001	0.001	0.001	1.61E-01
PE 42:5	0.027	0.017	0.030	0.019	8.20E-07	0.001	0.001	0.001	0.001	7.76E-02

C. Mean levels and standard deviations (SD) of circulating lysophosphatidylcholines (LPC) in men (1759) and women (2275)

	Absolute amounts of LPC species [µM]					Proportions of LPC species [% of total LPC]				
	Men		Women			Men		Women		
LPC species	Mean	SD	Mean	SD	*P*-value	Mean	SD	Mean	SD	*P*-value
LPC 15:0	1.732	0.881	1.773	0.885	1.50E-01	0.005	0.002	0.006	0.002	2.07E-40
LPC 16:1	3.223	1.396	3.078	1.171	5.23E-04	0.010	0.003	0.010	0.002	6.31E-17
LPC 16:0	175.786	69.311	159.44	58.06	4.86E-15	0.526	0.045	0.535	0.048	3.88E-10
LPC 18:3	0.786	0.367	0.649	0.303	4.92E-35	0.002	0.001	0.002	0.001	8.68E-09
LPC 18:2	40.402	15.086	33.411	12.671	4.74E-52	0.125	0.032	0.115	0.032	6.68E-21
LPC 18:1	28.368	10.128	24.601	8.495	1.37E-34	0.087	0.017	0.084	0.015	1.69E-08
LPC 18:0	62.171	26.288	56.984	22.80	9.14E-11	0.185	0.023	0.189	0.026	1.63E-09
LPC 20:5	1.266	1.099	0.994	0.861	5.54E-17	0.004	0.003	0.004	0.003	4.46E-08
LPC 20:4	10.068	5.362	8.431	4.439	3.50E-24	0.031	0.013	0.029	0.013	5.17E-05
LPC 20:3	3.172	1.280	2.663	1.051	8.56E-40	0.010	0.003	0.009	0.003	1.98E-10
LPC 20:0	0.427	0.153	0.406	0.135	9.92E-06	0.001	<0.001	0.001	<0.001	1.94E-09
LPC 22:6	2.433	1.337	2.162	1.285	1.60E-10	0.008	0.004	0.008	0.004	3.51E-01
LPC 22:5	0.859	0.527	0.728	0.484	1.38E-15	0.003	0.001	0.003	0.001	4.92E-03
LPC 22:4	0.488	0.370	0.451	0.334	1.43E-03	0.002	0.001	0.002	0.001	9.44E-02
LPC 22:0	0.715	0.471	0.717	0.389	9.06E-01	0.002	0.002	0.003	0.002	2.21E-07

D. Mean levels and standard deviations (SD) of circulating PE-plasmologens (PLPE) in men (1759) and women (2275)

	Absolute amounts of PLPE [µM]					Proportions of PLPE [% of total PLPE]				
	Men		Women			Men		Women		
PLPE species	Mean	SD	Mean	SD	*P*-value	Mean	SD	Mean	SD	*P*-value
PLPE 16:0/18:2	2.424	1.016	2.408	0.955	6.13E-01	0.044	0.011	0.045	0.011	6.23E-06
PLPE 16:0/18:1	0.908	0.34	0.896	0.298	2.53E-01	0.017	0.004	0.017	0.004	1.37E-05
PLPE 16:0/20:5	0.909	0.703	0.808	0.63	2.83E-06	0.016	0.009	0.015	0.009	5.97E-06
PLPE 16:0/20:4	6.795	3.059	6.021	2.469	1.83E-17	0.12	0.024	0.112	0.022	1.78E-27
PLPE 16:0/22:6	3.944	1.435	4.039	1.413	3.87E-02	0.072	0.018	0.077	0.018	6.33E-15
PLPE 16:0/22:5	1.838	0.874	1.671	0.672	5.83E-11	0.033	0.01	0.032	0.009	5.48E-05
PLPE 18:1/18:2	1.705	0.706	1.777	0.697	1.62E-03	0.031	0.008	0.034	0.009	1.80E-22
PLPE 18:1/18:1	0.76	0.284	0.759	0.266	9.03E-01	0.014	0.003	0.014	0.004	1.85E-08
PLPE 18:1/20:5	0.744	0.532	0.696	0.597	7.82E-03	0.013	0.007	0.013	0.007	1.73E-02
PLPE 18:1/20:4	5.218	2.363	4.829	1.966	3.94E-08	0.092	0.02	0.089	0.017	2.28E-05
PLPE 18:1/22:6	2.049	0.918	2.18	1.018	2.30E-05	0.038	0.013	0.041	0.012	1.16E-15
PLPE 18:0/18:2	3.935	1.701	3.961	1.618	6.33E-01	0.07	0.017	0.074	0.018	4.17E-11
PLPE 18:0/18:1	1.145	0.515	1.09	0.535	9.97E-04	0.021	0.005	0.02	0.005	9.24E-01
PLPE 18:0/20:5	1.691	1.601	1.516	1.35	2.64E-04	0.029	0.018	0.027	0.017	4.47E-04
PLPE 18:0/20:4	10.918	5.399	9.898	4.24	1.34E-10	0.19	0.033	0.182	0.032	4.41E-14
PLPE 18:0/22:6	3.729	1.692	3.828	1.708	7.17E-02	0.067	0.017	0.071	0.017	6.11E-13

E. Mean levels and standard deviations (SD) of circulating sphingomyelines (SPM) in men (1759) and women (2275)

	Absolute amounts of SPM [µM]					Proportions of SPM [% of total SPM]				
	Male		Female			Male		Female		
	Mean	SD	Mean	SD	*P*-value	Mean	SD	Mean	SD	*P*-value
SPM 14:0	12.579	4.094	14.126	4.698	7.26E-28	0.025	0.005	0.025	0.005	8.77E-01
SPM 15:0	7.342	2.605	8.379	2.964	6.13E-31	0.015	0.004	0.015	0.003	1.78E-02
SPM 16:1	20.375	4.709	24.186	5.918	1.45E-105	0.041	0.005	0.044	0.006	3.02E-46
SPM 16:0	137.144	30.381	149.993	32.84	3.07E-36	0.278	0.021	0.272	0.021	6.23E-19
SPM dih 16:0	6.068	2.329	6.775	2.485	7.12E-20	0.012	0.003	0.012	0.004	6.52E-01
SPM 17:0	4.706	1.673	5.345	1.841	1.82E-29	0.009	0.002	0.010	0.002	6.94E-02
SPM 18:2	0.943	0.416	1.310	0.615	3.26E-103	0.002	0.001	0.002	0.001	1.10E-54
SPM 18:1	13.576	3.622	16.622	4.661	1.47E-110	0.028	0.005	0.030	0.005	3.43E-57
SPM 18:0	26.513	6.970	29.677	7.690	1.28E-40	0.054	0.007	0.054	0.007	6.70E-01
SPM dih 18:0	1.561	1.120	1.662	1.152	5.81E-03	0.003	0.002	0.003	0.002	4.35E-02
SPM 20:1	6.759	3.756	9.155	4.210	1.07E-75	0.014	0.007	0.016	0.007	1.51E-36
SPM 20:0	18.896	8.459	20.584	8.842	1.27E-09	0.038	0.013	0.037	0.012	4.02E-02
SPM 22:2	3.621	3.323	4.111	3.486	7.13E-06	0.007	0.006	0.007	0.006	4.36E-01
SPM 22:1	29.937	7.724	35.254	8.936	9.43E-85	0.060	0.008	0.064	0.008	3.22E-37
SPM 22:0	36.634	10.847	39.051	10.88	5.14E-12	0.074	0.012	0.070	0.011	4.17E-17
SPM dih 22:0	2.303	2.009	2.225	1.957	2.23E-01	0.005	0.004	0.004	0.003	1.76E-06
SPM 23:1	14.370	4.082	17.461	4.885	5.73E-98	0.029	0.005	0.031	0.004	1.69E-60
SPM 23:0	16.816	5.265	18.659	5.453	1.82E-26	0.034	0.006	0.034	0.005	3.69E-01
SPM dih 23:0	0.785	0.575	0.864	0.632	4.11E-05	0.002	0.001	0.002	0.001	7.54E-01
SPM 24:3	3.803	2.132	4.781	2.420	2.30E-40	0.008	0.004	0.009	0.004	4.21E-14
SPM 24:2	32.952	8.129	39.410	9.897	2.73E-105	0.067	0.010	0.072	0.010	1.07E-40
SPM 24:1	74.513	17.742	80.506	18.71	2.18E-24	0.152	0.019	0.147	0.019	1.46E-16
SPM 24:0	25.581	7.398	25.662	7.011	7.26E-01	0.052	0.008	0.046	0.008	7.93E-83
SPM dih 24:0	0.915	0.831	0.967	0.841	5.48E-02	0.002	0.002	0.002	0.001	4.14E-02

F. Mean levels and standard deviations (SD) of circulating ceramides in men (1759) and women (2275)

	Absolute amounts of Ceramides [μM]					Proportions of Ceramides [% of total Ceramide]				
	Male		Female			Male		Female		
	Mean	SD	Mean	SD	*P*-value	Mean	SD	Mean	SD	*P*-value
CER 16:0	0.951	0.287	1.005	0.300	9.65E-09	0.111	0.027	0.119	0.030	1.63E-19
CER 18:0	0.216	0.065	0.225	0.069	9.10E-06	0.025	0.006	0.027	0.007	9.83E-14
CER 20:0	0.190	0.059	0.198	0.058	1.03E-05	0.022	0.006	0.023	0.006	5.46E-11
CER 22:0	1.171	0.342	1.166	0.352	6.98E-01	0.133	0.015	0.134	0.015	1.43E-02
CER 23:0	1.072	0.371	1.105	0.375	5.85E-03	0.121	0.019	0.126	0.018	2.48E-20
CER 24:1	1.635	0.570	1.585	0.561	5.73E-03	0.187	0.040	0.184	0.040	1.53E-02
CER 24:0	3.548	1.080	3.370	1.024	1.52E-07	0.402	0.040	0.387	0.038	1.30E-30
Glu-CER 16:0	0.462	0.146	0.493	0.158	4.58E-10	0.055	0.019	0.059	0.022	1.22E-12
Glu-CER 24:1	0.615	0.218	0.644	0.249	1.47E-04	0.073	0.031	0.078	0.037	2.86E-05
