# Supplementary material for: Genome-Wide Association Study Identifies Novel Loci Associated with Circulating Phospho- and Sphingolipid Concentrations
Source: PLoS Genet. 2012 Feb 16;8(2):e1002490. doi: 10.1371/journal.pgen.1002490 (PMC3280968; doi:10.1371/journal.pgen.1002490)
Supplement: Table S2 — Association findings on selected lipid-lipid ratios. Effect: regression coefficient; seEffect: standard error of the regression coefficient. (PDF) [file pgen.1002490.s008.pdf]

Table S2

Association findings on selected lipid-lipid ratios

Locus	Gene	SNP	Ratio			Process	Effect	seEffect	*P*-value
1p21.3	TMEM56	rs9437689	LPC15:0	/	LPC18:0	elongation	-0.0005	0.0002	8.8E-03
1p21.3	TMEM56	rs9437689	LPC18:0	/	LPC 18:2	saturation	0.0204	0.0113	7.1E-02
1p21.3	TMEM56	rs9437689	LPC18:0	/	LPC20:0	elongation	0.3991	0.7265	5.8E-01
1p21.3	TMEM56	rs9437689	LPC18:0	/	LPC22:0	elongation	1.3664	0.6594	3.8E-02
1p21.3	TMEM56	rs9437689	LPC18:0	/	LPC18:2	saturation	0.0204	0.0113	7.1E-02
1p21.3	TMEM56	rs9437689	LPC18:0	/	LPC18:3	saturation	1.5094	0.6640	2.3E-02
1p21.3	TMEM56	rs9437689	LPC18:0	/	LPC18:1	saturation	0.0354	0.0109	1.1E-03
1p21.3	TMEM56	rs9437689	LPC18:0	/	LPC 16:1	unclassified	0.3688	0.1227	2.6E-03
1p21.3	TMEM56	rs9437689	LPC18:0	/	LPC 22:4	unclassified	5.6493	1.6689	7.1E-04
1p21.3	TMEM56	rs9437689	LPC18:0	/	LPC 20:4	unclassified	0.1963	0.1464	3.1E-04
1p21.3	TMEM56	rs9437689	LPC18:0	/	LPC 20:3	unclassified	0.4878	0.1464	8.6E-04
1p21.3	TMEM56	rs9437689	LPC18:0	/	LPC 22:6	unclassified	0.2220	0.3619	3.6E-01
1p21.3	TMEM56	rs9437689	LPC18:0	/	LPC 22:5	unclassified	1.3394	0.6917	5.3E-02
1p21.3	TMEM56	rs9437689	LPC18:0	/	LPC 20:0	elongation	0.3991	0.7265	5.8E-01
1p21.3	TMEM56	rs9437689	LPC18:0	/	LPC 20:5	unclassified	-0.0018	0.6530	1.0E+00
1p21.3	TMEM56	rs9437689	LPC16:0	/	LPC18:0	elongation	-0.0578	0.0104	2.7E-08
2q24.3	KCNH7	rs1424760	PC O 36:5	/	PC O 38:5	elongation	-0.0152	0.0032	1.57E-06
2q24.3	KCNH7	rs1424760	PC O 36:5	/	PC O 40:5	elongation	-0.0984	0.0277	3.88E-04
2q24.3	KCNH7	rs1424760	PC O 36:5	/	PC O 42:5	elongation	-0.2898	0.0856	7.15E-04
2q24.3	KCNH7	rs1424760	PC O 36:5	/	PC 36:5	oxidation	-0.0132	0.0053	1.24E-02
2q24.3	KCNH7	rs1424760	PC O 36:5	/	PC O 36:4	saturation	-0.0120	0.0033	2.50E-04
2q24.3	KCNH7	rs1424760	PC O 36:5	/	PC O 36:3	saturation	-0.0474	0.0105	5.83E-06
2q24.3	KCNH7	rs1424760	PC O 36:5	/	PC O 36:2	saturation	-0.0297	0.0069	1.55E-05
2q24.3	KCNH7	rs1424760	PC O 36:5	/	PC O 36:1	saturation	-0.0461	0.0102	6.74E-06
2q24.3	KCNH7	rs1424760	PC O 36:5	/	PC O 36:0	saturation	-0.2855	0.2245	2.04E-01
2q24.3	KCNH7	rs1424760	PC O 36:5	/	PC 26:0	unclassified	-1.8165	0.5873	1.98E-03
2q24.3	KCNH7	rs1424760	PC O 36:5	/	PC 30:1	unclassified	0.5560	0.6367	3.83E-01
2q24.3	KCNH7	rs1424760	PC O 36:5	/	PC 30:0	unclassified	-0.0778	0.0385	4.31E-02
2q24.3	KCNH7	rs1424760	PC O 36:5	/	PC O 32:1	unclassified	-0.1182	0.0353	8.00E-04
2q24.3	KCNH7	rs1424760	PC O 36:5	/	PC O 32:0	unclassified	-0.1060	0.0299	3.95E-04
2q24.3	KCNH7	rs1424760	PC O 36:5	/	PC 32:2	unclassified	-0.0808	0.0401	4.41E-02
2q24.3	KCNH7	rs1424760	PC O 36:5	/	PC 32:1	unclassified	-0.0307	0.0097	1.57E-03
2q24.3	KCNH7	rs1424760	PC O 36:5	/	PC 32:0	unclassified	-0.0278	0.0053	1.75E-07
2q24.3	KCNH7	rs1424760	PC O 36:5	/	PC O 34:3	unclassified	-0.0244	0.0109	2.49E-02
2q24.3	KCNH7	rs1424760	PC O 36:5	/	PC O 34:2	unclassified	-0.0354	0.0075	2.08E-06
2q24.3	KCNH7	rs1424760	PC O 36:5	/	PC O 34:1	unclassified	-0.0455	0.0091	5.95E-07
2q24.3	KCNH7	rs1424760	PC O 36:5	/	PC O 34:0	unclassified	-0.2945	0.0735	6.12E-05
2q24.3	KCNH7	rs1424760	PC O 36:5	/	PC 34:4	unclassified	-0.2303	0.0815	4.73E-03

2q24.3	KCNH7	rs1424760	PC O 36:5 / PC 34:3	unclassified	-0.0287	0.0065	1.07E-05
2q24.3	KCNH7	rs1424760	PC O 36:5 / PC 34:2	unclassified	-0.0007	0.0002	4.27E-05
2q24.3	KCNH7	rs1424760	PC O 36:5 / PC 34:1	unclassified	-0.0019	0.0004	9.02E-06
2q24.3	KCNH7	rs1424760	PC O 36:5 / PC 34:0	unclassified	-0.0431	0.0514	4.02E-01
2q24.3	KCNH7	rs1424760	PC O 36:5 / PC 36:4	unclassified	-0.0016	0.0004	6.04E-05
2q24.3	KCNH7	rs1424760	PC O 36:5 / PC 36:3	unclassified	-0.0031	0.0007	1.76E-06
2q24.3	KCNH7	rs1424760	PC O 36:5 / PC 36:2	unclassified	-0.0016	0.0003	2.02E-06
2q24.3	KCNH7	rs1424760	PC O 36:5 / PC 36:1	unclassified	-0.0121	0.0025	1.45E-06
2q24.3	KCNH7	rs1424760	PC O 36:5 / PC 36:0	unclassified	-0.1107	0.0593	6.19E-02
2q24.3	KCNH7	rs1424760	PC O 36:5 / PC O 38:4	unclassified	-0.0228	0.0058	8.25E-05
2q24.3	KCNH7	rs1424760	PC O 36:5 / PC O 38:3	unclassified	-0.0865	0.0227	1.34E-04
2q24.3	KCNH7	rs1424760	PC O 36:5 / PC O 38:2	unclassified	-0.2815	0.1758	1.09E-01
2q24.3	KCNH7	rs1424760	PC O 36:5 / PC O 38:1	unclassified	-0.2596	0.4790	5.88E-01
2q24.3	KCNH7	rs1424760	PC O 36:5 / PC 38:7	unclassified	-0.1862	0.0505	2.30E-04
2q24.3	KCNH7	rs1424760	PC O 36:5 / PC 38:6	unclassified	-0.0049	0.0011	1.33E-05
2q24.3	KCNH7	rs1424760	PC O 36:5 / PC 38:5	unclassified	-0.0056	0.0013	1.21E-05
2q24.3	KCNH7	rs1424760	PC O 36:5 / PC 38:4	unclassified	-0.0024	0.0007	3.90E-04
2q24.3	KCNH7	rs1424760	PC O 36:5 / PC 38:3	unclassified	-0.0101	0.0027	1.68E-04
2q24.3	KCNH7	rs1424760	PC O 36:5 / PC 38:2	unclassified	-0.1124	0.0833	1.77E-01
2q24.3	KCNH7	rs1424760	PC O 36:5 / PC 38:1	unclassified	-0.0327	0.0253	1.96E-01
2q24.3	KCNH7	rs1424760	PC O 36:5 / PC 38:0	unclassified	-0.0575	0.0181	1.47E-03
2q24.3	KCNH7	rs1424760	PC O 36:5 / PC O 40:6	unclassified	-0.1043	0.0209	5.84E-07
2q24.3	KCNH7	rs1424760	PC O 36:5 / PC O 40:4	unclassified	-0.1146	0.0433	8.21E-03
2q24.3	KCNH7	rs1424760	PC O 36:5 / PC 40:7	unclassified	-0.0719	0.0152	2.16E-06
2q24.3	KCNH7	rs1424760	PC O 36:5 / PC 40:6	unclassified	-0.0164	0.0039	3.08E-05
2q24.3	KCNH7	rs1424760	PC O 36:5 / PC 40:5	unclassified	-0.0326	0.0095	6.00E-04
2q24.3	KCNH7	rs1424760	PC O 36:5 / PC 40:4	unclassified	-0.0437	0.0267	1.01E-01
2q24.3	KCNH7	rs1424760	PC O 36:5 / PC 40:3	unclassified	-0.0407	0.0630	5.18E-01
2q24.3	KCNH7	rs1424760	PC O 36:5 / PC 40:2	unclassified	-0.1520	0.0900	9.12E-02
2q24.3	KCNH7	rs1424760	PC O 36:5 / PC 40:1	unclassified	-0.0439	0.0545	4.20E-01
2q24.3	KCNH7	rs1424760	PC O 36:5 / PC 40:0	unclassified	-0.3735	0.1139	1.05E-03
2q24.3	KCNH7	rs1424760	PC O 36:5 / PC O 42:6	unclassified	-0.3138	0.1159	6.80E-03
2q24.3	KCNH7	rs1424760	PC O 36:5 / PC 42:5	unclassified	-0.1456	0.1366	2.87E-01
2q24.3	KCNH7	rs1424760	PC O 36:5 / PC 42:4	unclassified	-0.3689	0.3032	2.24E-01
2q37.3	ILKAP	rs12472274	PC 40:3 / PC 34:3	elongation	0.0122	0.0029	2.63E-05
2q37.3	ILKAP	rs12472274	PC 40:3 / PC 36:3	elongation	0.0015	0.0003	2.91E-07
2q37.3	ILKAP	rs12472274	PC 40:3 / PC 38:3	elongation	0.0057	0.0011	7.62E-08
2q37.3	ILKAP	rs12472274	PC 40:3 / PC 40:7	saturation	0.0276	0.0066	2.65E-05
2q37.3	ILKAP	rs12472274	PC 40:3 / PC 40:6	saturation	0.0070	0.0016	1.17E-05
2q37.3	ILKAP	rs12472274	PC 40:3 / PC 40:5	saturation	0.0233	0.0042	4.02E-08
2q37.3	ILKAP	rs12472274	PC 40:3 / PC 40:4	saturation	0.0571	0.0103	3.39E-08

2q37.3	ILKAP	rs12472274	PC 40:3 / PC 40:2	saturation	0.0373	0.0139	7.26E-03
2q37.3	ILKAP	rs12472274	PC 40:3 / PC 40:1	saturation	0.0236	0.0086	6.25E-03
2q37.3	ILKAP	rs12472274	PC 40:3 / PC 40:0	saturation	0.0672	0.0333	4.39E-02
2q37.3	ILKAP	rs12472274	PC 40:3 / PC 26:0	unclassified	0.4345	0.1966	2.71E-02
2q37.3	ILKAP	rs12472274	PC 40:3 / PC 30:1	unclassified	0.5413	0.2188	1.34E-02
2q37.3	ILKAP	rs12472274	PC 40:3 / PC 30:0	unclassified	0.0363	0.0136	7.67E-03
2q37.3	ILKAP	rs12472274	PC 40:3 / PC O 32:1	unclassified	0.0700	0.0185	1.52E-04
2q37.3	ILKAP	rs12472274	PC 40:3 / PC O 32:0	unclassified	0.0663	0.0138	1.53E-06
2q37.3	ILKAP	rs12472274	PC 40:3 / PC 32:2	unclassified	0.0568	0.0156	2.59E-04
2q37.3	ILKAP	rs12472274	PC 40:3 / PC 32:1	unclassified	0.0143	0.0036	6.27E-05
2q37.3	ILKAP	rs12472274	PC 40:3 / PC 32:0	unclassified	0.0128	0.0026	1.14E-06
2q37.3	ILKAP	rs12472274	PC 40:3 / PC O 34:3	unclassified	0.0241	0.0071	7.34E-04
2q37.3	ILKAP	rs12472274	PC 40:3 / PC O 34:2	unclassified	0.0192	0.0042	5.03E-06
2q37.3	ILKAP	rs12472274	PC 40:3 / PC O 34:1	unclassified	0.0216	0.0044	1.23E-06
2q37.3	ILKAP	rs12472274	PC 40:3 / PC O 34:0	unclassified	0.1044	0.0312	8.34E-04
2q37.3	ILKAP	rs12472274	PC 40:3 / PC 34:4	unclassified	0.1003	0.0323	1.91E-03
2q37.3	ILKAP	rs12472274	PC 40:3 / PC 34:2	unclassified	0.0004	0.0001	1.51E-06
2q37.3	ILKAP	rs12472274	PC 40:3 / PC 34:1	unclassified	0.0009	0.0002	9.97E-07
2q37.3	ILKAP	rs12472274	PC 40:3 / PC 34:0	unclassified	0.0427	0.0142	2.57E-03
2q37.3	ILKAP	rs12472274	PC 40:3 / PC O 36:5	unclassified	0.0160	0.0037	1.48E-05
2q37.3	ILKAP	rs12472274	PC 40:3 / PC O 36:4	unclassified	0.0126	0.0025	4.64E-07
2q37.3	ILKAP	rs12472274	PC 40:3 / PC O 36:3	unclassified	0.0267	0.0061	1.19E-05
2q37.3	ILKAP	rs12472274	PC 40:3 / PC O 36:2	unclassified	0.0152	0.0034	6.21E-06
2q37.3	ILKAP	rs12472274	PC 40:3 / PC O 36:1	unclassified	0.0231	0.0050	3.11E-06
2q37.3	ILKAP	rs12472274	PC 40:3 / PC O 36:0	unclassified	0.1608	0.0692	2.01E-02
2q37.3	ILKAP	rs12472274	PC 40:3 / PC 36:5	unclassified	0.0075	0.0022	7.11E-04
2q37.3	ILKAP	rs12472274	PC 40:3 / PC 36:4	unclassified	0.0009	0.0002	5.39E-07
2q37.3	ILKAP	rs12472274	PC 40:3 / PC 36:2	unclassified	0.0008	0.0002	3.66E-06
2q37.3	ILKAP	rs12472274	PC 40:3 / PC 36:1	unclassified	0.0052	0.0011	4.28E-06
2q37.3	ILKAP	rs12472274	PC 40:3 / PC 36:0	unclassified	0.0306	0.0175	8.11E-02
2q37.3	ILKAP	rs12472274	PC 40:3 / PC O 38:5	unclassified	0.0117	0.0025	2.34E-06
2q37.3	ILKAP	rs12472274	PC 40:3 / PC O 38:4	unclassified	0.0162	0.0033	1.03E-06
2q37.3	ILKAP	rs12472274	PC 40:3 / PC O 38:3	unclassified	0.0445	0.0103	1.53E-05
2q37.3	ILKAP	rs12472274	PC 40:3 / PC O 38:2	unclassified	0.1096	0.0527	3.75E-02
2q37.3	ILKAP	rs12472274	PC 40:3 / PC O 38:1	unclassified	0.2081	0.1508	1.68E-01
2q37.3	ILKAP	rs12472274	PC 40:3 / PC 38:7	unclassified	0.0455	0.0202	2.47E-02
2q37.3	ILKAP	rs12472274	PC 40:3 / PC 38:6	unclassified	0.0020	0.0005	3.01E-05
2q37.3	ILKAP	rs12472274	PC 40:3 / PC 38:5	unclassified	0.0032	0.0006	9.84E-07
2q37.3	ILKAP	rs12472274	PC 40:3 / PC 38:4	unclassified	0.0017	0.0003	1.90E-07
2q37.3	ILKAP	rs12472274	PC 40:3 / PC 38:2	unclassified	0.0348	0.0248	1.61E-01
2q37.3	ILKAP	rs12472274	PC 40:3 / PC 38:1	unclassified	0.0099	0.0042	1.80E-02

2q37.3	ILKAP	rs12472274	PC 40:3	/	PC 38:0	unclassified	0.0172	0.0045	1.59E-04
2q37.3	ILKAP	rs12472274	PC 40:3	/	PC O 40:6	unclassified	0.0502	0.0101	7.47E-07
2q37.3	ILKAP	rs12472274	PC 40:3	/	PC O 40:5	unclassified	0.0677	0.0139	1.15E-06
2q37.3	ILKAP	rs12472274	PC 40:3	/	PC O 40:4	unclassified	0.0863	0.0196	1.04E-05
2q37.3	ILKAP	rs12472274	PC 40:3	/	PC O 42:6	unclassified	0.2321	0.0473	9.37E-07
2q37.3	ILKAP	rs12472274	PC 40:3	/	PC O 42:5	unclassified	0.1554	0.0367	2.25E-05
2q37.3	ILKAP	rs12472274	PC 40:3	/	PC 42:5	unclassified	0.0751	0.0279	7.15E-03
2q37.3	ILKAP	rs12472274	PC 40:3	/	PC 42:4	unclassified	0.0179	0.0638	7.79E-01
3p22.2	ITGA9	rs197770	PLPE 18:0/22:6	/	PLPE 16:0/18:2	unclassified	-0.0659	0.0211	1.80E-03
3p22.2	ITGA9	rs197770	PLPE 18:0/22:6	/	PLPE 16:0/18:1	unclassified	-0.2058	0.0510	5.45E-05
3p22.2	ITGA9	rs197770	PLPE 18:0/22:6	/	PLPE 16:0/20:5	unclassified	0.0014	0.0712	9.84E-01
3p22.2	ITGA9	rs197770	PLPE 18:0/22:6	/	PLPE 16:0/20:4	unclassified	-0.0113	0.0068	9.90E-02
3p22.2	ITGA9	rs197770	PLPE 18:0/22:6	/	PLPE 16:0/22:6	unclassified	-0.0141	0.0065	3.06E-02
3p22.2	ITGA9	rs197770	PLPE 18:0/22:6	/	PLPE 16:0/22:5	unclassified	-0.0823	0.0283	3.71E-03
3p22.2	ITGA9	rs197770	PLPE 18:0/22:6	/	PLPE 18:1/18:2	unclassified	-0.0890	0.0288	2.02E-03
3p22.2	ITGA9	rs197770	PLPE 18:0/22:6	/	PLPE 18:1/18:1	unclassified	-0.1683	0.0606	5.45E-03
3p22.2	ITGA9	rs197770	PLPE 18:0/22:6	/	PLPE 18:1/20:5	unclassified	0.0397	0.0789	6.15E-01
3p22.2	ITGA9	rs197770	PLPE 18:0/22:6	/	PLPE 18:1/20:4	unclassified	-0.0095	0.0087	2.74E-01
3p22.2	ITGA9	rs197770	PLPE 18:0/22:6	/	PLPE 18:1/22:6	unclassified	-0.0067	0.0117	5.64E-01
3p22.2	ITGA9	rs197770	PLPE 18:0/22:6	/	PLPE 18:0/18:2	unclassified	-0.0369	0.0123	2.75E-03
3p22.2	ITGA9	rs197770	PLPE 18:0/22:6	/	PLPE 18:0/18:1	unclassified	-0.1218	0.0390	1.80E-03
3p22.2	ITGA9	rs197770	PLPE 18:0/22:6	/	PLPE 18:0/20:5	unclassified	0.0190	0.0360	5.97E-01
3p22.2	ITGA9	rs197770	PLPE 18:0/22:6	/	PLPE 18:0/20:4	unclassified	-0.0043	0.0039	2.73E-01
3q23	PAQR9	rs9832727	PE 34:1	/	PE 32:1	elongation	-0.3744	0.0728	2.67E-07
3q23	PAQR9	rs9832727	PE 34:1	/	PE 36:1	elongation	-0.0002	0.0152	9.90E-01
3q23	PAQR9	rs9832727	PE 34:1	/	PE 38:1	elongation	-0.9461	0.2529	1.83E-04
3q23	PAQR9	rs9832727	PE 36:1	/	PE 32:1	elongation	-0.1464	0.0509	4.05E-03
3q23	PAQR9	rs9832727	PE 36:1	/	PE 34:1	elongation	0.0030	0.0035	3.95E-01
3q23	PAQR9	rs9832727	PE 36:1	/	PE 38:1	elongation	-0.4938	0.0982	4.91E-07
3q23	PAQR9	rs9832727	PE 34:1	/	PE 34:3	saturation	-0.4371	0.0571	2.00E-14
3q23	PAQR9	rs9832727	PE 34:1	/	PE 34:2	saturation	-0.0356	0.0033	6.45E-28
3q23	PAQR9	rs9832727	PE 34:1	/	PE 34:0	saturation	-1.0042	0.3618	5.51E-03
3q23	PAQR9	rs9832727	PE 36:1	/	PE 36:5	saturation	-0.2493	0.0443	1.89E-08
3q23	PAQR9	rs9832727	PE 36:1	/	PE 36:4	saturation	-0.0265	0.0043	6.72E-10
3q23	PAQR9	rs9832727	PE 36:1	/	PE 36:3	saturation	-0.0446	0.0054	2.10E-16
3q23	PAQR9	rs9832727	PE 36:1	/	PE 36:2	saturation	-0.0119	0.0012	1.23E-22
3q23	PAQR9	rs9832727	PE 34:1	/	PE 32:2	unclassified	-1.3756	0.2755	5.94E-07
3q23	PAQR9	rs9832727	PE 34:1	/	PE 32:0	unclassified	-1.2343	0.2608	2.21E-06
3q23	PAQR9	rs9832727	PE 34:1	/	PE 36:5	unclassified	-0.4912	0.0872	1.78E-08
3q23	PAQR9	rs9832727	PE 34:1	/	PE 36:4	unclassified	-0.0601	0.0067	2.37E-19
3q23	PAQR9	rs9832727	PE 34:1	/	PE 36:3	unclassified	-0.0969	0.0120	6.37E-16

3q23	PAQR9	rs9832727	PE 34:1 / PE 36:2	unclassified	-0.0256	0.0030	4.97E-17
3q23	PAQR9	rs9832727	PE 34:1 / PE O 38:7	unclassified	-1.1138	0.3923	4.52E-03
3q23	PAQR9	rs9832727	PE 34:1 / PE 38:6	unclassified	-0.0754	0.0133	1.27E-08
3q23	PAQR9	rs9832727	PE 34:1 / PE 38:5	unclassified	-0.1104	0.0170	9.34E-11
3q23	PAQR9	rs9832727	PE 34:1 / PE 38:4	unclassified	-0.0356	0.0064	2.25E-08
3q23	PAQR9	rs9832727	PE 34:1 / PE 38:3	unclassified	-0.3293	0.0485	1.08E-11
3q23	PAQR9	rs9832727	PE 34:1 / PE 38:2	unclassified	-1.0948	0.2616	2.85E-05
3q23	PAQR9	rs9832727	PE 34:1 / PE O 40:3	unclassified	-0.2619	0.0458	1.05E-08
3q23	PAQR9	rs9832727	PE 34:1 / PE 40:6	unclassified	-0.1002	0.0197	3.48E-07
3q23	PAQR9	rs9832727	PE 34:1 / PE 40:5	unclassified	-0.3171	0.0565	1.95E-08
3q23	PAQR9	rs9832727	PE 34:1 / PE 40:4	unclassified	-0.9483	0.1897	5.76E-07
3q23	PAQR9	rs9832727	PE 34:1 / PE 40:3	unclassified	-3.8073	1.3216	3.97E-03
3q23	PAQR9	rs9832727	PE 34:1 / PE 42:7	unclassified	-3.3738	0.8529	7.62E-05
3q23	PAQR9	rs9832727	PE 34:1 / PE 42:6	unclassified	-3.9159	1.4365	6.41E-03
3q23	PAQR9	rs9832727	PE 34:1 / PE 42:5	unclassified	-7.7283	1.7911	1.60E-05
3q23	PAQR9	rs9832727	PE 36:1 / PE 32:2	unclassified	-0.6186	0.1239	5.94E-07
3q23	PAQR9	rs9832727	PE 36:1 / PE 32:0	unclassified	-0.6072	0.1155	1.47E-07
3q23	PAQR9	rs9832727	PE 36:1 / PE 34:3	unclassified	-0.2072	0.0340	1.14E-09
3q23	PAQR9	rs9832727	PE 36:1 / PE 34:2	unclassified	-0.0166	0.0022	3.64E-14
3q23	PAQR9	rs9832727	PE 36:1 / PE 34:0	unclassified	-0.5145	0.1517	6.96E-04
3q23	PAQR9	rs9832727	PE 36:1 / PE O 38:7	unclassified	-0.5248	0.1542	6.66E-04
3q23	PAQR9	rs9832727	PE 36:1 / PE 38:6	unclassified	-0.0342	0.0075	5.55E-06
3q23	PAQR9	rs9832727	PE 36:1 / PE 38:5	unclassified	-0.0567	0.0096	4.12E-09
3q23	PAQR9	rs9832727	PE 36:1 / PE 38:4	unclassified	-0.0172	0.0030	8.76E-09
3q23	PAQR9	rs9832727	PE 36:1 / PE 38:3	unclassified	-0.1516	0.0257	3.46E-09
3q23	PAQR9	rs9832727	PE 36:1 / PE 38:2	unclassified	-0.5731	0.1070	8.47E-08
3q23	PAQR9	rs9832727	PE 36:1 / PE O 40:3	unclassified	-0.1293	0.0212	1.08E-09
3q23	PAQR9	rs9832727	PE 36:1 / PE 40:6	unclassified	-0.0418	0.0098	2.21E-05
3q23	PAQR9	rs9832727	PE 36:1 / PE 40:5	unclassified	-0.1420	0.0314	6.05E-06
3q23	PAQR9	rs9832727	PE 36:1 / PE 40:4	unclassified	-0.3990	0.0894	8.01E-06
3q23	PAQR9	rs9832727	PE 36:1 / PE 40:3	unclassified	-1.7467	0.5206	7.93E-04
3q23	PAQR9	rs9832727	PE 36:1 / PE 42:7	unclassified	-1.5871	0.3596	1.02E-05
3q23	PAQR9	rs9832727	PE 36:1 / PE 42:6	unclassified	-1.8019	0.5870	2.14E-03
3q23	PAQR9	rs9832727	PE 36:1 / PE 42:5	unclassified	-3.2463	0.7245	7.44E-06
5p15.31	PAPD7	rs1566039	SPM 17:0 / SPM 16:0	elongation	0.0006	0.0002	2.48E-04
5p15.31	PAPD7	rs1566039	SPM 17:0 / SPM 18:1	unclassified	0.0047	0.0018	9.69E-03
5p15.31	PAPD7	rs1566039	SPM 17:0 / SPM 20:1	unclassified	0.0090	0.0039	1.94E-02
5p15.31	PAPD7	rs1566039	SPM 17:0 / SPM 22:1	unclassified	0.0017	0.0009	4.67E-02
5p15.31	PAPD7	rs1566039	SPM 17:0 / SPM 23:1	unclassified	0.0042	0.0015	3.98E-03
5p15.31	PAPD7	rs1566039	SPM 17:0 / SPM 24:1	unclassified	0.0012	0.0004	1.96E-03
5p15.31	PAPD7	rs1566039	SPM 17:0 / SPM 16:1	unclassified	0.0043	0.0012	5.54E-04

5p15.31	PAPD7	rs1566039	SPM 17:0	/	SPM dih 16:0	unclassified	0.0174	0.0066	8.80E-03
5p15.31	PAPD7	rs1566039	SPM 17:0	/	SPM 14:0	elongation	0.0041	0.0022	5.79E-02
5p15.31	PAPD7	rs1566039	SPM 17:0	/	SPM 15:0	elongation	0.0045	0.0027	8.89E-02
5p15.31	PAPD7	rs1566039	SPM 17:0	/	SPM 18:2	unclassified	0.1140	0.0416	6.20E-03
5p15.31	PAPD7	rs1566039	SPM 17:0	/	SPM 18:0	elongation	0.0020	0.0009	1.93E-02
5p15.31	PAPD7	rs1566039	SPM 17:0	/	SPM dih 18:0	unclassified	-0.0126	0.1202	9.17E-01
5p15.31	PAPD7	rs1566039	SPM 17:0	/	SPM 20:0	elongation	0.0059	0.0036	1.01E-01
5p15.31	PAPD7	rs1566039	SPM 17:0	/	SPM 22:2	unclassified	0.0417	0.1166	7.20E-01
5p15.31	PAPD7	rs1566039	SPM 17:0	/	SPM 22:0	elongation	0.0014	0.0009	1.10E-01
5p15.31	PAPD7	rs1566039	SPM 17:0	/	SPM dih 22:0	unclassified	0.1325	0.1571	3.99E-01
5p15.31	PAPD7	rs1566039	SPM 17:0	/	SPM 23:0	elongation	0.0026	0.0015	8.68E-02
5p15.31	PAPD7	rs1566039	SPM 17:0	/	SPM dih 23:0	unclassified	-0.3102	0.1969	1.15E-01
5p15.31	PAPD7	rs1566039	SPM 17:0	/	SPM 24:3	unclassified	0.0320	0.0341	3.47E-01
5p15.31	PAPD7	rs1566039	SPM 17:0	/	SPM 24:2	unclassified	0.0030	0.0009	7.24E-04
5p15.31	PAPD7	rs1566039	SPM 17:0	/	SPM 24:0	elongation	0.0037	0.0013	5.40E-03
5p15.31	PAPD7	rs1566039	SPM 17:0	/	SPM dih 24:0	unclassified	-0.0375	0.2489	8.80E-01
6p21.32	AGPAT1	rs1061808	PC 32:0	/	PC 26:0	elongation	-0.1283	0.6907	8.53E-01
6p21.32	AGPAT1	rs1061808	PC 32:0	/	PC 34:0	elongation	0.0105	0.0642	8.70E-01
6p21.32	AGPAT1	rs1061808	PC 32:0	/	PC 36:0	elongation	-0.1494	0.0776	5.40E-02
6p21.32	AGPAT1	rs1061808	PC 32:0	/	PC 38:0	elongation	-0.0760	0.0290	8.71E-03
6p21.32	AGPAT1	rs1061808	PC 32:0	/	PC 40:0	elongation	-0.2102	0.1449	1.47E-01
6p21.32	AGPAT1	rs1061808	PC 32:0	/	PC 32:2	saturation	-0.0192	0.0397	6.29E-01
6p21.32	AGPAT1	rs1061808	PC 32:0	/	PC 32:1	saturation	-0.0196	0.0085	2.15E-02
6p21.32	AGPAT1	rs1061808	PC 32:0	/	PC 30:1	unclassified	-0.4929	0.7887	5.32E-01
6p21.32	AGPAT1	rs1061808	PC 32:0	/	PC 30:0	unclassified	0.0199	0.0307	5.15E-01
6p21.32	AGPAT1	rs1061808	PC 32:0	/	PC O 32:1	unclassified	-0.1419	0.0405	4.60E-04
6p21.32	AGPAT1	rs1061808	PC 32:0	/	PC O 32:0	unclassified	-0.0293	0.0249	2.38E-01
6p21.32	AGPAT1	rs1061808	PC 32:0	/	PC O 34:3	unclassified	-0.0554	0.0194	4.19E-03
6p21.32	AGPAT1	rs1061808	PC 32:0	/	PC O 34:2	unclassified	-0.0142	0.0094	1.30E-01
6p21.32	AGPAT1	rs1061808	PC 32:0	/	PC O 34:1	unclassified	-0.0323	0.0074	1.21E-05
6p21.32	AGPAT1	rs1061808	PC 32:0	/	PC O 34:0	unclassified	-0.1918	0.0725	8.13E-03
6p21.32	AGPAT1	rs1061808	PC 32:0	/	PC 34:4	unclassified	-0.1059	0.1005	2.92E-01
6p21.32	AGPAT1	rs1061808	PC 32:0	/	PC 34:3	unclassified	-0.0144	0.0060	1.66E-02
6p21.32	AGPAT1	rs1061808	PC 32:0	/	PC 34:2	unclassified	-0.0006	0.0002	1.27E-04
6p21.32	AGPAT1	rs1061808	PC 32:0	/	PC O 36:5	unclassified	-0.0418	0.0103	5.08E-05
6p21.32	AGPAT1	rs1061808	PC 32:0	/	PC O 36:4	unclassified	-0.0203	0.0063	1.19E-03
6p21.32	AGPAT1	rs1061808	PC 32:0	/	PC O 36:3	unclassified	-0.0301	0.0138	2.90E-02
6p21.32	AGPAT1	rs1061808	PC 32:0	/	PC O 36:2	unclassified	-0.0153	0.0079	5.28E-02
6p21.32	AGPAT1	rs1061808	PC 32:0	/	PC O 36:1	unclassified	-0.0380	0.0103	2.10E-04
6p21.32	AGPAT1	rs1061808	PC 32:0	/	PC O 36:0	unclassified	-0.5504	0.2767	4.67E-02
6p21.32	AGPAT1	rs1061808	PC 32:0	/	PC 36:5	unclassified	-0.0075	0.0065	2.49E-01

6p21.32	AGPAT1	rs1061808	PC 32:0 / PC 36:4	unclassified	-0.0015	0.0005	9.55E-04
6p21.32	AGPAT1	rs1061808	PC 32:0 / PC 36:3	unclassified	-0.0013	0.0006	2.08E-02
6p21.32	AGPAT1	rs1061808	PC 32:0 / PC 36:2	unclassified	-0.0010	0.0003	1.96E-03
6p21.32	AGPAT1	rs1061808	PC 32:0 / PC 36:1	unclassified	-0.0108	0.0023	2.19E-06
6p21.32	AGPAT1	rs1061808	PC 32:0 / PC O 38:5	unclassified	-0.0245	0.0058	2.76E-05
6p21.32	AGPAT1	rs1061808	PC 32:0 / PC O 38:4	unclassified	-0.0290	0.0087	8.06E-04
6p21.32	AGPAT1	rs1061808	PC 32:0 / PC O 38:3	unclassified	-0.0641	0.0264	1.51E-02
6p21.32	AGPAT1	rs1061808	PC 32:0 / PC O 38:2	unclassified	-0.0329	0.2319	8.87E-01
6p21.32	AGPAT1	rs1061808	PC 32:0 / PC O 38:1	unclassified	-0.6705	0.6436	2.98E-01
6p21.32	AGPAT1	rs1061808	PC 32:0 / PC 38:7	unclassified	-0.1569	0.0641	1.43E-02
6p21.32	AGPAT1	rs1061808	PC 32:0 / PC 38:6	unclassified	-0.0045	0.0013	5.71E-04
6p21.32	AGPAT1	rs1061808	PC 32:0 / PC 38:5	unclassified	-0.0062	0.0016	6.99E-05
6p21.32	AGPAT1	rs1061808	PC 32:0 / PC 38:4	unclassified	-0.0028	0.0010	3.43E-03
6p21.32	AGPAT1	rs1061808	PC 32:0 / PC 38:3	unclassified	-0.0049	0.0029	9.18E-02
6p21.32	AGPAT1	rs1061808	PC 32:0 / PC 38:2	unclassified	-0.1738	0.1276	1.73E-01
6p21.32	AGPAT1	rs1061808	PC 32:0 / PC 38:1	unclassified	-0.1316	0.0637	3.90E-02
6p21.32	AGPAT1	rs1061808	PC 32:0 / PC O 40:6	unclassified	-0.1040	0.0278	1.87E-04
6p21.32	AGPAT1	rs1061808	PC 32:0 / PC O 40:5	unclassified	-0.1269	0.0371	6.32E-04
6p21.32	AGPAT1	rs1061808	PC 32:0 / PC O 40:4	unclassified	-0.1937	0.0584	9.17E-04
6p21.32	AGPAT1	rs1061808	PC 32:0 / PC 40:7	unclassified	-0.0300	0.0173	8.34E-02
6p21.32	AGPAT1	rs1061808	PC 32:0 / PC 40:6	unclassified	-0.0151	0.0046	9.78E-04
6p21.32	AGPAT1	rs1061808	PC 32:0 / PC 40:5	unclassified	-0.0510	0.0108	2.30E-06
6p21.32	AGPAT1	rs1061808	PC 32:0 / PC 40:4	unclassified	-0.1339	0.0331	5.15E-05
6p21.32	AGPAT1	rs1061808	PC 32:0 / PC 40:3	unclassified	-0.2391	0.1366	8.00E-02
6p21.32	AGPAT1	rs1061808	PC 32:0 / PC 40:2	unclassified	-0.1145	0.1174	3.29E-01
6p21.32	AGPAT1	rs1061808	PC 32:0 / PC 40:1	unclassified	-0.0637	0.0735	3.86E-01
6p21.32	AGPAT1	rs1061808	PC 32:0 / PC O 42:6	unclassified	-0.4519	0.1602	4.78E-03
6p21.32	AGPAT1	rs1061808	PC 32:0 / PC O 42:5	unclassified	-0.1808	0.1090	9.70E-02
6p21.32	AGPAT1	rs1061808	PC 32:0 / PC 42:5	unclassified	-0.2687	0.1706	1.15E-01
6p21.32	AGPAT1	rs1061808	PC 32:0 / PC 42:4	unclassified	-0.8565	0.4100	3.67E-02
6p21.32	AGPAT1	rs1061808	PC 32:0 / PC 34:1	unclassified	-0.0024	0.0003	1.82E-14
10q24.31	PKD2L1	rs603424	LPC 16:1 / LPC 18:1	elongation	-0.0057	0.0009	6.83E-11
10q24.31	PKD2L1	rs603424	LPC 16:1 / LPC 15:0	unclassified	-0.0810	0.0199	4.50E-05
10q24.31	PKD2L1	rs603424	LPC 16:1 / LPC 18:3	unclassified	-0.2538	0.0483	1.52E-07
10q24.31	PKD2L1	rs603424	LPC 16:1 / LPC 18:2	unclassified	-0.0059	0.0011	2.82E-08
10q24.31	PKD2L1	rs603424	LPC 16:1 / LPC 18:0	unclassified	-0.0024	0.0005	3.92E-07
10q24.31	PKD2L1	rs603424	LPC 16:1 / LPC 20:5	unclassified	-0.2217	0.0505	1.13E-05
10q24.31	PKD2L1	rs603424	LPC 16:1 / LPC 20:4	unclassified	-0.0229	0.0041	1.97E-08
10q24.31	PKD2L1	rs603424	LPC 16:1 / LPC 20:3	unclassified	-0.0584	0.0090	1.03E-10
10q24.31	PKD2L1	rs603424	LPC 16:1 / LPC 20:0	unclassified	-0.2269	0.0819	5.57E-03
10q24.31	PKD2L1	rs603424	LPC 16:1 / LPC 22:6	unclassified	-0.0919	0.0203	5.85E-06

10q24.31	PKD2L1	rs603424	LPC 16:1 / LPC 22:5	unclassified	-0.3135	0.0532	3.80E-09
10q24.31	PKD2L1	rs603424	LPC 16:1 / LPC 22:4	unclassified	-0.4582	0.1078	2.12E-05
10q24.31	PKD2L1	rs603424	LPC 16:1 / LPC 22:0	unclassified	-0.1342	0.0497	6.88E-03
10q24.31	PKD2L1	rs603424	LPC 16:1 / LPC 16:0	saturation	-0.0009	0.0001	2.25E-15
10q25.3	PNLIPRP1	rs10885997	PC 36:1 / PC 30:1	elongation	1.7219	2.0456	4.00E-01
10q25.3	PNLIPRP1	rs10885997	PC 36:1 / PC 32:1	elongation	-0.0589	0.0223	8.11E-03
10q25.3	PNLIPRP1	rs10885997	PC 36:1 / PC 38:1	elongation	-0.1256	0.0949	1.85E-01
10q25.3	PNLIPRP1	rs10885997	PC 36:1 / PC 40:1	elongation	-0.0848	0.1896	6.55E-01
10q25.3	PNLIPRP1	rs10885997	PC 36:1 / PC 36:5	saturation	-0.0450	0.0183	1.42E-02
10q25.3	PNLIPRP1	rs10885997	PC 36:1 / PC 36:4	saturation	-0.0057	0.0016	4.01E-04
10q25.3	PNLIPRP1	rs10885997	PC 36:1 / PC 36:3	saturation	-0.0094	0.0016	8.72E-09
10q25.3	PNLIPRP1	rs10885997	PC 36:1 / PC 36:2	saturation	-0.0034	0.0009	1.91E-04
10q25.3	PNLIPRP1	rs10885997	PC 36:1 / PC 36:0	saturation	0.1195	0.2250	5.95E-01
10q25.3	PNLIPRP1	rs10885997	PC 36:1 / PC 26:0	unclassified	0.0452	1.7631	9.80E-01
10q25.3	PNLIPRP1	rs10885997	PC 36:1 / PC 30:0	unclassified	-0.1133	0.0952	2.34E-01
10q25.3	PNLIPRP1	rs10885997	PC 36:1 / PC O 32:1	unclassified	-0.3872	0.1441	7.22E-03
10q25.3	PNLIPRP1	rs10885997	PC 36:1 / PC O 32:0	unclassified	-0.2177	0.1010	3.12E-02
10q25.3	PNLIPRP1	rs10885997	PC 36:1 / PC 32:2	unclassified	-0.3321	0.1159	4.17E-03
10q25.3	PNLIPRP1	rs10885997	PC 36:1 / PC 32:0	unclassified	-0.0678	0.0165	3.93E-05
10q25.3	PNLIPRP1	rs10885997	PC 36:1 / PC O 34:3	unclassified	-0.1579	0.0605	9.08E-03
10q25.3	PNLIPRP1	rs10885997	PC 36:1 / PC O 34:2	unclassified	-0.1002	0.0323	1.95E-03
10q25.3	PNLIPRP1	rs10885997	PC 36:1 / PC O 34:1	unclassified	-0.1007	0.0257	9.20E-05
10q25.3	PNLIPRP1	rs10885997	PC 36:1 / PC O 34:0	unclassified	-0.1108	0.2225	6.18E-01
10q25.3	PNLIPRP1	rs10885997	PC 36:1 / PC 34:4	unclassified	-0.6222	0.2707	2.15E-02
10q25.3	PNLIPRP1	rs10885997	PC 36:1 / PC 34:2	unclassified	-0.0033	0.0006	2.29E-08
10q25.3	PNLIPRP1	rs10885997	PC 36:1 / PC 34:0	unclassified	-0.1941	0.1847	2.93E-01
10q25.3	PNLIPRP1	rs10885997	PC 36:1 / PC O 36:5	unclassified	-0.0825	0.0345	1.67E-02
10q25.3	PNLIPRP1	rs10885997	PC 36:1 / PC O 36:4	unclassified	-0.0888	0.0216	4.00E-05
10q25.3	PNLIPRP1	rs10885997	PC 36:1 / PC O 36:3	unclassified	-0.1515	0.0436	5.13E-04
10q25.3	PNLIPRP1	rs10885997	PC 36:1 / PC O 36:2	unclassified	-0.0760	0.0260	3.47E-03
10q25.3	PNLIPRP1	rs10885997	PC 36:1 / PC O 36:1	unclassified	-0.1020	0.0304	8.00E-04
10q25.3	PNLIPRP1	rs10885997	PC 36:1 / PC O 36:0	unclassified	0.2959	0.6824	6.65E-01
10q25.3	PNLIPRP1	rs10885997	PC 36:1 / PC O 38:5	unclassified	-0.0714	0.0209	6.26E-04
10q25.3	PNLIPRP1	rs10885997	PC 36:1 / PC O 38:4	unclassified	-0.0928	0.0288	1.30E-03
10q25.3	PNLIPRP1	rs10885997	PC 36:1 / PC O 38:3	unclassified	-0.1424	0.0789	7.13E-02
10q25.3	PNLIPRP1	rs10885997	PC 36:1 / PC O 38:2	unclassified	0.0492	0.5562	9.30E-01
10q25.3	PNLIPRP1	rs10885997	PC 36:1 / PC O 38:1	unclassified	0.5036	1.5998	7.53E-01
10q25.3	PNLIPRP1	rs10885997	PC 36:1 / PC 38:7	unclassified	-0.2279	0.1893	2.29E-01
10q25.3	PNLIPRP1	rs10885997	PC 36:1 / PC 38:6	unclassified	-0.0113	0.0044	9.91E-03
10q25.3	PNLIPRP1	rs10885997	PC 36:1 / PC 38:5	unclassified	-0.0109	0.0045	1.61E-02
10q25.3	PNLIPRP1	rs10885997	PC 36:1 / PC 38:4	unclassified	-0.0054	0.0028	4.85E-02

10q25.3	PNLIPRP1	rs10885997	PC 36:1 / PC 38:3	unclassified	-0.0075	0.0066	2.60E-01
10q25.3	PNLIPRP1	rs10885997	PC 36:1 / PC 38:2	unclassified	-0.1623	0.2530	5.21E-01
10q25.3	PNLIPRP1	rs10885997	PC 36:1 / PC 38:0	unclassified	-0.1390	0.0658	3.45E-02
10q25.3	PNLIPRP1	rs10885997	PC 36:1 / PC O 40:6	unclassified	-0.0903	0.0923	3.28E-01
10q25.3	PNLIPRP1	rs10885997	PC 36:1 / PC O 40:5	unclassified	-0.3493	0.1163	2.68E-03
10q25.3	PNLIPRP1	rs10885997	PC 36:1 / PC O 40:4	unclassified	-0.3819	0.1757	2.98E-02
10q25.3	PNLIPRP1	rs10885997	PC 36:1 / PC 40:7	unclassified	-0.1540	0.0528	3.52E-03
10q25.3	PNLIPRP1	rs10885997	PC 36:1 / PC 40:6	unclassified	-0.0203	0.0132	1.25E-01
10q25.3	PNLIPRP1	rs10885997	PC 36:1 / PC 40:5	unclassified	-0.0410	0.0274	1.35E-01
10q25.3	PNLIPRP1	rs10885997	PC 36:1 / PC 40:4	unclassified	-0.1008	0.0855	2.38E-01
10q25.3	PNLIPRP1	rs10885997	PC 36:1 / PC 40:3	unclassified	-0.4902	0.2405	4.15E-02
10q25.3	PNLIPRP1	rs10885997	PC 36:1 / PC 40:2	unclassified	-0.7865	0.3095	1.11E-02
10q25.3	PNLIPRP1	rs10885997	PC 36:1 / PC 40:0	unclassified	-0.4509	0.3965	2.55E-01
10q25.3	PNLIPRP1	rs10885997	PC 36:1 / PC O 42:6	unclassified	-1.0469	0.4987	3.58E-02
10q25.3	PNLIPRP1	rs10885997	PC 36:1 / PC O 42:5	unclassified	-0.7446	0.3480	3.24E-02
10q25.3	PNLIPRP1	rs10885997	PC 36:1 / PC 42:5	unclassified	-0.5844	0.4897	2.33E-01
10q25.3	PNLIPRP1	rs10885997	PC 36:1 / PC 42:4	unclassified	-1.0514	1.1280	3.51E-01
10q25.3	PNLIPRP1	rs10885997	PC 36:1 / PC 34:1	elongation	-0.0048	0.0008	3.28E-10
10q25.3	PNLIPRP1	rs10885997	PC 36:1 / PC 34:3	unclassified	-0.0992	0.0163	1.15E-09
11p15.4	SYT9	rs10769780	PC O 42:6 / PC O 40:6	elongation	0.0036	0.0020	6.67E-02
11p15.4	SYT9	rs10769780	PC O 42:6 / PC O 42:5	saturation	0.0176	0.0068	9.96E-03
11p15.4	SYT9	rs10769780	PC O 42:6 / PC 26:0	unclassified	0.0622	0.0569	2.75E-01
11p15.4	SYT9	rs10769780	PC O 42:6 / PC 30:1	unclassified	0.0204	0.0594	7.31E-01
11p15.4	SYT9	rs10769780	PC O 42:6 / PC 30:0	unclassified	0.0063	0.0036	7.88E-02
11p15.4	SYT9	rs10769780	PC O 42:6 / PC O 32:1	unclassified	0.0089	0.0038	1.84E-02
11p15.4	SYT9	rs10769780	PC O 42:6 / PC O 32:0	unclassified	0.0090	0.0029	1.77E-03
11p15.4	SYT9	rs10769780	PC O 42:6 / PC 32:2	unclassified	0.0048	0.0038	2.11E-01
11p15.4	SYT9	rs10769780	PC O 42:6 / PC 32:1	unclassified	0.0019	0.0010	6.01E-02
11p15.4	SYT9	rs10769780	PC O 42:6 / PC 32:0	unclassified	0.0016	0.0006	1.24E-02
11p15.4	SYT9	rs10769780	PC O 42:6 / PC O 34:3	unclassified	0.0038	0.0015	1.16E-02
11p15.4	SYT9	rs10769780	PC O 42:6 / PC O 34:2	unclassified	0.0032	0.0009	2.12E-04
11p15.4	SYT9	rs10769780	PC O 42:6 / PC O 34:1	unclassified	0.0031	0.0010	1.70E-03
11p15.4	SYT9	rs10769780	PC O 42:6 / PC O 34:0	unclassified	0.0216	0.0074	3.37E-03
11p15.4	SYT9	rs10769780	PC O 42:6 / PC 34:4	unclassified	0.0177	0.0098	7.00E-02
11p15.4	SYT9	rs10769780	PC O 42:6 / PC 34:3	unclassified	0.0020	0.0008	8.19E-03
11p15.4	SYT9	rs10769780	PC O 42:6 / PC 34:2	unclassified	0.0001	0.0000	9.86E-04
11p15.4	SYT9	rs10769780	PC O 42:6 / PC 34:1	unclassified	0.0001	0.0001	5.50E-03
11p15.4	SYT9	rs10769780	PC O 42:6 / PC 34:0	unclassified	0.0072	0.0042	8.52E-02
11p15.4	SYT9	rs10769780	PC O 42:6 / PC O 36:5	unclassified	0.0022	0.0009	1.59E-02
11p15.4	SYT9	rs10769780	PC O 42:6 / PC O 36:4	unclassified	0.0017	0.0006	3.66E-03
11p15.4	SYT9	rs10769780	PC O 42:6 / PC O 36:3	unclassified	0.0053	0.0013	3.62E-05

11p15.4	SYT9	rs10769780	PC O 42:6	/	PC O 36:2	unclassified	0.0025	0.0007	3.86E-04
11p15.4	SYT9	rs10769780	PC O 42:6	/	PC O 36:1	unclassified	0.0038	0.0011	8.95E-04
11p15.4	SYT9	rs10769780	PC O 42:6	/	PC O 36:0	unclassified	0.0238	0.0205	2.46E-01
11p15.4	SYT9	rs10769780	PC O 42:6	/	PC 36:5	unclassified	0.0009	0.0006	1.30E-01
11p15.4	SYT9	rs10769780	PC O 42:6	/	PC 36:4	unclassified	0.0001	0.0001	4.87E-02
11p15.4	SYT9	rs10769780	PC O 42:6	/	PC 36:3	unclassified	0.0003	0.0001	1.16E-03
11p15.4	SYT9	rs10769780	PC O 42:6	/	PC 36:2	unclassified	0.0002	0.0000	5.58E-05
11p15.4	SYT9	rs10769780	PC O 42:6	/	PC 36:1	unclassified	0.0010	0.0003	2.00E-04
11p15.4	SYT9	rs10769780	PC O 42:6	/	PC 36:0	unclassified	0.0139	0.0054	1.01E-02
11p15.4	SYT9	rs10769780	PC O 42:6	/	PC O 38:5	unclassified	0.0015	0.0005	4.87E-03
11p15.4	SYT9	rs10769780	PC O 42:6	/	PC O 38:4	unclassified	0.0020	0.0007	6.54E-03
11p15.4	SYT9	rs10769780	PC O 42:6	/	PC O 38:3	unclassified	0.0073	0.0024	1.95E-03
11p15.4	SYT9	rs10769780	PC O 42:6	/	PC O 38:2	unclassified	0.0307	0.0150	4.10E-02
11p15.4	SYT9	rs10769780	PC O 42:6	/	PC O 38:1	unclassified	0.0423	0.0463	3.60E-01
11p15.4	SYT9	rs10769780	PC O 42:6	/	PC 38:7	unclassified	0.0047	0.0051	3.60E-01
11p15.4	SYT9	rs10769780	PC O 42:6	/	PC 38:6	unclassified	0.0003	0.0001	3.21E-02
11p15.4	SYT9	rs10769780	PC O 42:6	/	PC 38:5	unclassified	0.0003	0.0002	4.58E-02
11p15.4	SYT9	rs10769780	PC O 42:6	/	PC 38:4	unclassified	0.0002	0.0001	1.49E-02
11p15.4	SYT9	rs10769780	PC O 42:6	/	PC 38:3	unclassified	0.0010	0.0003	7.79E-04
11p15.4	SYT9	rs10769780	PC O 42:6	/	PC 38:2	unclassified	0.0036	0.0074	6.24E-01
11p15.4	SYT9	rs10769780	PC O 42:6	/	PC 38:1	unclassified	0.0046	0.0024	5.35E-02
11p15.4	SYT9	rs10769780	PC O 42:6	/	PC 38:0	unclassified	0.0052	0.0019	5.73E-03
11p15.4	SYT9	rs10769780	PC O 42:6	/	PC O 40:5	unclassified	0.0066	0.0026	1.01E-02
11p15.4	SYT9	rs10769780	PC O 42:6	/	PC O 40:4	unclassified	0.0098	0.0041	1.59E-02
11p15.4	SYT9	rs10769780	PC O 42:6	/	PC 40:7	unclassified	0.0032	0.0015	3.08E-02
11p15.4	SYT9	rs10769780	PC O 42:6	/	PC 40:6	unclassified	0.0009	0.0004	2.28E-02
11p15.4	SYT9	rs10769780	PC O 42:6	/	PC 40:5	unclassified	0.0023	0.0011	3.46E-02
11p15.4	SYT9	rs10769780	PC O 42:6	/	PC 40:4	unclassified	0.0056	0.0030	6.73E-02
11p15.4	SYT9	rs10769780	PC O 42:6	/	PC 40:3	unclassified	0.0098	0.0065	1.35E-01
11p15.4	SYT9	rs10769780	PC O 42:6	/	PC 40:2	unclassified	0.0244	0.0089	5.92E-03
11p15.4	SYT9	rs10769780	PC O 42:6	/	PC 40:1	unclassified	0.0158	0.0053	2.83E-03
11p15.4	SYT9	rs10769780	PC O 42:6	/	PC 40:0	unclassified	0.0214	0.0111	5.33E-02
11p15.4	SYT9	rs10769780	PC O 42:6	/	PC 42:5	unclassified	0.0096	0.0126	4.46E-01
11p15.4	SYT9	rs10769780	PC O 42:6	/	PC 42:4	unclassified	0.0525	0.0295	7.52E-02
11q12.1	OR5AQ1P	rs2945816	PC 26:0	/	PC 30:0	elongation	-0.0438	0.0092	2.14E-06
11q12.1	OR5AQ1P	rs2945816	PC 26:0	/	PC 32:0	elongation	-0.0130	0.0025	2.07E-07
11q12.1	OR5AQ1P	rs2945816	PC 26:0	/	PC 34:0	elongation	-0.0572	0.0135	2.11E-05
11q12.1	OR5AQ1P	rs2945816	PC 26:0	/	PC 38:0	elongation	-0.0353	0.0073	1.38E-06
11q12.1	OR5AQ1P	rs2945816	PC 26:0	/	PC 40:0	elongation	-0.1889	0.0402	2.67E-06
11q12.1	OR5AQ1P	rs2945816	PC 26:0	/	PC 30:1	unclassified	-0.1163	0.1206	3.35E-01
11q12.1	OR5AQ1P	rs2945816	PC 26:0	/	PC O 32:1	unclassified	-0.0634	0.0150	2.42E-05

11q12.1	OR5AQ1P	rs2945816	PC 26:0 / PC O 32:0	unclassified	-0.0515	0.0108	1.79E-06
11q12.1	OR5AQ1P	rs2945816	PC 26:0 / PC 32:2	unclassified	-0.0313	0.0093	7.27E-04
11q12.1	OR5AQ1P	rs2945816	PC 26:0 / PC 32:1	unclassified	-0.0139	0.0025	2.24E-08
11q12.1	OR5AQ1P	rs2945816	PC 26:0 / PC O 34:3	unclassified	-0.0187	0.0057	9.34E-04
11q12.1	OR5AQ1P	rs2945816	PC 26:0 / PC O 34:2	unclassified	-0.0136	0.0033	4.40E-05
11q12.1	OR5AQ1P	rs2945816	PC 26:0 / PC O 34:1	unclassified	-0.0196	0.0037	9.07E-08
11q12.1	OR5AQ1P	rs2945816	PC 26:0 / PC O 34:0	unclassified	-0.1204	0.0246	9.41E-07
11q12.1	OR5AQ1P	rs2945816	PC 26:0 / PC 34:4	unclassified	-0.1069	0.0256	3.00E-05
11q12.1	OR5AQ1P	rs2945816	PC 26:0 / PC 34:3	unclassified	-0.0101	0.0023	1.33E-05
11q12.1	OR5AQ1P	rs2945816	PC 26:0 / PC 34:2	unclassified	-0.0003	0.0001	1.33E-05
11q12.1	OR5AQ1P	rs2945816	PC 26:0 / PC 34:1	unclassified	-0.0009	0.0002	3.32E-08
11q12.1	OR5AQ1P	rs2945816	PC 26:0 / PC O 36:5	unclassified	-0.0193	0.0041	1.83E-06
11q12.1	OR5AQ1P	rs2945816	PC 26:0 / PC O 36:4	unclassified	-0.0134	0.0026	2.07E-07
11q12.1	OR5AQ1P	rs2945816	PC 26:0 / PC O 36:3	unclassified	-0.0248	0.0053	2.57E-06
11q12.1	OR5AQ1P	rs2945816	PC 26:0 / PC O 36:2	unclassified	-0.0123	0.0027	4.57E-06
11q12.1	OR5AQ1P	rs2945816	PC 26:0 / PC O 36:1	unclassified	-0.0215	0.0041	1.85E-07
11q12.1	OR5AQ1P	rs2945816	PC 26:0 / PC O 36:0	unclassified	-0.3439	0.0695	7.50E-07
11q12.1	OR5AQ1P	rs2945816	PC 26:0 / PC 36:4	unclassified	-0.0011	0.0002	2.76E-07
11q12.1	OR5AQ1P	rs2945816	PC 26:0 / PC 36:3	unclassified	-0.0013	0.0003	2.81E-06
11q12.1	OR5AQ1P	rs2945816	PC 26:0 / PC 36:2	unclassified	-0.0007	0.0001	2.40E-06
11q12.1	OR5AQ1P	rs2945816	PC 26:0 / PC 36:0	unclassified	-0.0501	0.0172	3.62E-03
11q12.1	OR5AQ1P	rs2945816	PC 26:0 / PC O 38:5	unclassified	-0.0145	0.0027	1.08E-07
11q12.1	OR5AQ1P	rs2945816	PC 26:0 / PC O 38:4	unclassified	-0.0190	0.0035	5.52E-08
11q12.1	OR5AQ1P	rs2945816	PC 26:0 / PC O 38:3	unclassified	-0.0430	0.0090	1.91E-06
11q12.1	OR5AQ1P	rs2945816	PC 26:0 / PC O 38:2	unclassified	-0.0987	0.0411	1.63E-02
11q12.1	OR5AQ1P	rs2945816	PC 26:0 / PC O 38:1	unclassified	-0.1692	0.1134	1.36E-01
11q12.1	OR5AQ1P	rs2945816	PC 26:0 / PC 38:7	unclassified	-0.0801	0.0200	6.02E-05
11q12.1	OR5AQ1P	rs2945816	PC 26:0 / PC 38:6	unclassified	-0.0028	0.0005	3.43E-08
11q12.1	OR5AQ1P	rs2945816	PC 26:0 / PC 38:3	unclassified	-0.0053	0.0010	4.50E-08
11q12.1	OR5AQ1P	rs2945816	PC 26:0 / PC 38:2	unclassified	-0.0569	0.0207	6.00E-03
11q12.1	OR5AQ1P	rs2945816	PC 26:0 / PC 38:1	unclassified	-0.0189	0.0066	4.29E-03
11q12.1	OR5AQ1P	rs2945816	PC 26:0 / PC O 40:6	unclassified	-0.0543	0.0110	7.58E-07
11q12.1	OR5AQ1P	rs2945816	PC 26:0 / PC O 40:5	unclassified	-0.0650	0.0138	2.49E-06
11q12.1	OR5AQ1P	rs2945816	PC 26:0 / PC O 40:4	unclassified	-0.0891	0.0210	2.10E-05
11q12.1	OR5AQ1P	rs2945816	PC 26:0 / PC 40:5	unclassified	-0.0220	0.0040	3.78E-08
11q12.1	OR5AQ1P	rs2945816	PC 26:0 / PC 40:4	unclassified	-0.0558	0.0111	4.79E-07
11q12.1	OR5AQ1P	rs2945816	PC 26:0 / PC 40:3	unclassified	-0.0602	0.0189	1.47E-03
11q12.1	OR5AQ1P	rs2945816	PC 26:0 / PC 40:2	unclassified	-0.0825	0.0245	7.42E-04
11q12.1	OR5AQ1P	rs2945816	PC 26:0 / PC 40:1	unclassified	-0.0356	0.0149	1.71E-02
11q12.1	OR5AQ1P	rs2945816	PC 26:0 / PC O 42:6	unclassified	-0.2545	0.0483	1.40E-07
11q12.1	OR5AQ1P	rs2945816	PC 26:0 / PC O 42:5	unclassified	-0.1435	0.0346	3.28E-05

11q12.1	OR5AQ1P	rs2945816	PC 26:0 / PC 42:5	unclassified	-0.1834	0.0449	4.32E-05
11q12.1	OR5AQ1P	rs2945816	PC 26:0 / PC 42:4	unclassified	-0.4219	0.0834	4.17E-07
11q12.1	OR5AQ1P	rs2945816	PC 26:0 / PC 36:5	unclassified	-0.0105	0.0019	1.92E-08
11q12.1	OR5AQ1P	rs2945816	PC 26:0 / PC 36:1	unclassified	-0.0053	0.0009	2.93E-09
11q12.1	OR5AQ1P	rs2945816	PC 26:0 / PC 38:5	unclassified	-0.0039	0.0007	1.21E-08
11q12.1	OR5AQ1P	rs2945816	PC 26:0 / PC 38:4	unclassified	-0.0022	0.0004	9.31E-09
11q12.1	OR5AQ1P	rs2945816	PC 26:0 / PC 40:7	unclassified	-0.0393	0.0069	1.12E-08
11q12.1	OR5AQ1P	rs2945816	PC 26:0 / PC 40:6	unclassified	-0.0098	0.0017	7.27E-09
11q14.1	DLG2	rs17148090	PC O 42:5 / PC O 36:5	elongation	-0.0088	0.0024	2.35E-04
11q14.1	DLG2	rs17148090	PC O 42:5 / PC O 38:5	elongation	-0.0038	0.0013	3.26E-03
11q14.1	DLG2	rs17148090	PC O 42:5 / PC O 40:5	elongation	-0.0186	0.0058	1.34E-03
11q14.1	DLG2	rs17148090	PC O 42:5 / PC O 42:6	saturation	-0.0451	0.0235	5.48E-02
11q14.1	DLG2	rs17148090	PC O 42:5 / PC 26:0	unclassified	-0.2542	0.1525	9.57E-02
11q14.1	DLG2	rs17148090	PC O 42:5 / PC 30:1	unclassified	-0.1194	0.1546	4.40E-01
11q14.1	DLG2	rs17148090	PC O 42:5 / PC 30:0	unclassified	-0.0114	0.0087	1.87E-01
11q14.1	DLG2	rs17148090	PC O 42:5 / PC O 32:1	unclassified	-0.0191	0.0094	4.27E-02
11q14.1	DLG2	rs17148090	PC O 42:5 / PC O 32:0	unclassified	-0.0093	0.0067	1.68E-01
11q14.1	DLG2	rs17148090	PC O 42:5 / PC 32:2	unclassified	-0.0236	0.0100	1.78E-02
11q14.1	DLG2	rs17148090	PC O 42:5 / PC 32:1	unclassified	-0.0068	0.0024	4.24E-03
11q14.1	DLG2	rs17148090	PC O 42:5 / PC 32:0	unclassified	-0.0050	0.0015	1.02E-03
11q14.1	DLG2	rs17148090	PC O 42:5 / PC O 34:3	unclassified	-0.0126	0.0039	1.39E-03
11q14.1	DLG2	rs17148090	PC O 42:5 / PC O 34:2	unclassified	-0.0077	0.0021	3.02E-04
11q14.1	DLG2	rs17148090	PC O 42:5 / PC O 34:1	unclassified	-0.0056	0.0022	1.15E-02
11q14.1	DLG2	rs17148090	PC O 42:5 / PC O 34:0	unclassified	-0.0277	0.0179	1.23E-01
11q14.1	DLG2	rs17148090	PC O 42:5 / PC 34:4	unclassified	-0.0857	0.0246	4.89E-04
11q14.1	DLG2	rs17148090	PC O 42:5 / PC 34:3	unclassified	-0.0051	0.0019	6.18E-03
11q14.1	DLG2	rs17148090	PC O 42:5 / PC 34:2	unclassified	-0.0002	0.0001	3.09E-04
11q14.1	DLG2	rs17148090	PC O 42:5 / PC 34:1	unclassified	-0.0004	0.0001	7.25E-04
11q14.1	DLG2	rs17148090	PC O 42:5 / PC 34:0	unclassified	-0.0198	0.0118	9.21E-02
11q14.1	DLG2	rs17148090	PC O 42:5 / PC O 36:4	unclassified	-0.0049	0.0014	5.69E-04
11q14.1	DLG2	rs17148090	PC O 42:5 / PC O 36:3	unclassified	-0.0110	0.0032	5.33E-04
11q14.1	DLG2	rs17148090	PC O 42:5 / PC O 36:2	unclassified	-0.0060	0.0017	5.18E-04
11q14.1	DLG2	rs17148090	PC O 42:5 / PC O 36:1	unclassified	-0.0092	0.0026	4.95E-04
11q14.1	DLG2	rs17148090	PC O 42:5 / PC O 36:0	unclassified	-0.0308	0.0516	5.51E-01
11q14.1	DLG2	rs17148090	PC O 42:5 / PC 36:5	unclassified	-0.0043	0.0016	5.20E-03
11q14.1	DLG2	rs17148090	PC O 42:5 / PC 36:4	unclassified	-0.0006	0.0001	1.30E-05
11q14.1	DLG2	rs17148090	PC O 42:5 / PC 36:3	unclassified	-0.0008	0.0002	6.94E-05
11q14.1	DLG2	rs17148090	PC O 42:5 / PC 36:2	unclassified	-0.0004	0.0001	8.48E-05
11q14.1	DLG2	rs17148090	PC O 42:5 / PC 36:1	unclassified	-0.0021	0.0007	9.44E-04
11q14.1	DLG2	rs17148090	PC O 42:5 / PC 36:0	unclassified	0.0012	0.0143	9.35E-01
11q14.1	DLG2	rs17148090	PC O 42:5 / PC O 38:4	unclassified	-0.0055	0.0017	1.42E-03

11q14.1	DLG2	rs17148090	PC O 42:5 / PC O 38:3	unclassified	-0.0181	0.0054	8.33E-04
11q14.1	DLG2	rs17148090	PC O 42:5 / PC O 38:2	unclassified	0.0045	0.0355	8.99E-01
11q14.1	DLG2	rs17148090	PC O 42:5 / PC O 38:1	unclassified	-0.1669	0.1201	1.65E-01
11q14.1	DLG2	rs17148090	PC O 42:5 / PC 38:7	unclassified	-0.0465	0.0137	6.74E-04
11q14.1	DLG2	rs17148090	PC O 42:5 / PC 38:6	unclassified	-0.0010	0.0003	1.60E-03
11q14.1	DLG2	rs17148090	PC O 42:5 / PC 38:5	unclassified	-0.0015	0.0004	4.28E-04
11q14.1	DLG2	rs17148090	PC O 42:5 / PC 38:4	unclassified	-0.0010	0.0002	2.00E-05
11q14.1	DLG2	rs17148090	PC O 42:5 / PC 38:3	unclassified	-0.0028	0.0007	1.39E-04
11q14.1	DLG2	rs17148090	PC O 42:5 / PC 38:2	unclassified	-0.0032	0.0195	8.68E-01
11q14.1	DLG2	rs17148090	PC O 42:5 / PC 38:1	unclassified	-0.0009	0.0066	8.88E-01
11q14.1	DLG2	rs17148090	PC O 42:5 / PC 38:0	unclassified	-0.0075	0.0052	1.50E-01
11q14.1	DLG2	rs17148090	PC O 42:5 / PC O 40:6	unclassified	-0.0114	0.0053	3.23E-02
11q14.1	DLG2	rs17148090	PC O 42:5 / PC O 40:4	unclassified	-0.0303	0.0091	8.13E-04
11q14.1	DLG2	rs17148090	PC O 42:5 / PC 40:7	unclassified	-0.0125	0.0040	1.84E-03
11q14.1	DLG2	rs17148090	PC O 42:5 / PC 40:6	unclassified	-0.0038	0.0011	4.32E-04
11q14.1	DLG2	rs17148090	PC O 42:5 / PC 40:5	unclassified	-0.0086	0.0027	1.54E-03
11q14.1	DLG2	rs17148090	PC O 42:5 / PC 40:4	unclassified	-0.0182	0.0076	1.68E-02
11q14.1	DLG2	rs17148090	PC O 42:5 / PC 40:3	unclassified	-0.0071	0.0179	6.93E-01
11q14.1	DLG2	rs17148090	PC O 42:5 / PC 40:2	unclassified	-0.0368	0.0234	1.16E-01
11q14.1	DLG2	rs17148090	PC O 42:5 / PC 40:1	unclassified	-0.0033	0.0139	8.15E-01
11q14.1	DLG2	rs17148090	PC O 42:5 / PC 40:0	unclassified	-0.0551	0.0260	3.42E-02
11q14.1	DLG2	rs17148090	PC O 42:5 / PC 42:5	unclassified	-0.0756	0.0324	1.97E-02
11q14.1	DLG2	rs17148090	PC O 42:5 / PC 42:4	unclassified	-0.1650	0.0775	3.33E-02
12q23.1	CDK17	rs12423247	PC O 42:6 / PC 26:0	unclassified	-0.6852	0.2049	8.25E-04
12q23.1	CDK17	rs12423247	PC O 42:6 / PC 30:1	unclassified	-0.1586	0.2003	4.28E-01
12q23.1	CDK17	rs12423247	PC O 42:6 / PC 30:0	unclassified	-0.0047	0.0121	6.96E-01
12q23.1	CDK17	rs12423247	PC O 42:6 / PC O 32:1	unclassified	-0.0409	0.0132	1.94E-03
12q23.1	CDK17	rs12423247	PC O 42:6 / PC O 32:0	unclassified	-0.0365	0.0100	2.65E-04
12q23.1	CDK17	rs12423247	PC O 42:6 / PC 32:2	unclassified	-0.0175	0.0131	1.82E-01
12q23.1	CDK17	rs12423247	PC O 42:6 / PC 32:1	unclassified	-0.0048	0.0035	1.68E-01
12q23.1	CDK17	rs12423247	PC O 42:6 / PC 32:0	unclassified	-0.0085	0.0022	1.42E-04
12q23.1	CDK17	rs12423247	PC O 42:6 / PC O 34:3	unclassified	-0.0218	0.0054	5.14E-05
12q23.1	CDK17	rs12423247	PC O 42:6 / PC O 34:2	unclassified	-0.0131	0.0030	1.69E-05
12q23.1	CDK17	rs12423247	PC O 42:6 / PC O 34:1	unclassified	-0.0120	0.0034	4.52E-04
12q23.1	CDK17	rs12423247	PC O 42:6 / PC O 34:0	unclassified	-0.0126	0.0248	6.11E-01
12q23.1	CDK17	rs12423247	PC O 42:6 / PC 34:4	unclassified	-0.0348	0.0343	3.10E-01
12q23.1	CDK17	rs12423247	PC O 42:6 / PC 34:3	unclassified	-0.0092	0.0027	5.21E-04
12q23.1	CDK17	rs12423247	PC O 42:6 / PC 34:2	unclassified	-0.0004	0.0001	2.25E-07
12q23.1	CDK17	rs12423247	PC O 42:6 / PC 34:1	unclassified	-0.0006	0.0002	9.21E-04
12q23.1	CDK17	rs12423247	PC O 42:6 / PC 34:0	unclassified	-0.0164	0.0140	2.41E-01
12q23.1	CDK17	rs12423247	PC O 42:6 / PC O 36:5	unclassified	-0.0099	0.0032	1.86E-03

12q23.1	CDK17	rs12423247	PC O 42:6 / PC O 36:4	unclassified	-0.0094	0.0021	6.21E-06
12q23.1	CDK17	rs12423247	PC O 42:6 / PC O 36:3	unclassified	-0.0196	0.0046	1.79E-05
12q23.1	CDK17	rs12423247	PC O 42:6 / PC O 36:2	unclassified	-0.0096	0.0025	9.76E-05
12q23.1	CDK17	rs12423247	PC O 42:6 / PC O 36:1	unclassified	-0.0097	0.0040	1.45E-02
12q23.1	CDK17	rs12423247	PC O 42:6 / PC O 36:0	unclassified	-0.0011	0.0692	9.87E-01
12q23.1	CDK17	rs12423247	PC O 42:6 / PC 36:5	unclassified	-0.0014	0.0020	5.01E-01
12q23.1	CDK17	rs12423247	PC O 42:6 / PC 36:4	unclassified	-0.0008	0.0002	1.53E-04
12q23.1	CDK17	rs12423247	PC O 42:6 / PC 36:3	unclassified	-0.0012	0.0003	1.15E-05
12q23.1	CDK17	rs12423247	PC O 42:6 / PC 36:2	unclassified	-0.0007	0.0001	8.36E-08
12q23.1	CDK17	rs12423247	PC O 42:6 / PC 36:1	unclassified	-0.0035	0.0010	2.19E-04
12q23.1	CDK17	rs12423247	PC O 42:6 / PC 36:0	unclassified	-0.0205	0.0187	2.73E-01
12q23.1	CDK17	rs12423247	PC O 42:6 / PC O 38:5	unclassified	-0.0081	0.0018	9.80E-06
12q23.1	CDK17	rs12423247	PC O 42:6 / PC O 38:4	unclassified	-0.0096	0.0026	2.38E-04
12q23.1	CDK17	rs12423247	PC O 42:6 / PC O 38:3	unclassified	-0.0210	0.0083	1.11E-02
12q23.1	CDK17	rs12423247	PC O 42:6 / PC O 38:2	unclassified	-0.0988	0.0511	5.30E-02
12q23.1	CDK17	rs12423247	PC O 42:6 / PC O 38:1	unclassified	0.0082	0.1663	9.61E-01
12q23.1	CDK17	rs12423247	PC O 42:6 / PC 38:7	unclassified	-0.0084	0.0174	6.28E-01
12q23.1	CDK17	rs12423247	PC O 42:6 / PC 38:6	unclassified	-0.0006	0.0004	1.33E-01
12q23.1	CDK17	rs12423247	PC O 42:6 / PC 38:5	unclassified	-0.0016	0.0006	7.92E-03
12q23.1	CDK17	rs12423247	PC O 42:6 / PC 38:4	unclassified	-0.0010	0.0003	2.40E-03
12q23.1	CDK17	rs12423247	PC O 42:6 / PC 38:3	unclassified	-0.0023	0.0010	2.50E-02
12q23.1	CDK17	rs12423247	PC O 42:6 / PC 38:2	unclassified	-0.0088	0.0241	7.15E-01
12q23.1	CDK17	rs12423247	PC O 42:6 / PC 38:1	unclassified	-0.0034	0.0084	6.88E-01
12q23.1	CDK17	rs12423247	PC O 42:6 / PC 38:0	unclassified	-0.0099	0.0066	1.32E-01
12q23.1	CDK17	rs12423247	PC O 42:6 / PC O 40:6	unclassified	-0.0094	0.0068	1.72E-01
12q23.1	CDK17	rs12423247	PC O 42:6 / PC O 40:5	unclassified	-0.0248	0.0090	5.52E-03
12q23.1	CDK17	rs12423247	PC O 42:6 / PC O 40:4	unclassified	-0.0502	0.0144	5.14E-04
12q23.1	CDK17	rs12423247	PC O 42:6 / PC 40:7	unclassified	-0.0121	0.0053	2.18E-02
12q23.1	CDK17	rs12423247	PC O 42:6 / PC 40:6	unclassified	-0.0013	0.0014	3.61E-01
12q23.1	CDK17	rs12423247	PC O 42:6 / PC 40:5	unclassified	-0.0084	0.0037	2.44E-02
12q23.1	CDK17	rs12423247	PC O 42:6 / PC 40:4	unclassified	-0.0275	0.0106	9.46E-03
12q23.1	CDK17	rs12423247	PC O 42:6 / PC 40:3	unclassified	-0.0151	0.0231	5.14E-01
12q23.1	CDK17	rs12423247	PC O 42:6 / PC 40:2	unclassified	-0.0275	0.0318	3.87E-01
12q23.1	CDK17	rs12423247	PC O 42:6 / PC 40:1	unclassified	-0.0318	0.0190	9.38E-02
12q23.1	CDK17	rs12423247	PC O 42:6 / PC 40:0	unclassified	-0.1161	0.0391	2.98E-03
12q23.1	CDK17	rs12423247	PC O 42:6 / PC O 42:5	unclassified	-0.0747	0.0237	1.67E-03
12q23.1	CDK17	rs12423247	PC O 42:6 / PC 42:5	unclassified	0.0051	0.0436	9.07E-01
12q23.1	CDK17	rs12423247	PC O 42:6 / PC 42:4	unclassified	0.0901	0.1024	3.79E-01
13q21.2	PCDH20	rs7337573	PC 32:1 / PC 30:1	elongation	-16.2242	60.0870	7.87E-01
13q21.2	PCDH20	rs7337573	PC 32:1 / PC 34:1	elongation	-0.0759	0.0246	2.05E-03
13q21.2	PCDH20	rs7337573	PC 32:1 / PC 36:1	elongation	-0.6500	0.1840	4.12E-04

13q21.2	PCDH20	rs7337573	PC 32:1	/	PC 38:1	elongation	-4.3126	4.5079	3.39E-01
13q21.2	PCDH20	rs7337573	PC 32:1	/	PC 40:1	elongation	-19.5434	6.3659	2.14E-03
13q21.2	PCDH20	rs7337573	PC 32:1	/	PC O 32:1	oxidation	-22.7312	4.4887	4.10E-07
13q21.2	PCDH20	rs7337573	PC 32:1	/	PC 32:0	saturation	-2.6546	0.5109	2.04E-07
13q21.2	PCDH20	rs7337573	PC 32:1	/	PC 26:0	unclassified	-50.6455	30.3818	9.55E-02
13q21.2	PCDH20	rs7337573	PC 32:1	/	PC 30:0	unclassified	-8.7083	3.7446	2.00E-02
13q21.2	PCDH20	rs7337573	PC 32:1	/	PC O 34:3	unclassified	-9.1861	1.9206	1.73E-06
13q21.2	PCDH20	rs7337573	PC 32:1	/	PC 34:4	unclassified	-13.8075	5.3763	1.02E-02
13q21.2	PCDH20	rs7337573	PC 32:1	/	PC 34:3	unclassified	-1.8894	0.4304	1.13E-05
13q21.2	PCDH20	rs7337573	PC 32:1	/	PC 34:0	unclassified	-70.41	19.35	2.75E-04
13q21.2	PCDH20	rs7337573	PC 32:1	/	PC O 36:5	unclassified	-3.6737	1.0464	4.47E-04
13q21.2	PCDH20	rs7337573	PC 32:1	/	PC O 36:4	unclassified	-3.1230	0.6567	1.98E-06
13q21.2	PCDH20	rs7337573	PC 32:1	/	PC O 36:0	unclassified	-18.15	16.29	2.65E-01
13q21.2	PCDH20	rs7337573	PC 32:1	/	PC 36:5	unclassified	-1.2139	0.4212	3.95E-03
13q21.2	PCDH20	rs7337573	PC 32:1	/	PC 36:4	unclassified	-0.1750	0.0434	5.59E-05
13q21.2	PCDH20	rs7337573	PC 32:1	/	PC 36:0	unclassified	-13.40	9.93	1.77E-01
13q21.2	PCDH20	rs7337573	PC 32:1	/	PC O 38:5	unclassified	-2.95	0.67	1.08E-05
13q21.2	PCDH20	rs7337573	PC 32:1	/	PC O 38:3	unclassified	-15.06	2.80	7.90E-08
13q21.2	PCDH20	rs7337573	PC 32:1	/	PC O 38:2	unclassified	-39.42	11.98	1.01E-03
13q21.2	PCDH20	rs7337573	PC 32:1	/	PC O 38:1	unclassified	-132.48	36.23	2.56E-04
13q21.2	PCDH20	rs7337573	PC 32:1	/	PC 38:7	unclassified	-11.8776	5.6460	3.54E-02
13q21.2	PCDH20	rs7337573	PC 32:1	/	PC 38:6	unclassified	-0.3908	0.1156	7.22E-04
13q21.2	PCDH20	rs7337573	PC 32:1	/	PC 38:5	unclassified	-0.5695	0.1464	1.00E-04
13q21.2	PCDH20	rs7337573	PC 32:1	/	PC 38:4	unclassified	-0.2848	0.0816	4.85E-04
13q21.2	PCDH20	rs7337573	PC 32:1	/	PC 38:3	unclassified	-0.8829	0.1997	9.87E-06
13q21.2	PCDH20	rs7337573	PC 32:1	/	PC 38:2	unclassified	-11.8647	6.7355	7.82E-02
13q21.2	PCDH20	rs7337573	PC 32:1	/	PC 38:0	unclassified	-6.5739	2.0357	1.24E-03
13q21.2	PCDH20	rs7337573	PC 32:1	/	PC O 40:5	unclassified	-18.2214	3.7250	1.00E-06
13q21.2	PCDH20	rs7337573	PC 32:1	/	PC 40:7	unclassified	-6.8560	1.5539	1.02E-05
13q21.2	PCDH20	rs7337573	PC 32:1	/	PC 40:6	unclassified	-0.9649	0.3405	4.61E-03
13q21.2	PCDH20	rs7337573	PC 32:1	/	PC 40:5	unclassified	-2.7408	0.8555	1.36E-03
13q21.2	PCDH20	rs7337573	PC 32:1	/	PC 40:4	unclassified	-5.1475	2.2526	2.23E-02
13q21.2	PCDH20	rs7337573	PC 32:1	/	PC 40:3	unclassified	-15.1946	6.6836	2.30E-02
13q21.2	PCDH20	rs7337573	PC 32:1	/	PC 40:2	unclassified	-38.7784	8.6624	7.58E-06
13q21.2	PCDH20	rs7337573	PC 32:1	/	PC 40:0	unclassified	-20.2498	9.2188	2.81E-02
13q21.2	PCDH20	rs7337573	PC 32:1	/	PC O 42:6	unclassified	-41.5929	13.4535	1.99E-03
13q21.2	PCDH20	rs7337573	PC 32:1	/	PC O 42:5	unclassified	-39.4633	9.8226	5.88E-05
13q21.2	PCDH20	rs7337573	PC 32:1	/	PC 42:5	unclassified	-35.9763	11.0778	1.16E-03
13q21.2	PCDH20	rs7337573	PC 32:1	/	PC 42:4	unclassified	-33.3531	22.4458	1.37E-01
13q21.2	PCDH20	rs7337573	PC 32:1	/	PC 32:2	saturation	-16.7985	2.5499	4.46E-11
13q21.2	PCDH20	rs7337573	PC 32:1	/	PC O 32:0	unclassified	-28.7017	3.2735	1.82E-18

13q21.2	PCDH20	rs7337573	PC 32:1 / PC O 34:2	unclassified	-9.2892	1.1192	1.04E-16
13q21.2	PCDH20	rs7337573	PC 32:1 / PC O 34:1	unclassified	-6.5824	1.0022	5.11E-11
13q21.2	PCDH20	rs7337573	PC 32:1 / PC O 34:0	unclassified	-56.3320	9.6174	4.70E-09
13q21.2	PCDH20	rs7337573	PC 32:1 / PC 34:2	unclassified	-0.1211	0.0187	9.57E-11
13q21.2	PCDH20	rs7337573	PC 32:1 / PC O 36:3	unclassified	-8.8210	1.5119	5.40E-09
13q21.2	PCDH20	rs7337573	PC 32:1 / PC O 36:2	unclassified	-7.7711	0.9565	4.50E-16
13q21.2	PCDH20	rs7337573	PC 32:1 / PC O 36:1	unclassified	-7.8715	1.2481	2.85E-10
13q21.2	PCDH20	rs7337573	PC 32:1 / PC 36:3	unclassified	-0.3715	0.0583	1.82E-10
13q21.2	PCDH20	rs7337573	PC 32:1 / PC 36:2	unclassified	-0.2217	0.0377	4.00E-09
13q21.2	PCDH20	rs7337573	PC 32:1 / PC O 38:4	unclassified	-6.2303	0.9935	3.59E-10
13q21.2	PCDH20	rs7337573	PC 32:1 / PC O 40:6	unclassified	-19.3170	2.8979	2.63E-11
13q21.2	PCDH20	rs7337573	PC 32:1 / PC O 40:4	unclassified	-30.6446	5.3823	1.24E-08
13q22.1	KLF12	rs17718828	PC O 42:5 / PC 26:0	unclassified	0.0715	0.1325	5.89E-01
13q22.1	KLF12	rs17718828	PC O 42:5 / PC 30:1	unclassified	0.0619	0.1195	6.04E-01
13q22.1	KLF12	rs17718828	PC O 42:5 / PC 30:0	unclassified	0.0240	0.0069	5.40E-04
13q22.1	KLF12	rs17718828	PC O 42:5 / PC O 32:1	unclassified	0.0227	0.0076	2.63E-03
13q22.1	KLF12	rs17718828	PC O 42:5 / PC O 32:0	unclassified	0.0212	0.0055	1.26E-04
13q22.1	KLF12	rs17718828	PC O 42:5 / PC 32:2	unclassified	0.0174	0.0076	2.26E-02
13q22.1	KLF12	rs17718828	PC O 42:5 / PC 32:1	unclassified	0.0055	0.0020	6.33E-03
13q22.1	KLF12	rs17718828	PC O 42:5 / PC 32:0	unclassified	0.0063	0.0013	6.75E-07
13q22.1	KLF12	rs17718828	PC O 42:5 / PC O 34:3	unclassified	0.0046	0.0032	1.48E-01
13q22.1	KLF12	rs17718828	PC O 42:5 / PC O 34:2	unclassified	0.0053	0.0018	2.68E-03
13q22.1	KLF12	rs17718828	PC O 42:5 / PC O 34:1	unclassified	0.0076	0.0018	3.46E-05
13q22.1	KLF12	rs17718828	PC O 42:5 / PC O 34:0	unclassified	0.0519	0.0147	4.32E-04
13q22.1	KLF12	rs17718828	PC O 42:5 / PC 34:4	unclassified	0.0490	0.0199	1.40E-02
13q22.1	KLF12	rs17718828	PC O 42:5 / PC 34:3	unclassified	0.0044	0.0016	5.16E-03
13q22.1	KLF12	rs17718828	PC O 42:5 / PC 34:2	unclassified	0.0002	0.0000	5.42E-05
13q22.1	KLF12	rs17718828	PC O 42:5 / PC 34:1	unclassified	0.0004	0.0001	1.71E-04
13q22.1	KLF12	rs17718828	PC O 42:5 / PC 34:0	unclassified	0.0156	0.0087	7.19E-02
13q22.1	KLF12	rs17718828	PC O 42:5 / PC O 36:5	unclassified	0.0054	0.0019	5.11E-03
13q22.1	KLF12	rs17718828	PC O 42:5 / PC O 36:4	unclassified	0.0043	0.0012	2.30E-04
13q22.1	KLF12	rs17718828	PC O 42:5 / PC O 36:3	unclassified	0.0078	0.0026	3.03E-03
13q22.1	KLF12	rs17718828	PC O 42:5 / PC O 36:2	unclassified	0.0049	0.0014	5.86E-04
13q22.1	KLF12	rs17718828	PC O 42:5 / PC O 36:1	unclassified	0.0089	0.0022	5.77E-05
13q22.1	KLF12	rs17718828	PC O 42:5 / PC O 36:0	unclassified	0.1032	0.0425	1.52E-02
13q22.1	KLF12	rs17718828	PC O 42:5 / PC 36:5	unclassified	0.0051	0.0014	2.33E-04
13q22.1	KLF12	rs17718828	PC O 42:5 / PC 36:4	unclassified	0.0005	0.0001	1.00E-05
13q22.1	KLF12	rs17718828	PC O 42:5 / PC 36:3	unclassified	0.0005	0.0002	3.94E-04
13q22.1	KLF12	rs17718828	PC O 42:5 / PC 36:2	unclassified	0.0003	0.0001	9.77E-04
13q22.1	KLF12	rs17718828	PC O 42:5 / PC 36:1	unclassified	0.0017	0.0006	2.29E-03
13q22.1	KLF12	rs17718828	PC O 42:5 / PC 36:0	unclassified	0.0140	0.0111	2.06E-01

13q22.1	KLF12	rs17718828	PC O 42:5 / PC O 38:5	unclassified	0.0040	0.0011	1.92E-04
13q22.1	KLF12	rs17718828	PC O 42:5 / PC O 38:4	unclassified	0.0059	0.0014	2.73E-05
13q22.1	KLF12	rs17718828	PC O 42:5 / PC O 38:3	unclassified	0.0172	0.0045	1.21E-04
13q22.1	KLF12	rs17718828	PC O 42:5 / PC O 38:2	unclassified	0.0007	0.0300	9.80E-01
13q22.1	KLF12	rs17718828	PC O 42:5 / PC O 38:1	unclassified	0.0220	0.0962	8.19E-01
13q22.1	KLF12	rs17718828	PC O 42:5 / PC 38:7	unclassified	0.0406	0.0117	5.05E-04
13q22.1	KLF12	rs17718828	PC O 42:5 / PC 38:6	unclassified	0.0013	0.0003	2.20E-06
13q22.1	KLF12	rs17718828	PC O 42:5 / PC 38:5	unclassified	0.0014	0.0004	5.78E-05
13q22.1	KLF12	rs17718828	PC O 42:5 / PC 38:4	unclassified	0.0008	0.0002	5.80E-05
13q22.1	KLF12	rs17718828	PC O 42:5 / PC 38:3	unclassified	0.0021	0.0006	3.69E-04
13q22.1	KLF12	rs17718828	PC O 42:5 / PC 38:2	unclassified	0.0440	0.0164	7.26E-03
13q22.1	KLF12	rs17718828	PC O 42:5 / PC 38:1	unclassified	0.0126	0.0049	1.01E-02
13q22.1	KLF12	rs17718828	PC O 42:5 / PC 38:0	unclassified	0.0150	0.0042	3.83E-04
13q22.1	KLF12	rs17718828	PC O 42:5 / PC O 40:6	unclassified	0.0169	0.0044	1.24E-04
13q22.1	KLF12	rs17718828	PC O 42:5 / PC O 40:5	unclassified	0.0180	0.0049	2.03E-04
13q22.1	KLF12	rs17718828	PC O 42:5 / PC O 40:4	unclassified	0.0252	0.0074	6.77E-04
13q22.1	KLF12	rs17718828	PC O 42:5 / PC 40:7	unclassified	0.0116	0.0034	5.33E-04
13q22.1	KLF12	rs17718828	PC O 42:5 / PC 40:6	unclassified	0.0041	0.0009	6.15E-06
13q22.1	KLF12	rs17718828	PC O 42:5 / PC 40:5	unclassified	0.0096	0.0023	2.44E-05
13q22.1	KLF12	rs17718828	PC O 42:5 / PC 40:4	unclassified	0.0183	0.0062	3.30E-03
13q22.1	KLF12	rs17718828	PC O 42:5 / PC 40:3	unclassified	0.0290	0.0139	3.64E-02
13q22.1	KLF12	rs17718828	PC O 42:5 / PC 40:2	unclassified	0.0623	0.0180	5.27E-04
13q22.1	KLF12	rs17718828	PC O 42:5 / PC 40:1	unclassified	0.0205	0.0104	4.94E-02
13q22.1	KLF12	rs17718828	PC O 42:5 / PC 40:0	unclassified	0.0500	0.0216	2.05E-02
13q22.1	KLF12	rs17718828	PC O 42:5 / PC O 42:6	unclassified	0.0252	0.0191	1.88E-01
13q22.1	KLF12	rs17718828	PC O 42:5 / PC 42:5	unclassified	0.0778	0.0278	5.17E-03
13q22.1	KLF12	rs17718828	PC O 42:5 / PC 42:4	unclassified	0.1209	0.0641	5.93E-02
16p13.11	PDXDC1	rs4500751	LPC 20:3 / LPC 18:3	elongation	-0.1953	0.0345	1.44E-08
16p13.11	PDXDC1	rs4500751	PC 34:2 / PC 32:2	elongation	-0.7693	1.1987	5.21E-01
16p13.11	PDXDC1	rs4500751	PC 34:2 / PC 36:2	elongation	0.0124	0.0065	5.52E-02
16p13.11	PDXDC1	rs4500751	PC 34:2 / PC 38:2	elongation	-2.6762	2.9079	3.57E-01
16p13.11	PDXDC1	rs4500751	PC 34:2 / PC 40:2	elongation	7.6720	3.8020	4.36E-02
16p13.11	PDXDC1	rs4500751	PC 36:3 / PC 34:3	elongation	-0.2870	0.0400	7.40E-13
16p13.11	PDXDC1	rs4500751	PC 36:3 / PC 38:3	elongation	0.0965	0.0183	1.26E-07
16p13.11	PDXDC1	rs4500751	PC 36:3 / PC 40:3	elongation	-0.6588	0.8305	4.28E-01
16p13.11	PDXDC1	rs4500751	PC 38:3 / PC 34:3	elongation	-0.1746	0.0206	2.39E-17
16p13.11	PDXDC1	rs4500751	PC 38:3 / PC 36:3	elongation	-0.0095	0.0016	6.63E-09
16p13.11	PDXDC1	rs4500751	PC 38:3 / PC 40:3	elongation	-0.6666	0.2744	1.51E-02
16p13.11	PDXDC1	rs4500751	PE 38:3 / PE 34:3	elongation	-0.0977	0.0148	3.83E-11
16p13.11	PDXDC1	rs4500751	PE 38:3 / PE 36:3	elongation	-0.0223	0.0029	2.86E-14
16p13.11	PDXDC1	rs4500751	PE 38:3 / PE 40:3	elongation	-0.5886	0.2297	1.04E-02

16p13.11	PDXDC1	rs4500751	LPC 20:3	/	LPC 18:3	elongation	-1.9E-01	3.5E-02	1.44E-08
16p13.11	PDXDC1	rs4500751	PC 36:3	/	PC O 36:3	oxidation	-0.4933	0.1147	1.70E-05
16p13.11	PDXDC1	rs4500751	LPC 20:3	/	LPC 20:5	saturation	-0.1309	0.0338	1.08E-04
16p13.11	PDXDC1	rs4500751	LPC 20:3	/	LPC 20:4	saturation	-0.0121	0.0024	4.55E-07
16p13.11	PDXDC1	rs4500751	LPC 20:3	/	LPC 20:0	saturation	-0.4076	0.0642	2.11E-10
16p13.11	PDXDC1	rs4500751	PC 34:2	/	PC 34:4	saturation	4.2067	3.4559	2.24E-01
16p13.11	PDXDC1	rs4500751	PC 34:2	/	PC 34:3	saturation	0.1812	0.1595	2.56E-01
16p13.11	PDXDC1	rs4500751	PC 34:2	/	PC 34:1	saturation	0.0518	0.0124	3.12E-05
16p13.11	PDXDC1	rs4500751	PC 34:2	/	PC 34:0	saturation	2.6534	2.1982	2.27E-01
16p13.11	PDXDC1	rs4500751	PC 36:3	/	PC 36:5	saturation	-0.0894	0.0667	1.80E-01
16p13.11	PDXDC1	rs4500751	PC 36:3	/	PC 36:4	saturation	-0.0051	0.0042	2.24E-01
16p13.11	PDXDC1	rs4500751	PC 36:3	/	PC 36:2	saturation	-0.0183	0.0024	1.62E-14
16p13.11	PDXDC1	rs4500751	PC 36:3	/	PC 36:1	saturation	-0.0527	0.0203	9.30E-03
16p13.11	PDXDC1	rs4500751	PC 38:3	/	PC 38:7	saturation	-0.9658	0.2282	2.32E-05
16p13.11	PDXDC1	rs4500751	PC 38:3	/	PC 38:6	saturation	-0.0205	0.0048	1.90E-05
16p13.11	PDXDC1	rs4500751	PC 38:3	/	PC 38:5	saturation	-0.0325	0.0053	8.95E-10
16p13.11	PDXDC1	rs4500751	PC 38:3	/	PC 38:4	saturation	-0.0113	0.0025	4.45E-06
16p13.11	PDXDC1	rs4500751	PC 38:3	/	PC 38:2	saturation	-0.7037	0.2577	6.33E-03
16p13.11	PDXDC1	rs4500751	PC 38:3	/	PC 38:1	saturation	-0.3847	0.1168	9.90E-04
16p13.11	PDXDC1	rs4500751	PC 38:3	/	PC 38:0	saturation	-0.3273	0.0842	1.02E-04
16p13.11	PDXDC1	rs4500751	LPC 20:3		LPC 20:4	saturation	-1.2E-02	2.40E-03	4.55E-07
16p13.11	PDXDC1	rs4500751	LPC 20:3		LPC 20:0	saturation	-4.1E-01	6.42E-02	2.11E-10
16p13.11	PDXDC1	rs4500751	LPC 20:3	/	LPC 15:0	unclassified	-0.0987	0.0155	2.03E-10
16p13.11	PDXDC1	rs4500751	LPC 20:3	/	LPC 16:0	unclassified	-0.0009	0.0001	5.78E-17
16p13.11	PDXDC1	rs4500751	LPC 20:3	/	LPC 18:2	unclassified	-0.0059	0.0007	2.22E-18
16p13.11	PDXDC1	rs4500751	LPC 20:3	/	LPC 18:1	unclassified	-0.0059	0.0007	5.95E-16
16p13.11	PDXDC1	rs4500751	LPC 20:3	/	LPC 18:0	unclassified	-0.0026	0.0004	4.17E-13
16p13.11	PDXDC1	rs4500751	LPC 20:3	/	LPC 22:6	unclassified	-0.0575	0.0131	1.14E-05
16p13.11	PDXDC1	rs4500751	LPC 20:3	/	LPC 22:5	unclassified	-0.1815	0.0306	3.19E-09
16p13.11	PDXDC1	rs4500751	LPC 20:3	/	LPC 22:4	unclassified	-0.2912	0.0739	8.08E-05
16p13.11	PDXDC1	rs4500751	LPC 20:3	/	LPC 22:0	unclassified	-0.1880	0.0375	5.24E-07
16p13.11	PDXDC1	rs4500751	PC 34:2	/	PC 26:0	unclassified	-14.0170	21.0477	5.05E-01
16p13.11	PDXDC1	rs4500751	PC 34:2	/	PC 30:1	unclassified	-21.8421	26.5008	4.10E-01
16p13.11	PDXDC1	rs4500751	PC 34:2	/	PC 30:0	unclassified	2.8389	1.2387	2.19E-02
16p13.11	PDXDC1	rs4500751	PC 34:2	/	PC O 32:1	unclassified	4.2301	1.5463	6.23E-03
16p13.11	PDXDC1	rs4500751	PC 34:2	/	PC O 32:0	unclassified	2.3136	1.0041	2.12E-02
16p13.11	PDXDC1	rs4500751	PC 34:2	/	PC 32:1	unclassified	0.6633	0.3301	4.45E-02
16p13.11	PDXDC1	rs4500751	PC 34:2	/	PC 32:0	unclassified	0.8089	0.1579	3.00E-07
16p13.11	PDXDC1	rs4500751	PC 34:2	/	PC O 34:3	unclassified	0.1537	0.5423	7.77E-01
16p13.11	PDXDC1	rs4500751	PC 34:2	/	PC O 34:2	unclassified	0.1039	0.2465	6.73E-01
16p13.11	PDXDC1	rs4500751	PC 34:2	/	PC O 34:1	unclassified	0.9813	0.2901	7.17E-04

16p13.11	PDXDC1	rs4500751	PC 34:2 / PC O 34:0	unclassified	9.1318	2.8826	1.54E-03
16p13.11	PDXDC1	rs4500751	PC 34:2 / PC O 36:5	unclassified	1.4076	0.3802	2.14E-04
16p13.11	PDXDC1	rs4500751	PC 34:2 / PC O 36:4	unclassified	1.0067	0.2214	5.42E-06
16p13.11	PDXDC1	rs4500751	PC 34:2 / PC O 36:3	unclassified	1.0796	0.3820	4.71E-03
16p13.11	PDXDC1	rs4500751	PC 34:2 / PC O 36:2	unclassified	0.1740	0.1988	3.82E-01
16p13.11	PDXDC1	rs4500751	PC 34:2 / PC O 36:1	unclassified	1.2118	0.3565	6.76E-04
16p13.11	PDXDC1	rs4500751	PC 34:2 / PC O 36:0	unclassified	7.0610	9.8814	4.75E-01
16p13.11	PDXDC1	rs4500751	PC 34:2 / PC 36:5	unclassified	0.4403	0.2547	8.39E-02
16p13.11	PDXDC1	rs4500751	PC 34:2 / PC 36:4	unclassified	0.0835	0.0164	3.60E-07
16p13.11	PDXDC1	rs4500751	PC 34:2 / PC 36:3	unclassified	0.1370	0.0139	6.84E-23
16p13.11	PDXDC1	rs4500751	PC 34:2 / PC 36:1	unclassified	0.2488	0.0843	3.18E-03
16p13.11	PDXDC1	rs4500751	PC 34:2 / PC 36:0	unclassified	4.5179	2.6380	8.68E-02
16p13.11	PDXDC1	rs4500751	PC 34:2 / PC O 38:5	unclassified	1.1721	0.2212	1.17E-07
16p13.11	PDXDC1	rs4500751	PC 34:2 / PC O 38:4	unclassified	1.6412	0.3051	7.51E-08
16p13.11	PDXDC1	rs4500751	PC 34:2 / PC O 38:3	unclassified	5.9179	0.8440	2.35E-12
16p13.11	PDXDC1	rs4500751	PC 34:2 / PC O 38:2	unclassified	-5.7422	6.3371	3.65E-01
16p13.11	PDXDC1	rs4500751	PC 34:2 / PC O 38:1	unclassified	2.0246	20.1514	9.20E-01
16p13.11	PDXDC1	rs4500751	PC 34:2 / PC 38:7	unclassified	4.6061	2.2269	3.86E-02
16p13.11	PDXDC1	rs4500751	PC 34:2 / PC 38:6	unclassified	0.1874	0.0460	4.56E-05
16p13.11	PDXDC1	rs4500751	PC 34:2 / PC 38:5	unclassified	0.2461	0.0640	1.19E-04
16p13.11	PDXDC1	rs4500751	PC 34:2 / PC 38:4	unclassified	0.1867	0.0352	1.15E-07
16p13.11	PDXDC1	rs4500751	PC 34:2 / PC 38:3	unclassified	0.7619	0.0973	5.00E-15
16p13.11	PDXDC1	rs4500751	PC 34:2 / PC 38:1	unclassified	0.1630	0.9832	8.68E-01
16p13.11	PDXDC1	rs4500751	PC 34:2 / PC 38:0	unclassified	2.2936	0.8205	5.18E-03
16p13.11	PDXDC1	rs4500751	PC 34:2 / PC O 40:6	unclassified	3.7555	0.9773	1.22E-04
16p13.11	PDXDC1	rs4500751	PC 34:2 / PC O 40:5	unclassified	5.7987	1.3760	2.51E-05
16p13.11	PDXDC1	rs4500751	PC 34:2 / PC O 40:4	unclassified	8.0582	2.0451	8.14E-05
16p13.11	PDXDC1	rs4500751	PC 34:2 / PC 40:7	unclassified	1.7255	0.5905	3.48E-03
16p13.11	PDXDC1	rs4500751	PC 34:2 / PC 40:6	unclassified	0.6465	0.1696	1.38E-04
16p13.11	PDXDC1	rs4500751	PC 34:2 / PC 40:5	unclassified	1.2681	0.4241	2.79E-03
16p13.11	PDXDC1	rs4500751	PC 34:2 / PC 40:4	unclassified	5.1794	1.2387	2.90E-05
16p13.11	PDXDC1	rs4500751	PC 34:2 / PC 40:3	unclassified	5.2017	2.9994	8.29E-02
16p13.11	PDXDC1	rs4500751	PC 34:2 / PC 40:1	unclassified	-0.5837	2.1982	7.91E-01
16p13.11	PDXDC1	rs4500751	PC 34:2 / PC 40:0	unclassified	13.8997	5.0887	6.30E-03
16p13.11	PDXDC1	rs4500751	PC 34:2 / PC O 42:6	unclassified	13.1865	5.5928	1.84E-02
16p13.11	PDXDC1	rs4500751	PC 34:2 / PC O 42:5	unclassified	13.1801	3.9710	9.03E-04
16p13.11	PDXDC1	rs4500751	PC 34:2 / PC 42:5	unclassified	6.3255	6.1291	3.02E-01
16p13.11	PDXDC1	rs4500751	PC 34:2 / PC 42:4	unclassified	22.9032	14.3418	1.10E-01
16p13.11	PDXDC1	rs4500751	PC 36:3 / PC 26:0	unclassified	-16.4598	5.9751	5.87E-03
16p13.11	PDXDC1	rs4500751	PC 36:3 / PC 30:1	unclassified	-14.9157	7.8391	5.71E-02
16p13.11	PDXDC1	rs4500751	PC 36:3 / PC 30:0	unclassified	-0.2856	0.3370	3.97E-01

16p13.11	PDXDC1	rs4500751	PC 36:3 / PC O 32:1	unclassified	-1.1277	0.4720	1.69E-02
16p13.11	PDXDC1	rs4500751	PC 36:3 / PC O 32:0	unclassified	-0.9458	0.3059	1.99E-03
16p13.11	PDXDC1	rs4500751	PC 36:3 / PC 32:2	unclassified	-1.5244	0.3342	5.09E-06
16p13.11	PDXDC1	rs4500751	PC 36:3 / PC 32:1	unclassified	-0.1302	0.0821	1.13E-01
16p13.11	PDXDC1	rs4500751	PC 36:3 / PC 32:0	unclassified	-0.1261	0.0477	8.27E-03
16p13.11	PDXDC1	rs4500751	PC 36:3 / PC O 34:3	unclassified	-0.8686	0.1831	2.08E-06
16p13.11	PDXDC1	rs4500751	PC 36:3 / PC O 34:2	unclassified	-0.5229	0.0870	1.88E-09
16p13.11	PDXDC1	rs4500751	PC 36:3 / PC O 34:1	unclassified	-0.2865	0.0801	3.46E-04
16p13.11	PDXDC1	rs4500751	PC 36:3 / PC O 34:0	unclassified	-0.5842	0.8200	4.76E-01
16p13.11	PDXDC1	rs4500751	PC 36:3 / PC 34:4	unclassified	-1.8623	0.8609	3.05E-02
16p13.11	PDXDC1	rs4500751	PC 36:3 / PC 34:1	unclassified	-0.0083	0.0030	6.06E-03
16p13.11	PDXDC1	rs4500751	PC 36:3 / PC 34:0	unclassified	-0.2596	0.6452	6.87E-01
16p13.11	PDXDC1	rs4500751	PC 36:3 / PC O 36:5	unclassified	-0.1813	0.1111	1.03E-01
16p13.11	PDXDC1	rs4500751	PC 36:3 / PC O 36:4	unclassified	-0.0950	0.0633	1.33E-01
16p13.11	PDXDC1	rs4500751	PC 36:3 / PC O 36:2	unclassified	-0.3756	0.0678	3.01E-08
16p13.11	PDXDC1	rs4500751	PC 36:3 / PC O 36:1	unclassified	-0.2921	0.0956	2.26E-03
16p13.11	PDXDC1	rs4500751	PC 36:3 / PC O 36:0	unclassified	-4.4086	2.6992	1.02E-01
16p13.11	PDXDC1	rs4500751	PC 36:3 / PC 36:0	unclassified	-0.8497	0.8154	2.97E-01
16p13.11	PDXDC1	rs4500751	PC 36:3 / PC O 38:5	unclassified	-0.0544	0.0631	3.89E-01
16p13.11	PDXDC1	rs4500751	PC 36:3 / PC O 38:4	unclassified	-0.0418	0.0824	6.12E-01
16p13.11	PDXDC1	rs4500751	PC 36:3 / PC O 38:3	unclassified	0.3548	0.2032	8.07E-02
16p13.11	PDXDC1	rs4500751	PC 36:3 / PC O 38:2	unclassified	-5.3578	1.8509	3.79E-03
16p13.11	PDXDC1	rs4500751	PC 36:3 / PC O 38:1	unclassified	-6.5767	5.6876	2.48E-01
16p13.11	PDXDC1	rs4500751	PC 36:3 / PC 38:7	unclassified	-1.0635	0.6287	9.07E-02
16p13.11	PDXDC1	rs4500751	PC 36:3 / PC 38:6	unclassified	-0.0105	0.0135	4.37E-01
16p13.11	PDXDC1	rs4500751	PC 36:3 / PC 38:5	unclassified	-0.0221	0.0154	1.50E-01
16p13.11	PDXDC1	rs4500751	PC 36:3 / PC 38:4	unclassified	0.0055	0.0085	5.16E-01
16p13.11	PDXDC1	rs4500751	PC 36:3 / PC 38:2	unclassified	-2.5518	1.0342	1.36E-02
16p13.11	PDXDC1	rs4500751	PC 36:3 / PC 38:1	unclassified	-0.8597	0.3178	6.82E-03
16p13.11	PDXDC1	rs4500751	PC 36:3 / PC 38:0	unclassified	-0.3238	0.2332	1.65E-01
16p13.11	PDXDC1	rs4500751	PC 36:3 / PC O 40:6	unclassified	-0.4067	0.2904	1.61E-01
16p13.11	PDXDC1	rs4500751	PC 36:3 / PC O 40:5	unclassified	-0.4244	0.3873	2.73E-01
16p13.11	PDXDC1	rs4500751	PC 36:3 / PC O 40:4	unclassified	-0.6002	0.5616	2.85E-01
16p13.11	PDXDC1	rs4500751	PC 36:3 / PC 40:7	unclassified	-0.3878	0.1562	1.31E-02
16p13.11	PDXDC1	rs4500751	PC 36:3 / PC 40:6	unclassified	-0.0064	0.0460	8.89E-01
16p13.11	PDXDC1	rs4500751	PC 36:3 / PC 40:5	unclassified	-0.1309	0.1036	2.07E-01
16p13.11	PDXDC1	rs4500751	PC 36:3 / PC 40:4	unclassified	0.0905	0.2952	7.59E-01
16p13.11	PDXDC1	rs4500751	PC 36:3 / PC 40:2	unclassified	-0.9260	1.0400	3.73E-01
16p13.11	PDXDC1	rs4500751	PC 36:3 / PC 40:1	unclassified	-2.1936	0.6963	1.63E-03
16p13.11	PDXDC1	rs4500751	PC 36:3 / PC 40:0	unclassified	-0.1676	1.4100	9.05E-01
16p13.11	PDXDC1	rs4500751	PC 36:3 / PC O 42:6	unclassified	-2.7309	1.6418	9.62E-02

16p13.11	PDXDC1	rs4500751	PC 36:3 / PC O 42:5	unclassified	-1.0558	1.1434	3.56E-01
16p13.11	PDXDC1	rs4500751	PC 36:3 / PC 42:5	unclassified	-3.2007	1.7259	6.37E-02
16p13.11	PDXDC1	rs4500751	PC 36:3 / PC 42:4	unclassified	-2.4614	4.0517	5.44E-01
16p13.11	PDXDC1	rs4500751	PC 38:3 / PC 26:0	unclassified	-8.4111	2.1290	7.79E-05
16p13.11	PDXDC1	rs4500751	PC 38:3 / PC 30:1	unclassified	-6.7508	2.5117	7.19E-03
16p13.11	PDXDC1	rs4500751	PC 38:3 / PC 30:0	unclassified	-0.3397	0.1196	4.50E-03
16p13.11	PDXDC1	rs4500751	PC 38:3 / PC O 32:1	unclassified	-0.8759	0.1841	1.96E-06
16p13.11	PDXDC1	rs4500751	PC 38:3 / PC O 32:0	unclassified	-0.6912	0.1252	3.41E-08
16p13.11	PDXDC1	rs4500751	PC 38:3 / PC 32:2	unclassified	-0.7947	0.1238	1.35E-10
16p13.11	PDXDC1	rs4500751	PC 38:3 / PC 32:1	unclassified	-0.1227	0.0257	1.90E-06
16p13.11	PDXDC1	rs4500751	PC 38:3 / PC 32:0	unclassified	-0.1326	0.0222	2.25E-09
16p13.11	PDXDC1	rs4500751	PC 38:3 / PC O 34:3	unclassified	-0.4703	0.0764	7.54E-10
16p13.11	PDXDC1	rs4500751	PC 38:3 / PC O 34:2	unclassified	-0.2806	0.0394	1.13E-12
16p13.11	PDXDC1	rs4500751	PC 38:3 / PC O 34:1	unclassified	-0.2307	0.0364	2.40E-10
16p13.11	PDXDC1	rs4500751	PC 38:3 / PC O 34:0	unclassified	-0.9688	0.3043	1.46E-03
16p13.11	PDXDC1	rs4500751	PC 38:3 / PC 34:4	unclassified	-1.3760	0.2765	6.47E-07
16p13.11	PDXDC1	rs4500751	PC 38:3 / PC 34:2	unclassified	-0.0067	0.0007	3.88E-20
16p13.11	PDXDC1	rs4500751	PC 38:3 / PC 34:1	unclassified	-0.0085	0.0013	1.15E-10
16p13.11	PDXDC1	rs4500751	PC 38:3 / PC 34:0	unclassified	-0.2406	0.2036	2.37E-01
16p13.11	PDXDC1	rs4500751	PC 38:3 / PC O 36:5	unclassified	-0.2058	0.0412	5.76E-07
16p13.11	PDXDC1	rs4500751	PC 38:3 / PC O 36:4	unclassified	-0.1233	0.0244	4.18E-07
16p13.11	PDXDC1	rs4500751	PC 38:3 / PC O 36:3	unclassified	-0.3275	0.0520	3.01E-10
16p13.11	PDXDC1	rs4500751	PC 38:3 / PC O 36:2	unclassified	-0.2158	0.0311	4.29E-12
16p13.11	PDXDC1	rs4500751	PC 38:3 / PC O 36:1	unclassified	-0.2436	0.0404	1.66E-09
16p13.11	PDXDC1	rs4500751	PC 38:3 / PC O 36:0	unclassified	-3.0133	0.8737	5.63E-04
16p13.11	PDXDC1	rs4500751	PC 38:3 / PC 36:5	unclassified	-0.0869	0.0209	3.13E-05
16p13.11	PDXDC1	rs4500751	PC 38:3 / PC 36:4	unclassified	-0.0095	0.0017	2.47E-08
16p13.11	PDXDC1	rs4500751	PC 38:3 / PC 36:2	unclassified	-0.0114	0.0012	1.81E-20
16p13.11	PDXDC1	rs4500751	PC 38:3 / PC 36:1	unclassified	-0.0478	0.0071	1.68E-11
16p13.11	PDXDC1	rs4500751	PC 38:3 / PC 36:0	unclassified	-0.7616	0.2821	6.93E-03
16p13.11	PDXDC1	rs4500751	PC 38:3 / PC O 38:5	unclassified	-0.1209	0.0249	1.16E-06
16p13.11	PDXDC1	rs4500751	PC 38:3 / PC O 38:4	unclassified	-0.1354	0.0324	2.97E-05
16p13.11	PDXDC1	rs4500751	PC 38:3 / PC O 38:3	unclassified	-0.2013	0.0787	1.05E-02
16p13.11	PDXDC1	rs4500751	PC 38:3 / PC O 38:2	unclassified	-2.9325	0.6946	2.42E-05
16p13.11	PDXDC1	rs4500751	PC 38:3 / PC O 38:1	unclassified	-4.2202	1.8662	2.37E-02
16p13.11	PDXDC1	rs4500751	PC 38:3 / PC O 40:6	unclassified	-0.4752	0.1098	1.50E-05
16p13.11	PDXDC1	rs4500751	PC 38:3 / PC O 40:5	unclassified	-0.6306	0.1426	9.80E-06
16p13.11	PDXDC1	rs4500751	PC 38:3 / PC O 40:4	unclassified	-0.9019	0.2112	1.95E-05
16p13.11	PDXDC1	rs4500751	PC 38:3 / PC 40:7	unclassified	-0.3470	0.0620	2.18E-08
16p13.11	PDXDC1	rs4500751	PC 38:3 / PC 40:6	unclassified	-0.0545	0.0130	2.75E-05
16p13.11	PDXDC1	rs4500751	PC 38:3 / PC 40:5	unclassified	-0.1636	0.0271	1.60E-09

16p13.11	PDXDC1	rs4500751	PC 38:3	/ PC 40:4	unclassified	-0.3343	0.0827	5.29E-05
16p13.11	PDXDC1	rs4500751	PC 38:3	/ PC 40:2	unclassified	-0.9296	0.3544	8.72E-03
16p13.11	PDXDC1	rs4500751	PC 38:3	/ PC 40:1	unclassified	-0.9966	0.2425	3.96E-05
16p13.11	PDXDC1	rs4500751	PC 38:3	/ PC 40:0	unclassified	-1.1215	0.4663	1.62E-02
16p13.11	PDXDC1	rs4500751	PC 38:3	/ PC O 42:6	unclassified	-2.3465	0.5745	4.42E-05
16p13.11	PDXDC1	rs4500751	PC 38:3	/ PC O 42:5	unclassified	-1.4771	0.4133	3.52E-04
16p13.11	PDXDC1	rs4500751	PC 38:3	/ PC 42:5	unclassified	-2.2158	0.5454	4.85E-05
16p13.11	PDXDC1	rs4500751	PC 38:3	/ PC 42:4	unclassified	-2.9028	1.2988	2.54E-02
16p13.11	PDXDC1	rs4500751	PE 38:3	/ PE 32:2	unclassified	-0.1656	0.0585	4.68E-03
16p13.11	PDXDC1	rs4500751	PE 38:3	/ PE 32:1	unclassified	-0.0813	0.0209	1.03E-04
16p13.11	PDXDC1	rs4500751	PE 38:3	/ PE 32:0	unclassified	-0.1535	0.0594	9.73E-03
16p13.11	PDXDC1	rs4500751	PE 38:3	/ PE 34:2	unclassified	-0.0074	0.0010	1.38E-14
16p13.11	PDXDC1	rs4500751	PE 38:3	/ PE 34:1	unclassified	-0.0089	0.0019	5.02E-06
16p13.11	PDXDC1	rs4500751	PE 38:3	/ PE 34:0	unclassified	-0.1423	0.0762	6.18E-02
16p13.11	PDXDC1	rs4500751	PE 38:3	/ PE 36:5	unclassified	-0.0834	0.0195	1.84E-05
16p13.11	PDXDC1	rs4500751	PE 38:3	/ PE 36:4	unclassified	-0.0070	0.0018	1.03E-04
16p13.11	PDXDC1	rs4500751	PE 38:3	/ PE 36:2	unclassified	-0.0060	0.0007	3.35E-17
16p13.11	PDXDC1	rs4500751	PE 38:3	/ PE 36:1	unclassified	-0.0239	0.0044	6.46E-08
16p13.11	PDXDC1	rs4500751	PE 38:3	/ PE O 38:7	unclassified	-0.1199	0.0741	1.05E-01
16p13.11	PDXDC1	rs4500751	PE 38:3	/ PE 38:6	unclassified	-0.0073	0.0020	2.21E-04
16p13.11	PDXDC1	rs4500751	PE 38:3	/ PE 38:5	unclassified	-0.0153	0.0029	1.04E-07
16p13.11	PDXDC1	rs4500751	PE 38:3	/ PE 38:4	unclassified	-0.0030	0.0010	2.17E-03
16p13.11	PDXDC1	rs4500751	PE 38:3	/ PE 38:2	unclassified	-0.1653	0.0435	1.44E-04
16p13.11	PDXDC1	rs4500751	PE 38:3	/ PE 38:1	unclassified	-0.0914	0.0395	2.07E-02
16p13.11	PDXDC1	rs4500751	PE 38:3	/ PE O 40:3	unclassified	-0.0371	0.0072	3.06E-07
16p13.11	PDXDC1	rs4500751	PE 38:3	/ PE 40:6	unclassified	-0.0096	0.0032	2.61E-03
16p13.11	PDXDC1	rs4500751	PE 38:3	/ PE 40:5	unclassified	-0.0416	0.0096	1.50E-05
16p13.11	PDXDC1	rs4500751	PE 38:3	/ PE 40:4	unclassified	-0.1199	0.0319	1.72E-04
16p13.11	PDXDC1	rs4500751	PE 38:3	/ PE 42:7	unclassified	-0.4566	0.1433	1.44E-03
16p13.11	PDXDC1	rs4500751	PE 38:3	/ PE 42:6	unclassified	-0.5943	0.2395	1.31E-02
16p13.11	PDXDC1	rs4500751	PE 38:3	/ PE 42:5	unclassified	-0.7820	0.2958	8.21E-03
16p13.11	PDXDC1	rs4500751	LPC 20:3	/ LPC 16:1	unclassified	-0.0588	0.0059	4.01E-23
16p13.11	PDXDC1	rs4500751	PC 36:3	/ PC 34:2	unclassified	-0.0119	0.0011	4.37E-25
16p13.3	ALG1	rs870288	PC 30:1	/ PC 32:1	elongation	-0.0061	0.0022	6.58E-03
16p13.3	ALG1	rs870288	PC 30:1	/ PC 34:1	elongation	-0.0006	0.0001	8.56E-05
16p13.3	ALG1	rs870288	PC 30:1	/ PC 36:1	elongation	-0.0032	0.0008	9.45E-05
16p13.3	ALG1	rs870288	PC 30:1	/ PC 38:1	elongation	-0.0198	0.0064	2.13E-03
16p13.3	ALG1	rs870288	PC 30:1	/ PC 40:1	elongation	-0.0431	0.0131	1.04E-03
16p13.3	ALG1	rs870288	PC 30:1	/ PC 26:0	unclassified	-0.1847	0.1012	6.80E-02
16p13.3	ALG1	rs870288	PC 30:1	/ PC 30:0	unclassified	-0.0206	0.0092	2.61E-02
16p13.3	ALG1	rs870288	PC 30:1	/ PC O 32:1	unclassified	-0.0504	0.0129	9.22E-05

16p13.3	ALG1	rs870288	PC 30:1 / PC O 32:0	unclassified	-0.0421	0.0098	1.64E-05
16p13.3	ALG1	rs870288	PC 30:1 / PC 32:2	unclassified	-0.0342	0.0107	1.36E-03
16p13.3	ALG1	rs870288	PC 30:1 / PC 32:0	unclassified	-0.0088	0.0021	2.94E-05
16p13.3	ALG1	rs870288	PC 30:1 / PC O 34:3	unclassified	-0.0185	0.0050	1.97E-04
16p13.3	ALG1	rs870288	PC 30:1 / PC O 34:2	unclassified	-0.0121	0.0030	6.20E-05
16p13.3	ALG1	rs870288	PC 30:1 / PC O 34:1	unclassified	-0.0147	0.0033	9.72E-06
16p13.3	ALG1	rs870288	PC 30:1 / PC O 34:0	unclassified	-0.0672	0.0229	3.38E-03
16p13.3	ALG1	rs870288	PC 30:1 / PC 34:4	unclassified	-0.0533	0.0241	2.69E-02
16p13.3	ALG1	rs870288	PC 30:1 / PC 34:3	unclassified	-0.0062	0.0021	2.87E-03
16p13.3	ALG1	rs870288	PC 30:1 / PC 34:2	unclassified	-0.0003	0.0001	8.69E-06
16p13.3	ALG1	rs870288	PC 30:1 / PC 34:0	unclassified	-0.0281	0.0123	2.23E-02
16p13.3	ALG1	rs870288	PC 30:1 / PC O 36:5	unclassified	-0.0121	0.0033	2.03E-04
16p13.3	ALG1	rs870288	PC 30:1 / PC O 36:4	unclassified	-0.0094	0.0022	1.31E-05
16p13.3	ALG1	rs870288	PC 30:1 / PC O 36:3	unclassified	-0.0171	0.0045	1.54E-04
16p13.3	ALG1	rs870288	PC 30:1 / PC O 36:2	unclassified	-0.0103	0.0024	2.20E-05
16p13.3	ALG1	rs870288	PC 30:1 / PC O 36:1	unclassified	-0.0152	0.0037	4.26E-05
16p13.3	ALG1	rs870288	PC 30:1 / PC O 36:0	unclassified	-0.1477	0.0447	9.59E-04
16p13.3	ALG1	rs870288	PC 30:1 / PC 36:5	unclassified	-0.0039	0.0016	1.77E-02
16p13.3	ALG1	rs870288	PC 30:1 / PC 36:4	unclassified	-0.0008	0.0002	1.21E-05
16p13.3	ALG1	rs870288	PC 30:1 / PC 36:3	unclassified	-0.0010	0.0002	4.97E-05
16p13.3	ALG1	rs870288	PC 30:1 / PC 36:2	unclassified	-0.0006	0.0001	8.77E-06
16p13.3	ALG1	rs870288	PC 30:1 / PC 36:0	unclassified	-0.0429	0.0133	1.25E-03
16p13.3	ALG1	rs870288	PC 30:1 / PC O 38:5	unclassified	-0.0092	0.0021	1.22E-05
16p13.3	ALG1	rs870288	PC 30:1 / PC O 38:4	unclassified	-0.0127	0.0029	1.24E-05
16p13.3	ALG1	rs870288	PC 30:1 / PC O 38:3	unclassified	-0.0302	0.0079	1.29E-04
16p13.3	ALG1	rs870288	PC 30:1 / PC O 38:2	unclassified	-0.0351	0.0277	2.04E-01
16p13.3	ALG1	rs870288	PC 30:1 / PC O 38:1	unclassified	-0.2107	0.0774	6.49E-03
16p13.3	ALG1	rs870288	PC 30:1 / PC 38:7	unclassified	-0.0419	0.0151	5.54E-03
16p13.3	ALG1	rs870288	PC 30:1 / PC 38:6	unclassified	-0.0015	0.0004	8.48E-05
16p13.3	ALG1	rs870288	PC 30:1 / PC 38:5	unclassified	-0.0021	0.0006	2.93E-04
16p13.3	ALG1	rs870288	PC 30:1 / PC 38:4	unclassified	-0.0013	0.0003	9.32E-06
16p13.3	ALG1	rs870288	PC 30:1 / PC 38:3	unclassified	-0.0034	0.0009	7.58E-05
16p13.3	ALG1	rs870288	PC 30:1 / PC 38:2	unclassified	-0.0284	0.0105	6.83E-03
16p13.3	ALG1	rs870288	PC 30:1 / PC 38:0	unclassified	-0.0198	0.0059	8.11E-04
16p13.3	ALG1	rs870288	PC 30:1 / PC O 40:6	unclassified	-0.0352	0.0080	1.20E-05
16p13.3	ALG1	rs870288	PC 30:1 / PC O 40:5	unclassified	-0.0462	0.0107	1.46E-05
16p13.3	ALG1	rs870288	PC 30:1 / PC O 40:4	unclassified	-0.0748	0.0163	4.27E-06
16p13.3	ALG1	rs870288	PC 30:1 / PC 40:7	unclassified	-0.0193	0.0050	1.07E-04
16p13.3	ALG1	rs870288	PC 30:1 / PC 40:6	unclassified	-0.0051	0.0012	2.59E-05
16p13.3	ALG1	rs870288	PC 30:1 / PC 40:5	unclassified	-0.0135	0.0035	9.14E-05
16p13.3	ALG1	rs870288	PC 30:1 / PC 40:4	unclassified	-0.0387	0.0097	7.03E-05

16p13.3	ALG1	rs870288	PC 30:1	/	PC 40:3	unclassified	-0.0529	0.0181	3.50E-03
16p13.3	ALG1	rs870288	PC 30:1	/	PC 40:2	unclassified	-0.0697	0.0207	7.51E-04
16p13.3	ALG1	rs870288	PC 30:1	/	PC 40:0	unclassified	-0.0909	0.0273	8.71E-04
16p13.3	ALG1	rs870288	PC 30:1	/	PC O 42:6	unclassified	-0.1482	0.0327	5.92E-06
16p13.3	ALG1	rs870288	PC 30:1	/	PC O 42:5	unclassified	-0.1062	0.0261	4.68E-05
16p13.3	ALG1	rs870288	PC 30:1	/	PC 42:5	unclassified	-0.0994	0.0275	3.04E-04
16p13.3	ALG1	rs870288	PC 30:1	/	PC 42:4	unclassified	-0.1841	0.0645	4.31E-03
16q23.1	CNTNAP4	rs4485401	Glu-CER 24:1	/	CER 24:1	glucosylation	0.0112	0.0032	4.45E-04
16q23.1	CNTNAP4	rs4485401	Glu-CER 24:1	/	CER 16:0	unclassified	0.0178	0.0050	3.72E-04
16q23.1	CNTNAP4	rs4485401	Glu-CER 24:1	/	CER 18:0	unclassified	0.1025	0.0253	5.16E-05
16q23.1	CNTNAP4	rs4485401	Glu-CER 24:1	/	CER 20:0	unclassified	0.1089	0.0275	7.40E-05
16q23.1	CNTNAP4	rs4485401	Glu-CER 24:1	/	CER 22:0	unclassified	0.0244	0.0051	1.47E-06
16q23.1	CNTNAP4	rs4485401	Glu-CER 24:1	/	CER 23:0	unclassified	0.0251	0.0060	2.87E-05
16q23.1	CNTNAP4	rs4485401	Glu-CER 24:1	/	CER 24:0	unclassified	0.0088	0.0019	2.29E-06
16q23.1	CNTNAP4	rs4485401	Glu-CER 24:1	/	Glu-CER16:0	unclassified	0.0210	0.0063	8.94E-04
17p13.2	PLD2	rs12051548	SPM 23:0	/	SPM 14:0	elongation	-0.1013	0.0235	1.59E-05
17p13.2	PLD2	rs12051548	SPM 23:0	/	SPM 15:0	elongation	-0.1848	0.0376	8.80E-07
17p13.2	PLD2	rs12051548	SPM 23:0	/	SPM 16:0	elongation	-0.0093	0.0017	2.24E-08
17p13.2	PLD2	rs12051548	SPM 23:0	/	SPM 18:0	elongation	-0.0421	0.0093	6.22E-06
17p13.2	PLD2	rs12051548	SPM 23:0	/	SPM 20:0	elongation	0.0758	0.0536	1.57E-01
17p13.2	PLD2	rs12051548	SPM 23:0	/	SPM 24:0	elongation	-0.0144	0.0086	9.50E-02
17p13.2	PLD2	rs12051548	SPM 23:0	/	SPM 23:1	saturation	-0.0682	0.0132	2.54E-07
17p13.2	PLD2	rs12051548	SPM 23:0	/	SPM dih 16:0	unclassified	-0.0406	0.0625	5.16E-01
17p13.2	PLD2	rs12051548	SPM 23:0	/	SPM 17:0	unclassified	-0.3157	0.0633	6.14E-07
17p13.2	PLD2	rs12051548	SPM 23:0	/	SPM 18:2	unclassified	-1.6595	0.5427	2.23E-03
17p13.2	PLD2	rs12051548	SPM 23:0	/	SPM 18:1	unclassified	-0.1148	0.0211	5.04E-08
17p13.2	PLD2	rs12051548	SPM 23:0	/	SPM dih 18:0	unclassified	-0.3372	1.5948	8.33E-01
17p13.2	PLD2	rs12051548	SPM 23:0	/	SPM 20:1	unclassified	-0.1154	0.0406	4.45E-03
17p13.2	PLD2	rs12051548	SPM 23:0	/	SPM 22:2	unclassified	1.0854	1.4841	4.65E-01
17p13.2	PLD2	rs12051548	SPM 23:0	/	SPM 22:1	unclassified	-0.0376	0.0079	2.13E-06
17p13.2	PLD2	rs12051548	SPM 23:0	/	SPM 22:0	unclassified	-0.0066	0.0061	2.83E-01
17p13.2	PLD2	rs12051548	SPM 23:0	/	SPM dih 22:0	unclassified	0.3693	2.2341	8.69E-01
17p13.2	PLD2	rs12051548	SPM 23:0	/	SPM dih 23:0	unclassified	-2.5432	2.4830	3.06E-01
17p13.2	PLD2	rs12051548	SPM 23:0	/	SPM 24:3	unclassified	-1.1067	0.5732	5.35E-02
17p13.2	PLD2	rs12051548	SPM 23:0	/	SPM 24:2	unclassified	-0.0393	0.0091	1.72E-05
17p13.2	PLD2	rs12051548	SPM 23:0	/	SPM 24:1	unclassified	-0.0118	0.0033	4.44E-04
17p13.2	PLD2	rs12051548	SPM 23:0	/	SPM dih 24:0	unclassified	-3.4499	2.8752	2.30E-01
17p13.2	PLD2	rs12051548	SPM 23:0	/	SPM 16:1	unclassified	-0.0788	0.0124	2.43E-10
18q11.2	ABHD3	rs11662721	PC 32:2	/	PC 34:2	elongation	0.0005	0.0001	6.99E-08
18q11.2	ABHD3	rs11662721	PC 32:2	/	PC 38:2	elongation	0.0525	0.0347	1.31E-01
18q11.2	ABHD3	rs11662721	PC 32:2	/	PC 40:2	elongation	0.0704	0.0510	1.67E-01

18q11.2	ABHD3	rs11662721	PC 32:2 / PC 32:1	saturation	0.0086	0.0035	1.32E-02
18q11.2	ABHD3	rs11662721	PC 32:2 / PC 32:0	saturation	0.0144	0.0032	6.83E-06
18q11.2	ABHD3	rs11662721	PC 32:2 / PC 26:0	unclassified	0.0881	0.2369	7.10E-01
18q11.2	ABHD3	rs11662721	PC 32:2 / PC 30:1	unclassified	-0.1862	0.3179	5.58E-01
18q11.2	ABHD3	rs11662721	PC 32:2 / PC 30:0	unclassified	0.0363	0.0129	4.85E-03
18q11.2	ABHD3	rs11662721	PC 32:2 / PC O 32:1	unclassified	0.0918	0.0242	1.47E-04
18q11.2	ABHD3	rs11662721	PC 32:2 / PC O 32:0	unclassified	0.0551	0.0162	6.55E-04
18q11.2	ABHD3	rs11662721	PC 32:2 / PC O 34:3	unclassified	0.0415	0.0092	5.79E-06
18q11.2	ABHD3	rs11662721	PC 32:2 / PC O 34:2	unclassified	0.0190	0.0047	4.49E-05
18q11.2	ABHD3	rs11662721	PC 32:2 / PC O 34:1	unclassified	0.0234	0.0052	6.95E-06
18q11.2	ABHD3	rs11662721	PC 32:2 / PC O 34:0	unclassified	0.1439	0.0415	5.21E-04
18q11.2	ABHD3	rs11662721	PC 32:2 / PC 34:4	unclassified	0.0881	0.0285	2.02E-03
18q11.2	ABHD3	rs11662721	PC 32:2 / PC 34:3	unclassified	0.0117	0.0022	1.76E-07
18q11.2	ABHD3	rs11662721	PC 32:2 / PC 34:1	unclassified	0.0010	0.0002	4.73E-06
18q11.2	ABHD3	rs11662721	PC 32:2 / PC 34:0	unclassified	0.0176	0.0350	6.15E-01
18q11.2	ABHD3	rs11662721	PC 32:2 / PC O 36:5	unclassified	0.0269	0.0063	2.29E-05
18q11.2	ABHD3	rs11662721	PC 32:2 / PC O 36:4	unclassified	0.0178	0.0039	3.95E-06
18q11.2	ABHD3	rs11662721	PC 32:2 / PC O 36:3	unclassified	0.0365	0.0072	3.62E-07
18q11.2	ABHD3	rs11662721	PC 32:2 / PC O 36:2	unclassified	0.0196	0.0039	4.87E-07
18q11.2	ABHD3	rs11662721	PC 32:2 / PC O 36:1	unclassified	0.0276	0.0061	5.40E-06
18q11.2	ABHD3	rs11662721	PC 32:2 / PC O 36:0	unclassified	0.3795	0.1227	1.98E-03
18q11.2	ABHD3	rs11662721	PC 32:2 / PC 36:5	unclassified	0.0138	0.0031	9.09E-06
18q11.2	ABHD3	rs11662721	PC 32:2 / PC 36:4	unclassified	0.0014	0.0003	7.81E-07
18q11.2	ABHD3	rs11662721	PC 32:2 / PC 36:1	unclassified	0.0065	0.0014	1.36E-06
18q11.2	ABHD3	rs11662721	PC 32:2 / PC 36:0	unclassified	0.0548	0.0373	1.42E-01
18q11.2	ABHD3	rs11662721	PC 32:2 / PC O 38:5	unclassified	0.0161	0.0040	4.55E-05
18q11.2	ABHD3	rs11662721	PC 32:2 / PC O 38:4	unclassified	0.0250	0.0054	3.67E-06
18q11.2	ABHD3	rs11662721	PC 32:2 / PC O 38:3	unclassified	0.0631	0.0131	1.53E-06
18q11.2	ABHD3	rs11662721	PC 32:2 / PC O 38:2	unclassified	0.2475	0.0726	6.56E-04
18q11.2	ABHD3	rs11662721	PC 32:2 / PC O 38:1	unclassified	0.5684	0.2311	1.39E-02
18q11.2	ABHD3	rs11662721	PC 32:2 / PC 38:7	unclassified	0.0620	0.0293	3.42E-02
18q11.2	ABHD3	rs11662721	PC 32:2 / PC 38:6	unclassified	0.0021	0.0007	2.47E-03
18q11.2	ABHD3	rs11662721	PC 32:2 / PC 38:5	unclassified	0.0052	0.0010	1.58E-07
18q11.2	ABHD3	rs11662721	PC 32:2 / PC 38:4	unclassified	0.0028	0.0006	5.25E-07
18q11.2	ABHD3	rs11662721	PC 32:2 / PC 38:1	unclassified	0.0084	0.0167	6.17E-01
18q11.2	ABHD3	rs11662721	PC 32:2 / PC 38:0	unclassified	0.0338	0.0127	7.96E-03
18q11.2	ABHD3	rs11662721	PC 32:2 / PC O 40:6	unclassified	0.0544	0.0158	5.90E-04
18q11.2	ABHD3	rs11662721	PC 32:2 / PC O 40:5	unclassified	0.0762	0.0224	6.58E-04
18q11.2	ABHD3	rs11662721	PC 32:2 / PC O 40:4	unclassified	0.1034	0.0323	1.37E-03
18q11.2	ABHD3	rs11662721	PC 32:2 / PC 40:7	unclassified	0.0370	0.0092	5.75E-05
18q11.2	ABHD3	rs11662721	PC 32:2 / PC 40:6	unclassified	0.0072	0.0023	2.02E-03

18q11.2	ABHD3	rs11662721	PC 32:2	/	PC 40:5	unclassified	0.0259	0.0061	2.29E-05
18q11.2	ABHD3	rs11662721	PC 32:2	/	PC 40:4	unclassified	0.0936	0.0179	1.81E-07
18q11.2	ABHD3	rs11662721	PC 32:2	/	PC 40:3	unclassified	0.0863	0.0407	3.41E-02
18q11.2	ABHD3	rs11662721	PC 32:2	/	PC 40:1	unclassified	0.0437	0.0332	1.88E-01
18q11.2	ABHD3	rs11662721	PC 32:2	/	PC 40:0	unclassified	0.1234	0.0670	6.55E-02
18q11.2	ABHD3	rs11662721	PC 32:2	/	PC O 42:6	unclassified	0.1441	0.0790	6.81E-02
18q11.2	ABHD3	rs11662721	PC 32:2	/	PC O 42:5	unclassified	0.2574	0.0595	1.52E-05
18q11.2	ABHD3	rs11662721	PC 32:2	/	PC 42:5	unclassified	0.0235	0.0801	7.69E-01
18q11.2	ABHD3	rs11662721	PC 32:2	/	PC 42:4	unclassified	0.2010	0.1805	2.66E-01
18q11.2	ABHD3	rs11662721	PC 32:2	/	PC 36:2	elongation	0.0011	0.0002	9.35E-10
18q11.2	ABHD3	rs11662721	PC 32:2	/	PC 36:3	unclassified	0.0018	0.0003	1.80E-09
18q11.2	ABHD3	rs11662721	PC 32:2	/	PC 38:3	unclassified	0.0075	0.0013	6.71E-09
19p13.11	LPAR	rs2304130	CER18:0	/	CER 16:0	elongation	-0.0088	0.0030	3.30E-03
19p13.11	LPAR	rs2304130	CER18:0	/	CER 20:0	elongation	-0.0213	0.0095	2.45E-02
19p13.11	LPAR	rs2304130	CER18:0	/	CER 22:0	elongation	-0.0086	0.0021	3.38E-05
19p13.11	LPAR	rs2304130	CER18:0	/	CER 23:0	elongation	-0.0113	0.0026	1.72E-05
19p13.11	LPAR	rs2304130	CER18:0	/	CER 24:0	elongation	-0.0047	0.0009	2.67E-07
19p13.11	LPAR	rs2304130	CER18:0	/	CER 24:1	unclassified	-0.0075	0.0016	4.24E-06
19p13.11	LPAR	rs2304130	CER18:0	/	Glu-CER 16:0	unclassified	-0.0072	0.0071	3.06E-01
19p13.11	LPAR	rs2304130	CER18:0	/	Glu-CER 24:1	unclassified	-0.0032	0.0062	6.12E-01
19q13.32	APOE	rs7259004	SPM 22:0	/	SPM 14:0	elongation	-0.2242	0.0526	2.06E-05
19q13.32	APOE	rs7259004	SPM 22:0	/	SPM 15:0	elongation	-0.4065	0.0949	1.85E-05
19q13.32	APOE	rs7259004	SPM 22:0	/	SPM 16:0	elongation	-0.0160	0.0034	2.88E-06
19q13.32	APOE	rs7259004	SPM 22:0	/	SPM 18:0	elongation	-0.0500	0.0209	1.67E-02
19q13.32	APOE	rs7259004	SPM 22:0	/	SPM 20:0	elongation	-0.0890	0.0584	1.27E-01
19q13.32	APOE	rs7259004	SPM 22:0	/	SPM 23:0	elongation	-0.0322	0.0256	2.07E-01
19q13.32	APOE	rs7259004	SPM 22:0	/	SPM 24:0	elongation	0.0091	0.0162	5.74E-01
19q13.32	APOE	rs7259004	SPM 24:0	/	SPM 14:0	elongation	-0.1543	0.0382	5.48E-05
19q13.32	APOE	rs7259004	SPM 24:0	/	SPM 15:0	elongation	-0.2740	0.0699	8.98E-05
19q13.32	APOE	rs7259004	SPM 24:0	/	SPM 16:0	elongation	-0.0098	0.0022	9.05E-06
19q13.32	APOE	rs7259004	SPM 24:0	/	SPM 18:0	elongation	-0.0325	0.0148	2.76E-02
19q13.32	APOE	rs7259004	SPM 24:0	/	SPM 20:0	elongation	-0.0577	0.0453	2.03E-01
19q13.32	APOE	rs7259004	SPM 24:0	/	SPM 23:0	elongation	-0.0185	0.0172	2.84E-01
19q13.32	APOE	rs7259004	SPM 22:0	/	SPM dih 22:0	hydroxlylation	2.3039	3.1022	4.58E-01
19q13.32	APOE	rs7259004	SPM 22:0	/	SPM 22:2	saturation	-2.5994	2.5108	3.01E-01
19q13.32	APOE	rs7259004	SPM 22:0	/	SPM 22:1	saturation	-0.0584	0.0150	9.70E-05
19q13.32	APOE	rs7259004	SPM 24:0	/	SPM 24:3	saturation	-0.9002	0.5515	1.03E-01
19q13.32	APOE	rs7259004	SPM 24:0	/	SPM 24:2	saturation	-0.0707	0.0121	5.11E-09
19q13.32	APOE	rs7259004	SPM 24:0	/	SPM 24:1	saturation	-0.0199	0.0039	3.74E-07
19q13.32	APOE	rs7259004	SPM 22:0	/	SPM 16:1	unclassified	-0.1415	0.0252	1.98E-08
19q13.32	APOE	rs7259004	SPM 22:0	/	SPM dih 16:0	unclassified	-0.0848	0.1316	5.19E-01

19q13.32	APOE	rs7259004	SPM 22:0	/	SPM 17:0	unclassified	-0.5759	0.1623	3.89E-04
19q13.32	APOE	rs7259004	SPM 22:0	/	SPM 18:2	unclassified	-2.7498	1.0348	7.88E-03
19q13.32	APOE	rs7259004	SPM 22:0	/	SPM 18:1	unclassified	-0.1628	0.0454	3.32E-04
19q13.32	APOE	rs7259004	SPM 22:0	/	SPM dih 18:0	unclassified	-1.9022	2.5833	4.62E-01
19q13.32	APOE	rs7259004	SPM 22:0	/	SPM 20:1	unclassified	-0.3422	0.0822	3.12E-05
19q13.32	APOE	rs7259004	SPM 22:0	/	SPM 23:1	unclassified	-0.1418	0.0386	2.36E-04
19q13.32	APOE	rs7259004	SPM 22:0	/	SPM dih 23:0	unclassified	-7.7533	4.1561	6.21E-02
19q13.32	APOE	rs7259004	SPM 22:0	/	SPM 24:3	unclassified	-1.3237	0.8068	1.01E-01
19q13.32	APOE	rs7259004	SPM 22:0	/	SPM 24:2	unclassified	-0.1051	0.0189	2.91E-08
19q13.32	APOE	rs7259004	SPM 22:0	/	SPM 24:1	unclassified	-0.0316	0.0069	4.52E-06
19q13.32	APOE	rs7259004	SPM 22:0	/	SPM dih 24:0	unclassified	-0.9234	4.8614	8.49E-01
19q13.32	APOE	rs7259004	SPM 24:0	/	SPM 16:1	unclassified	-0.0929	0.0170	4.79E-08
19q13.32	APOE	rs7259004	SPM 24:0	/	SPM dih 16:0	unclassified	-0.0549	0.0831	5.09E-01
19q13.32	APOE	rs7259004	SPM 24:0	/	SPM 17:0	unclassified	-0.3778	0.1174	1.28E-03
19q13.32	APOE	rs7259004	SPM 24:0	/	SPM 18:2	unclassified	-1.9144	0.7592	1.17E-02
19q13.32	APOE	rs7259004	SPM 24:0	/	SPM 18:1	unclassified	-0.1052	0.0321	1.04E-03
19q13.32	APOE	rs7259004	SPM 24:0	/	SPM dih 18:0	unclassified	-1.1139	1.7786	5.31E-01
19q13.32	APOE	rs7259004	SPM 24:0	/	SPM 20:1	unclassified	-0.2284	0.0596	1.28E-04
19q13.32	APOE	rs7259004	SPM 24:0	/	SPM 22:2	unclassified	-1.9880	1.7405	2.53E-01
19q13.32	APOE	rs7259004	SPM 24:0	/	SPM 22:1	unclassified	-0.0388	0.0115	7.53E-04
19q13.32	APOE	rs7259004	SPM 24:0	/	SPM dih 22:0	unclassified	1.6482	2.1440	4.42E-01
19q13.32	APOE	rs7259004	SPM 24:0	/	SPM 23:1	unclassified	-0.0899	0.0275	1.06E-03
19q13.32	APOE	rs7259004	SPM 24:0	/	SPM dih 23:0	unclassified	-4.9578	2.8577	8.28E-02
19q13.32	APOE	rs7259004	SPM 24:0	/	SPM dih 24:0	unclassified	0.3069	3.3308	9.27E-01
