# Supplementary material for: Genome-Wide Association Study Identifies Novel Loci Associated with Circulating Phospho- and Sphingolipid Concentrations
Source: PLoS Genet. 2012 Feb 16;8(2):e1002490. doi: 10.1371/journal.pgen.1002490 (PMC3280968; doi:10.1371/journal.pgen.1002490)
Supplement: Table S3 — GWAS of standard plasma lipid measures in the EUROSPAN Consortium. Allele1: Effect allele; Effect: regression coefficient; StdErr: standard error of the regression coefficient. (PDF) [file pgen.1002490.s009.pdf]

Table S3

GWAS of standard plasma lipid measures in EUROSPAN consortium

	SNP	Chromosomal region	Allele1	Allele2	Effect	StdErr	*P*-value	Gene region
HDL-C	rs7205804	16q13	a	g	0.06	0.01	8.5E-20	CETP
	rs1532624	16q13	a	c	0.06	0.01	8.6E-20	CETP
	rs11508026	16q13	t	c	0.06	0.01	2.4E-19	CETP
	rs711752	16q13	a	g	0.06	0.01	6.5E-19	CETP
	rs3764261	16q13	a	c	0.05	0.01	6.9E-17	CETP
	rs247616	16q13	t	c	0.05	0.01	7.8E-17	CETP
	rs173539	16q13	t	c	0.05	0.01	8.5E-17	CETP
	rs1800775	16q13	a	c	0.06	0.01	9.2E-17	CETP
	rs1864163	16q13	a	g	-0.06	0.01	1.7E-12	CETP
	rs9926440	16q13	c	g	-0.06	0.01	1.8E-12	CETP
	rs9929488	16q13	c	g	-0.06	0.01	1.2E-11	CETP
	rs12708967	16q13	t	c	0.06	0.01	8.0E-11	CETP
	rs9939224	16q13	t	g	-0.05	0.01	7.3E-09	CETP
	rs11076175	16q13	a	g	0.05	0.01	1.1E-08	CETP
	rs7499892	16q13	t	c	-0.05	0.01	1.2E-08	CETP
	rs7203984	16q13	a	c	0.05	0.01	3.7E-08	CETP
LDL-C								
	rs445925	19q13	a	g	-0.43	0.04	9.2E-26	APOE
	rs7259004	19q13	c	g	-0.56	0.07	3.2E-16	APOE
	rs7254892	19q13	a	g	-0.55	0.08	5.3E-13	APOE
	rs1531517	19q13	a	g	-0.35	0.06	1.4E-09	APOE
	rs4420638	19q13	a	g	-0.23	0.04	2.6E-09	APOE
	rs4803750	19q13	a	g	0.33	0.06	6.4E-09	APOE
TG								
	rs2232662	10q24	t	c	5.50	0.87	2.7E-10	PDCD11
	rs964184	11q23	c	g	-0.16	0.03	1.6E-08	APOA5
TC								
	rs445925	19q13	a	g	-0.29	0.04	4.6E-11	APOE
	rs7259004	19q13	c	g	-0.41	0.07	3.9E-08	APOE
