# Supplementary material for: Genome-Wide Association Study Identifies Novel Loci Associated with Circulating Phospho- and Sphingolipid Concentrations
Source: PLoS Genet. 2012 Feb 16;8(2):e1002490. doi: 10.1371/journal.pgen.1002490 (PMC3280968; doi:10.1371/journal.pgen.1002490)
Supplement: Table S4 — Conditional analysis of the genome-wide significant loci. Effect: regression coefficient; StdErr: standard error of the regression coefficient. (PDF) [file pgen.1002490.s010.pdf]

Table S4

Conditional analysis of the genome-wide significant loci.

		Original analysis			Conditional analysis			
Lipid	MarkerName	Effect	StdErr	*P*-value	Adjusted by	Effect	StdErr	*P*-value
%LPC 20:3	rs4500751	-0.0005	0.0001	5.59E-23	HDL-C	-0.0005	0.0001	6.29E-23
%LPC 20:3	rs4500751				LDL-C	-0.0005	0.0001	2.98E-23
%LPC 20:3	rs4500751				TC	-0.0005	0.0001	9.26E-23
%LPC 20:3	rs4500751				TG	-0.0005	0.0001	2.05E-23
%LPC 16:1	rs603424	-0.0005	0.0001	6.41E-14	HDL-C	-0.0005	0.0001	1.95E-13
%LPC 16:1	rs603424				LDL-C	-0.0005	0.0001	8.48E-14
%LPC 16:1	rs603424				TC	-0.0005	0.0001	1.48E-13
%LPC 16:1	rs603424				TG	-0.0005	0.0001	4.97E-14
%LPC 18:0	rs9437689	0.0028	0.0005	4.92E-08	HDL-C	0.0029	0.0005	4.32E-08
%LPC 18:0	rs9437689				LDL-C	0.0029	0.0005	9.47E-09
%LPC 18:0	rs9437689				TC	0.0029	0.0005	1.20E-08
%LPC 18:0	rs9437689				TG	0.003	0.0005	1.44E-08
% Saturated LPC	rs9932186	-0.0068	0.0012	3.33E-08	HDL-C	-0.006	0.0012	4.01E-07
% Saturated LPC	rs9932186				LDL-C	-0.0064	0.0012	7.16E-08
% Saturated LPC	rs9932186				TC	-0.0063	0.0012	1.52E-07
% Saturated LPC	rs9932186				TG	-0.0057	0.0012	8.17E-07
PC 34:4	rs4666002	0.1366	0.021	7.48E-11	HDL-C	0.1295	0.0208	4.79E-10
PC 34:4	rs4666002				LDL-C	0.1409	0.0206	8.65E-12
PC 34:4	rs4666002				TC	0.1238	0.0197	3.15E-10
PC 34:4	rs4666002				TG	0.1079	0.0198	5.24E-08
PC 30:1	rs870288	-0.1495	0.026	9.57E-09	HDL-C	-0.137	0.0267	2.86E-07
PC 30:1	rs870288				LDL-C	-0.1434	0.0267	7.53E-08
PC 30:1	rs870288				TC	-0.1331	0.0267	6.02E-07
PC 30:1	rs870288				TG	-0.1421	0.0266	8.83E-08
PC O 32:1	rs1077989	0.1675	0.0195	9.29E-18	HDL-C	0.1532	0.0186	1.62E-16
PC O 32:1	rs1077989				LDL-C	0.1567	0.0191	2.74E-16
PC O 32:1	rs1077989				TC	0.1585	0.0185	8.84E-18
PC O 32:1	rs1077989				TG	0.1599	0.0199	1.02E-15
PC 40:3	rs12472274	0.1548	0.0273	1.52E-08	HDL-C	0.153	0.0278	3.78E-08
PC 40:3	rs12472274				LDL-C	0.1522	0.028	5.19E-08
PC 40:3	rs12472274				TC	0.1581	0.0278	1.25E-08
PC 40:3	rs12472274				TG	0.1529	0.0277	3.38E-08
PC O 42:6	rs10769780	0.0551	0.01	3.31E-08	HDL-C	0.0464	0.0099	2.86E-06
PC O 42:6	rs10769780				LDL-C	0.0515	0.01	2.86E-07
PC O 42:6	rs10769780				TC	0.0469	0.0099	2.12E-06

PC O 42:6	rs10769780				TG	0.0545	0.0101	6.76E-08
PC O 42:6	rs12423247	-0.1963	0.0355	3.09E-08	HDL-C	-0.2048	0.035	4.85E-09
PC O 42:6	rs12423247				LDL-C	-0.1949	0.0355	4.09E-08
PC O 42:6	rs12423247				TC	-0.1900	0.035	5.861-08
PC O 42:6	rs12423247				TG	-0.1991	0.0357	2.50E-08
PC O 42:5	rs17148090	-0.1303	0.0237	4.14E-08	HDL-C	-0.1138	0.0233	1.06E-06
PC O 42:5	rs17148090				LDL-C	-0.1213	0.0238	3.40E-07
PC O 42:5	rs17148090				TC	-0.1216	0.0235	2.30E-07
PC O 42:5	rs17148090				TG	-0.1236	0.0239	2.22E-07
PC O 42:5	rs17718828	0.1136	0.0201	1.49E-08	HDL-C	0.1031	0.0197	1.68E-07
PC O 42:5	rs17718828				LDL-C	0.1138	0.02	1.29E-08
PC O 42:5	rs17718828				TC	0.1224	0.0224	4.81E-08
PC O 42:5	rs17718828				TG	0.1106	0.0201	3.86E-08
PC 32:1	rs7337573	-58.4035	10.464	2.39E-08	HDL-C	-57.8278	10.2457	1.66E-08
PC 32:1	rs7337573				LDL-C	-57.7103	10.278	1.97E-08
PC 32:1	rs7337573				TC	-57.6265	9.977	7.65E-09
PC 32:1	rs7337573				TG	-58.4546	9.3558	4.16E-10
PC/LPC	rs10404486	-0.8825	0.1533	8.53E-09	HDL-C	-0.8354	0.1527	4.44E-08
PC/LPC	rs10404486				LDL-C	-0.8685	0.1541	1.76E-08
PC/LPC	rs10404486				TC	-0.852	0.1527	2.39E-08
PC/LPC	rs10404486				TG	-0.8547	0.1521	1.92E-08
%PC O 36:5	rs1424760	-0.0002	0	4.11E-08	HDL-C	-0.0002	<0.0001	1.30E-07
%PC O 36:5	rs1424760				LDL-C	-0.0002	<0.0001	1.04E-07
%PC O 36:5	rs1424760				TC	-0.0002	<0.0001	8.20E-08
%PC O 36:5	rs1424760				TG	-0.0002	<0.0001	1.65E-07
%PC 26:0	rs2945816	-0.8873	0.1584	2.15E-08	HDL-C	-0.891	0.1612	3.23E-08
%PC 26:0	rs2945816				LDL-C	-0.8751	0.1612	5.67E-08
%PC 26:0	rs2945816				TC	-0.8663	0.1613	7.77E-08
%PC 26:0	rs2945816				TG	-0.8764	0.1612	5.45E-08
%PC 36:4	rs102275	0.0117	0.0001	9.88E-204	HDL-C	0.0117	0.0004	1.23E-197
%PC 36:4	rs102275				LDL-C	0.0118	0.0004	7.99E-201
%PC 36:4	rs102275				TC	0.0118	0.0004	1.61E-200
%PC 36:4	rs102275				TG	0.0118	0.0004	1.11E-200
%PC 36:1	rs10885997	-0.0006	0.0001	7.88E-09	HDL-C	-0.0006	0.0001	2.66E-08
%PC 36:1	rs10885997				LDL-C	-0.0005	0.0001	7.21E-08
%PC 36:1	rs10885997				TC	-0.0005	0.0001	4.43E-08
%PC 36:1	rs10885997				TG	-0.0006	0.0001	2.66E-08
%PC 38:5	rs17606561	0.001	0.0002	1.49E-11	HDL-C	0.001	0.0002	1.27E-10
%PC 38:5	rs17606561				LDL-C	0.001	0.0002	1.32E-10
%PC 38:5	rs17606561				TC	0.001	0.0002	6.73E-11
%PC 38:5	rs17606561				TG	0.001	0.0002	8.07E-11

%PC O 32:0	rs964184	0.0001	0.0229	2.82E-08	HDL-C	0.0001	<0.0001	5.16E-10
%PC O 32:0	rs964184				LDL-C	0.0001	<0.0001	1.22E-10
%PC O 32:0	rs964184				TC	0.0001	<0.0001	4.05E-10
%PC O 32:0	rs964184				TG	0.0001	<0.0001	6.51E-06
%PC 32:2	rs11662721	-0.0001	0	1.36E-08	HDL-C	0.0001	<0.0001	6.58E-09
%PC 32:2	rs11662721				LDL-C	0.0001	<0.0001	2.90E-08
%PC 32:2	rs11662721				TC	0.0001	<0.0001	1.33E-08
%PC 32:2	rs11662721				TG	0.0001	<0.0001	2.93E-08
%PC 32:0	rs1061808	-0.0002	0	7.78E-10	HDL-C	-0.0002	<0.0001	2.47E-09
%PC 32:0	rs1061808				LDL-C	-0.0002	<0.0001	9.76E-10
%PC 32:0	rs1061808				TC	-0.0002	<0.0001	1.77E-09
%PC 32:0	rs1061808				TG	-0.0002	<0.0001	3.47E-09
PE 36:4	rs10468017	0.3944	0.0287	6.59E-43	HDL-C	0.38	0.0291	5.43E-39
PE 36:4	rs10468017				LDL-C	0.4012	0.0291	3.62E-43
PE 36:4	rs10468017				TC	0.3979	0.0291	1.30E-42
PE 36:4	rs10468017				TG	0.4415	0.0291	6.22E-52
%Monounsaturated PE	rs9832727	-0.0062	0.0006	5.88E-29	HDL-C	-0.0062	0.0006	8.96E-28
%Monounsaturated PE	rs9832727				LDL-C	-0.0063	0.0006	3.51E-28
%Monounsaturated PE	rs9832727				TC	-0.0063	0.0006	3.13E-28
%Monounsaturated PE	rs9832727				TG	-0.0062	0.0006	6.74E-29
PLPE 18:0/22:6	rs197770	-0.2504	0.0446	1.95E-08	HDL-C	-0.2361	0.0436	6.16E-08
PLPE 18:0/22:6	rs197770				LDL-C	-0.229	0.0442	2.19E-07
PLPE 18:0/22:6	rs197770				TC	-0.2204	0.0431	3.24E-07
PLPE 18:0/22:6	rs197770				TG	-0.238	0.0452	1.37E-07
Glu-CER 16:0	rs13106975	-0.228	0.029	4.15E-15	HDL-C	-0.032	0.0037	2.08E-18
Glu-CER 16:0	rs13106975				LDL-C	-0.0322	0.0033	1.91E-22
Glu-CER 16:0	rs13106975				TC	-0.0334	0.0033	6.62E-24
Glu-CER 16:0	rs13106975				TG	-0.032	0.0037	3.36E-18
CER 16:0	rs680379	-0.1989	0.0241	1.61E-16	HDL-C	-0.1912	0.0246	7.32E-15
CER 16:0	rs680379				LDL-C	-0.1891	0.0245	1.16E-14
CER 16:0	rs680379				TC	-0.1864	0.0244	2.22E-14
CER 16:0	rs680379				TG	-0.1976	0.0244	5.45E-16
CER 18:0	rs2304130	-0.0015	0.0003	5.85E-09	HDL-C	-0.0014	0.0003	3.80E-08
CER 18:0	rs2304130				LDL-C	-0.0014	0.0003	2.76E-08
CER 18:0	rs2304130				TC	-0.0013	0.0003	1.24E-07
CER 18:0	rs2304130				TG	-0.0015	0.0003	1.17E-08
Glu-CER 24:1	rs4485401	0.1467	0.026	1.60E-08	HDL-C	0.1437	0.0264	4.94E-08
Glu-CER 24:1	rs4485401				LDL-C	0.1442	0.0264	4.55E-08
Glu-CER 24:1	rs4485401				TC	0.1416	0.0263	7.73E-08
Glu-CER 24:1	rs4485401				TG	0.1411	0.0225	3.71E-10
SPM 22:0	rs7259004	-4.1912	0.6735	4.89E-10	HDL-C	-4.4372	0.6686	3.21E-11

SPM 22:0	rs7259004				LDL-C	-0.7022	0.5473	0.1995
SPM 22:0	rs7259004				TC	-1.7392	0.5297	0.001025
SPM 22:0	rs7259004				TG	-4.0973	0.6792	1.62E-09
SPM 23:0	rs12051548	-2.0175	0.3319	1.21E-09	HDL-C	-2.0798	0.3237	1.32E-10
SPM 23:0	rs12051548				LDL-C	-1.9746	0.2629	5.92E-14
SPM 23:0	rs12051548				TC	-1.7828	0.2487	7.60E-13
SPM 23:0	rs12051548				TG	-2.1163	0.3324	1.94E-10
SPM 16:1	rs174479	1.2149	0.1588	1.99E-14	HDL-C	1.1147	0.1522	2.40E-13
SPM 16:1	rs174479				LDL-C	1.0415	0.1347	1.04E-14
SPM 16:1	rs174479				TC	0.9822	0.1232	1.57E-15
SPM 16:1	rs174479				TG	1.1913	0.1594	7.83E-14
SPM 17:0	rs1566039	0.1813	0.0317	1.09E-08	HDL-C	0.1808	0.0315	9.66E-09
SPM 17:0	rs1566039				LDL-C	0.1326	0.0289	4.49E-06
SPM 17:0	rs1566039				TC	0.1211	0.0282	1.77E-05
SPM 17:0	rs1566039				TG	0.1901	0.0323	3.91E-09
SPM 20:1	rs7258249	0.2899	0.0236	1.09E-34	HDL-C	0.2844	0.0241	4.30E-32
SPM 20:1	rs7258249				LDL-C	0.2833	0.0241	7.32E-32
SPM 20:1	rs7258249				TC	0.283	0.0241	8.30E-32
SPM 20:1	rs7258249				TG	0.2825	0.0241	9.19E-32
SPM 14:0	rs17101394	0.0027	0.0002	3.10E-57	HDL-C	0.0028	0.0002	2.70E-59
SPM 14:0	rs17101394				LDL-C	0.0028	0.0002	1.30E-58
SPM 14:0	rs17101394				TC	0.0028	0.0002	2.75E-59
SPM 14:0	rs17101394				TG	0.0028	0.0002	2.00E-57
