# Supplementary material for: Genome-Wide Association Study Identifies Novel Loci Associated with Circulating Phospho- and Sphingolipid Concentrations
Source: PLoS Genet. 2012 Feb 16;8(2):e1002490. doi: 10.1371/journal.pgen.1002490 (PMC3280968; doi:10.1371/journal.pgen.1002490)
Supplement: Table S5 — ConsensusPathDB pathway enrichment for phospholipid related loci. Gene list: ALG14, GCKR, KCNH7, ILKAP, ITGA9, PAQR9, ELOVL2, AGPAT1, PKD2L1, PNLIPRP2, SYT9, OR8I2, FADS1, DLG2, APOA1, CDK17, PCDH20, KLF12, PLEKHH1, LIPC, ALG1, PDXDC1, CDH8, ABHD3, ZNF600. +: Pathways with P-value<0.01; *: P-value after correction for False Discovery Rate; KEGG: http://www.genome.jp/kegg/; Reactome: http://www.reactome.org; SMPDB: http://www.smpdb.ca/; HumanCyc: http://humancyc.org/; PharmGKB: http://www.pharmgkb.org/; Wikipathways: http://www.wikipathways.org; EHMN: http://www.ehmn.bioinformatics.ed.ac.uk. (PDF) [file pgen.1002490.s011.pdf]

Table S5

ConsensusPathDB pathway enrichment for phospholipid related loci+

Pathway name	Pathway size	Number of candidate genes	*P*-value	Corrected *P*-value *	Pathway source
Fat digestion and absorption	45	3 (6.7%)	0.00021	0.0022	KEGG
Lipid digestion, mobilization, and transport	48	3 (6.2%)	0.00026	0.0022	Reactome
Glycerolipid metabolism	54	3 (5.6%)	0.00037	0.0022	KEGG
Glycerolipid Metabolism	12	2 (16.7%)	0.00044	0.0022	SMPDB
Metabolism of lipids and lipoproteins	269	5 (1.9%)	0.00056	0.0022	Reactome
triacylglycerol degradation	16	2 (14.3%)	0.00060	0.0022	HumanCyc
Chylomicron-mediated lipid transport	17	2 (11.8%)	0.00089	0.0028	Reactome
Statin Pathway, Pharmacodynamics	25	2 (8.3%)	0.0018	0.0044	PharmGKB
Triacylglyceride Synthesis	24	2 (8.3%)	0.0018	0.0044	Wikipathways
Statin Pathway	31	2 (6.9%)	0.0026	0.0051	Wikipathways
Lipoprotein metabolism	29	2 (6.9%)	0.0026	0.0051	Reactome
Biosynthesis of the N-glycan precursor	30	2 (6.7%)	0.0028	0.0051	Reactome
Glycerophospholipid metabolism	113	3 (2.7%)	0.0031	0.0053	EHMN
Triglyceride Biosynthesis	35	2 (5.9%)	0.0036	0.0055	Reactome
N-Glycan biosynthesis	35	2 (5.7%)	0.0038	0.0055	KEGG
N-Glycan biosynthesis	41	2 (4.9%)	0.0052	0.0071	EHMN
