# Supplementary material for: Genome-Wide Association Study Identifies Novel Loci Associated with Circulating Phospho- and Sphingolipid Concentrations
Source: PLoS Genet. 2012 Feb 16;8(2):e1002490. doi: 10.1371/journal.pgen.1002490 (PMC3280968; doi:10.1371/journal.pgen.1002490)
Supplement: Table S6 — ConsensusPathDB pathway enrichment for sphingolipid related loci. Gene list: ATP10D, SRD5A1, FADS2, SGPP1, CNTNAP4, PLD2, LPAR2, LASS4, APOE, SPTLC3. +: Pathways with P-value<0.01; *: P-value after correction for False Discovery Rate; Reactome: http://www.reactome.org; Wikipathways: http://www.wikipathways.org; PID: http://pid.nci.nih.gov/. (PDF) [file pgen.1002490.s012.pdf]

Table S6

ConsensusPathDB pathway enrichment for sphingolipid related loci[+]

Pathway name	Pathway size	Number of candidate genes	*P*-value	Corrected *P*-value [*]	Pathway source
Metabolism of lipids and lipoproteins	269	5 (1.9%)	3.7×10^{-6}	1.0×10^{-5}	Reactome
Sphingolipid metabolism	32	3 (9.4%)	4.1×10^{-6}	1.0×10^{-5}	Reactome
Sphingolipid Metabolism	43	2 (10.0%)	0.00019	0.00031	Wikipathways
LPA receptor mediated events	67	2 (3.2%)	0.0019	0.0024	PID
