# Supplementary material for: Genome-Wide Association Study Identifies Novel Loci Associated with Circulating Phospho- and Sphingolipid Concentrations
Source: PLoS Genet. 2012 Feb 16;8(2):e1002490. doi: 10.1371/journal.pgen.1002490 (PMC3280968; doi:10.1371/journal.pgen.1002490)
Supplement: Table S7 — Genome wide significant SNPs and their associations with IMT (CHARGE Consortium, Bis et al, Nat Genet 43:940–947, 2011). Effect: regression coefficient; seEffect: standard error of the regression coefficient. (PDF) [file pgen.1002490.s013.pdf]

Table S7

Association to IMT

| SNP | Chromosome | | Risk allele | Effect | seEffect | *P*-value |
|---|---|---|---|---|---|---|
| rs9437689 | 1 | ALG14 | T | -0.001 | 0.002 | 7.34E-01 |
| rs12472274 | 2 | ILKAP | A | 0.001 | 0.002 | 5.45E-01 |
| rs1424760 | 2 | KCNH7 | T | -0.002 | 0.002 | 3.39E-01 |
| rs4666002 | 2 | GCKR | C | 0.001 | 0.002 | 7.35E-01 |
| rs197770 | 3 | ITGA9 | A | 0.001 | 0.002 | 8.31E-01 |
| rs9832727 | 3 | PAQR9 | C | 0.003 | 0.002 | 8.42E-02 |
| rs13106975 | 4 | ATP10D | T | 0.000 | 0.002 | 9.06E-01 |
| rs1566039 | 5 | PAPD7 | A | -0.001 | 0.002 | 7.78E-01 |
| rs1061808 | 6 | AGPAT1 | T | -0.001 | 0.002 | 3.79E-01 |
| rs17606561 | 6 | ELOVL2 | A | 0.003 | 0.002 | 8.01E-02 |
| rs10885997 | 10 | PNLIPRP2 | A | -0.002 | 0.002 | 2.87E-01 |
| rs603424 | 10 | PKD2L1 | A | 0.003 | 0.002 | 1.05E-01 |
| rs102275 | 11 | FADS1-2-3 | T | 0.005 | 0.002 | 7.20E-04 |
| rs10769780 | 11 | SYT9 | T | 0.001 | 0.002 | 6.62E-01 |
| rs17148090 | 11 | DLG2 | A | -0.002 | 0.003 | 5.84E-01 |
| rs174479 | 11 | FADS1-2-3 | C | 0.000 | 0.002 | 8.30E-01 |
| rs964184 | 11 | APOA1-5 | C | -0.003 | 0.002 | 1.42E-01 |
| rs12423247 | 12 | CDK17 | A | 0.008 | 0.004 | 5.34E-02 |
| rs17718828 | 13 | KLF12 | T | 0.001 | 0.003 | 6.39E-01 |
| rs1077989 | 14 | PLEKHH1 | A | 0.001 | 0.002 | 3.49E-01 |
| rs17101394 | 14 | SGPP1 | A | -0.002 | 0.002 | 3.41E-01 |
| rs10468017 | 15 | LIPC | T | -0.002 | 0.002 | 1.86E-01 |
| rs4485401 | 16 | CNTNAP4 | A | 0.001 | 0.002 | 6.47E-01 |
| rs4500751 | 16 | PDXDC1 | T | -0.002 | 0.002 | 2.48E-01 |
| rs870288 | 16 | ALG1 | A | 0.001 | 0.002 | 5.38E-01 |
| rs9932186 | 16 | CDH8 | T | -0.004 | 0.002 | 2.95E-02 |
| rs12051548 | 17 | PLD2 | C | -0.011 | 0.006 | 7.31E-02 |
| rs11662721 | 18 | ABHD3 | T | -0.003 | 0.002 | 1.17E-01 |
| rs10404486 | 19 | ZNF600 | T | -0.002 | 0.003 | 4.77E-01 |
| rs2304130 | 19 | LPAR2 | A | 0.000 | 0.003 | 9.13E-01 |
| rs7258249 | 19 | LASS4 | A | 0.003 | 0.002 | 1.17E-01 |
| rs7259004 | 19 | APOE | C | -0.009 | 0.005 | 5.41E-02 |
| rs680379 | 20 | SPTLC3 | A | -0.002 | 0.002 | 1.03E-01 |
