# Supplementary material for: Genome-Wide Association Study Identifies Novel Loci Associated with Circulating Phospho- and Sphingolipid Concentrations
Source: PLoS Genet. 2012 Feb 16;8(2):e1002490. doi: 10.1371/journal.pgen.1002490 (PMC3280968; doi:10.1371/journal.pgen.1002490)
Supplement: Table S8 — Genome wide significant SNPs and their associations with type 2 diabetes risk (DIAGRAM Consortium, Voight et al, Nat Genet 42:579–589, 2010). 95% CI: 95% Confidence Interval. (PDF) [file pgen.1002490.s014.pdf]

Table S8

Association to type 2 diabetes risk

SNP	Chromosome	Gene region	Risk allele	*P*-value	Odds Ratio	95 % CI
rs9437689	1	ALG14	T	2.33E-01	1.02	0.98-1.07
rs12472274	2	ILKAP	G	4.52E-01	1.02	0.97-1.07
rs1424760	2	KCNH7	C	1.91E-01	1.03	0.99-1.08
rs4666002	2	GCKR	C	2.90E-01	1.02	0.98-1.07
rs197770	3	ITGA9	G	8.59E-01	1.01	0.95-1.07
rs9832727	3	PAQR9	G	1.92E-01	1.03	0.98-1.08
rs13106975	4	ATP10D	G	8.32E-02	1.04	0.99-1.09
rs1566039	5	PAPD7	G	6.01E-01	1.01	0.97-1.06
rs1061808	6	AGPAT1	G	2.58E-03	1.07	1.02-1.11
rs17606561	6	ELOVL2	A	9.29E-01	1.00	0.95-1.05
rs10885997	10	PNLIPRP2	G	2.12E-01	1.03	0.98-1.07
rs603424	10	PKD2L1	A	1.00E+00	1.00	0.94-1.06
rs102275	11	FADS 1-2-3	T	2.04E-02	1.05	1.01-1.09
rs10769780	11	SYT9	T	6.07E-01	1.01	0.96-1.07
rs17148090	11	DLG2	A	4.64E-01	1.03	0.95-1.12
rs174479	11	FADS 1-2-3	C	5.25E-03	1.09	1.02-1.15
rs964184	11	APOA5	G	1.05E-01	1.05	0.99-1.11
rs12423247	12	CDK17	A	6.92E-01	1.03	0.90-1.17
rs17718828	13	KLF12	C	2.66E-01	1.04	0.97-1.11
rs1077989	14	PLEKHH1	C	8.19E-02	1.04	1.00-1.08
rs17101394	14	SGPP1	A	3.57E-01	1.03	0.97-1.09
rs10468017	15	LIPC	C	1.44E-01	1.04	0.99-1.08
rs4485401	16	CNTNAP4	G	2.95E-01	1.02	0.98-1.07
rs4500751	16	PDXDC1	C	5.38E-01	1.01	0.97-1.06
rs870288	16	ALG1	G	6.27E-01	1.01	0.97-1.06
rs9932186	16	CDH8	G	9.76E-02	1.05	0.99-1.10
rs11662721	18	ABHD3	T	1.99E-01	1.03	0.98-1.09
rs10404486	19	ZNF600	T	6.15E-01	1.02	0.94-1.10
rs2304130	19	LPAR2	G	5.31E-02	1.08	1.00-1.17
rs7258249	19	LASS4	A	3.29E-01	1.03	0.97-1.08
rs680379	20	SPTLC3	A	9.50E-01	1.00	0.96-1.04
