# Supplementary material for: Genome-Wide Association Study Identifies Novel Loci Associated with Circulating Phospho- and Sphingolipid Concentrations
Source: PLoS Genet. 2012 Feb 16;8(2):e1002490. doi: 10.1371/journal.pgen.1002490 (PMC3280968; doi:10.1371/journal.pgen.1002490)
Supplement: Table S9 — Genome wide significant SNPs and their associations with coronary artery disease risk (CARDIoGRAM Consortium, Schunkert et al, Nat Genet 43: 333–338, 2011). 95% CI: 95% Confidence Interval. (PDF) [file pgen.1002490.s015.pdf]

Table S9

Association to coronary artery disease risk

SNP	Chromosome	Gene region	Risk allele	*P*-value	Odds Ratio	95 % CI
rs9437689	1	ALG14	C	2.19E-01	1.02	0.99-1.05
rs4666002	2	GCKR	C	4.77E-01	1.01	0.98-1.04
rs12472274	2	ILKAP	G	8.98E-01	1.00	0.96-1.03
rs1424760	2	KCNH7	C	7.84E-01	1.00	0.96-1.03
rs197770	3	ITGA9	G	6.31E-01	1.01	0.97-1.06
rs13106975	4	ATP10D	G	3.32E-01	0.98	0.95-1.02
rs1566039	5	PAPD7	G	4.08E-01	1.01	0.98-1.05
rs17606561	6	ELOVL2	G	9.00E-01	1.00	0.97-1.03
rs1061808	6	AGPAT1	G	1.11E-01	1.02	0.99-1.06
rs603424	10	PKD2L1	G	2.37E-02	0.95	0.91-0.99
rs10885997	10	PNLIPRP2	G	9.61E-01	1.00	0.97-1.03
rs102275	11	FADS1-2-3	C	6.41E-01	0.99	0.97-1.02
rs964184	11	APOA5	C	8.02E-10	0.88	0.85-0.92
rs17148090	11	DLG2	G	7.72E-01	0.99	0.94-1.05
rs12423247	12	CDK17	T	3.63E-01	0.96	0.88-1.05
rs17718828	13	KLF12	C	1.89E-01	1.04	0.98-1.09
rs17101394	14	SGPP1	G	8.66E-01	1.00	0.96-1.05
rs1077989	14	PLEKHH1	C	4.61E-01	0.99	0.96-1.02
rs10468017	15	LIPC	C	5.21E-01	0.99	0.96-1.02
rs4500751	16	PDXDC1	C	9.39E-01	1.00	0.97-1.03
rs870288	16	ALG1	G	6.37E-01	1.01	0.98-1.04
rs4485401	16	CNTNAP4	G	9.36E-01	1.00	0.97-1.03
rs9932186	16	CDH8	G	2.89E-02	1.04	1.00-1.08
rs11662721	18	ABHD3	C	4.51E-01	1.01	0.98-1.05
rs10404486	19	ZNF600	C	3.99E-01	1.02	0.97-1.07
rs680379	20	SPTLC3	G	7.87E-01	1.00	0.98-1.03
