# Supplementary material for: Genome-Wide Association Study Identifies Novel Loci Associated with Circulating Phospho- and Sphingolipid Concentrations
Source: PLoS Genet. 2012 Feb 16;8(2):e1002490. doi: 10.1371/journal.pgen.1002490 (PMC3280968; doi:10.1371/journal.pgen.1002490)
Supplement: Text S1 — Extensive materials and methods. (DOC) [file pgen.1002490.s016.doc]

**Supplementary Methods**

**Ethics statement**

All studies were approved by the appropriate Research Ethics Committees. The Northern Swedish Population Health Study (NSPHS) was approved by the local ethics committee at the University of Uppsala (Regionala Etikpro¨vningsna¨mnden, Uppsala). The ORCADES study was approved by the NHS Orkney Research Ethics Committee and the North of Scotland REC. The CROATIA_Vis study was approved by the ethics committee of the medical faculty in Zagreb and the Multi-Centre Research Ethics Committee for Scotland. The ERF study was approved by the Erasmus institutional medical-ethics committee in Rotterdam, The Netherlands. The MICROS study was approved by the ethical committee of the Autonomous Province of Bolzano.

**Study populations**

The ERF study is a family-based study which includes over 3000 participants descending from 22 couples living in the Rucphen region in the southwest Netherlands in the 19th century. All descendants were invited to visit the clinical research center in the region where they were examined in person and where blood was drawn (fasting). The 820 participants included in the lipidomics studies consisted of the first series of participants [1]. The MICROS study is part of the genomic health care program ‘GenNova’ and was carried out in three villages of the Val Venosta in the South Tyrol region of Italy (Stelvio, Vallelunga and Martello). This study was an extensive survey conducted in the period 2001–2003. An extensive description of the study is available elsewhere [2]. Briefly, study participants were volunteers from three isolated villages located in the Italian Alps, in a German-speaking region bordering with Austria and Switzerland. Due to geographical, historical and political reasons, the entire region experienced a prolonged period of isolation from surrounding populations. Information on the health status of participants was collected through a standardized questionnaire. Laboratory data were obtained from standard blood analyses. Genotyping was performed on just under 1400 participants with 1334 available for analysis after data cleaning. All participants were included in the lipidomics studies. The Swedish samples are part of the Northern Swedish Population Health Study (NSPHS) representing a family-based population study including a comprehensive health investigation and collection of data on family structure, lifestyle, diet, medical history and samples for laboratory analyses. Samples were collected from the northern part of the Swedish mountain region (County of Norrbotten, Parish of Karesuando). Historic population accounts show that there has been little immigration or other dramatic population changes in this area during the last 200 years [3]. The Orkney Complex Disease Study (ORCADES) is an ongoing family-based, cross-sectional study in the isolated Scottish archipelago of Orkney. Genetic diversity in this population is decreased compared to Mainland Scotland, consistent with the high levels of endogamy historically [4]. Data for participants aged 18 to 100 years, from a subgroup of ten islands, were used for this analysis. Fasting blood samples were collected and over 200 health-related phenotypes and environmental exposures were measured in each individual. All participants gave informed consent. The CROATIA_Vis study includes 986 unselected Croatians, aged 18–93 years, who were recruited into the study during 2003 and 2004 from the villages of Vis and Komiza on the Dalmatian island of Vis [5,6]. The settlements on Vis island have unique population histories and have preserved their isolation from other villages and from the outside world for centuries. Participants were phenotyped for over 450 disease-related quantitative traits. Biochemical and physiological measurements were performed, detailed genealogies reconstructed, questionnaires of lifestyle and environmental exposures collected, and blood samples and lymphocytes extracted and stored for further analyses. Samples in all studies were taken in the fasting state.

**Lipidomics**

Lipidomics data were generated in the lipidomics laboratory of Institute for Clinical Chemistry and Laboratory Medicine, University Hospital Regensburg, Germany by validated electrospray ionization tandem mass spectrometric (ESI-MS/MS) methods published previously [7,8]. Each sample batch (about 100 samples) contained 3 quality control samples which are plasma samples with low, medium and high lipid content. The coefficient of variation of these controls were below 10% for major species and below 20% for minor lipid species. In brief, samples were analyzed by direct flow injection using a precursor ion scan of *m/z* 184 specific for phosphocholine containing lipids including phosphatidylcholine (PC), sphingomyelin (SM) [8] and lysophosphatidylcholine (LPC) [7]. A neutral loss scan of *m/z* 141 was used for phosphatidylethanolamine (PE) [9] and PE-based plasmalogens (PE-pl) and was analyzed according to the principles described by Zemski-Berry [10]. In brief, fragment ions of *m/z* 364, 380 and 382 were used for PE p16:0, p18:1 and p18:0 species, respectively. Quantification was achieved by calibration lines generated by addition of naturally occurring lipid species to plasma and internal standards belonging to the same lipid class (PC14:0/14:0, PC 22:0/22:0, PE 14:0/14:0, PE 20:0/20:0, LPC 13:0, LPC 19:0). Calibration lines were generated for the following naturally occurring species: PC 34:1, 36:2, 38:4, 40:0 and PC O 16:0/20:4; LPC 16:0, 18:1, 18:0; PE 34:1, 36:2, 38:4, 40:6 and PE p16:0/20:4. Correction of isotopic overlap of lipid species as well as data analysis was performed by self programmed Excel macros for all lipid classes according to the principles described previously [8]. Nomenclature of sphingomyelin species is based on the assumption that d18:1 (dihydroxy 18:1 sphingosine) is the main base of plasma sphingomyelin species, where the first number refers to the number of carbon atoms in the chain and the second number to the number of double bonds in the chain. The performed analysis does not always allow an exact assignment. In this case, an “O” is added to the subspecies name, e.g., PC O 36:5 and PC O 32:1. This denotes that the two species are most likely be assigned to PC species containing an ether bond (alkyl) and may constitute plasmalogens. However, we cannot exclude PC O 36:5 may be assigned to PC 35:5, an unlikely odd carbon number species. Similarly, PC O 32:1 may be assigned to PC31:1.

**Genotyping and imputation**

DNA samples were genotyped according to the manufacturer's instructions on Illumina Infinium HumanHap300v2, HumanHap300v1 or HumanCNV370v1 SNP bead microarrays. Analysis of the raw data was done in the BeadStudio software with the recommended parameters for the Infinium assay and using the genotype cluster files provided by Illumina. Genotype data for these 5 populations were imputed using MACH 1.0 software (v1.0.16) [11,12] using the HapMap CEU population (release 22, build 36) as the reference. Within each genotyping batch, only SNPs showing a call rate > 98%, MAF> 1% and HWE P-value > 10^-6^ were used for imputations.

**Statistical analysis**

As all of the studies included related individuals, testing for association between lipid and allele dosage were performed using a mixed model approach as implemented with the ‘mmscore’ option in the GenABEL software [13]. This option combines the Family Based Score Test for Association (FASTA) method of Abecasis et al. [14] and a kinship matrix estimated from genotyped SNPs [15]. Results from the 5 populations were combined using fixed-effects model meta-analyses with reciprocal weighting on standard errors of the effect-size estimates using the METAL software adjusting for the genomic inflation factor for each population [16].

Persons with measurements greater than +/-4 standard deviations from the mean were excluded from further analysis. Phenotypes that did not yield normally distributed age and sex adjusted residuals were transformed prior to analysis.

To correct for multiple testing, we adopted a Bonferroni correction for the number of phenotypes studied. Since most of the lipid values are correlated with each other, we used the number of principal components that accounted for 79% of the phenotypic variance for this correction and applied it to the classical genome-wide significance threshold (5×10^-8^).

**Pathway analysis**

Ingenuity Pathway Analysis Systems-Path Explorer tool was used for the pathway analysis. For each locus, the nearest gene and the most biologically plausible candidate gene within 300Kb were selected for the pathway analysis. Then this list of genes was checked for relationship to any of the metabolites/proteins from glycerophospholipid and sphingolipid pathways with a maximum one intermediate molecule.

The “over-representation analysis” function of ConsensusPathDB [17] was applied for identification of pathways where a set of candidate genes are enriched (over-represented). Release 20 (13.07.2011) of ConsensusPathDB was used which combines 27 different databases. All genes within 50kb of each SNP (*P-value* < 2.2×10^-9^) were first selected as candidate genes. Genes from phospholipid related loci and from sphingolipid related loci were distributed in two separate candidate lists used for over-representation analysis. The results obtained assisted in the selection of a single candidate gene for each SNP. ConsensusPathDB results for the selected candidate genes are reported in supplement tables 3 and 4.

**References**

1. Aulchenko YS, Heutink P, Mackay I, Bertoli-Avella AM, Pullen J, et al. (2004) Linkage disequilibrium in young genetically isolated Dutch population. Eur J Hum Genet 12: 527-534.

2. Pattaro C, Marroni F, Riegler A, Mascalzoni D, Pichler I, et al. (2007) The genetic study of three population microisolates in South Tyrol (MICROS): study design and epidemiological perspectives. BMC Med Genet 8: 29.

3. Johansson A, Vavruch-Nilsson V, Edin-Liljegren A, Sjolander P, Gyllensten U (2005) Linkage disequilibrium between microsatellite markers in the Swedish Sami relative to a worldwide selection of populations. Hum Genet 116: 105-113.

4. McQuillan R, Leutenegger AL, Abdel-Rahman R, Franklin CS, Pericic M, et al. (2008) Runs of homozygosity in European populations. Am J Hum Genet 83: 359-372.

5. Rudan I, Campbell H, Rudan P (1999) Genetic epidemiological studies of eastern Adriatic Island isolates, Croatia: objective and strategies. Coll Antropol 23: 531-546.

6. Vitart V, Biloglav Z, Hayward C, Janicijevic B, Smolej-Narancic N, et al. (2006) 3000 years of solitude: extreme differentiation in the island isolates of Dalmatia, Croatia. Eur J Hum Genet 14: 478-487.

7. Liebisch G, Drobnik W, Lieser B, Schmitz G (2002) High-throughput quantification of lysophosphatidylcholine by electrospray ionization tandem mass spectrometry. Clin Chem 48: 2217-2224.

8. Liebisch G, Lieser B, Rathenberg J, Drobnik W, Schmitz G (2004) High-throughput quantification of phosphatidylcholine and sphingomyelin by electrospray ionization tandem mass spectrometry coupled with isotope correction algorithm. Biochim Biophys Acta 1686: 108-117.

9. Brugger B, Erben G, Sandhoff R, Wieland FT, Lehmann WD (1997) Quantitative analysis of biological membrane lipids at the low picomole level by nano-electrospray ionization tandem mass spectrometry. Proc Natl Acad Sci U S A 94: 2339-2344.

10. Zemski Berry KA, Murphy RC (2004) Electrospray ionization tandem mass spectrometry of glycerophosphoethanolamine plasmalogen phospholipids. J Am Soc Mass Spectrom 15: 1499-1508.

11. Nothnagel M, Ellinghaus D, Schreiber S, Krawczak M, Franke A (2009) A comprehensive evaluation of SNP genotype imputation. Hum Genet 125: 163-171.

12. Li Y, Willer CJ, Ding J, Scheet P, Abecasis GR MaCH: using sequence and genotype data to estimate haplotypes and unobserved genotypes. Genet Epidemiol 34: 816-834.

13. Aulchenko YS, Ripke S, Isaacs A, van Duijn CM (2007) GenABEL: an R library for genome-wide association analysis. Bioinformatics 23: 1294-1296.

14. Abecasis GR, Cherny SS, Cookson WO, Cardon LR (2002) Merlin--rapid analysis of dense genetic maps using sparse gene flow trees. Nat Genet 30: 97-101.

15. Amin N, van Duijn CM, Aulchenko YS (2007) A genomic background based method for association analysis in related individuals. PLoS One 2: e1274.

16. Willer C, Li Y, Abecasis G METAL: fast and efficient meta-analysis of genomewide association scans. Bioinformatics 26: 2190-2191.

17. Kamburov A, Wierling C, Lehrach H, Herwig R (2009) ConsensusPathDB--a database for integrating human functional interaction networks. Nucleic Acids Res 37: D623-628.

DIAGRAM Consortium members are:

Benjamin F Voight^1,2,3^, Laura J Scott^4^, Valgerdur Steinthorsdottir^5^, Andrew P Morris^6^, Christian Dina^7,8^, Ryan P Welch^9^, Eleftheria Zeggini^6,10^, Cornelia Huth^11,12^, Yurii S Aulchenko^13^, Gudmar Thorleifsson^5^, Laura J McCulloch^14^, Teresa Ferreira^6^, Harald Grallert^11,12^, Najaf Amin^13^, Guanming Wu^15^, Cristen J Willer^4^, Soumya Raychaudhuri^1,2,16^, Steve A McCarroll^1,17^, Claudia Langenberg^18^, Oliver M Hofmann^19^, Josée Dupuis^20^**,**^21^, Lu Qi^22-24^, Ayellet V Segrè^1,2,17^, Mandy van Hoek^25^, Pau Navarro^26^, Kristin Ardlie^1^, Beverley Balkau^27,28^, Rafn Benediktsson^29,30^, Amanda J Bennett^14^, Roza Blagieva^31^, Eric Boerwinkle^32^, Lori L Bonnycastle^33^, Kristina Bengtsson Boström^34^, Bert Bravenboer^35^, Suzannah Bumpstead^10^, Noël P Burtt^1^, Guillaume Charpentier^36^, Peter S Chines^33^, Marilyn Cornelis^24^, David J Couper^37^, Gabe Crawford^1^, Alex SF Doney^38,39^, Katherine S Elliott^6^, Amanda L Elliott^1,17,40^, Michael R Erdos^33^, Caroline S Fox^21,41^, Christopher S Franklin^42^, Martha Ganser^4^, Christian Gieger^11^, Niels Grarup^43^, Todd Green^1,2^, Simon Griffin^18^, Christopher J Groves^14^, Candace Guiducci^1^, Samy Hadjadj^44^, Neelam Hassanali^14^, Christian Herder^45^, Bo Isomaa^46,47^, Anne U Jackson^4^, Paul RV Johnson^48^, Torben Jørgensen^49,50^, Wen HL Kao^51,52^, Norman Klopp^11^, Augustine Kong^5^, Peter Kraft^22,23^, Johanna Kuusisto^53^, Torsten Lauritzen^54^, Man Li^51^, Aloysius Lieverse^55^, Cecilia M Lindgren^6^, Valeriya Lyssenko^56^, Michel Marre^57,58^, Thomas Meitinger^59,60^, Kristian Midthjell^61^, Mario A Morken^33^, Narisu Narisu^33^, Peter Nilsson^56^, Katharine R Owen^14^, Felicity Payne^10^, John RB Perry^62,63^, Ann-Kristin Petersen^11^, Carl Platou^61^, Christine Proença^7^, Inga Prokopenko^6,14^, Wolfgang Rathmann^64^, N William Rayner^6,14^, Neil R Robertson^6,14^, Ghislain Rocheleau^65-67^, Michael Roden^45,68^, Michael J Sampson^69^, Richa Saxena^1,2,40^, Beverley M Shields^62,63^, Peter Shrader^3,70^, Gunnar Sigurdsson^29,30^, Thomas Sparsø^43^, Klaus Strassburger^64^, Heather M Stringham^4^, Qi Sun^22,23^, Amy J Swift^33^, Barbara Thorand^11^, Jean Tichet^71^, Tiinamaija Tuomi^46,72^, Rob M van Dam^24^, Timon W van Haeften^73^, Thijs van Herpt^25,55^, Jana V van Vliet-Ostaptchouk^74^, G Bragi Walters^5^, Michael N Weedon^62,63^, Cisca Wijmenga^75^, Jacqueline Witteman^13^, Richard N Bergman^76^, Stephane Cauchi^7^, Francis S Collins^77^, Anna L Gloyn^14^, Ulf Gyllensten^78^, Torben Hansen^43,79^, Winston A Hide^19^, Graham A Hitman^80^, Albert Hofman^13^, David J Hunter^22,23^, Kristian Hveem^61,81^, Markku Laakso^53^, Karen L Mohlke^82^, Andrew D Morris^38,39^, Colin NA Palmer^38,39^, Peter P Pramstaller^83^, Igor Rudan^42,84,85^, Eric Sijbrands^25^, Lincoln D Stein^15^, Jaakko Tuomilehto^86^, Andre Uitterlinden^25^, Mark Walker^87^, Nicholas J Wareham^18^, Richard M Watanabe^76,88^, Goncalo R Abecasis^4^, Bernhard O Boehm^31^, Harry Campbell^42^, Mark J Daly^1,2^, Andrew T Hattersley^62,63^, Frank B Hu^22-24^, James B Meigs^3,70^, James S Pankow^89^, Oluf Pedersen^43,90,91^, H.-Erich Wichmann^11,12,92^, Inês Barroso^10^, Jose C Florez^1,2,3,93^, Timothy M Frayling^62,63^, Leif Groop^56,72^, Rob Sladek^65-67^, Unnur Thorsteinsdottir^5,94^, James F Wilson^42^, Thomas Illig^11^, Philippe Froguel^7,95^, Cornelia M van Duijn^13^, Kari Stefansson^5,94^, David Altshuler^1,2,3,17,40,93^, Michael Boehnke^4^, Mark I McCarthy^6,14,96^.

**Affiliations**

1. Broad Institute of Harvard and Massachusetts Institute of Technology (MIT), Cambridge, Massachusetts 02142, USA
2. Center for Human Genetic Research, Massachusetts General Hospital, 185 Cambridge Street, Boston, Massachusetts 02114, USA
3. Department of Medicine, Harvard Medical School, Boston, Massachusetts 02115, USA
4. Department of Biostatistics, University of Michigan, Ann Arbor, Michigan 48109-2029, USA
5. deCODE Genetics, 101 Reykjavik, Iceland
6. Wellcome Trust Centre for Human Genetics, University of Oxford, Oxford, OX3 7BN, UK
7. CNRS-UMR-8090, Institute of Biology and Lille 2 University, Pasteur Institute, F-59019 Lille, France
8. INSERM UMR915 CNRS ERL3147 F-44007 Nantes, France
9. Bioinformatics Program, University of Michigan, Ann Arbor MI USA 48109
10. Wellcome Trust Sanger Institute, Hinxton, CB10 1HH, UK
11. Institute of Epidemiology, Helmholtz Zentrum Muenchen, 85764 Neuherberg, Germany
12. Institute of Medical Informatics, Biometry and Epidemiology, Ludwig-Maximilians-Universität, 81377 Munich, Germany
13. Department of Epidemiology, Erasmus University Medical Center, P.O. Box 2040, 3000 CA Rotterdam, The Netherlands.
14. Oxford Centre for Diabetes, Endocrinology and Metabolism, University of Oxford, OX3 7LJ, UK
15. Ontario Institute for Cancer Research, 101 College Street, Suite 800, Toronto, Ontario M5G 0A3, Canada
16. Division of Rheumatology, Immunology and Allergy, Brigham and Women's Hospital, Harvard Medical School, Boston, Massachusetts 02115, USA
17. Department of Molecular Biology, Harvard Medical School, Boston, Massachusetts 02115, USA
18. MRC Epidemiology Unit, Institute of Metabolic Science, Addenbrooke's Hospital, Cambridge CB2 0QQ, UK
19. Department of Biostatistics, Harvard School of Public Health, Boston, Massachusetts 02115, USA
20. Department of Biostatistics, Boston University School of Public Health, Boston, Massachusetts 02118, USA
21. National Heart, Lung, and Blood Institute’s Framingham Heart Study, Framingham, Massachusetts 01702, USA
22. Department of Nutrition, Harvard School of Public Health, 665 Huntington Ave, Boston, MA 02115, USA
23. Department of Epidemiology, Harvard School of Public Health, 665 Huntington Ave, Boston, MA 02115, USA
24. Channing Laboratory, Dept. of Medicine, Brigham and Women's Hospital and Harvard Medical School, 181 Longwood Ave, Boston, MA 02115, USA
25. Department of Internal Medicine, Erasmus University Medical Centre, PO-Box 2040, 3000 CA Rotterdam, The Netherlands
26. MRC Human Genetics Unit, Institute of Genetics and Molecular Medicine, Western General Hospital, Edinburgh, EH4 2XU, UK
27. INSERM U780, F-94807 Villejuif. France
28. University Paris-Sud, F-91405 Orsay, France
29. Landspitali University Hospital, 101 Reykjavik, Iceland
30. Icelandic Heart Association, 201 Kopavogur, Iceland
31. Division of Endocrinology, Diabetes and Metabolism, Ulm University, 89081 Ulm, Germany
32. The Human Genetics Center and Institute of Molecular Medicine, University of Texas Health Science Center, Houston, Texas 77030, USA
33. National Human Genome Research Institute, National Institute of Health, Bethesda, Maryland 20892, USA
34. R&D Centre, Skaraborg Primary Care, 541 30 Skövde, Sweden
35. Department of Internal Medicine, Catharina Hospital, PO-Box 1350, 5602 ZA Eindhoven, The Netherlands
36. Endocrinology-Diabetology Unit, Corbeil-Essonnes Hospital, F-91100 Corbeil-Essonnes, France
37. Department of Biostatistics and Collaborative Studies Coordinating Center, University of North Carolina at Chapel Hill, Chapel Hill, North Carolina, 27599, USA
38. Diabetes Research Centre, Biomedical Research Institute, University of Dundee, Ninewells Hospital, Dundee DD1 9SY, UK
39. Pharmacogenomics Centre, Biomedical Research Institute, University of Dundee, Ninewells Hospital, Dundee DD1 9SY, UK
40. Department of Genetics, Harvard Medical School, Boston, Massachusetts 02115, USA
41. Division of Endocrinology, Diabetes, and Hypertension, Brigham and Women’s Hospital, Harvard Medical School, Boston, Massachusetts 02115, USA
42. Centre for Population Health Sciences, University of Edinburgh, Teviot Place, Edinburgh, EH8 9AG, UK
43. Hagedorn Research Institute, DK-2820 Gentofte, Denmark
44. Centre Hospitalier Universitaire de Poitiers, Endocrinologie Diabetologie, CIC INSERM 0801, INSERM U927, Université de Poitiers, UFR, Médecine Pharmacie, 86021 Poitiers Cedex, France
45. Institute for Clinical Diabetology, German Diabetes Center, Leibniz Center for Diabetes Research at Heinrich Heine University Düsseldorf, 40225 Düsseldorf, Germany
46. Folkhälsan Research Center, FIN-00014 Helsinki, Finland
47. Malmska Municipal Health Center and Hospital, 68601 Jakobstad, Finland
48. Diabetes Research and Wellness Foundation Human Islet Isolation Facility and Oxford Islet Transplant Programme, University of Oxford, Old Road, Headington, Oxford, OX3 7LJ, UK
49. Research Centre for Prevention and Health, Glostrup University Hospital, DK-2600 Glostrup, Denmark
50. Faculty of Health Science, University of Copenhagen, 2200 Copenhagen, Denmark
51. Department of Epidemiology, Johns Hopkins University, Baltimore, Maryland 21287, USA
52. Department of Medicine, and Welch Center for Prevention, Epidemiology, and Clinical Research, Johns Hopkins University, Baltimore, Maryland 21287, USA
53. Department of Medicine, University of Kuopio and Kuopio University Hospital, FIN-70211 Kuopio, Finland
54. Department of General Medical Practice, University of Aarhus, DK-8000 Aarhus, Denmark
55. Department of Internal Medicine, Maxima MC, PO-Box 90052, 5600 PD Eindhoven, The Netherlands
56. Department of Clinical Sciences, Diabetes and Endocrinology Research Unit, University Hospital Malmö, Lund University, 205 02 Malmö, Sweden
57. Department of Endocrinology, Diabetology and Nutrition, Bichat-Claude Bernard University Hospital, Assistance Publique des Hôpitaux de Paris, 75870 Paris Cedex 18, France
58. INSERM U695, Université Paris 7, 75018 Paris , France
59. Institute of Human Genetics, Helmholtz Zentrum Muenchen, 85764 Neuherberg, Germany
60. Institute of Human Genetics, Klinikum rechts der Isar, Technische Universität München, 81675 Muenchen, Germany
61. Nord-Trøndelag Health Study (HUNT) Research Center, Department of Community Medicine and General Practice, Norwegian University of Science and Technology, NO-7491 Trondheim, Norway
62. Genetics of Complex Traits, Institute of Biomedical and Clinical Science, Peninsula Medical School, University of Exeter, Magdalen Road, Exeter EX1 2LU, UK
63. Diabetes Genetics, Institute of Biomedical and Clinical Science, Peninsula Medical School, University of Exeter, Barrack Road, Exeter EX2 5DW, UK
64. Institute of Biometrics and Epidemiology, German Diabetes Center, Leibniz Center for Diabetes Research at Heinrich Heine University Düsseldorf, 40225 Düsseldorf, Germany
65. Department of Human Genetics, McGill University, Montreal H3H 1P3, Canada
66. Department of Medicine, Faculty of Medicine, McGill University, Montreal, H3A 1A4, Canada
67. McGill University and Genome Quebec Innovation Centre, Montreal, H3A 1A4. Canada
68. Department of Metabolic Diseases, Heinrich Heine University Düsseldorf, 40225 Düsseldorf, Germany
69. Department of Endocrinology and Diabetes, Norfolk and Norwich University Hospital NHS Trust , Norwich, NR1 7UY, UK.
70. General Medicine Division, Massachusetts General Hospital, Boston, Massachusetts, USA
71. Institut interrégional pour la Santé (IRSA), F-37521 La Riche, France
72. Department of Medicine, Helsinki University Hospital, University of Helsinki, FIN-00290 Helsinki, Finland
73. Department of Internal Medicine, University Medical Center Utrecht, 3584 CG Utrecht,The Netherlands
74. Molecular Genetics, Medical Biology Section, Department of Pathology and Medical Biology, University Medical Center Groningen and University of Groningen, 9700 RB Groningen, The Netherlands
75. Department of Genetics, University Medical Center Groningen and University of Groningen, 9713 EX Groningen, The Netherlands
76. Department of Physiology and Biophysics, University of Southern California School of Medicine, Los Angeles, California 90033, USA
77. National Institute of Health, Bethesda, Maryland 20892, USA
78. Department of Genetics and Pathology, Rudbeck Laboratory, Uppsala University, S-751 85 Uppsala, Sweden.
79. University of Southern Denmark, DK-5230 Odense, Denmark
80. Centre for Diabetes, Barts and The London School of Medicine and Dentistry, Queen Mary University of London, London E1 2AT, UK
81. Department of Medicine, The Hospital of Levanger, N-7600 Levanger, Norway
82. Department of Genetics, University of North Carolina, Chapel Hill, North Carolina 27599, USA
83. Institute of Genetic Medicine, European Academy Bozen/Bolzano (EURAC), Viale Druso 1, 39100 Bolzano, Italy
84. Croatian Centre for Global Health, Faculty of Medicine, University of Split, Soltanska 2, 21000 Split, Croatia
85. Institute for Clinical Medical Research, University Hospital "Sestre Milosrdnice", Vinogradska 29, 10000 Zagreb, Croatia
86. Department of Chronic Disease Prevention, National Institute for Health and Welfare, Helsinki FIN-00300, Finland,
87. Diabetes Research Group, Institute of Cellular Medicine, Newcastle University, Framlington Place, Newcastle upon Tyne NE2 4HH, UK
88. Department of Preventitive Medicine, Keck Medical School, University of Southern California, Los Angeles, CA, 90089-9001, USA
89. Division of Epidemiology and Community Health, University of Minnesota, Minneapolis, Minnesota 55454, USA
90. Department of Biomedical Science, Panum, Faculty of Health Science, University of Copenhagen, 2200 Copenhagen, Denmark
91. Faculty of Health Science, University of Aarhus, DK–8000 Aarhus, Denmark
92. Klinikum Grosshadern, 81377 Munich, Germany
93. Diabetes Unit, Massachusetts General Hospital, Boston, Massachusetts 02144, USA
94. Faculty of Medicine, University of Iceland, 101 Reykjavík, Iceland
95. Genomic Medicine, Imperial College London, Hammersmith Hospital, W12 0NN, London, UK
96. Oxford National Institute for Health Research Biomedical Research Centre, Churchill Hospital, Old Road Headington, Oxford, OX3 7LJ, UK

CARDIoGRAM Consortium members are:

**Executive Committee**: Sekar Kathiresan^1,2,3^, Muredach P. Reilly^4^, Nilesh J. Samani^5,6^, Heribert Schunkert^7^

**Executive Secretary:** Jeanette Erdmann^7^

**Steering Committee:** Themistocles L. Assimes^8^, Eric Boerwinkle^9^, Jeanette Erdmann^7^, Alistair Hall^10^, Christian Hengstenberg^11^, Sekar Kathiresan^1,2,3^, Inke R. König^12^, Reijo Laaksonen^13^, Ruth McPherson^14^, Muredach P. Reilly^4^, Nilesh J. Samani^5,6^, Heribert Schunkert^7^, John R. Thompson^15^, Unnur Thorsteinsdottir^16,17^, Andreas Ziegler^12^

**Statisticians**: Inke R. König^12^ (chair), John R. Thompson^15^ (chair), Devin Absher^18^, Li Chen^19^, L. Adrienne Cupples^20,21^, Eran Halperin^22^, Mingyao Li^23^, Kiran Musunuru^1,2,3^, Michael Preuss^12,7^, Arne Schillert^12^, Gudmar Thorleifsson^16^, Benjamin F. Voight^2,3,24^, George A. Wells^25^

**Writing group**: Themistocles L. Assimes^8^, Panos Deloukas^26^, Jeanette Erdmann^7^, Hilma Holm^16^, Sekar Kathiresan^1,2,3^, Inke R. König^12^, Ruth McPherson^14^, Muredach P. Reilly^4^, Robert Roberts^14^, Nilesh J. Samani^5,6^, Heribert Schunkert^7^, Alexandre F. R. Stewart^14^

**ADVANCE:** Devin Absher^18^, Themistocles L. Assimes^8^, Stephen Fortmann^8^, Alan Go^27^, Mark Hlatky^8^, Carlos Iribarren^27^, Joshua Knowles^8^, Richard Myers^18^, Thomas Quertermous^8^, Steven Sidney^27^, Neil Risch^28^, Hua Tang^29^

**CADomics**: Stefan Blankenberg^30^, Tanja Zeller^30^, Arne Schillert^12^, Philipp Wild^30^, Andreas Ziegler^12^, Renate Schnabel^30^, Christoph Sinning^30^, Karl Lackner^31^, Laurence Tiret^32^, Viviane Nicaud^32^, Francois Cambien^32^, Christoph Bickel^30^, Hans J. Rupprecht^30^, Claire Perret^32^, Carole Proust^32^, Thomas Münzel^30^

**CHARGE**: Maja Barbalic^33^, Joshua Bis^34^, Eric Boerwinkle^9^, Ida Yii-Der Chen^35^, L. Adrienne Cupples^20,21^, Abbas Dehghan^36^, Serkalem Demissie-Banjaw^37,21^, Aaron Folsom^38^, Nicole Glazer^39^, Vilmundur Gudnason^40,41^, Tamara Harris^42^, Susan Heckbert^43^, Daniel Levy^21^, Thomas Lumley^44^, Kristin Marciante^45^, Alanna Morrison^46^, Christopher J. O´Donnell^47^, Bruce M. Psaty^48^, Kenneth Rice^49^, Jerome I. Rotter^35^, David S. Siscovick^50^, Nicholas Smith^43^, Albert Smith^40,41^, Kent D. Taylor^35^, Cornelia van Duijn^36^, Kelly Volcik^46^, Jaqueline Whitteman^36^, Vasan Ramachandran^51^, Albert Hofman^36^, Andre Uitterlinden^52,36^

**deCODE**: Solveig Gretarsdottir^16^, Jeffrey R. Gulcher^16^, Hilma Holm^16^, Augustine Kong^16^, Kari Stefansson^16,17^, Gudmundur Thorgeirsson^53,17^, Karl Andersen^53,17^, Gudmar Thorleifsson^16^, Unnur Thorsteinsdottir^16,17^

**GERMIFS I and II:** Jeanette Erdmann^7^, Marcus Fischer^11^, Anika Grosshennig^12,7^, Christian Hengstenberg^11^, Inke R. König^12^, Wolfgang Lieb^54^, Patrick Linsel-Nitschke^7^, Michael Preuss^12,7^, Klaus Stark^11^, Stefan Schreiber^55^, H.-Erich Wichmann^56,58,59^, Andreas Ziegler^12^, Heribert Schunkert^7^

**GERMIFS III (KORA)**: Zouhair Aherrahrou^7^, Petra Bruse^7^, Angela Doering^56^, Jeanette Erdmann^7^, Christian Hengstenberg^11^, Thomas Illig^56^, Norman Klopp^56^, Inke R. König^12^, Patrick Linsel-Nitschke^7^, Christina Loley^12,7^, Anja Medack^7^, Christina Meisinger^56^, Thomas Meitinger^57,60^, Janja Nahrstedt^12,7^, Annette Peters^56^, Michael Preuss^12,7^, Klaus Stark^11^, Arnika K. Wagner^7^, H.-Erich Wichmann^56,58,59^, Christina Willenborg^12,7^, Andreas Ziegler^12^, Heribert Schunkert^7^

**LURIC/AtheroRemo**: Bernhard O. Böhm^61^, Harald Dobnig^62^, Tanja B. Grammer^63^, Eran Halperin^22^, Michael M. Hoffmann^64^, Marcus Kleber^65^, Reijo Laaksonen^13^, Winfried März^63,66,67^, Andreas Meinitzer^66^, Bernhard R. Winkelmann^68^, Stefan Pilz^62^, Wilfried Renner^66^, Hubert Scharnagl^66^, Tatjana Stojakovic^66^, Andreas Tomaschitz^62^, Karl Winkler^64^

**MIGen**: Benjamin F. Voight^2,3,24^, Kiran Musunuru^1,2,3^, Candace Guiducci^3^, Noel Burtt^3^, Stacey B. Gabriel^3^, David S. Siscovick^50^, Christopher J. O’Donnell^47^, Roberto Elosua^69^, Leena Peltonen^49^, Veikko Salomaa^70^, Stephen M. Schwartz^50^, Olle Melander^26^, David Altshuler^71,3^, Sekar Kathiresan^1,2,3^

**OHGS**: Alexandre F. R. Stewart^14^, Li Chen^19^, Sonny Dandona^14^, George A. Wells^25^, Olga Jarinova^14^, Ruth McPherson^14^, Robert Roberts^14^

**PennCATH/MedStar**: Muredach P. Reilly^4^, Mingyao Li^23^, Liming Qu^23^, Robert Wilensky^4^, William Matthai^4^, Hakon H. Hakonarson^72^, Joe Devaney^73^, Mary Susan Burnett^73^, Augusto D. Pichard^73^, Kenneth M. Kent^73^, Lowell Satler^73^, Joseph M. Lindsay^73^, Ron Waksman^73^, Christopher W. Knouff^74^, Dawn M. Waterworth^74^, Max C. Walker^74^, Vincent Mooser^74^, Stephen E. Epstein^73^, Daniel J. Rader^75,4^

**WTCCC**: Nilesh J. Samani^5,6^, John R. Thompson^15^, Peter S. Braund^5^, Christopher P. Nelson^5^, Benjamin J. Wright^76^, Anthony J. Balmforth^77^, Stephen G. Ball^78^, Alistair S. Hall^10^, Wellcome Trust Case Control Consortium

**Affiliations**

1 Cardiovascular Research Center and Cardiology Division, Massachusetts General Hospital, Boston, MA, USA;

2 Center for Human Genetic Research, Massachusetts General Hospital, Boston, MA, USA;

3 Program in Medical and Population Genetics, Broad Institute of Harvard and Massachusetts Institute of Technology (MIT), Cambridge, MA, USA;

4 The Cardiovascular Institute, University of Pennsylvania, Philadelphia, PA, USA;

5 Department of Cardiovascular Sciences, University of Leicester, Glenfield Hospital, Leicester, UK;

6 Leicester National Institute for Health Research Biomedical Research Unit in Cardiovascular Disease, Glenfield Hospital, Leicester, LE3 9QP, UK;

7 Medizinische Klinik II, Universität zu Lübeck, Lübeck, Germany;

8 Department of Medicine, Stanford University School of Medicine, Stanford, CA, USA;

9 University of Texas Health Science Center, Human Genetics Center and Institute of Molecular Medicine, Houston, TX, USA;

10 Division of Cardiovascular and Neuronal Remodelling, Multidisciplinary Cardiovascular Research Centre, Leeds Institute of Genetics, Health and Therapeutics, University of Leeds, UK;

11 Klinik und Poliklinik für Innere Medizin II, Universität Regensburg, Regensburg, Germany;

12 Institut für Medizinische Biometrie und Statistik, Universität zu Lübeck, Lübeck, Germany;

13 Science Center, Tampere University Hospital, Tampere, Finland;

14 The John & Jennifer Ruddy Canadian Cardiovascular Genetics Centre, University of Ottawa Heart Institute, Ottawa, Canada;

15 Department of Health Sciences, University of Leicester, Leicester, UK;

16 deCODE Genetics, 101 Reykjavik, Iceland;

17 University of Iceland, Faculty of Medicine, 101 Reykjavik, Iceland;

18 Hudson Alpha Institute, Huntsville, Alabama, USA;

19 Cardiovascular Research Methods Centre, University of Ottawa Heart Institute, 40 Ruskin Street, Ottawa, Ontario, Canada, K1Y 4W7;

20 Department of Biostatistics, Boston University School of Public Health, Boston, MA USA;

21 National Heart, Lung and Blood Institute's Framingham Heart Study, Framingham, MA, USA;

22 The Blavatnik School of Computer Science and the Department of Molecular Microbiology and Biotechnology, Tel-Aviv University, Tel-Aviv, Israel, and the International Computer Science Institute, Berkeley, CA, USA;

23 Biostatistics and Epidemiology, University of Pennsylvania, Philadelphia, PA, USA;

24 Department of Medicine, Harvard Medical School, Boston, MA, USA;

25 Research Methods, Univ Ottawa Heart Inst;

26 Department of Clinical Sciences, Hypertension and Cardiovascular Diseases, Scania University Hospital, Lund University, Malmö, Sweden;

27 Division of Research, Kaiser Permanente, Oakland, CA, USA;

28 Institute for Human Genetics, University of California, San Francisco, San Francisco, CA, USA;

29 Dept Cardiovascular Medicine, Cleveland Clinic;

30 Medizinische Klinik und Poliklinik, Johannes-Gutenberg Universität Mainz, Universitätsmedizin, Mainz, Germany;

31 Institut für Klinische Chemie und Laboratoriumsmediizin, Johannes-Gutenberg Universität Mainz, Universitätsmedizin, Mainz, Germany;

32 INSERM UMRS 937, Pierre and Marie Curie University (UPMC, Paris 6) and Medical School, Paris, France;

33 University of Texas Health Science Center, Human Genetics Center, Houston, TX, USA;

34 Cardiovascular Health Resarch Unit and Department of Medicine, University of Washington, Seattle, WA USA;

35 Cedars-Sinai Medical Center, Medical Genetics Institute, Los Angeles, CA, USA; 36 Erasmus Medical Center, Department of Epidemiology, Rotterdam, The Netherlands;

37 Boston University, School of Public Health, Boston, MA, USA;

38 University of Minnesota School of Public Health, Division of Epidemiology and Community Health, School of Public Health (A.R.F.), Minneapolis, MN, USA;

39 University of Washington, Cardiovascular Health Research Unit and Department of Medicine, Seattle, WA, USA;

40 Icelandic Heart Association, Kopavogur Iceland;

41 University of Iceland, Reykjavik, Iceland;

42 Laboratory of Epidemiology, Demography, and Biometry, Intramural Research Program, National Institute on Aging, National Institutes of Health, Bethesda MD, USA;

43 University of Washington, Department of Epidemiology, Seattle, WA, USA;

44 University of Washington, Department of Biostatistics, Seattle, WA, USA;

45 University of Washington, Department of Internal Medicine, Seattle, WA, USA;

46 University of Texas, School of Public Health, Houston, TX, USA;

47 National Heart, Lung and Blood Institute, Framingham Heart Study, Framingham, MA and Cardiology Division, Massachusetts General Hospital, Boston, MA, USA;

48 Center for Health Studies, Group Health, Departments of Medicine, Epidemiology, and Health Services, Seattle, WA, USA;

49 The Wellcome Trust Sanger Institute, The Wellcome Trust Genome Campus, Hinxton, Cambridge, UK;

50 Cardiovascular Health Research Unit, Departments of Medicine and Epidemiology, University of Washington, Seattle;

51 Boston University Medical Center, Boston, MA, USA;

52 Department of Internal Medicine, Erasmus Medical Center, Rotterdam, The Netherlands;

53 Department of Medicine, Landspitali University Hospital, 101 Reykjavik, Iceland; 54 Boston University School of Medicine, Framingham Heart Study, Framingham, MA, USA;

55 Institut für Klinische Molekularbiologie, Christian-Albrechts Universität, Kiel, Germany; 56 Institute of Epidemiology, Helmholtz Zentrum München – German Research Center for Environmental Health, Neuherberg, Germany;

57 Institut für Humangenetik, Helmholtz Zentrum München, Deutsches Forschungszentrum für Umwelt und Gesundheit, Neuherberg, Germany;

58 Institute of Medical Information Science, Biometry and Epidemiology, Ludwig-Maximilians-Universität München, Germany;

59 Klinikum Grosshadern, Munich, Germany;

60 Institut für Humangenetik, Technische Universität München, Germany;

61 Division of Endocrinology and Diabetes, Graduate School of Molecular Endocrinology and Diabetes, University of Ulm, Ulm, Germany;

62 Division of Endocrinology, Department of Medicine, Medical University of Graz, Austria;

63 Synlab Center of Laboratory Diagnostics Heidelberg, Heidelberg, Germany;

64 Division of Clinical Chemistry, Department of Medicine, Albert Ludwigs University, Freiburg, Germany;

65 LURIC non profit LLC, Freiburg, Germany;

66 Clinical Institute of Medical and Chemical Laboratory Diagnostics, Medical University Graz, Austria;

67 Institute of Public Health, Social and Preventive Medicine, Medical Faculty Manneim, University of Heidelberg, Germany;

68 Cardiology Group Frankfurt-Sachsenhausen, Frankfurt, Germany;

69 Cardiovascular Epidemiology and Genetics Group, Institut Municipal d’Investigació Mèdica, Barcelona; Ciber Epidemiología y Salud Pública (CIBERSP), Spain;

70 Chronic Disease Epidemiology and Prevention Unit, Department of Chronic Disease Prevention, National Institute for Health and Welfare, Helsinki, Finland;

71 Department of Molecular Biology and Center for Human Genetic Research, Massachusetts General Hospital, Harvard Medical School, Boston, USA;

72 The Center for Applied Genomics, Children’s Hospital of Philadelphia, Philadelphia, Pennsylvania, USA;

73 Cardiovascular Research Institute, Medstar Health Research Institute, Washington Hospital Center, Washington, DC 20010, USA;

74 Genetics Division and Drug Discovery, GlaxoSmithKline, King of Prussia, Pennsylvania 19406, USA;

75 The Institute for Translational Medicine and Therapeutics, School of Medicine, University of Pennsylvania, Philadelphia, PA, USA;

76 Department of Cardiovascular Surgery, University of Leicester, Leicester, UK;

77 Division of Cardiovascular and Diabetes Research, Multidisciplinary Cardiovascular Research Centre, Leeds Institute of Genetics, Health and Therapeutics, University of Leeds, Leeds, LS2 9JT, UK;

78 LIGHT Research Institute, Faculty of Medicine and Health, University of Leeds, Leeds, UK
