# Supplementary material for: Genome-Wide Association Study Identifies Novel Loci Associated with Circulating Phospho- and Sphingolipid Concentrations
Source: PLoS Genet. 2012 Feb 16;8(2):e1002490. doi: 10.1371/journal.pgen.1002490 (PMC3280968; doi:10.1371/journal.pgen.1002490)
Supplement: Text S2 — Full list of phospholipids that are significantly associated to FADS1-2-3 and LIPC region SNPs. (DOC) [file pgen.1002490.s017.doc]

Full list of traits that are associated (P-value < 5 × 10^-8^) FADS1-2-3 and LIPC region SNPs

FADS-1-2-3 region (11q12.2)

%LPC18:2, %LPC20:3, %LPC20:4, %LPC20:5, %LPC22:4, %LPC22:5, ,%PC32:0, %PC32:2,%PC34:2, %PC34:3, %PC34:4, %PC36:2, %PC36:3, %PC36:4, %PC36:5,%PC38:2, %PC38:3, %PC38:4, %PC38:5, %PC40:3,%PC40:4, %PC40:5, %PC40:6, %PC42:4, %PE34:2, %PE34:3,%PE36:2,%PE36:3, %PE36:4, %PE36:5, %PE38:3, %PE38:4, %PE38:5, %PE40:4, %PE40:5, %PE40:6, %PLPE16:0/18:1, %PLPE16:0/18:2, %PLPE16:0/20:4, %PLPE18:0/18:1, %PLPE18:0/18:2, %PLPE18:0/20:4, %PLPE18:1/18:1, %PLPE18:1/18:2, %PLPE18:1/20:4, LPC20:4, LPC20:5, LPC22:4, LPC22:5, LPC22:6, PC32:0, PC34:2, PC34:4, PC36:2, PC36:3, PC36:4, PC36:5, PC38:4, PC38:5, PC38:6, PC40:3, PC40:4, PC40:5, PC40:6, PC42:4, PCO34:1, PCO34:2, PCO36:2, PCO36:3, PCO36:4, PCO36:5, PCO38:3, PCO38:4, PCO38:5, PCO40:4, PCO40:5, PCO40:6, PCO42:5, PCO42:6, PE34:2, PE34:3, PE36:2, PE36:3, PE38:3, PE38:4, PLPE16:0/18:2, PLPE16:0/20:4, PLPE18:0/18:2, PLPE18:0/20:4, PLPE18:1/18:2, PLPE18:1/20:4, pep_unsa, pc/pe, pc_alkyl

LIPC region (15q21.3)

%PE32:0, %PE32:2, %PE34:0, %PE36:1,%PE38:1, %PE38:2, %PE40:3, %PE40:4, %PE42:5, %PE42:6, %PE42:7, %PEO38:7, %PEO40:3, PC34:2, PE32:1, PE34:1, PE34:2, PE34:3, PE36:1, PE36:2, PE36:3, PE36:4, PE36:5, PE38:2, PE38:3, PE38:4, PE38:5, PE38:6, PE40:4, PE40:5, PE40:6, PE42:7, PEO40:3, pc/pe, pe_alkyl, pe_mono, pe_poly, pe_sat, pe_tot, pe_unsa, pep_alkyl, pep_poly, pep_sat, pep_unsa
